# Supplementary material for: Genome-wide characterization of the xyloglucan endotransglucosylase/hydrolase gene family in Solanum lycopersicum L. and gene expression analysis in response to arbuscular mycorrhizal symbiosis
Source: PeerJ. 2023 May 3;11:e15257. doi: 10.7717/peerj.15257 (PMC10163873; doi:10.7717/peerj.15257)
Supplement: Supplemental Information 1 [file peerj-11-15257-s001.docx]

**File S1.** Genomic sequences of tomato *XTHs.*

>SlXTH1

TTCCTCACCCCCATTGGGCCATATTCATCATTCTCTAAAAAAAGAAAAAAAGAAAAATACACAAACACTGGTCTCTGATTGGATTTGTTTTTCTCACCATGGGTATCATAAAAGGAGTTTTATTTAGTATTGTTTTGATTAATTTGTCACTTGTTGTATTTTGTGGGTATCCTAGAAGGCCAGTAGATGTGCCCTTTTGGAAAAACTATGAGCCAAGTTGGGCTAGTCACCATATTAAGTTCCTCAATGGTGGTACCACTACTGATCTTATTCTCGACAGATCTTCAGGTACTAACTACACTATGTTTTTCAATTTTACGGTCACTCAATTTATAAGTAAATAATTTTTTGAGATAATAATTATTAATTGAGTTATCGTAAACTTTACATTTTCTTTTTGTATTTGAAGGAGCTGGATTTCAGTCAAAGAAATCATATCTGTTTGGGCATTTCAGTATGAAAATGAGGCTTGTTGGTGGAGACTCAGCTGGTGTTGTCACTGCATTTTACGTAAGTATATAGTACTACTCGCGATTCGATTTATTTTTCATATTTTTCTTTATTTATTTCAAGAAAAAAACTCATAAACTGCATTTTAATACGTTATGTATATGCTTGATTTAATATCGCAAAATATTTAAATATTTTATTGTTATATTTATTTGCACCATGCTAGAAAATAGATACTACTTAGATCTGTATAGTGTTTTGTTTAACGACGTATTCTGTGTTGTACGTTATAATGACTAATTTTAGCAACACTCCTACTTTATACGAACTTAAAATCGACAATGTATAAACTCGATGTTTTTTTGTCATTTTGTTGAGAGAGTGAAGTGATAAAATATTTGATTATATATTATTTTTCAGCTGTCATCGAATAATGCAGAGCACGATGAGATAGATTTTGAATTTTTGGGGAACAGAACTGGGCAGCCATACATATTGCAGACAAATGTATTCACAGGAGGAAAAGGAAACAGAGAACAGAGAATATATCTTTGGTTTGATCCAACCAAGGGCTACCATTCTTATTCTGTTCTTTGGAATACATACCTCATTGTGTAAGTTTTTTTTTTATTATATATATAAAATTTACGTAGTATATTAATAATATAATAAATAATTTTAATGTATCTAATTAAACAGAAGCATGATATAATTTGAAATTTTTATACTTTATATATCAAGTCAAACGAATTTTAATCTTTGTTAAGTATTTTCATTATTGTTGTTTCTGTAAAGTTTTTCTTATGTGGTCCCAATATATTTCTTGGGATGCGTGCACTTGATTTCTCTACGTGGTGGTCCTACAACTACAATGTTTAGAACTAGTGTGGTAACAATTGTTGTTTAATAGTGAAAAAAAGCAAAATGCACATTTGTCTAGGAATTGTTTTAAGTAAAAAGGATAAAAGAAAGCATGAAAACTTGCAAGTTTAACATGGTATAGACATTATAATAAGAACCTTTGTCATGTGAGTATGCATGTTGGTCCTTTTGTGGTAATCAAGATTTATGTTTTTGATAAATGTATGTATGTTTTAATGTTTATTATAATATACTTCTTTTTTATTTCATCGCAAAATTTCAGTAAGATCTTGATCAAATTGATATTTATGATCTTTTAACTTTGGATATTAAATAATTGATGTGACAATTAATACAAAATTTCGAAATTTTGTGTTTAAATGTCCATTTCAAATTATTTGGTCCCACATACTTTAAGCAAGCTGGCTAATTGGTGTCCTTGCTACAGAATTTTTGTCTTCTATCTTGCACCTAAAAGTAATGTCTCCTTGTTAAATGTGTATCTTTTTTCTTTCATAACACTATTCTCCCCCTTGCTTAGAAATTTTAGTACAATGGTTGAAAAGATGTTCAAAGCAATAAACATTTTTTTTTAATCTATTTCATACATACTAGATTATTCAACATTGGGCTTTTTTCATTGGGCCTCTTACCAAATGGATTGGGCTTTGTGTTAACATTTCAGGATCTTTGTGGACGACGTTCCAATTAGAGCATTCAAAAATTCGAAAGATCTTGGTGTGAAATTTCCATTCAATCAGCCCATGAAGATATACTCGAGTCTATGGGACGCAGATGATTGGGCCACAAGAGGTGGGCTTGAGAAAACCAATTGGGCCAACGCCCCATTCACCGCGTCATACACATCGTTCCACGTGGATGGATGTGAAGCTGCCACGCCACAAGAAGTCCAAGTTTGTAACACTAAAGGCATGAAATGGTGGGATCAAAAGGCCTTCCAAGATTTAGATGCATTACAGTATAGGAGACTTCGTTGGGTTCGTCAAAAATACACTGTTTATAACTATTGCACTGATAAAGCGAGGTACCCTGTTCCACCACCAGAGTGCACTAAGGACAGAGATATTTAAAATCATAATCAAAATTAAGAGGGACTTTATGAAGAAAAAAAACTTAATATGCTTTATGTGTGAGTATTTTAATGATCCTTAAAACAAAGTGCTTTTAATTGAGCTGTATTTCCCTAATTCTTTTTGAGTGTATCATTATTGGTGGAGTCATGAGGATATTATGTATCTCATGCCAGGCCTTTCATGTCTCTTGTGTTTCATCATCATAACCATAATCATGATTGATGTATTGTAATTTATCGAACTATTTGTTACTTATCTCTTAATTAAAAATATGAATTAATACTTTTTTTATTTAAGATAATTTGACTAAATCCTGAATCTCA

>SlXTH2

GTCCATGCACTTCAAACACAAAAATATTTAAAACTTATAAAAAAAAACAATACTCAAACAACATGATCAAAACATCAAGTTGTATATTTACTTTCTTTCTTCTAATATGTTTCTTCGTAGTGGTGGCTTTTGGTGGTACTTTCGACCAAGAATTTGATGTTACATGGGGTTATGGGAGGGTGAAAATACTCGAAAACGGGCAACTTCTTACTCTTTCCCTCGATAGAAGTTCTGGCTCTGGATTTAAGTCTAAACAACAATATATGTTTGCAAAGATTGACATGAAGATCAAACTTGTCCCTGGTAATTCTGCTGGCACCGCTACTACATACTATGTAAGTTAATCATCCTTAATACTATATACATATACTATACTTTGATATATTAACATATCGTACTTTTAGGTTACTAATTCATACTCTCGATAAATGAGATAACTTATAGTTATTTCGAACATAGTTATACATTTTTCTATTACAATTTAATTTTGATCGTGAATTCAAATACAGTTATCTTCGGTGGGGTCAGCTCATGATGAGATTGACTTTGAGTTTCTTGGGAATGTAAGTGGAGAACCATATACTCTTCATACAAATGTGTATGCACAAGGCAAGGGAGATAGAGAGCAACAATTTCATCTTTGGTTTGACCCTACTAAGGATTTCCACACCTACTCTATTCTTTGGAATCCTCGAAATATCATGTAAGCATACCTTTTTATTATATATATATATATATATATATACTTAAACATTTCCTATGTAAATTAACCTTATGTTATAAAATAAAATAGATGATTGATATTTTAAAGCTTTTAGATAAGAGATCACCATGGAAACACAAACTTTCAAGATATTAATTAGGGATAAAATGAATGAGACATGTCTATCATTAATCAAAAGTCTTATCAGTAAATTTGAAAAATTAGATAGTTATATATATATGAAGCACTTTCAGATTAAATAGTTAAATAGTAGTAAAACATAAATTGGATGTTTTATCATTTCATATATTGCTAAAATAGGGGTGGGGGTCGGGGGGATAAAGTTTAGCAACTATATGAAAATGACAATATTAAAACTCATTTATTAAGAAAAGGATTTAACTTATATTCACACTAATACTGTTATGTTCTTTTTTTAGTTTAAATAAAATAAAGTTTTTTTGAAAAGTGGAACTTTCACTATAAGAAATAAGCGATACTCAAAATTCCATAACTAATAAATAAAATTTTTATGTTATTAACTACAAATCATATGAAAATTCAATTAGCTACTAGAAAATATTAAGCGATGGATATTGGCTAGGAATTACCGATAAATTCTATTGCTAATTTCATGATTTCTAGTAGTGTTTGGAGCAACAATAAATTTTGTTTTTGGGTGACCATGGGTTCGAGCTGTAGAAATAATCGGTAATATAATACTACTCTAAATTAAAGTAAACTGTCTACATCATGCTACTTGGAATGCCACCCTTTCTTAAACCCCACGTGAACGTTGGATACGTCGTGCACCAGATTGTTATTAGTTTCCTAGACACATGCTATACAAATTACTCACATATTCTTGTAATTTTATATATCAGATTTTTGGTAGATGGGACACCAATAAGACAATACAAGAATCTTGAAGCAACAAATGGAATACCTTACCCAAAGAACCAACCAATGTGGTTATACTCAAGTTTATGGAATGCTGAGGAATGGGCAACAAGAGGTGGCCTTGTAAGGACTGATTGGAGTAAAGCCCCTTTTATTGCTTCTTATAGAAACTTCAATGCCCAAACTTCCAAGAATCCCACAGCCAATTCTTGGTTAACTCAATCATTGGACAATGTTGGGTTAACAAGGATGAAATGGGTGCAAAAGAACTATATGATATACAATTATTGCACTGATACTAAACGTTTCCCTCAAGGGTTCCCTCATGAATGCACTCTAAATTAATAAGTTATTGTGTTAAAATATCCTCTATATTTATTTTTTCTTGCTAAATTGGAGTGATACGGTAAGAGTTGTTGTTGTGTGAATAAAGATATTATTATAGTTTCCAGTGGTGAAAGTCGAAACAACTTCTCGCAGAAATGGATATGATAAAGATGAGTGTGTATCAAAAGCTTAATGTATTAAACTGTTATTTTTAAAAAAGTTCGTTCTTTATACTATAAAGAAATGTATATTAGCCCACTTGAGAGTGATATTGTTTGATTGTACTTATGAAATAAGTTTGAAATATTGTTTAAGATCTTGACTTGTTCTTGTTATCTCAAAAGTATCGAGGGACACAAGTTATGCTTATCTATAATATATGGTACTCATAA

>SlXTH3

CTAATTCATCCAACAAACTTTGAAATATTAAGAATTAAACATGGCTTCTTCTTCTTCTAAATTAGTACTTGTAATGTGTTTTATGATTAGTGCTTTTGGCATTGCAATTGGGGCCAAGTTTGATCAAGAATTCGACATTACATGGGGTGATGGCAGAGCAAAAATACTTAACAATGGCGACCTCCTTACTCTCTCACTTGACAAAATCTCAGGCTCTGGTTTTCAATCCAAGAATGAATATCTCTTTGGTAAAATTGACATGCAGCTCAAACTTGTCCCAGGAAATTCTGCTGGCACTGTCACTGCTTACTATGTAAGTAATTCCCTTGATTTCTGAATCATTTTTGAGCTATCTATACGTGAATATCCCCTGAAATTAAACTCTTTTACTGCAGTTGTCATCACAAGGACCAACACATGATGAGATAGATTTTGAATTCTTGGGAAATTTAAGTGGTGATCCTTATACTCTCCATACTAATGTATTTAGTCAAGGCAAAGGAAACAGAGAACAACAATTTCATCTCTGGTTTGACCCTACTGCTGATTTCCACACGTATTCCATCACTTGGAATCCACAACGCATCATGTAAGTAGTTAAACATTCAAACTTTCTTCAAATCTAACACATTCTTTTGGGATAAATCTTGATTTGGGGTTCTCTTATTTTTATGGTTGAAACAGATTTTATGTGGACGGAACGCCAATTAGAGAATACAAGAATAGTGAATCGATTGGAGTTTCATATCCAAAGAACCAACCCATGAGGATATATTCGAGTCTTTGGAATGCTGATGATTGGGCTACAAGAGGAGGCCTTGTTAAGACTGATTGGAGCCAAGCACCCTTTAGTGCTTCTTACAGAAACTTCAGTGCTAATGCTTGCATTCCCACTTCTTCATCTTCTTGCAGTTCCAATTCTGCAGCTTCAACAAGCAATTCATGGTTGAATGAAGAGTTAGATAACACAAGCCAAGAGAGGCTCAAATGGGTGCAGAAGAATTACATGGTTTATGATTACTGCACTGATTCAAAGCGATTTCCACAGGGATTTCCAGCAGATTGTGTTCAGAATATCTGAGCATTAATAATGAAAAAATAGTGTATTACTTTAAAAACTATTGTATTGATTCTTTTATTGTTTTGTACCCATCAGTAGAAGATGCAATAATTATTGAGGATTAGAAACATCTTAGTTTTGTACTAAGTTATATAAACAATGAAATAGATACTTTTTCTTCTTCTAATT

>SlXTH4

ATGAAGGGAGTTTTAGTTGCTTTTGTTTTGATTAATTTGTCAATATTGGCAAGTTGTGGGGCTCCAAGGAAGGTAATTGATGTGCCTTTTTGGAACAACTATGAACCAAGTTGGTCTAGTCACCATATTAAGTACCTTAATGGTGGTACTACGGCTGAACTTCTTCTTGACAAATCCTCTGGTAAACGAGAATATTTTTTTTGGTATCGTTTCAGTTTATTTATCTCACTTCTATATTTAGTTTATTTAAAAAAACATACTTAATTTTCTTTATTTAATTAATTGACAAGCTCTTTAATCTTATTTTTCGACATGTTAAAGATTATAATACTATAAGAAAAATATACATGACAAATAAATTGAAACTGAGCAAATATATTGAAGCACATAGGCTTTTTCATATCTTTGACTCTCGTCACTGAAAATTCTAGATCCGCTATTAATTTTTTTTTTTGAATGGTATAGCCAATAATGTACTAATTTTGAATCTTGAATGATTGAAGGAACTGGATTTCAATCAAAGAGATCGTATCTATTTGGTCATTTCAGCATGAAAATGAAGCTTGTTGGAGGAGATTCTGCTGGTGTTGTCACTGCTTTTTATGTAAGTTAACTAGTACATATTAAAAAGAAAGAATCGCAATTTTAATTAAAATACAAAGCATCTGATTTTTTTTTTTTAAAAATAATTTGTAAGTGATTATAAGAGATCATATTCATATTCTTATACTGATGAATGTTTTTATTATCGATGATTTGTCAGTTATCATCGACTAATGCTGAACACGATGAGATAGATTTCGAATTCCTCGGGAATAGAACCGGTCAGCCATACATATTGCAGACTAATGTGTTCACAGGAGGCAAAGGAGACAGAGAACAGAGGATCTATCTTTGGTTTGATCCAACCAAGGACTTTCATTCATATTCTGTTCTTTGGAACACTTACCAAATTGCGTAAGTTGATATTTTCTTTTCGTACGAAGTGTATGGTATGTAAATCTTGTTCCTGTCTTATAAGTACTGACAATAAAAATAGTATAATAACCCAACTACTTATTTATTCTTTAATCATTTTTTAATTTTGAAACTACTAAACATTAATTAATAAAGCCAGTATGATAAAACAATCATGTCAAATGTTACTTCATCGGTTTAAAAAGGGAAAATATCCAAGTACCCCCTCAACCTATGCCCGAAATTCCAGAGACACACTTATACTATACTAAGGTCCTATTATCCCCTCTGAACTTATTTTATATGTAATTTTCTACTCCTTTTTAGCCTACGTGGCACTAGTTCAGAAAAAAAGTCAATCATCGTTGGGCCCACAAGATAGTGCCATGTAAGCTAAAAAGGGGTAAAAAAATATTTATAAAATAAGTTCAGGGGTAATAGGACCTTAGTATAGTATAAGTGTGTCTCTGAGATTTCGGGCATAGATTGAGGGGGTACTTGGGCATTATCCCGTTTAAAAAAGAATGAATTTCTTTCATTTTAGTCTATTTAAAAAAGAATGATGTCTTTTTTTTTGTAACAACTTTCCACATGACATGTTTAAGGTCACAAAATTAAAGGACAATTTTATACATTTGACATAACTTTAATTTAGGACCACATGATCAAAAGTGTTCTTTATTTTCTTAAACTATGTGTCATGTCAAACTAGACCATTCTTTGTGAAATGGAGAGAACATTTTATTAATGCGTTCCAAGCCAAATATGCAAGTATAGTGAAAGTGGGGGTGGGGTGGGGTGAGGTGGGGGTGTTATAGTATTGAAATTTTTCATTAGGATGAGAGAAAATTGCGTGACTACTTACTAACACCATATTCTAATCTTCGAATCTAAGCATTTTTCTATCGAGGGTTATGTTTTCGATGAATTAGGAAATGTGTCATCCCTTGTCAGGTTTAATAATTTCTAAAAATTAACAGGATTTTTGTGGATGATGTCCCAATAAGAGTATTCAAGAATTCAAAAGACATAGGAGTGAAATTTCCGTTCAATCAGCCAATGAAGATCTACTCAAGCCTATGGAACGCGGATGATTGGGCTACAAGAGGAGGGTTAGAGAAAACTAATTGGTCTGGGGCGCCATTCATCGCTTCCTATACTTCATTCCACATTGATGGATGTGAGGCTGTCACACCACAAGAGGTACAAGTTTGTAACACCAATGGCATGAAATGGTGGGATCAAAAGGCTTTCCAAGATTTAGATGGCCCTGAATATAGAAAACTTCATAGGGTTAGACAAAATTTCACAATATATAACTATTGTACTGATAGAAAAAGGTACCCTACACTTCCTCTAGAGTGTACAAGGGATAGAGATCTTTAA

>SlXTH5

TCTCTCAAAGCACATATAAAAACACATAGTGTGGCCTCAGAAAAAAACAGAAACAAAAAAAAATGGATTTCATCAGAATGAAAATATGTCTTTCTGTCTTATTTTTTTTCCATGTTTGGTTTTGTAGAGCTTTTAATGATGTCTCAACAATTCCTTTTAACAAAGGATTCAGCCATCTCTTTGGTGATGGAAATATTCTTCATGCTAACGATGATAACAGCCTTCAACTTCATCTCAACCAAAACACAGGTAAACATACATATATAATCAATTTCAAATTTCACTCCGTTTTGTGTTTGATACGACAGAAGTCATCAAAAATATTTGTTATACCCATAATATGTCTAAATTTTTTTTTTCTAATTATTATTTGAAATTTATATTACAGGTTCAGGGTTCAAGTCTTCTGACCTTTACAACCATGGTTTCTTCAGTGCTAAAATTAAATTGCCATCAGATTATACTGCAGGAATAGTTGTTGCCTTCTATGTATGTATACTTTAATATTTTTTTTTCAAATACTACAATTTTATCGTAGTTTCAATCAGAGTCAAGAATTCAAATCTATTTTCATAATATAATATTTCAACGAAGGGATTTTCTTTGCATCCCTCCATCTTCACCCACGGCTTTAATACGGGAGTCATCCTTTATCAGATATTATTTCTTTGTTGAAAAACTATATTGTATAAACAAATAATTTATTTTATGTACACCTCAATCTCTTTGGTGTATTTTATTTATAATTTATTTTTTTTTAATAAAAACTCTGACTTTGTTTATTTGGATGCAGACGACGAATCAAGATGTATTTAAGAAGACACACGATGAACTAGATTTTGAATTTTTGGGAAATATAAAAGGAAAAGCATGGAGATTTCAAACAAATATGTATGGAAATGGAAGCACACATAGAGGAAGAGAAGAAAGATATACTCTATGGTTTGATCCTTCTAAAGAGTTCCATCGTTATAGTATTTTGTGGACCAACAAAAACATCATGTGAGTTTCACTTGCTTTAATTTATTTTAAGTAACTTCTTTCTTTAAATTTAAGTAAAGTAAAGCATACTTTTAATAACGCGGATTCGTTGAATATAAATTTTCTCATTTTTGCGGCTTCTTTTTCTACTATTTTGACCTTTTCGAAAACACCGTTGTTACTCTTCTACTACAACTTTCGAGTATCACATGTTAGAATAATATAACTGTATCGTTTGTCTGATTTTAATTTAATAAAAAAATGAATAAAAAAAAGTATAATCTTAGATAAAGTCAAATTTTATTTAAAAATTTAATCTAAAATTCATGACGTTTTGTTGGTGAATATTTTACGCAAAATGCTTATTTAAAATGTATGGTGTGACCTTTTTTTTTTTCATTTTACGTGATGATGAAATAGTGTTTGACTGGTTCAATGAATCTATATGTTAATCATATTTTGTATCTAATTTATTCAAAGGACGACGACGTATAACTGAATTGGAAAAGTCTAAATGTTCCGTGATCAAATTGTAGATAACTATCCTAATTAATCTGCATTAAAGTAAATCATTGACAAAATGACAATTATGTCTTAATTCATGACTTGACAATTTAATTTTTTATTCTTTTTTTCCTTTCTGGATGTGAACATGAGTTTTCTTAATTACATATAGATTATGTAGTTACAATATTTTTTTAGAAAAATAAATTTGAGAGTGGGAAAATATATCAAAACATCTGATTAGGATTTACCGAAAATAACTAATTTATTTTTTGATTAGATAAGATTGAATTCTATGTAATCATTACTCTTTCCATACCCCACTTGAATTATTTTTCATTAGAAAAAAGGTACATATATATGATATCAACAAATCCACATTAAAGTAATTAATTAAACGATAATATCGTTTACAATATTGAACTTTATTATTGACAATTTAATTTGGTTGTTTTTTTGGATGTGAACGTCAAGAGTTTCTTTAATCACATAAATATCATATGGCTATAATATTTCTTTAGAAGATAAATAAATTCGAAAAGAAAAAAAATACAAAACAAATGATATGAGATTGAGATCAGTATAAATAATCGAATAAGCATTACCCTCTTCGGATTATACTAAGATTGGATATATTATAGTTGTTTGTTAACCTATATTAAAGATCCTCTTTTAATTCCAACCAATATGCTGCTATAGTTTAACCACTTTGTAATAGATTGAATTAACTAATATAAATTATTTCTCACAATATAGTGTAACCACTTTGTATTACATTGAATTGATTTAACATTATCAATTATTAATTTCTTAATTAGAAAAAAGGTACATACAACCTAATCCACATTAAACTAATTAAATGATAGATATCTCTATCAACATTTGAACCTCTCAATTATTGACAATTTTTTTATTAATTATAATTTCAATTTTGACAACAGATTTTATATAGATGATGTTCCAATTAGAGAAATTGTAAGAAATGATGCAATGGGTGGAGACTACCCATCAAAGCCAATGGGCCTATATGCAACAATTTGGGATGCTTCAGATTGGGCTACTTCAGGTGGAAAATACAAAACAAATTACAAATATGCACCATTTATAGCTGAATTTACTGATTTAGTACTCAATGGATGTGCAATGGACCCATTGGAACAAGTTGTAAACCCTAGTCTTTGTGATGAAAAAGATGTTGAACTCCAAAAGTCGGATTTTTCAAGGATTACATCGAGACAAAGAATGTCCATGAAAAGATTTAGGGCGAAATATATGTACTATTCTTACTGTTACGATTCATTGAGATACTCAGTGCCACCACCAGAGTGCGAGATTGATCCAGTTGAGCAACAACATTTTAAAGAGACTGGAAGGTTGAAGTTTATAAACAAGCACCACGGACATCGTCATCCTAAGAAAACAAAAAGTGAAGTTCTTGATGCTAGGAAGTATGGAAATGAAGATGAAGAGTGATTCAAAGTTTTGTTTACTAATAGTAATATTGTTGGTGTGTATATAGATAAAAGGAGGGAAAGAGTGAATTCGTTTATTTTTCTTGGATTATAGATTTTGTTATAGGATATATTGTCAGAGTGAAGGGTGAAAAATGATTATATATAAAGTGTGAACACTGATTGAGTAGTTATATTGTAATATAAATAAAGAGATTGAATTATGTATATTGAATTACTATTAAAGACTCCATACATTTCTTATTCTTCAAATTAAATAATCATATTTCATATAAT

>SlXTH6

ATGGAATTCCTTCTTTATTTACTTTTATTTTTCTTACTCAATTCAAGATTAATCAATGCTCAAGGTCCCCCTTCACCTGGCTACTATCCTAGTTCTAGGGCACAATCTATAGGATTTAACCAAGGTTTTAGAAACCTTTGGGGTCCTCAACATCAATCATTGGACCAAAGTACCTTAACTATATGGCTTGATAAAAATTCAGGTCTCACTTCTTCTCTTTCTTTTTTTATTTTTTTAGAAGTACGTTTAACGTCCCTCAAAATCTATAGATATGCTCTAAGACATTACTATCATGGCTACTATCAACCCGAGCCCTATGTAGTTGCTTGCTTAGAATTCTCGAAGGAATTAATGTGTATACATTGATGTTATATTGTTATCAGTTTGTGATAATAACATATAATTATGATATCAATTAAAGGTGGAGTGAGAATTTTTATTCTAAAATATAAATTTGTAGCTTTCTCTCTTACTCCGTCTTTATTATCAATTAATGCTATTATAGATACTTTTTTTTTATTTATGTAATTGTGATGTTTGTATCAATTTGCATCGTCGTATCACATTGGTTACATTTTTTCACTTTTCTTTATTACACATTTGTTTTTCTTTTTTAATTTATTCTATGTATTTGAATGCAGGAGGAAGTGGTTTTAAATCTCTAAAGAATTATCGTTCTGGTTATTTTGGGAGTAGTATTAAGCTACAACCTGGTTTTACTGCTGGAATTATTACTTCTTTTTATGTGAGTATTTAATTTAATTTGATTTATTATTACAAAAAAAAAACATTTTTAGTTTTATTGATATATTATTAATATTTATTTTGTAGCTTTCAAATAATCAAGATTATCCGGGGAACCATGATGAAATTGATATTGAATTTCTTGGAACAACACCAAACAAGCCATATACTTTACAAACAAATGTGTATATAAGAGGAAGTGGAGATGGAAATATTATTGGAAGAGAAATGAAATTTCATCTTTGGTTTGATCCAACAAAAGATTATCACAATTATGCAATCCTTTGGGACCCCAATGAGATCATGTTAGTATATTTTTTTTAAAAACTTCTTCTTTTTTTTTGCTTATGGTCTATTTTTTTTAGTCTTTTTCATAAATAGTTTAGTGTTAAAAAAATATGTTCAAAAGTAGAAATTTTTTAACGTACAGATACGTTGATAGTGTTACGAATTTCAAACTTAAGCAGTAAAACAATTTAAAATATTTTAATAATTATTGAAATTCATGACAGCGTTATACGAAATTCAAATTTGTAAAAAAAAAAAATACATAACAATGTTTTAGATTTTAAATTCTAACAATTAATTGAATTTGTGTTTATTGTTTGATTAGAAATATGAAAGTAACATTTCCAAATTGAGTGTAAAAGCTATTTTAGTACTATAGAAGATCACTCACTTTTTTATATTATATGTTAATACCAATATAGAAATTTGTGATTAATTCATAAGTGATTTTGACGGTGTAAAAATTTGTATATTTAATTCTAAATATAAATTTTAAAAATGTCACAACCATGGGGTCATGATATAATTATGCTATTTTTGTCAAAAAGCATTATTTTTCATAAATGTAACCAATATGACCCCATGTCCAAAAACAACAATAACAACTACTCTACTACTACTTTTTGTTTTTTGTTTTTTTTTTTCCTCTTGTGTGTCTTTGCTATTATTATTATTATTATTTATTTATTTGAATATGACTTTGTAGATTTTTTGTCGATGATGTCCCTATTAGAAGATACCCTAAAAAAAATGATGCAACATTTCCACAAAGACCTATGTATGTCTATGGTTCAATTTGGGATGCATCATCTTGGGCAACGGAGGAAGGACGAATTAAAGCGGATTATCGATACCAACCTTTTATCGGAAAATATAGTAATAATTTCAAGGTTGAAGGTTGCGCGGCCTACGAGAGTCCCTCTTGTCGTCGAGCGCCTTCTAGCTCTCCTTCGGGGGGTGGAGGGTTGAGTCGACAACAGATAGAGGCTATGTTGTGGGTGCATAGGAACTATAAGGTGTACGATTATTGTAGGGATCCTAGGAGGGACCATACTCACACACCTGAGTGTTAG

>SlXTH7

ACACCACAACATCCAATTATATAATTAGAAAAAAAACAATGGCCACATTGACTTGCTCTTCCTTAAAAAATTCAGCTTTTGTTCTAATATTGGTATATGCCTTGACCTTTTCATTCTCACTAGTAAGTGCACGACCCGCCACTTTTTTACAGGATTTTAAAATCGCTTGGTCCGACTCTCACATCAAACAACTCGATGGCGGCAGGGGAATTCAACTTATTCTCGATCAAAACTCAGGTATTATCCTTTTTAAAACTTTGAGTCAAAATTTCAATTTTTAATATTTTTTAACATCATCTGCCTCTGTATGTGTCATGCTCATGCATGATTCATGATAATAATGATTGTTACTTTGCTTTAAGGATGTGGATTTGCTTCGAGAAGCAAATACCTGTTTGGACGTGTTAGCATGAAGATCAAGCTCGTTCCAGGTGACTCTGCAGGAACTGTTACCGCCTTTTACGTAAGTATAAATGCAGTAGTTATTCATTAATTTGACGTTAGACATGATATTTATTGACATTCTTTTTTTTCAGATGAATTCGGACACAGATAACGTAAGAGACGAACTTGACTTCGAATTCTTGGGAAACCGGACAGGGCAGCCGTACACTGTTCAAACGAATGTTTATGTCCACGGAAAAGGTGACAAGGAACAAAGGGTTAACCTTTGGTTCGATCCATCCGCTGATTTTCACACATACACCATTTTTTGGAACCATCATCAAGCCGTGTAAGTACATGTATATATATTGTTTAAGACTACGAAATTAAAATAACATTTTTGTACATTATTTCATAACCATGATTTGTTTATTTTTTTTTAAAAAAAAAAATCGATTTTGTAGAGTATAACATGTAAGTTACTGTTTTCACTAGGTTCGGTTCAATTGATTAATGTCCATCCTTAGAAAATTACATGTAATTAATCAAATTGACTAAATATTTTTGTAGTAGTACGTACTTATGTAAAACTAATTAATTTTTTTTGTAAATTTGAAGGTTCTCAGTGGATGGAATACCCATTAGAGTGTACAAGAACAACGAAGCAAAAGGAATCCCATTCCCCAAATTTCAACCCATGGGTGTCTACTCAACATTGTGGGAAGCTGACGACTGGGCTACAAGAGGTGGCTTAGAGAAAATAAATTGGAGCAAATCCCCATTTTACGCATACTACAAGGATTTTGACATTGAAGGATGTGCAATGCCAGGACCAGCAAATTGTGCCTCCAACCCAAGTAATTGGTGGGAAGGACCTTCTTATCAACAACTGAGCCCAGTACAAGCAAGGCAATATCGTTGGGTTCGAATGAATCACATGATCTATGATTATTGCACAGACAAATCGAGAAACCCCGTTCCCCCACCAGAATGTAGGGCCGGAATTTGAAACTTCTCCTTAATTGTGTATACATGGGGGGGGGGGGGGGGGATGAAATTTGTGCCCATTGATATGGACAGTCACGTTCATGTCTTATCAATGCTTGCTCCGTTGTTATATGTACTAAGATTATAGAAAGAGTGGCCTAAAGTCAAATGTCGTTTTATTGCTGTAATATATTGTTGTCTGTATATGTAAATTGTACGTACACTCTACGAGAGCCAATAATGTTTCTTCCTGTTTGATTG

>SlXTH8

ATGGTGAATTTTCTTCTGGAAATTTTTATATTTTGCTATGTTGTTGTATTAGTTTCTGGATTTTCAGAAAATCTCGAAACGTCGTCGTTTAATGAAGGATATTCACAACTTTTTGGTCATGATAATCTTATGGTCATTCAAGATGGAAAATCAGTTCATATTTCTCTAGATGAAAGAACAGGTTCATATTTTATTTTATTTTTTCACTTATTTGACACTATTTACGTTCGAAACGGAGTCAAGAATTGAAGTTTATGAGTTAAAAATAATTAAATTGTTCAACGTTCTAGATAGGTATTTGAATTTTCTTTTTATATTCGTCCTTTTATTTATTAAAGTTTTATATATTCAAGGAGATAAGTTACACTATAAAGTTATATTTATGATTTCACAAGGGCATAGTGAAAATTATGATTTTTTTAAATAACATACTTGATTTTTAATATGTGTATTGCTTCGTAAATTCAGTTAATATGGAACAGAACTTTTTATATTGAAAGATATATTAGTGTATATTTAAGATCACATATTTTAAAATTATTTTAGTTTTACATGATTGTTACAATATATTTAAAATTATAAATTTTAAAATATTATGTCAAGTCAAAATATATTTACTTTATTTTGATGGAATTTATTCATTTGGAAATTAATGGGTTAATGTGCAGGAGCTGGATTTGTGTCACAAGACTTGTACCTTCATGGCTTATTCAGTGCTTCTATTAAATTACCAGAAGATTACACTGCTGGAGTGGTGGTTGCATTTTATGTAAGTTGAAAACAAGTAATTTTATATATGTATATTTTCGATAAATATGTTTAACTGACCACCCTTCGATTAATGTATTAACGTTAGACTATAAGTTTTGATAAGAGGGTTATATTAAGTTCACAGCCAAACTAGTATAACGTTAGACTAGTTTGCGTATATTTTATCTTCTTCAGATCTCAGGCTCTACTTGAGTATTACACTGAATATGTTATTGTTGTTAATTTTTTTTGGGGTTACTATTGACAGATGTCAAATGGAGACATGTTTGAGAAGAATCATGATGAAATTGACTTTGAGTTTTTGGGAAATATTAGAGCAAAAAATTGGAGGATTCAAACTAATATTTATGGAAATGGTAGCACAAATGTTGGTAGAGAAGAAAGATATGGACTTTGGTTTGATCCAACTGAAGATTTTCATACATATACAATTCTTTGGACTGACAGCCACATCATGTAAGTACCTAATTCAACCTCCCCTTTTAAAAGAAAACGGTTTTGGGTCAGCTTTCACACACGTCAATCTGTCATTAATCATAAAATATATGTTATCTCTCACCAGTATAAGTACTTCAGATAAGTTTGCCCAAAACTTAAGTGGATGAGGGAAATTTACCGAACATCTTTATTATCTATATTGAGATTGAAATCGTAGTCTTTCATAACCTTTTTTAGTTCATTGACTACTAGACCAGACCGTTTATGCTTTTCTTGATTTTGGTCAAGTGCTTTGCAGGAAATGATTTGTCCTGTTCTTCATTCAGTGTTAGTGACAGATGTAGCTTCGTAGCTACACGTTCAACTGAATCTAGTATTTTCAACACAATATACATGTGGAAAAAATAAATTTTATAATTTGAACTCGTAATTTCAAAAGTAGATAAATTCTTGATTCGCCTCTATGCAAATGTCATCCCTTGTTCAAGAGGTACTAAAGATGGAGTTTTTTTTTTTTTCCTTTTTGTTTGATCAACTTATATTCTAACCTTATACATCTTAGTAAAATGACATAACAGTACGGATAGGTCAAGTCTGTGTACACTCTACCTTCTCCAGACCCTATTTTGTGAGACTACGCTATGGACATGTTGTTGTTGTTATTCAACAATTGATTATACTTTGTTTGTTTTTGTGCATTTTTTTTGCAGCTTTTATGTAGATAATGTACCTATAAGAGAGATCAAGAGAACACAAGCAATGAGTGAGGACTTCCCTTCTAAGCCAATGTCTTTATATGGTACAATATGGGATGGCTCTAGTTGGGCTACTAATGGGGGTAAATACAAAGTCAATTACAAATATGCCCCTTACGTCGCGAAGTTCTCCGATTTCGTCCTCCATGGATGTGGTGTTGATCCAATTGAATTGTCTCCCAAGTGTGATATAGTCCTGGATTCTGCATCCATCCCAACTAGAATATCCCCTGACCAAAGGCGAAAAATGGAGAGGTTTCGAAACAAGTACTTGCAATATTCATATTGCTATGACCGGACACGATACAATGTTCCTCAATCTGAATGTGTGATTGATCCTAAGGAAGCTAATCGCCTCCGAGGATTCGACCCTATGACCTTTGGTGGTGTCCCTCGTCATCAGAACAAACGACACCACCAAAGGCAATCGAGGAGGGAAGATACGTCCGCGAAATAA

>SlXTH9

ATGTCTTCTAAATTTTCATCAACATTGCTTCTTCTTATTTCAATACTAATGAGTATCCAATTACTAGCCTCAGCTGGTAATTTCTATAGAGATGTAGACATAACTTGGGGCGAAGGACGCGGTAAAATACAAGAAGGCGGTAGAGGCCTTGCCCTATCGCTTGATAAACTTTCTGGCTCTGGCTTTCAATCCAAAAATGAGTACCTTTTTGGAAGATTCGATATGCAACTTAAACTCGTCCCTAAAAACTCTGCTGGCACTGTAACAACTTTCTTCGTAAGTCTTAAGGCCTCTTATTATAATTTGAACTTCATTATTCTTATTATAACATGCCATCCTATTGCTCATGATGGATTGTTTCCTCTGTTAGTTATCTTCACAAGGAGAAGGACATGATGAGATCGATTTCGAGTTCTTAGGCAATGTCTCTGGCCAGCCTTACACTATCCATACCAATGTATACACACAAGGAAAAGGAAACAAAGAACAACAATTTCATCTTTGGTTTGATCCAACTGCCGCGTTTCACACTTACACCATCGTCTGGAATCCTCATCGCATAGTGTAAGTTAACACAAAATCACCTCACAAATGTATATATATACTCTGAGTATCAGATCACCTAAAAGATAACAACATGTTAAAATATTTTCTTAAGATGAACTACATTTGATGTTACTAAATTTTTCGATATATATTCAGGTTCTTAGTAGATAACAGTCCAATTAGAGTGTTCAACAACCATGAAAGCATGGGAATTCCATTTCCCAAGAGTCAAGCAATGAAAGTATACTGCAGTTTATGGAATGCAGATGAATGGGCAACCCAAGGAGGCAGAGTGAAAACAGATTGGGCACTTGCACCATTCACTGCTTATTACAGAAACATTAACATTGATGGTTGTGCAGTATCATCAGGTAACCTCTTCATGTAAGTCCATCGGTTCAATAAACAACGCGAAGCCATGGCAAACACATGAACTTGATGGTAAGGGACGGAATAGACTACGATGGGTGCAGACCAAACACATGGTTTACAATTACTGTGCTGATTCTAAGAGGTTTCCACAAGGCTTTTCTGCTGAATGCAAGAGTTCAAGATTTTAA

>SlXTH10

ACATTCACAATAACAAATTCTAATTTATCCCTTTAAATCACTACTCTAATTTTCTATAATGTTGCTGCAGCTTTCTCTTCTTACACTAGTCTTACTATCCCCTGTTTCCGCTGATAATTTCTACCAAGACGCGGCGGTCACGTTTGGTGACCAGCGCGCTCAGATACAAGATGGAGGGCGCCTTCTCACATTGTCACTTGATAAAATTTCAGGTTCCGGATTTCAGTCTAAGAATGAGTATTTATTCGGAAGGTTCGATATGCAGCTTAAACTCGTACCTGGAAATTCTGCTGGCACTGTCACCACATTCTATGTAAGTAATAACACACTTTTCGTATTCCTACTATAACTAATTAAACAAAAACAAGAACATGTTGACTCAAGTATGTTTTTTTTTGGGTGTAATTTGCAGTTGTCTTCTCAAGGAGCAGGGCATGATGAAATTGATTTTGAGTTTCTAGGAAATTCATCAGGACTACCTTACACGGTTCATACCAATGTTTACTCTCAAGGAAAAGGCAATAAAGAACAACAATTTCGTCTCTGGTTTGATCCAACTTCGTCGTTCCACACTTACTCTATTGTTTGGAACTCTCAACGGATCATGTACGTAACAAGATAAACAAAACAATTACAAATATTTACTGATATATAATGTCTGACATTTTGGGGATTTTTTTTTCAGATTTTTGGTGGATAATATCCCAATTAGAGTGTTCAACAACCACGAAGCACTTGGTGTTGCATACCCAAAGAATCAAGCAATGAGAGTTTACGCGAGTCTATGGAATGCTGATGATTGGGCTACACAAGGAGGACGGGTGAAGACAGATTGGTCTATGGCTCCGTTTACAGCTTCTTACAGGAATTTCAATACAAATGCTTGTGTTTGGTCAGCTGCTACGTCTACTTCGTCTTGTGGAGGTTCTAAGACTGAGTCAGTAAACAATGATGAGACATGGCAAACGCAACAACTGAACGCTAATGGAAGAAATAGAATACGATGGGTTCAGCAGAAGTACATGATCTACAATTACTGTGCAGATGCTAATAGGTTCTCTCAAGGCTTTTCTCCTGAATGCAAGCGTTCAAGGTTCTAAGGCGGATATATATAGTATATGAATGTAAAATTATGTTTGTTTCACTTTTCTATTCTTTTAATTTTGATCAGGTAAAAAAAAGAACATAGTGTAATTATTTGTGTATGCAATATATTCTTTATTCTTTTTGTAATCATGAAATAGAAATAATAATGAATTGTTTTCCTGACAAGCAT

>SlXTH11

ATGTTGCTGCAGCAGCTATCTGTTCTTGCTCTACTTCTCTTGCTATGTCCTGTTTGGGCTGACAATTTCTACCAAGATGCAACGGTTACCTTTGGTGATCAGCGAGCTCAGATACAAGATGGTGGGCGCCTTCTCGCCTTGTCCCTTGACAAAATTTCAGGTTCAGGATTTCAGTCTAAGAATGAATATTTATTTGGAAGGTTCGATATGCAGCTCAAACTAGTACCTGGAAATTCTGCTGGCACTGTCACTACCTTCTATGTAAGTTAACACAGTTTTCTCCTCTTATTCTGTATATGTAACTAATTAACAAACCAAAAAAAAAACAAAAAAACACATGTTCACTCACTGAAGTGTTTTTTGTGCAATATGAAGTTGTCTTCTCAAGGAGCAGGGCACGACGAAATTGATTTTGAGTTTCTGGGAAATTCATCAGGCCAACCGTACACGGTTCATACTAATGTCTACTCTCAAGGAAAAGGCAACAAAGAACAACAGTTTCGCCTATGGTTTGATCCCACCTCGTCGTTCCACACCTACTCTATTGTTTGGAACTCTCAACGCATCATGTATGTTACTAATTGAACTTTAATTGAAGTCTAAAAACAACTTACTTGGTGCTTATGTCTGACATTTGGGGGATTTTTCTTTTTCTAACTGCCCAGATTTTTGGTGGATAATATCCCAATAAGAGTATTCAACAACCACGAAAAGCTTGGTGTTGCATTCCCAAAGAACCAAGCAATGAGAGTTTATGCCAGTTTATGGAATGCTGATGACTGGGCAACACAAGGAGGGCGAGTGAAGACGGATTGGTCAATGGCTCCGTTTACAGCTTCTTACAGGAATTTCAACACAAATGCTTGTGTTTGGTCAGCTGCATCGTCTACTTCGTCCTGTGGAGGCTCTAAGACTGATTCAGTAAACAATGATCAGGCATGGCAAACTCAAGAACTGAACGGTAATGACAGAAATAGGCTTCGATGGGTTCAGCAGAAATACATGATCTACAATTACTGTGCAGATGCTAAAAGGTTCTCTCAAGGCCTTTCTCCTGAATGCAAACGTTCAAGGTTCTAA

>SlXTH12

TCACACACAAGCAAAAAATCATATATTATTCAAAGTTCAAGTAAAAAATTTTAACTACAAATGGGGTCTTTTACCCATTATGGGTTCTTGATGTTAGCACTTTTATTTAGTTCTTGCATGGTTACTTATGGTGGAAATTTTTATCAAGAATTTGACTTCACTTGGGGTGGCAATAGAGCCAAGATTTTCAATGGAGGTCAACTTTTATCTTTATCTTTAGACAAAGTTTCTGGCTCTGGTTTTCAATCAAAAAAAGAACATCTCTTTGGAAGAATTGATATGCAAATCAAACTCGTTGCTGGAAACTCTGCTGGCACTGTCACAACATATTACGTAAGTACGATTAAATTTCACATATTGATGCTGAACTTAAACTATTATAACATTATAGAATGTTTGTGAAAGCTACTTGTAGTCCAATTATTTGTCTTTCAAGTAAGGGCACTTCAAAATATTTGCAATTTTGAGAAATTTTTTGGAAGTCATGTCATATTTATGAAGTTATTATATTATTCACAAGCAACTTGTTTAATTTTACATGTAGAAATAACCACAAATATGTAACAACTTGGTTGGTACTCATGTGACATATTTTTTTTATATGGCCAAGAGCTTTTCTATTTTGGATGTTATAAGCAACTTTTTATGTGTGATAATAATGACTTCTTGATTCCCTATTCCTTTTCCTTTTATTTTGATTCCTCAAAGCATATTACATTGTCACACTTTACAAGTTGTCAACTTCTTTTTCTATAATAGAATTATACCCATCTTAATTTATTCTAAGTTTTACTTGTCAAAACATAAAAAATATTGACATAATCAGATTACTGATAACATATAGCTATAAGTACACTTTTGAAACTATGCTAATACTGTCATACTTATATTTATTGCTTGAACCTTTCTAAAATACTATTATACTTATATTTGTTACTTTTTAAAAAGTTCGAGCAACATAATTGATGACTAGAGTTTAATGTTACTTCAATTTGGAAATTAATAAAATCTAAGTAGCATTTTTACTAATTTTTTTTTTAATTGCAGTTATCTTCTCAAGGACCCACTCATGATGAAATTGACTTTGAGTTCTTGGGAAATGTTACTGGTGAACCTTATATTCTACACACAAATATTTATGCCCAAGGCAAGGGTAACAAAGAGCAACAATTTTACCTTTGGTTTGACCCTACAAAGAACTTCCACACCTACTCCATCATTTGGAAACCCCAACACATCATGTAAGCAAATACCTTAACATTATATATAAGTATTAAAAACCTCTATTATATGCTTCGGAGAAATAAGAAAATTAATCTCACGTATGATCACTGAATTTTATTCACTTAACTATATATACTTACATGTCTAAACACTATTCTTCTTTTTTTCAAGTATCAATAGTTTCATGTCTGAACGCATAACTATTTGTTCTCTATTGCGTTAAAGTAACTGAACTCGAATACAAAATTTCCTATCATAGTGACATTTATTCATTTATAATTTAATTTATTTCAGATTTTTGGTGGACAACACACCAATAAGAGTATACAAAAATGCTGAATCAGTTGGTGTACCATTTCCAAAGAATCAGCCCATGAGGATTTACTCAAGCCTTTGGAATGCTGATGATTGGGCCACAAGAGGAGGCCTAGTAAAAACTGATTGGGCCCAAGCCCCATTCACAGCCTACTATAGAAACTACATGGCCCAAAGCTTTAGCCCATCACAATTTTCTGATCAAAAATGGCAAAATCAAGAACTTGATTCTAATGGCAGAAGAAGACTTAGATGGGTTCAAAAGAATTTCATGATTTATAATTATTGTACTGATATTAAGAGGTTTCCTCAAGGTTTTCCTCCAGAATGTAGAAGATTTTGAGAGGGTTATGTAGTTTTTTTTTTGTTTTTTTTTTTTTGGGTGAAATTCTTTCATGTGTTTGTGGTTTTATTTTGATAGATTGTTAGCCAACTAAAATAAATTAATATGTTTTTTCTTTGTTTTATTTTGTATGTTATTTGAAGGTAGCTAGTAGTTTATTTTGTATCTATTTTATTTGATATCCTTTTTAGTA

>SlXTH13

ATGGCATTATTTTCATCAAGAAATTCATCAAGATCTAGGTCCTCTCTTCCATATTTGGTGTTTCTCTTAATTGCTGCCTTTTTTGTCTTCAAGGTACTCTTCTATTTCTTTAATTAATTAATTAATTATTCATCATCTCAAAAATTGAATATATATTTGTTGATTAATGTTCAGTATTGAAAAAACATCAAATTTTAATGAATAAATTTTGTAAGTAGTAATTGAAAATTGACTTGTCGGAAATATTTCTGTGAGTTATAATTGATGGTGTATTTCGATAGGAACTTTTGGAATACCCCTTAAATAGTGGTTAAACATGCAATGTTTAATGTTGAAAAAATATTAAGTTCTGATAAATAACTTTTGATAGAACAAATTGAAAATTTGACTTGTCGAAAATGTTTCTGATAGCTATTTGATGGTATTTCGATAAGAACTTTTGAACCCCTTTATAAATAGTGGTTGAAGAAGTATTGGCATGAATTTAAATTTTGTTATTCAATGCTTAATACTGAAAAAGAAAACCATTAAGTTTAATTTTAACGAATAACTTTCGATAGAACTAATCGAAAATTGACTTGTCAAAAATATTTCTTTCAAGCTATTTGATGGAATTTTGCTAGAAACTTTCCACTCCCCTTATAAATAGTGGTTAAACCTACATTGACATGAATTTGGATTTTGTTATTCAATGTCCAATATTGAAAAAATCATCAAAATTCAACGGATAACTTTTGATAGAACTAAAAAAAATTGACTTGTCGAAAATATTTCTGCTAGCTATTTGACGGAATTTCGATAAGAACCTTCAGACCCCTTGAATAGTGGTTAAGCATACATTGACATGAATTTGAATTTTATCATATCATACAATGTTCAATATTGAAAAAACATCAAATTTTCAACGAATAACTTTCGATAGAATTATCCAATTAAAAAATTAACTAGTTTCGAAATATTTTTAATGATTTTTTTCGATTACATGAGCAGGTAGATATACTCATATCTCAGTCTTTTAGTTCAGCCCGTCGCAACCTGGAAAAAACTCCTAATCGTATCGTTGTGAACCCCCAAAAATCATCGGAAGAACGTGTTGTTGACAGGTACAAAAATATTTTTTTATTTTTCTTATAAAATGAATAAATAATATTTGTACAATTATAATTCATCTTATTAGGAGTAAAAAAATAAAATTTCAAGTTAGATTATATTTACGTATAAAAATAATATCATTTTTCACGACATAATATCAATAATAGTGACTAACAAATATAACATGAGAGAACTATTCACTTATGTGACATATATAATGAGCTTTGAAAGAGGGAAGTATTTGAAATATAAATTTGGCTTAAATTATTAATATCATTCTCGAATTATTAAAAAAACATCTTTTTACTTGATTAATTAAACTTAGATATACACCCGATCTTGCAGTAATATGAGTGAAATACATTATTAAATTCTCGTCAAGTTTAGAGCTGCTTTCGGTACTTTTCTCGACTTTTCATTAAGAGTCACATGCTCTTACAAGAGTTTGAAACCACTTGATATGCCATGTTGTATATCGGGAGTATATCTAAGTTTAGTTAGCCAAGTAGAACGGTATATATAAGACTGTCAATAGTTTGAAACAAAATTAATAATTTACATCAAGCTCGAAGGTGTTTTCAATACTCATCTCACTTTAAAATAAAATAAATAAGAGAATTTAATTTATATACATCCATAAAGTTCAAAATATGAATTTTCTTCCTTTTTTTTTAATGGCAAACAACTAATTTTTTTTTTTATTTTGAAAATTTGCTGCAGCCTCCCTGTAGTTTTAGTAAATGGTACATTTGACCAGCATATTATGATATCATGGGGAGATGACAGAGGAAAAATACTTGAAAATGGAGAGCTTTTAACACTCTCCTTAGACAAGAAGTCTGGATCAGGCTTTCAGTCTAAAAAAGAGTACCTCTTTGCTAAAATTGATATGCAAATTAAGCTCGTCCCTGGAAATTCTGCTGGCACTGTTACTACGTTTTACGTAAGCCTTCTTTACTAACCTTTGCATGAAAAAAAAATCGATTTTTCTTATGATATACTCTTGGTCCTTCAATTATTGATATATTATTTGACATGTGTCCTATCGGTCCATTTATATGAAAAATATGACATATTTAGAAAAAAAAATACAAATCATGTTATCATCAATTATAGAGTATTAGTATAAACTTAACATAACAGATGCTTAATAACATAGTGGAGCATTCAACAATTATGTTATACTTTTGGTCCTTCAATTATTGATGTATTATGATTTTGGTCCTTGTATTATCTGGTTAAGCATCTTTGACCTTCGATCAATGGAAATGTTTCTTATCGATCCATTTATGAGAGAATTATGATCTATTGAGAAAAATTTTCGAATAATGTTATCATCAATTATAGAGTATCAGTACAAACTTAACGTAACATATCCTTAATTATATAGTGGAGCATTTAACAATTATTGATATATATGACTTTGGTCTTTGTGTTAGCTTATTAAGTACATTTGACCTTCAATCCACGGAAATGTGTCTTATCGATCAATTTATGTGAGAAGTATGACATATTTGAATAATTTTTCGAATTATGCAATCATCAACTATATGAAGTAACAATACAAACTTAACGTCTTTTTTTTTTCTTTTGTTTGTAGCTATCATCACAAGGGAACAAGCATGATGAAATAGATTTTGAATTCTTGGGGAATTCAACAGGGAACCCTTATACTCTTCATACAAATGTTTTTAGTTTAGGCAAAGGCAATAGGGAACAACAATTCTTCTTGTGGTTTGATCCAACTGCAGATTATCACACATATTCAATCCTATGGAATTCTAAATGTATTATGTAAGTACTTTTATTATATTTGTCTCTGTTCATTTTTACTTATTTACTATACTAACTTTCTTGCTTTACGTTTCTCTTAGTATAACTCTCTTCGCCGGTCTCTACTTGTCACTAATTGTTCAACAATATTTGTTCAGTTTGGAAAATCAAAAGATAATTTATCGTTTTTTTTCTATTTTACCTTGTTGTTAAATGTTAGTCAATATCTAATACGTGTCTTAAATTTAATATATTTAAAAGATAATATAGTAATATTACTTGTGTTACTTACTGTTTCTTGAGGTGCGTGTCAAGTCAATATTAAATAATATTATTGGACAGAGGGAGTACGTTTACTTTGCTTTAGAAAATTCCATTTCAAAGTTTTTTTTTTCACTTGAAGTTTACCTAAACTAGTTAATTACATAATCCATTTTCATATCAATGAGTGAGATGACAATTTCTATGATCTCGGGATAATGCACAAATACTTCCTCAACTTTTGTTCGTAATCTCAGAGACACATTTATACTATACTAAGGTCCTGTTACCTTCTTGAACTTATTTTATTAATAATTTTCTATCCCTTTTCGGCTTACGTGGCACTATTTTGTGGGCCCAACGCTGGTTGACTTTTTTTTCCCAAGTTTGTGTCATGTAGGACGAAAAGGGGTAGAAAATTATTTATAAAATAAGTTCAGGGGAGTAATAAGACCTTAGTATAGTATAAGTGTGTCTCTGGAATTTCGGGCATAGGTTGAGGGGGTATTTGTACATTTTTTCTATAATCTTTATCGATGCTTATTTCATGAAATAGTTTTGAGGTTGAGTTATGCTCAACATCCATTATTTTTCATGATTTTATAATAAGTAGAATGCCAATTCATCTCCTTATATGATCTTCGATCCTTACCCGTTTGAGCTAGCTATTGAAATATTGATTGCAATATCAAAAGTATTACATTTTCTTATGAGATATTTCCTCATTTTTGTTAATGTAGATTCTATGTTGATGATATACCAATTAGAGAATACAAAAATCCAGAGAGACTTGGTCTTTCATATTTAAAATACCAACCAATGAGACTATACTCAAGTCTATGGAACGCAGATGATTGGGCTACACAAGGTGGTCGTATCAAAACCAATTGGGAACTAGCACCTTTTGTAGCGTCCTACAAAAATTTCACATATGAAGCTTGTATTTATTCAAGATTAACTAGTTCATCTTCATGTGATATCGACTCTCCAACTCCTATCAACAACGCTTGGTTAACATATGAGTTAGATCGAACAAGTCGTGTTAGAATGAAAGCTTTGCAGAAAAAACATATGATTTATGATTATTGCAACGATAAATGGAGATTTCCTAAAGGTCCTGCTCCTGAATGCAAGCTTCTTCAATAA

>SlXTH14

ATGTCAACAATTTTTTTCCTTCCTATTTTTCTTTGTTTTATTTTTCTCCATTCAACTAATGCTAATTATTGGCCAATTTCACCTGGATATTATCCAAGTACAAAATTTAAATCCATGAGTTTTTATCAAGGATTTAAGAATCTCTGGGGTCCTAATCACCAGAGTGTAGATAACAATGGCATTAATATTTGGCTTGATAGAAATTCAGGTTTAATTTTCTTTAATTATTCTTGATTAGTAGTTTTACTACTTAATTTAATTTATTTTTTAAAAAAATATATATTTTTAATGATTATTTTGTATAGGCAGTGGATTCAAGTCGGTTAAACCGTTTCGATCCGGGTATTTTGGGGCTTCTATTAAACTCCAACCCGGTTATACGGCCGGAGTCATCACCGCTTTCTACGTAAGAATTATTTTTTCAGACTCGTTAAAAATATTATCGTATTTATATCTAAAAATATATTATCGTCGATAAAAATGGGTTATTGAGTGATCGATATATGTTGAGATTGTAGATATATTTGTTGTATATTTTACTAGTATCACATTCGCTAACTTTTTTTTTTGAGAATCTGATACACATGTCGATATTTTTGAAAAGCTGAACAATGTAGATTGGAGCGAGAGTGTAAATAATATTTTTTTTTGTGTATTTTTCGTTTTAATTTATATGAAAGTCTGTTGTTTGGATTCTTCAAAGCCGTACTAAAAATGTATGTTTTTTTGACACAAATTGATACTTTTGAAGAACCGAACAACATAGATTGACGATATAAATATTTGTATTAACGTATTTCTATTTTCAACGGCTCTTTGATCAGGATAATATTACCTAAGTTCCTTTAACTTTAAATGAATGACGGAGAATGCATGTGCTACAGTTGATTGGACACAAAATTAAAGAAAAAAAAAGAATTTATAATCTAAAATAACTTTTACGACTATAAATTATTTCCGTGAGGATAAAATAAGAATTTTATAAGTTAATTATTTTTTTAAAATTACAGTCTAATAGATTGAAAAGAAAAGTGTAGATTAAGAAAAAAAATTTATTATTATTATTATTTTAATACGTTTGATTTTTAATTAGTTTGTATATTTAATTGATTAATTTCATTAAACTTTTTTTTTAATTATTATTTGAAAGCTTTCAAATAATGAAGCACATCCAGGGTTCCATGATGAAGTGGACATAGAATTTCTTGGAACAACATTTGGAAAACCATATACATTACAAACAAATGTATATATTAGAGGAAGTGGTGATGGAAAAATTATAGGAAGAGAAATGAAATTTCATTTGTGGTTTGATCCCACAAAAAATTTTCATCACTATGCTATTTTGTGGAGTCCGAGAGAAATCATGTAAGTACTAACGTAACAATATCGTTATTATTATCAATCTGATAAATCTATCGAGATTTAATTTCATATAAAGTACTTTTATTTATTTTACTTCATTTAAGTAAGGAAATATTCTACCGACAAAAATCATTTGACACTAAAGTATATATGGTTGTCCTCCAATGAATTTAATCATGGTTTCCACCAAATAAATTTAAGGACATAATGTTATATTTTGATGCTTCATTTTATTAATTATTTATCATAAAGGGGACCATGTTATATTTTGATACTTCAAAAAATTTAAGGACATATGTTATATTTTGATACTTCAAAAAATTTCGAAAATCAATGGGTGCTTGAGCATCCGCGTCTGGCTTACTGGGTCCGCCCCTAATGTTAAGAGAATAATTCATGTTGTAGTCCTTTTCCCAATAGTAGTTTGAAATTCCACCATTTTTCACAAAAATAGATTTTCATATTCATTAGGTTCACGCGCCTTAAAAAAAAGGATAATTTTGTAGTAACGATAAAATTATTTTAATATGATCTATATGTTATAGATTCAAATGCTTTTGTTATAACGTAATGTCTATTTTGCATCTTCTTGAGATGTGATGTGTATTATATTATTCAGTATAAACGTAGAATGTTTTATGTATTTGAAAAACTAATTTGATCGGTTTCGTAATTTAGATTTTTGGTGGATGATGTACCAATAAGGAGGTATGCAAGGAGAAGTGATGCAACATTTCCATTGAGGCCAATGTGGTTATATGGATCCATATGGGATGCATCTTCATGGGCTACTGAGAATGGAAAATACAAAGCTGATTATAACTACCAACCATTCTATGGAAAATTCACGAATTTCAAGGCGAGTGGTTGCACCGCCTACTCGTCTCGATGGTGTCGACCTGTGTCTGCCTCGCCCTATAGATCCGGTGGCCTTAGCAGGCAACAACGTCAGGCCATGAATTGGGTTCGAAGTCACTATATGGTGTATGATTATTGCAGGGACTTTAAAAGAGATCACTCCCTTACACCAGAATGTTGGCGCAAGTGAAACAATTTTCTTATTTGTATCTTATATTCATCGGATGAACATAACTAAGCGATTTGTTGAAGCTGTGGGATGTATCGATGGTACACGTGTCAATCTTTTATAGGATGAATTTCCTATTTTGTGTGAACTACTTTTTTTTTCTTTTTTGTTATTGGGTATGGGGTTTGGGGTGTGGGGTGTGGGGTCTTGTGATAGGTTCCACTTGTCAATATCAATTGTAAGAGTAGTTGAGTGTCCAAGCTTATAGATTCCAAATAAAAAATCAATAATACAT

>SlXTH15

TTAAGAAGCTTCACTTCTCAAGTTCCTCTTGTGACGTTTTTAATTCCATTTATTTTTTTTAAAATTTTGGGGAGAAAATGGCTTCTCCTATAGCTTATTTTCTTGTACTTAGTGCAATAATTGTTGTACTTTTTTCATCAACACAAGCTGAAGTACAAGGTTCATTTGATGACAATTTTAGTAAAAGTTGTCCAGAAACTCATTTCAAGACTTCTGAAGATGGACAGATCTGGTATTTATCATTGGACAAAAAAGCAGGTAATTATAATTCAATAATTTAGTGTTTTTTTTAATACTACTTTGATAAATAAAAAAATTAAAAAATTTGAATCTTTTATGTTTTGACGAAAAATGTTCAACTGATCACCTTTCGATCTACGTAACATTGTTGAGTTTCTCTTTATTTGGGTGATTGAAATTCAAGAATTCAATGTTTTTTTCAATATATACAAATTTTCATTGTTTTTTTTACATTTGACTCGTTATGTTATGAGAAAGGTGTTCAATTAATCGCATCCTTATAGTGGTGATTAAAATTCAAGAATTTAATGTTCTATTTTTAATATATACCAATTGACATTTGCATCTTTTATTTTACTTATTTTTGCAAATATTGGTTGATTTTTTAATTTTTTTTTTATGTAGGATGTGGATTTATGACCAAACAGAAATATAGATTTGGGTGGTTTAGCATGAAGTTGAAATTGGTGGGAGGTGACTCTGCTGGTGTTGTGACAGCTTACTATGTAAGTTGCAATTTTTTTCATACATAATATTAAAAATGATAGATCGATGTATAAAATATTTCACGTTCAGATATAATTTTAAAAGTGTAATGCGTATAATTTATTTCAAAGCAAATATTAGTGATTGATTTTCAATGCTCAAATTTCATGACTTATTAGTCACTCAGAAATTACTTTTTTAATTGCTTCGACATCCCTCTAAAAAAAGAATTTTTACATTACTATAGTAATTACTAAATTACTTTGTTTTCTAGATTACTAATCTCACATTTTATAAACAACTTTGAACATGTATCAACTCTAGCAATCCAACACATAATGTAAATAATTTTTTGATTTTACTTGTGCGATTAGTCATATTGTAATATGTTTAATGGAATTTTTAACAGTCTTTCTATCTTTATAAGTAGGGGTAAAAATCTACGTACACATCACTTTTTCCATATTCTATTTGCAAGATTACATTAAGTATGTTATCATAGGCAGTCTATCTACCTGCACAAGGTAGGAAAAAGTATGCGTACACACTTTTTGATCTCCAGATTGTACTTATACTGAGTATATTATTATTGTTAAACTATATGGATTTTTGAATGAATTGTAGATGTGCACAGAAGATGGAGCAGGACCAACAAGAGATGAATTAGATTTTGAGTTCTTGGGGAATAGGACAGGTGAACCTTATCTTATTCAAACAAATGTGTACAAGAATGGAACTGGTAATCGTGAGATGAGACATGTTTTATGGTTTGACCCCACTGAGGATTTTCACACCTACTCAGTTCTTTGGAATACTCACCAAATTGTGTAAGTACCTATTAATTTTCATTATATTAAATTACGATGAACGAACGAGATTCTTTCACGTTTAATAAGAGTTTGCGTCAAAAATGTTGTCAAACATGTACAGGTTTTTCGTGGATAAGGTACCAATAAGAGTGTACAAAAACGCGAATTACACAAACAATTTCTTCCCAAATGAGAAGCCAATGTACTTATTTTCAAGTATATGGAATGCAGATGATTGGGCTACAAGAGGTGGTTTAGAGAAAACAAATTGGAAAAATCAACCATTTGTTTCATCATACAAAGATTTTAGTGTGGATGGTTGTCAATGGGAAGATCCATATCCATCTTGTGTTTCAACAACAACACAAAATTGGTGGGATCAATATGATTCATGGCATTTATCAAGTGATCAAAAATTGGATTATGCTTGGGTACAAAGAAATCTTGTCATTTATGATTATTGTCAAGATACTGAAAGATTTCCAAAAAAACCTGAGGAGTGTTGGTTAAATCCATGGGAATAATTAAATAATTCAACTTAGGTTAAAAAAAATATATATTATGAGAGGGATTAAGGAGTATTTTGATTATTTTATTTTTGATGTTGAGTTATTACTCGAGTATATATATATATATATATATAT

>SlXTH16

CATCTTAAACAAAACATATATTCATAAAATCTCTGAAAATTTAAAAAAAATAAAATGGTGAGTTTTAATTGGGTTTTTTCGAGCTTTGTGATGCTGTTTATGGTGGGTTTGGTTAGCTCTGCAAAATTTGAGGAGCTTTATCAACCCAGTTGGGCTTTTGACCATTTGACAACTGAAGGAGAGATTCTTAGAATGAAATTGGATCATCTTTCTGGTACTTTCTTTGAGTTTTTCTTAATGGTGTATTGTTGATTACTTAAAGATATTTTTGGTTGCTTTGCTTATTTGTGAAATTTTAGGTACTGGGTTTCAATCTAAGAGCAAATATATGTTTGGGAAAGTTACTGTTCAGATTAAGCTTGTTGAAGGTGACTCTGCTGGAACTGTCACTGCATTCTATGTAAGTCTAAAACTAATTTCTTTAATGTATATTTTTAAGAATATAGTACCAGTTTTTATCTTGAATTTTTGAAACGAGAACATTGATTTTGGGAATGATTTTTCTTTGGGTTGCTACAACAACACTTATTTAGGGGTAGAATGAAATTTCTAATGGCAGACAAATGTTAGTAGTGTGTCATTATGATTATATCAGATTTTTTTCAGTACATGCATTGGCTTATAATTTTAAGATCTGTGAACCATGTTAAATTGTGATAAATCAAGCATTTAATTCGTTTTTTTTCTTCTAATAGATTGAGTTTAATGATGAAAATCATCTGTTGTTGTTGAAGCTGATTTTATGTATAATCTAATGGACTCTGTTCTTACTTGTGTTTAAATTGCAGATGTCATCAGATGGACCAACCCACAATGAGTTTGATTTTGAATTTTTAGGCAATACAACTGGTGAACCATATACAGTACAAACAAATGTGTATGTCAATGGTGTTGGTAACAGAGAACAGAGATTGAAGCTTTGGTTCGACCCATCGAAGGATTTTCACTCTTATTCCATCATGTGGAATCAACGTCAAGTTGTGTACGTATCTAATTTTTGAAACTTTCATTTCAAGTTCTTGAATATTTTTTCCTTTTAGTAACTTATTTTCTTAAATTTGTTGTAAACAGATTCTTGGTAGATGAAACCCCTGTTCGAGTGCATTCGAATTTGGAGCATAGAGGAATCCCATACCCCAAGGATCAACCAATGGGTGTGTATAGTTCGATTTGGAATGCAGATGATTGGGCTACACAAGGCGGGCTCGTTAAGACTGATTGGTCACACGCGCCCTTCGTAGCATCCTATAAGGGATTTGAGATTAACGGTTGTGAGTGCCCGGCAACTGTTGCAGCTGCTGAGAATACTCGGCGTTGCAGCAGTAATGGGCAGAAGAAGTACTGGTGGGATGAACCTGTTATGTCCGAGTTGAATTTGCACCAGAGTCACCAGCTGATATGGGTCAGGGCGAACCATATGGTTTACGATTATTGCACGGATAGCGCTAGGTTCCCTGTTGCCCCTGTTGAGTGCCAGCACCACCAGCACAAGACGAATCATAACTAGGTGTGGAGGAAAATTGGAGTTCAGTCTTGCATTGTATAAAAAGATTTAGATAAAAAAGAAAAAAATGACATGAACTCCATGTTACATATTTTTTGTGCCACTAGTATGTAAAAATTTTCATTGTCTCTTTGTTTTAATGTTTATGTTAGTTTAATGAAGTATTTTCATTTGCATTTTTCTCTTGGTCAAATTTTACTTGAAGATATTTTTATTATTATTGAACAAGAATGAAATCAACTTAATTGTCAACGTTTGATCATATGAGAAATTGAATCAAATAATGATTATAGATAATAAATTTAAATCAAAATTGTGGGGGTGTACGGAAACATGACCAAAAAAATGTATTTATGTGGACAAAATTAAGATTATGAGGCAATTTTGCACATTTCCATGTGATAATTGTGCATGATTTTGTAGAATTTTTTTTCTTCGTTGGACC

>SlXTH17

GATCATCATTATTTCTCTTCAACAATAATATTTCAGCTAATTTGAAAAAAAATGGCTAATTCTCATTTACTTTTAATTTCCATTGTATTAATGGGCAATTTAGTGGCTGTATTAGCAGCTGGTAATTTTAATGACCTTACAGAAATCACTTGGGGTGATGGACGTGGTAAAATATTAGATGGAGGTAAAGGTCTCTCTTTGTCACTTGATAATTATTCCGGGTCGGGTTTTCAATCGAAAAATGAATATCTCTACGGAAGATTCGACATGCAACTCAAACTCGTCCCTAAAAACTCTGCTGGCACTGTCACCACGTTCTTTGTAAGTCGTTATTCAACTCTTTTTTTTCTCATGAATTATTTTTGAATTAATTGATTGTAAATAACTGCTACGTTTAAAAATATTTTTTTAGCTATCGTCACAAGGAGAAGGACACGATGAGATCGATTTCGAGTTCTTGGGAAATGTGACTGGTGAGCCTTATACGGTACACACCAATGTTTATTCTCAAGGAAAAGGAAACAAAGAACAACAATTTCACCTTTGGTTCGATCCAACTGCAGCATTTCACACTTACACCATTGTTTGGAACGCTAACCGCATAGTGTAAGGCGCTATACTTGTACGTAATTTTTTTTCAAAAAAAATGACATATTTCTTATATGAACTTTTCATTTACCCATTTATTCATGATTATAGTCTCACACAAATAATAAAGCATATTTAAAATGGCATGTTTCGGAGGTCGCTACTAAAAAAGTGAATTTACAATGGATGAAATCCATTAAAAATTGAATCGACCGGACGTCCATGGAAACTCATATTTCTTTATTAGTATGATTTAGAGTCACACAAATTTATTACATATCTGATACTATAAGTTTTAAAAATCTTATTTTTTATCCTCAAACTTTGTCACAATCAAACTGCTCCACGTCAAGGGTGGATCTAGACTTCGAGTATCGATCATCACCTATAAGTTCACGTGAACTTAATAGTTTTTCAAAAGAATATAATAAATAGTAATTAATTATAAATTCAATCGTCCTATAGTGATTTGAGATATCTATACAAATTTATTAATTTCAAATTTCTAATCTGTCAATGTGATCATATAAATTGAAACAAATGGAGTACATAAGAGGGGGCGCAGAGCCAATGGCATCATTAGGACAACACTATTTAACTTTTAAACCTTTTTTTTAATGTATATATAGTAAATACTGAACTCCTTCGATCTATGTGCTTACGTATTCATTTTTGATCTTCTTAATGATAATTCTGACTCCCGCGCTACTAGTAATTGGAGTAACTTCTTTATTACAGTGATATGTTTACCTTGGTAGATACTTACATGTTTGATTATGAAAATCAGGTTTTTGGTGGATCAGATTCCAATTAGAGTATACAACAACCATGAGAGCATTGGAATTGCATACCCCAAAAGTCAACCAATGAAAGTCTATTGTAGTTTATGGAATGCAGATGAATGGGCTACACAAGGTGGTAGAGTCAAAACTGATTGGTCACAAGCACCATTTACTGCCTATTATAGGAACATTAACATTGATGGCTGCGTCGTTAAATCAGGCGCTTCCTCATGTGCCTCACGGTCTACTGAATCCACGAACAGTGCTAAGTCGTGGGAGACACATGAGCTTGATGCTAAGGGTCGGAACAGGGTCCGATGGGTACAGAGCAAACATATGGTTTATAATTATTGCGCGGATTCTAAGAGGTTTCCTCAAGGATATTCACAAGAGTGTAAACGATCAAGGTTTTAAAAAATCGAATTTAATTTAATTACGTATGGTTCGAAACTCGATGAATATGTAAGCTGGTTATGCGATTGATTGTTTTTGTGTGTGAAATTCTTTATTGTTTTGTTTTGTTGTATTACCAATAAAATAAAAGGTTGCTTATTTATTTTTTTTGATTAAATGATATATGTACTATTCAAGTTTTATA

>SlXTH18

ATGGCAAAACTCATAGATTTTAATTCTTTGGTTTTGATGATTATTGCAATAATTGCATTATTTCATTCATATGTAGTCATTGGGATGACATCAAGTAGCATGTATGTTAATTGGGGTGCTCATCATTGTAAACTTCTAGGGGATGATCTTCAACTTGTTCTTGATAAATCTGCAGGTATAAGTTTTAATTTTTTTTCGATTTTTATACATTTAGTAACGGAGCCAGAAATTTATATATATGATGGGAATAATTCGAAAAATATAAGCATGTTATATTTGGGATTTGAATCTAGAACCAAAAATAATATTAGAACTCTTTACCCATGTACTAAAAATTTGTATTTATATTGACGCGATCCAATAGTTTATATACATAATATACAATAAAAATTAACCTATTTATACAGTGGAATTTTTTCGACATTGGCTCTAGTCTTAACAACCAACTAACCACACCCTTGCTTTGATGATGGTTGCTCTAGGTAGAGATAGATTCAGATAGATTCAGAATTTGGATATAATTGGTTCGAAATGAGTGTGATGTAGATATATATCTTGAAATTTACACATTGTGTACACAATTTGAAAATAGTAGTCAGTACTTAAAGAATTTACTCTAGAATCGTCTAAAACGTTATAATTTAAATTTCAGGCTCTGGTGCTCAATCAAAAAGATCATTTCTTTTTGGTAGCTTTGAAATGCTTATCAAGTTAGTACCCAATAATTCTGCTGGAACTGTCACAACATACTATGTAAGTATACTTATATAACATTTATTATGATGTGACAAAATTAAGCGACTTTAAAAATTTTATGCACTAATAATATATATAAGATGTAAACATTTTTTATATGATAATATATTTTTATTTATCATGACAGCTATCTTCTACTGGTACCAAGCATGATGAAATCGATTTCGAGTTTTTAGGAAATATATCAGGACAACCTTATATTATACACACAAATATTTACACCCAAGGTGTTGGAAATAGAGAGCAACAATTTTACCCTTGGTTTGATCCAACTGCTGATTTTCACAACTATACTATTCATTGGAACCCTAACGCCGTTGTGTAAGTCGATCTTTTCAGACAACTTCTTTTCATTTTATAAAAATCGTCTTATCTATCGACTAAACTATCTCCACCAATCACCTTTATTATCTATAAATTTCAAATATCTTTACGATTATCTCAAAATTCTAACTTTATATTAGCTTAACCTCATCAAAACACTATCCGCTATATTTTATTTGTCCAATTTATGTATTATATTTTTCCTTTTTAGTCTATCCTAAAAGGAATATCATATGCTCTTATTTAAAAACAATGTAACTTTAAACTTCTCATTTTACTCGTAACAAGATGACTTATATCCACACAAATATTTATCACTTGTTTTTTTACCACAAATTTCAAAAAAATTTCTTTCTTAAACTTTATGTCCAGCCAAATAGTATCAGTGCGACACATAAATTAAAACAGATGAACTAATAATAATATTAATAGTACAAATAGTCAATCTTTAATAGTAAATATTTCTTTCAAAATATATTCCACTCTCCAACCAAACACTCTAAGATGACTTCTGTCGCACCAAACACAGGAAACTAGTTGAAATTTGATGTGAAAATGTTTGTATTTTTTTTTCCAGATGGTACATTGATAGTATTCCAATTAGGGTTTTTAGAAACTACCAATCCAAAGGCATTCCATTCCCAAACAAACAAGGAATGAGAGTCTACACTAGTCTATGGAATGCAGATGATTGGGCAACAAGAGGTGGTCTTGTTAAAATTGATTGGACAAATGCACCATTTATTGCAACTTATAGAAAATTTAGACCAAGAGCTTGTTATTGGAATGGACCAATGAGTATTTCCCAATGTTCAATTCCTACAAAAACCAATTGGTGGAGTTCACCTACATACAATAAATTGAGTGCAAATAAACTTGGTCAAATGAACTCAATGAGGAGTAAGTATATGATCTATGATTATTGCAAAGATGTGAAAAGATTCAAAGGAGTTATACCTATTGAGTGCTCATTGCCACAATACTAG

>SlXTH19

ATGCAATTCAAAAACACATACACCATGAAGACTACTTTCTTACTTTTCTTGATTCTAAGTTTCTTCTTCTCTGCTTTGGCTGGAAATTTTAACCAAGATTTTGATATTACATGGGGTGATGACCGCGCCAAAATACTCGAAAACGGACAACTTATGACCCTTTCCCTCGATAAAGTCTCTGGCTCTGGTTTTCGATCCAAAAACCAGTATTTGTTTGGAAAGATTGATTTGAAAATCAAACTTGTGCCTGGTAACTCTGCTGGCACCGTTACAACATACTATGTAAGTCATCTTTTTCATCGTTACTACATGTTATATTAGGATTTTGTGAGTATACATATATATACCTCGTCACCCTAGGTTATTAATTCTATAGTCCAAAAAATATATTTTAATATGCCTTCTAAAATATGAAATGCATAATCTTGATCGGAAAATATGAAAAGACTCTGATGTATATAGCTAAGTTTTATAATATATTGAATGGTGATATTTGATTAGATGTTAGTATATTTTATAAGTTTGAGTTTTAATTGGTGTGAATTTACGAAATGTAGCTATCTTCAATAGGATCAAGTCATGATGAGATTGACTTTGAGTTTCTTGGGAATTTGAGTGGTGACCCATATATTCTTCATACAAATGTATTCACACAAGGAAAGGGAGATAGAGAGCAACAATTTTATCTTTGGTTTGATCCCACTAAGGACTTTCATACATATTCTATTCTTTGGAATCCTCAAAGCATCATGTAAGTAGTTCTCGTCTCTCTCACTCCTTTTATTTTGTGTCTCAATATATTACGGGGATAAAGGTCTAAAATATATTTAGATGTTGATAGAAAAAACTGTAATGATATTAAATTTTGATTTTTTTTTAATTTATGTATCATTTAATAGTGTATTTTGAAGGCATATATACGTCCACATGGACATCAGAAATATTGCATTATTATAAATAGAAATGTATCCATGTAAGCATATATATGTCTTTAAAATACATTATTAAATGGTGCAAGGGATAAACGGTCCTCCATAAAGTTCGGTTTCATCATAACAATTTCGGCCAAAGCTGAAGTATTTTTCGAACCTTTTCTCCCTATATTATTTGATTTGATTTAAAGTTGTTTTTAAATTCTTTAAAAAAATTAAAATAGATATTCATATGATTGAAAATAATTTCATGAAAGATAAAAAAGAATTTTAAAGTCAAGTTATTGTTAATTATAGAAATGTAACATTTTTTTAAACGAACTAAAAAAGAAAAATGTGAGCAAACCAATTATTATATTTCACCTGTATTTTACTAACTTCCATTTTGGACATTTACTTGGTATTGATATTTTCTTACCTACCGTTATTATATTGATAAACTTCATTATTAGTTAGTGTAGTACTACTAATAATATTGGAAGGTTTCACGTAATTTATTGTGGTGATTAGTTTTATATTTCCACCTAGGTACCTTTCTTGATTATTCAAAAACAACTCTACTGTAACTAATTTTGATTGAACTTTTTGGATTTAGTGCTTATACACAAGAAGTTAAAATTAAATCATATTTTGAAATTTAAGGGATTGCTGGATATATTACTACCAAAAAATATCAATGGAAAAATTATTTTGGCCAGAGTAGTTTTTACAATAAAATTAAATATATTTATGGGACTTACGCCTCATTTTCGATGCCTATGCCTCTATCCGTGCTGCGTAAACCACCTCTCCCGCCTCGTCGTTTGAAACACTAATTATAAATATTTTTCTATATAAAATACCTTATTCGCCCCGCCTTTTAAAACACTGATTATATAAATATCTTTTTTTTTCATAAAATACCTCCACACCCCATCTTTTGAAATACTGATTATAAGTACTTTTCTACGTAAAACACCTCCCCCTCCGTCCTGCCTTTTGGTCACACTGGTTATAATAAGTACTTTTCTACGTGAATACTTTCCCACCCTGCCTTTTGAAACACTAATTATAGTAAGTAACTTTTTGCATAAAACACCTCTCTCGCCGCCGTTTTTAAAACACTGATTATAAATATTTTTCTACATAAATACTTCTTGTCCCCGTCTTTTCAAACACGGATTATAAATACTTCTCTTGAACTTAATTAATTTGTCCTTATATGTTGATTACTAGTTAGTACTTATCTTTGACCCCCCATTTATTTAACAAAAAAAAAAATCAGATTTTCAGTAGATGGGACACCAATTAGGCAATTCAAGAATCTAGAATCAAGTGGTATCCCCTATCCAAAGTCACAACCAATGTGGATATACTCAAGTTTATGGAATGCAGATGATTGGGCTACAAGAGGTGGACTTGTCAAAATTGATTGGACTAAAGCCCCATTTATTGCTTCATATACAAATTTCAATGCACAAGCTTGTGTATGGTCTTCAACTTCAACTTCTTCTTCTTGCAATTCTACTACACAAGATTCTTGGCTAAGTGAAAACTTGGATATAACAGGCAAATCAAGGATTAAATGGGTGCAAAATAATTACATGATTTATAATTATTGTAATGATATTAAACGTTTTCCTCAAGGGTTTCCTCTTGAGTGTTCTCTCAATTAGTGCATGTAAATTAAGTTAATTATTGCATTGTAATTAAAATAATGTACTTAGGATTAGTAGAAATGTTTTGTTTGATTTGAGGAATAAGATGGAGAAATTCCATTTATACCTATTTGATAATTTTTAAT

>SlXTH20

CAATATATTCTCAAATTTTTTATAAATCCACTAAAAATAACAATGCCTTTTCTATTTTCCTTTAATATTAGACTTATTTTAGTTTTAGTATTTATAAGTTGTATGGTTGTTAAATATTGTGCTAGTAATGATCTTAATCAAGATTTTGATATTACATGGGGAAATGAAAGGGGGAAAATACTAAATAATGGTGAAATACTTACTCTTACACTTGATAATATTTCAGGTTCTGGATTTCAATCAAAGAAGGAATATTTATTTGGGAAAATTGATATGCAAATAAAATTAGTTCAAGGGAACTCTGCTGGCACTGTCACCGCATACTATGTAATATTTTATTTTTACAAAAAAAAATCAAAAAAATTGGCTATTTTTGTTAATTGTTATGTTTTTTTTAATGTGGGTATTTTTTTTTTTTTTAATTTAGTTGTCATCACAAGGATCAAGTCATGATGAGATAGATTTTGAGTTTCTTGGTAATTTAAGTGGAGAGCCATATACACTACATACAAATGTGTATACACAAGGCAAAGGTGATAGAGAGCAACAATTTCACTTGTGGTTTGATCCTGCTAATGATTTTCATACTTATTCTATCCTTTGGAATCCACAAACTATTGTGTAAGTACTCTAAAACAATATTTTATTTTATTTTATAAAAAAATTTGATAATATTTCATTCATCTGTCCATTTTTACTCGTCTGTTTTTATTTTATAACAAGTTCATCTGTCCATTTTTACTTGTCAGTTAGATAATATTTCATTCATCTGTCCATTTTTACTAGTCTGTTATACGAAAACAGTTTGTTATTGTTTGGAATATCCTTATTAATTAGATACTATATACTTTGGTCCTTGTTATATCCTTGTCAATTAGATACTATATACTATGTATTTAATATCTAATATATTTTCTAAAATATTAAATTTAATGTATTTAAGAAAAAGGTGAGATAATGACATTGTTTAAACTACTTATCATTTTTCTTTTAATCTATCAAGTCAATAGTGATAATTATGATTTGAACAACTAAAAATTAATAGTGGATAAATATGATTGGACAGAGAACGTATCAAGTTTTTCTTTCCGAAATGTGTTTTGCTTACTATGAAAATAGTTCCTAGGTCATCTGATAAGAGTTCTCAAGTTCTGATAGTCCATGTTTCACTTGTTTCACTCTTAACGAGAGTTCTCAGGTTGATAATTGAGTTTCGTGAATTTTTAATGAAAAAGTATATTTAAAAATAGAAGTTATGATATATCAGATAATATATAGTATAAATAACTTTAGTTATCTTCTAGATGATCTGACAGTTGAAAAGTTTATTGTAATTTTTCTTCAGATTTTCAGTGGACAATGTACCAATAAGGGAGTTTAAAAATATGGAAAACATTGGAGTTGCATTTCCAAAATCTCAATCAATGAAACTTTATTCAAGTTTATGGAATGCTGATGAATGGGCCACAAGAGGTGGGCTTATCAAGACTGATTGGGCCCAAGCCCCATTTACAGCCTCTTATAGAAATTTCAATGCCAATATTTGCAATAATAATAATAATAATAATGATTCTTGCAAATATTTGGTAGAAAATTTGGATCCTGTGAATGAAGAAAAATTGAGAAGGGTGCAACAAAAATACATGATATATAATTATTGTACTGATAATAAGAGATTTCCTCAAGGTTTTCCTCTAGAGTGTAGTGTTAGTTAATTAATTAATTAAATTTAATTAATGTAGGTAGAAAAGTTGGTATAATAATATTAATAGCCTCCTCTAGTTTTGTCTCTTTCAAGTTTATTATATATTGTGGCCTAAGTGATAAGGGAGGACTACTATTGTAATATTTATATGTTTATGTTTTTAATAATGGAAGTTGAAAGTGTTTATGTACTT

>SlXTH21

TGGTGTCTTTTTTTTTTCTCAATCAAAATAAGTCCATTTTTCTCTTCTTCTTCTTCCTCTCTTCTGTGGAGACTGATGAGACTTCATTTTTTCCCCCACTTCTCATGCATTACAACTCTATAGAAAAATACAACAAAAAAGAATTAATCTTTGGGTGTTCATAACTGATAAAAGGTGTAAAGGAAAAAGAAAATACCCAAAAAATAGAAAATAAAAATGGTGAACTATTATATGTTCTTTTTCATATTTTTGTCTTGTATTCTTGTTTTGGTTTCTGGGTTTTCAAGAAATCTGCCAATTTTAGCTTTTGATGAAGGTTACTCTCATTTATTTGGTGATAATAATCTTATGATCCTTAAAGATGGAAAATCAGTTCATATTTCTCTTGACAAAAGAACAGGTTTGTTAAAAAATATGTTTTATTAAGGAGAATTAATTTATTTATTGTTTTTTTGGTGTTTTTTTAATGGATGAGGTGTTTTGCAGGGGCTGGATTTGTGTCTCAAGACCTTTATTTTCATGGATTTTTTAGTGCTTCTATTAAGTTACCTGCAGATTATACAGCTGGTGTTGTTGTTGCATTTTATGTAAGTTATTATTAACTAAGTAATTGATTTTTTTTGTATAAATTAGAAGTTTTTTACTTGTCATGAGTGAAAGTTTTGATTTTTTCTGTTTTTTTTTCCACTCCCATATGGATTCTTGATGATTTTTTCATATGTTAATTTATTTTGTAAAATTTTCTTGTGTAAAGATTGGATTTTTAACCTTGCAATGATGTTTAAATAGTTAATTTTCAGTCTGTGTTATGTTAATGAAATGAACCAATAGTTTTATTTTTCATTATGTTAATTAGTACAAGTTTTTGCTTCCCATGAGTGAAAGTTTTGATTTTTTTGTTTCAGTTGTCCATATGGATTCTTGATGAATTTTTTGTAAATATATCACCTTTTTCCTCAGTTTATAAAATCATTTTTACCTTTAAAGAATGTGTTTTTAATCATCTAATGGAAATTGTGTTTATAATAATAAATTAGTAGCTTTTTCTTCCAAGAGGTGAAAGTTCTGTTCTTTTCAGACTCTTCTTGATGATTTTTGAACTGTTAATTAATTTTGTAGATTTCTCCCTTTGAATGGTTTTTTAACCAATCTAATGGGAATTGGTAATTTTCAAGTTGTGTTAATAATGAAAACTAAAATCTTGATTTTTTTGTTGAACTTTTACTAAAGTTTGTTGATGACAGATGTCTAATGGGGATATGTTTGAGAAGAACCATGATGAAATTGATTTTGAGTTTTTGGGAAATATTAGAGGCAAAGACTGGAGAATTCAGACTAATATTTATGGGAATGGTAGCACAAATGTTGGCAGAGAAGAAAGATATGGACTTTGGTTTGATCCTTCTGAAGATTTTCATCAATACAGTATCCTTTGGACTGAGAATTTGATCATGTAAGTTAATATCTCTGCCCTTTTAATGTTTCATTCCTAATTTTGATCATGTATGGTCTACGGGGCGGAGCTAGGTTGAAAAATTACATCATATATCATAGATATTGAACCCCCTTGGCTTGAATTCCCTTGGTGAATATCCTGGTTCCGTCACTGGTGTGGTTTGTAACCTATGTTGCTCGGACTCGAAATATAGTGGATTTTTGGAGGATTTGACAGGGGCTATGAGTACGAGTACGAGAACATTTTTAGAGAGTTAGGTTGTAACAACTTTTTTGTTTGTCATACCATGAGTTTTCACATCCAGCTATTAAAATGGTTTCTTTTATGAGTAAAGGTTCATTTAGATGTCTGTGTTTTGTGATGCACATGTTTCTGTTTTTAACTAATTTTGTATAATAAGTACAACAATTATTACCAAACTTTTTGAATTATTTGTTTTTAATAGTTAAGCTGAAGAATCAAATGAATAGAACAATGTGTGACTAGAAATTTTGTTCTGATATGTTGTCACAAGATTTTTGATTGATTTTTTGTGTCAATGAATGTACATGAGTGACAAGGCCTTTCTTAACATACCCTTTTGTTGGCATGGGACTATGGGAATCTTTTGTTTGTTTCTTATTATTATTTTGCTTAAGATGGTACATTCCCTATTATGATGGTTGTGCTCGTCGAGGACTTGGCCGTCAATAAGGAATGGGGTAAGGTCTACGTATACATTAGTCTCTCAAACCACATTTGTGGGATTGCATTGGGTTTGTTTTATGCTAGCAATGCAAATATTGCGCGGACTCTTCAAATATGCAGCCTATTGTTTGTCTGATCCTCCAAAATTAGTGTATATCCGGACGGATCAGACAGACGCTACAACATTTTTGGAGAGTCCGAGCAACATAGCTATCATGTACTTTGTGAACTCTTCAAATATGCCTCCCTGATTCTGTTTTGTTTGTATTTGTGTTGTTTTGCATCTTGTTGCAGCTTTTATGTAGATAATGTCCCCATAAGAGAGATCAAGAGGACAAAAGCCATGGGTGGGGACTTCCCATCTAAGCCAATGTCCTTGATAGCTACAATATGGGATGGTTCTAATTGGGCTACAAATGGTGGAAAATACAAAGTCAATTACAAATACGCCCCGTATATCGCTGAGTTCTCCGATTTCATCCTCCACGGATGCGCGGTTGATCCAATCGAACTGTCATCCAAATGTGACAACACTACGCCAAAAACTCCAACGATCCCTACCGATATCACCCTTGACCAAAGACGAAAGATGGAGAACTTCAGAAAGAAGCAAATGCAATATTCATACTGCTATGACAAGACCAGGTACAAGGTCCCTCCTCCCGAGTGCGTGATCGACCCTAAGGAAGCCGAACGACTCCGAGCCTTTGACCCCGTTACATTTGGAGGATCCCACCACCATCATGGGAGACGACATCACCGGAGCAGACCAAAGTTGAAGGGTGATGATGATGTATCCTTTATGTAAAAGAAAAAAGACCCAATCCCATAAGTTTTTCTCTATATAGTCACATGATGATATGATCATTTGAGGTTTTGTTATTTTCACATGTGAATAGAGGATATATTGTTGGGGTATGTATATATGTCATACCTATATAGGGGTCCCAAGCCATGTTCTAGTTGATGTATTTTGTTGTTTGGGCCTTTAATTTATAAATTCAACTTGTTGGTGATTTTGTGTACTTTTTTTGTGACACATTAATAAATGCATGAATAGTACAAAGATTATTTATTATAC

>SlXTH22

ATGGGCAGCTCTCTAGTTCTTTCATTGGCTAATTTGTTGATTATTTCAACAATTGTGTCATTTGGTTCTTTAGTTATGGTTAATGGTATTTTCTCAGATAATATGTACATTAATTGGGGTTCTCATCATTCTTGGATGCAAGGAGATGATCTTCAACTTGTCCTTGATCAATCTTCTGGTAAATTAATTTCGTCTCAATCATTTTCGTTGAAAAATTACAGTTGTAATATCTTAGTGTAATCTGTATAGGTCATGATCAGTTTGAACTGTGAAAATAGTTATTGATGATTGTATTAACTGTCTACATCTTTTCTTTTTCCAATCCTATTGATGTGAAATATTTTGTGCATTCGAACTTTATGTTATATATACATTATGTCACTAAATAATTGTTACTTTTGGTTTATTGCATTTTCAGGTTCAGGTGTACAATCAAAAGGAACATTTCTATTTGGAAGTATAGAAATGCAAATTAAATTGGTACCTGGAAATTCTGCTGGAACAGTCACTGCATACTATGTAAGTGTCACCTTAAATAATCTTCAATAGATAATACAACAAATTTTATATATAATATATATGTATATATATATATTTATATATATATATATATATATATATAGGATCTGGCTCCTCTCCATTTTCCCCAAGTTCTAGCTTGGATTGAAGACACATGTCATAGTTTAGATTTAATTGTTGAGATCCAATTGATTAAAATAAAAGTCCTAACAATAATTATTTACTTCCATATATTTATGTATATTTGCTTCTCAAATATTTTTACAATCCAACAAATTCTAGATTTACATTATATTTTTTCTAAATATATTTAAAAGAATTTCTTTTCTTAATTACTTAAGTAATTTTTTGGTGATTTTTTTATATAATATAATAACATAAATCCTAAAATAAAACGGTTCATATTATATGAATTGATAAAAAACTTATCATATAAATAATATTAAACATTTAAGTGATCACCACTAAAAAAATCCTTAGTTAAATCTTTTTTTTTTTGAGCTTTTAAATAGTTTCAATCGACCGAATTTTTCTTAATTATATTTATAAGTTGTGGCCATTAATAACCCTTTATTTATATGATTATTAATATGCATTCAAAATATATAACATAATCAATAAAACTTGAATGGTTCAAGATTTTTTTTTTCCATATGGCGTTTGGTACCCACATTTTGAAGTCTAATATATCTAAATTCGCGCTGAAAAGTGTTAAAAATGCTTCCGAACAGCAACTCCATACCCAAAGGGACTCGAACCCGACCATGAGTTTGTGTAGATTATTGATTCTGTGTTTGGTCTTCTTATATAATCTTGGACAGTTCTCTCCTTATGAATTAACTTATGTGATTGAGTTATAAGCTTGACGTCTATTTCTTTAACATGGTATTAGATATAAAAAATATTTGTGGGACGTTATTTTTTGTGTACTGATTAAATAAAAAGATAAGTAAGAAAATTACTTTTTTTTTTACAAACATATATATATTTGATGAAAAATATTTTCGTTTATATCGAACATATAATAAATGTAATTCATATTGTAAATAGATGTATTATAATTATTATTTCAAAGGGAGTATCTAATTTTTTGGTTTTTAATTTCACAGTTATCCTCAACTGGTGACAAGCATGATGAAATTGACTTTGAGTTTCTAGGAAATGTATCAGGACAACCATATATTATACACACAAATATATTTACTCAAGGTGCTGGAGGCAGGGAACAACAATTTTATCCATGGTTTGATCCAACTGCTGATTATCATAATTATACCATTCATTGGAACCCTAATGCAGTCGTGTAAGTTAAACGCTCTTTATCAAAAAATTATATATACATATTATATGTATATTTTAATGGAGCGTTGATCGAGTCAATTCTTGACTAATGTTTATTTATTCATGTTGTAGATGGTACGTTGACGATATACCAATTAGAGTCTATAAAAACTATCAGAGTCAAGATATTCCCTATCCGAACGCGCAAGCAATGGGGGTTTACTCTAGCCTTTGGAATGCTGATAGTTGGGCAACTAGAGGTGGTCTTGTCAAATGTGACTGGACCAATGCACCATTTATAGCCAAGTATCGAAATTTCGCCCCACGGGCCTGTGCCTGGAACGGACCTATTAGCATTAGTCAATGTGCAACTCAAACTCCAAGTAACTGGTATACTGCTCCTGAGTATAATCAATTGAGTTACGCGAAACAAGGTCAAATGGAATGGGTTAGGAGCAATTACATGATTTATGATTATTGTAAAGATACGAAGCGATTTAACGGACAATTTCCTGGAGAGTGTTTTAAACCTCAATTTTAA

>SlXTH23

GGATCCAGCCTATCTTCATTCAAAAGGGAAAAAAAAAACCACCATGGAGAGCAATGCTTCTTCAATGGCTCGTGTTCTTTTGATTTTATCAGTAATTTTTACCCTTTTTTCATCATCAAATGGTGTAGTTGGAGGTGCATTTGAAGAAAATTTCAGTAAAAGTTGTCCTGGTACACATTTCAAGACTTCTAAAGATGGACAGATCTGGTATCTTACCTTAGACCAAGTATCAGGTGATTAAAATCCTATAAATTTGAAACGCATTTTATTTTGTTCTTTTGAATTTTGAATATATTTCAATTTTTTTTGTGTGTGGTATGTTAGATTGTGGGTTCATAACAAAACAGAGCTATAGATTTGGTTGGTATAGCACAAAGTTGAAATTAGTAGGAGGTGACTCTGCTGGTGTTGTGACAGCATTTTATGTAAGTTCACTTCAAACATTTTGAACATTTTTGTGGGAATTTTTTTTTAATGTTTTTTTTTGGGGGGTTACAGATGTGCTCAGAAGTAGAGGCAGGGCCATTGAGAGATGAGATAGATTTTGAGTTTTTGGGAAACAGAACAGGGCAGCCTTATCTTATTCAGACAAATGTGTATAACAATGGGAGTGGTGGACGTGAAATGAGGCATCAACTTTGGTTTGATCCTACTCTCGACTTTCATACTTATTCCATTCTTTGGAACTCTCATCAAATTGTGTAAGTCTTCTTTTTCACTCTCTAAGTTAATTTTTGTTTTCCTTTTTCTAGTCGATATTCGAAATTCAATCAGCGTTGGGTAGATCCATTAAGAAGGTAAAATGTTTGCTACATTCAATGTACTTCATCCATATGCGCATAAGGATTAAAGTAAATTTATCAGAATTCACGACCAGTAGATCCATCGTAAAATGTAACCTTTTTTGGATTGAAAATTACATTTATGTAGTAGATACGATTACTCTTCAGTTTTTTCTTATGATTACATTTTTTACCGCCATTTCTAGGATCTAAATCTAAGAAATTTGGTTAATGATATAAAAATTCGAACCATCTTACTACTCCCATCTATGGTTACTTTTTGTTTCTCTAATTATATACATAAAGGTACCACAATACTATAGTAACAATCTTTCAAAGAAAAAAAAAAGGTCATTTAAAATTGCGACTAACATCAGGATACTCGTGAAATGGATTTGAACATACCGAAATGGATATGTTGAAAACAAATAATTTCAAATTAACGATGACTCGATCAAGTATCACATACATTCTTGTAAATGCAGATTTTTTGTGGATAAAGTACCAATAAGGGTATACAAGAACGCGAATCACACAAACAATTTCTTTCCAGCCCAGAGGCCGATGTACGTGTTTTCAAGCATATGGAATGCAGATAATTGGGCTACTAGAGGAGGCTTGGACAAGATAAACTGGGAAAATGCACCATTTGTAGCATCTTATAAGGATTTTACCATAGACGCTTGTCCATGGAAAAACCCTTACCCTGCTTGTGCTTCATCCACCACACAGCACTGGTGGGATCAGAATAATACTTGGCACCTATCAAGTAAAGAGAAGATTGATTATGCTTGGGTTCAGAGGAACTTTGTGGTTTATAATTATTGCCAGGATACTGTGAGGAACAAGTACAAGCCTCAAGAGTGTTGGTTAAATCCATTGGACTAATATTAAACATTAGAAAAAATTCATTGGTGAGAGAAAAGTCATAGTAGTATTTTGATCTTGATTTTGAGTGTATATATTGGACGATTGAGCCATTGTAGATCCATCGATTGGTATATTGTAAACCATCGGCATTACTACTATTGGAAAGTGAAAAGAGTGGTTCACTATATTTACAAATGTTATAACGATATTCTTAATGTAATCAGTATGTTTTATTCAAGGAAGTTAAG

>SlXTH24

CAAATTCATCCAACAAACTTTGAAATCTTAACAGTTAAACATGGCTTCTTCTTCTAAGTTAGTACTTGTAATGTGTTTTATGATTAGTGCTTTTGGCATTGCAATTGGGGCCAAGTTTGATCAAGAATTCGACATTACATGGGGTGATGGCAGAGCAAAAATACTTAACAATGGCGACCTCCTTACTCTCTCACTTGACAAAATCTCTGGCTCTGGTTTTCAACCCAAGAATGAATATCTGTTTGGTAAAATTGACATGCAGCTCAAACTTGTCCCAGGAAATTCTGCTGGCACTGTCACTGCTTACTATGTAAGTGATTCCCTTAATTTCTGAATCATTTTTGAGCTATATATACATGAATATCCCCTGAAATTCAACTCTTCTATTGCAGTTGTCATCACAAGGACCAACACATGATGAAATAGATTTTGAATTCTTGGGAAATTTAAGTGGTGATCCTTATACTCTCCATACTAATGTATTTAGTCAAGGCAAAGGAAACAGAGAACAACAATTTCATCTTTGGTTTGACCCTACTGCTGATTTCCACACGTATTCCATCACTTGGAATCCACAACGCATCATGTAAGTAGTTAAACATTCAAACTTTCTTCAAATCTAACACATTCTTTTGCGATAAATCTTGATTTGGGGTCTCTTATTTTTATGGTTGAAACAGATTTTATGTGGACGGAACGCCAATTAGAGAATACAAGAATAGTGAATCGATTGGAGTTTCATATCCAAAGAACCAACCCATGAGGATATATTCGAGTCTTTGGAATGCAGATGATTGGGCTACAAGAGGAGGACTTGTTAAGACTGATTGGAGCCAAGCACCCTTTAGTGCTTCTTACAGAAACTTCAGTGCTAATGCTTGTATTCCCACTTCTTCATCTTCTTGCAGTTCCATTTCTGCAACTTCAACAAGCAATTCATGGTTGAATGAAGAGTTAGATAACACAAGCCAAGAGAGGCTCAAATGGGTGCAGAAGAATTACATGGTTTATGATTACTGCACTGATTCAAAGCGATTTCCACAGGGATTTCCAGCAGATTGTGTTCAGAATATCTGAGCATTAATAATGAAAAAATAGTGTATTACTTTAAAAACTATTGTATTGATTCTTTTATTGTTTTGTACCCATCAGAAGAAGATGCAATAATTATTGAGGATTAGAAACATCTTAGTTTTGTACTAAGTTATATAAACAATGAAATAGATACTTTTTTTTCTTCTAA

>SlXTH25

CTATTTTTTTCACTCCATAAATAGCCCAATTTTGCCCCACACATTCTCATTATTACAAATAATAAAATAAAAAATGGAATTTTTCCTTCATGATAGAAAATTTATATTATCAGCATTCTTGATTTTATGCATGATTATTGTTGTTTCATGTCGAGGTCCAGTGTACAAACCTCCAGAAATCGAAAAATTAACTGATCATTTTAGTCGATTATCGGTTAATCAGAGTTATAATGTGTTTTATGGAGGTTCTAATATTCATATTACAAATAATGGGTCAAGTGCTGAAATTATTTTAGATAAATCTTCAGGTACACTACAACAATTTCATACATGTTTTTTTTTTGGCTTAGTTTATAACTTAGAGTATTAATAACATTTTTTATTTCGTCACTCCACAGGTTCTGGACTAATCTCTAAAGAGAAATATTACTATGGTTTCTTTAATGCTGCACTAAAATTGCCTGCTCATTTTACATCAGGAGTTGTTGTTGCCTTTTATGTAAGTTTCTATTTTATCCTTAATAATAATCGTGGTGTCTGGATTAATTCATATGTATACGAAGAATATTTTGTGTAACTCTGTCTATCAAGGCGTGGACATATTGGGAAACCTTTTTTTTTTTTTTGCCTTTGATGAACTGTTTTGAACATAAGATATCCTGGTTTTCAACTTACTTCATGCATGCCTGGTAATTAGTACTTCGTATGTCCGTATTCAAAGTACGACTTTTGAAATTTGTGATGTAAGAACAAAGTTTTTGTGGCTATAATTATCTCGTTAAGTATAATTTTTTTTTTCAATTTTCAGATGTCAAACTCAGATGTGTTTCCACACAACCATGATGAAATAGACTTTGAATTGCTTGGACATGAGAAGAGAAGGGATTGGGTTTTACAGACAAATCTTTATGGAAATGGAAGTGTTCACACTGGAAGAGAAGAAAAATTCTACCTATGGTTTGATCCAACTTTGGATTTTCATGATTATACCATCCTTTGGAATAATCATCACATAGTGTAAGTCCGTTTGTTTCAATTTATTTGTCTGATTTTCAAGACAAAGTAATGATATCATAATTCGCTTAAGTTTGAACTGATTATTGGCCTGCTTATTTATTAACCGAGTCCATTTCGAAGGATTGACATCTCTCCAACTAAGCTACACCCCATTATGTTTGGAAACTATGTATACTTTTATCTACTTGGTTTCAAAAGTTGACAATTTTATTGTAAATTTCAACAGATTTCTCGTGGACAATGTACCAGTAAGAGAAGTGGTTCACAACACAGCAATCTCTTCAGTTTACCCATCAAAGCCAATGTCAACTATATTGACAATATGGGATGGATCAGAATGGGCAACACATGGAGGAAAATACCCTGTAAACTATAATTATGCACCTTTTATAACAACAATCAAAGGTATTGAATTAGAAGGTTGTGTAAAACAACAACAAAATACATGTTCTAAGAGAAGTAGTACTTCAAGTTTGGACCCTGTTGATGGAGAAGGATTTATGAAGTTATCATCACAACAGATGAAAGGATTGGATTGGGCTAGGAGAAAACATATGTTTTACTCATACTGTCAAGATACTAAGAGATACAAAGTTCTACCACCAGAATGCACTTCTGAATAGACGGAATATGACAGTCTGGTGGACACAGTATCTCGCATTAGCAAGAACGACTCTTCCCTTATGGAGGAACATCTGTTTGACTAAATTCATTCTATGATGTAAAAACTTATTGGTCTGAGTTCCTAACATGTTCAAGAATCTTACTTGGAGGTATTCAAATGAAATAATTTCTTTTTCTCTTTATATTTTCGAGTTTGCTAATCAAATTGATAAAGTCTAAAGATGCTAAATTGTGCAATAATTGAACTTGATTGCATTCTCCAAATATTATGTACTATATAATGGGCATATATAAATAAACATTTAAG

>SlXTH26

ATGGATCATCGAGTTCTTTCATTTGTATCAAAATCAATAACACCTTTCTCTCTCCTATTATTACTGTACATTTTTCCGGCGGCTGAGACGGCGGCGAACATGACGTATAAGGCGTTTAATCTGCCGACGATTACTTTCAAAGAAGGATATTCCCCTCTTTTTAGTGATTTCAATATTGAACGATCTCCTGATGATCGAAGCTTTCGTCTCCTCCTTAATAAATTCTCAGGTAATTTCAATTAATTCAACTTTTGAAATTTATCGTAAAATTTCACTCAATATGTTATTGTTACGTGAATTGTCAATTGCAGGATCGGGTGTTATTTCAACAGAATATTACAATTATGGATTTTTCAGCGCTAGTATAAAGTTACCGGCCATATATACGGCCGGCATCGTCGTCGCTTTCTATGTAAGTATCCAACAAATAATTAATATATGCATTTTTTTTTATATCTGTGTTGTTTTTTTATCAATGAATATACATAGAATATTTTTTTAAAAGCCTGATTTTGACCACCTATTTTTGTCATTATATTATTATATGCATATACAATTATAAGAGTTATATTGCATCATTGTTCATTGGAATTTAAAATAAGTGATGTACATGGTATACAATTCATCGAAGCAATCACTAATACTTATAACTAGCTAGTTCTATATATGTACGTAATGCAGAATGTTTCATGCATCACAAAAGTATACTGTGATGATCCAACTAGGTACGGAGGTAGTTTTTAGTATATGAGTAAGATAAAAACTTAAAACTTCGTATCTTAGTTAAGAGGGGAAAAGGATAAATATACTCTTGAAATATCGTAAATGGTATGCAGATATCCTCCGTTGTACTTTTGGGACACTGATGTCCCATCTGTCCAAAAACTAGAGTATATATGCACTTCAATCTAACGATAGAATAAATAAGGATACGTGGCGCAATTTTATTCGTCGATAAATGTCGGATCGATCATAAGTTTGTGACACGTGTATGTCCGTTAGTATAAAGGGTGTATGTGTTCTAGTTTTCGAACGGCAGGGGTATCAATATCCCATAAGTATGACGGAGGGTATTTACATACCATTTCAGATAGTTCGGGGGTATATTTGTTTTTTTACCTTGTTAAGAAATTCATTAAATATGTATAAAAAATAAATTCAGAACTCAGTTACTAACATTCACTACAATAAAAATGATCATTAGCGGCATTTAATTCTTAATTGCCGCTAAATATGTATTTTTAGCGGCAATTATCACTCTTTACGTATGTCCCTAAAGCCTTTAGCGACATTCTAATGCCAGTAAAGATGTTAGCACTATTTATTAATGTCAATATTTAATGCCGCTAAAAATTATCTTTGCTATAGTGATTTGAGATCATTGACCATGTCTGCTTGTTGGAAGATGTTGTGTTTTTTCATTTAATGAGTTCATATATGGGGATATATGTAGACATCAAATGCAGATACATTTGAGAAGAATCATGATGAGTTAGATATTGAGTTTTTGGGGAATGTGAACGGTCAACCATGGAGGTTTCAGACTAACATGTATGGAAATGGCAGTGTTAGCCGTGGTAGAGAAGAGAGGTATAGAATGTGGTTTGATCCAAGCAAGGACTTTCATCAGTACAGCATTCTTTGGACACCAAAAAACATCATGTAAGTTGCCAAATTATATTCCTTTCTCTTGCTCTTCGATTATTATCGGTTTCAATACTATTATTTTCTAGTAATACTATTTCTGGCCGACTAATTAGGATTCCCATAGCAAATTTATTTGGTCACCTATTGCTTTTGTTGAATAAGATAATGATTATCAATATGCGTTGTAGTGCAGTGATAAGATTATTTCATTCTTAATTAGAGGTTTCGAATTCAAGACTCGAGCATGAAAAAAATTCTAATAGAAACTCTAATGTAGACACCAATATCGAATGAAAAAAAAAGATAAATTAAATATACAATCAAAACATCCTTGTAATGTTCATTCATATAAAAAAAAAAGTTGTTTTAACTAACTAATGAAAAATTTACAAAGAGTCTCAATAACACCCTTTGGACGAGGGTTATGACGTTCATTCATATAAAAAATTGTTTTACCTATCTAACTAATGAAAAAAAACAAAGAGTCTTAATATAACACCCTTTGGACGAGAATACGTTGTTCTATCACTGATTAAATTGGGTTATTCTTTTCATCTGTTATATATATATATAAGAGATTTAAAAATAAGTGATCATAGAAGATTATTTTCTCAAGAGGATGAAAGTAAAAATAAATTGAGGAGAAATGAAAATTGAAAATAAATAGAGAAAATGAAGTACCATTGGAAAAGGGAAGAGAAAAATGTTGATTTGCTCATTAATTAATTAATCTGAATAGCTAATCTTTATTAAAGGGATATAAAGTTCTAATTGCGTGATTTATGTAATGCAACTATTACTTTTTCCTTTTTCTAGAAAAAAATCTTAAAGCTGAAATACTGGCGTGATTGGCTGTTCTCCTAATGACATTTGCACTAATTGAATTTTGTACAACACTTAAAAAATTATCTACACTTGAAAAGAAAGGTCAATAATCATGACTTTGACCTTAATTATTACTTAATAAGTATCTGAAGCATTAAAGTGACCTTCAGGTCACACGAATTCGAATCATGAATGCACTTTTTTTGAATTTTATGTGAACACGAAATACTTTCTGCATCGAATCATCATGTTTTGTTCTTAATCATCAACAAGTTTTTAGGTTACATTACCACTATATTTTTGCCATCAAGCTTATTTATATGTGTTACCATCAAGATTATTTATCAAATTTTGTTTGTACTCTATAAATTAATGAGTACATTAAATAAAGGACTACATAAGTAGTAAATATCAATGTCTTCTAGGTTTTGTACGAGGAAATGATCTCTTGTGTCTACTAACTAGTATATCATCGAATAAAGTGATCACACTAAAGTTCACAAAATAAATTTGTGAACATTTTCCCTAAAACTTGAAGATAGTATTTCTATTTTAGGTTCATAAGATATGTTGAAATAGTCGCGTTATAATTATTTAATGATTGAAATTATACTGATATATATCGAAAGTGTGATGATTTTACTCTCAGGAGTGTAATTTTACCTGTTGCAACATAATATTGTCTGCTATATTTTCAAGTTGTTACACTTATTATAAGCAGTTGCCAATCTTTATGTTGCTCTAACTTAAAATGCTTTCGCGCACCTGTGTTAGATCCTCAATAATGCACTAACTTTAGAGAATATCACGTTCCGTCAGCAGTTTTGAAGAGCTCGTGCAACATAGGACTATAGTTATAGAAGTTTTGATTTTGTTTCCTCTCTTTTACAGATTTTACATTGATGAAACACCACTTAGAGAAATAAATCGCCATCCAGCAATGGGAGGTGACTTTCCAGCAAAACCAATGGCTTTATATGCCACAATTTGGGATGCATCTTCTTGGGCTACAAATGGTGGCAAAGCTAAAGTGGACTATAAATATGAACCTTTTGCAACAGAGCTTAAAGACTTAGTTCTTGAAGGATGCATAGTAGATCCATCTGAGCAAATTCCATCAACAAATTGCACTGACAGGAATGCTAAATTACTTGCTCAAGATTACTCTAACATCACGCCCGAAAGGCGAAACAACATGAAATTTTTTAGGGAAAGATACATGTACTATTCTTATTGTTATGATAACCTTAGGTACCCTGTGCCACCACCAGAATGTGTGATTGTTCAGTCCGAAAGAGATTTGTTTAGGGACAGTGGAAGGCTTAGGCAGAAGATGAAGTTTGGTGGGAGCCACAGCCATACCCAAAGCCACCGGAAACACCGCCCTGGACGGAGCTCTAGGCGGCGGAATAAGGTGGCTGGCGGTGCATCAAAATCTGGCCGACGAGGTTCTGCTGCTGCTGCAATGTGATAAGTTGTATAACTAAGGTATACAAGGTGAATATTATGGTTTGTAAAAAGTATATTACACTCCTATAGTTGGTATACATACTCATTAGTGGTATAAATATTAATGTGGTGGCTGGGTGAAGAGTAAAAACCACATCCATTAATTTTTATGAGCAATGGTTTTCTTGGTTTCTTCACTATGTATATTGGCACTTCATATTCATCCAAGCAATGTTATGATGAATGGTTGCATGGTTTTCGCAGCTATTTGTCA

>SlXTH27

ATGGCTAATCTCCTCTTAATTGGAGTTGTAATTGCTATGCTATGCTCTGAAATTAAATGTTCATTTGAAGACAACTTTAGTAAAAGTGATTGTCCTGACTCTCACTTCAAGACTTCTGAAGATGGACAGATCTGGTACCTATCATTAGATAACAAAGCAGGTAAGTACGTTACGTACAGTCAAATTTCTTTATAACAACATACGTTATTTGTCTAGATAGATATGTTATGAAAAAACATATAGTGCTTATTATTTTTTCATTTATTTTTGACATTTGAAACCATAAAGTACTTTTTCTTCTTATAGGTTGTGGATTTATGACCAGACAGAGATATAGATTTGGTTGGTTTAGCATGAAATTGAAATTGGTAGGAGGTGACTCAGCTGGTGTTGTTACAGCTTATTATGTAAGTTGCGACATTTTGAATATATATGTCATGCATAACTTAATTTAGTTATATATATACATTGATCATATAAAAAAATTTCTTGTGATTAATCTATTTTTGAAGTTCCTTACTATCTCAAGTTTCAACTACAAGAATTTAGTCTACCTAAATTTTTAGGTGTTAGGACTCATAAAATTTATATCTTAAATTCGTCAGATGTGCACAGAAGATGGGGCAGGGCCAACTAGAGATGAGCTAGACTTTGAGTTCTTGGGGAATAGGACAGGGGAACCATATCTTATTCAGACAAATGTGTATAAAAATGGTACTGGTGGGCGTGAGATGAGGCATGTTTTATGGTTTGACCCTACTCAAGACTTCCATACATATTCCATTCTTTGGAATTCTCATCAAATTGTGTAAGCATCTCTTTCGATTTTAAATTTGAATTAGTTTGATCAGTAGCAGAGTCTGTAATTTTACATATATAATTCAAAGTATTAAGAATGAACACATAAAAAGTCGAGAGGATTCAACATCTAATATGTTTATCTAAAAAGTAATTTTAACATTACATTATAAAATATATATTTTGGGGGAATGAGGTTTAGATGAACCACTTGCACTCATGTGACTTCAACTTGTAACAAATTTCAATGCGAGTATCAGACATGTAGATACGGAGATTCCACTTTTAAAACATTTCATGGTTGTACTTAATTTATTAGGGTAGAGATCTGTTTTTATAAGTCAAATGAATAAATAATACTATTACACCTAGTACTACCTTCTTAGAAATTGGGGTGCACTGGTAAAAATATTATAACTTTTAAATTAGTCAACTCTTAACTTTCAAGCAAAAGGAAATAGATTTAAATGTATAAATGTCAGCCATTTCAATTTGTCACTCTTATTTTTCTTTTTACTCCATTTTAAAAAAGATTTTTTTTTCTTCACATTCCGACTTTTCACCTGATATATTTACGATCATAAATCAGATTAAAGGTTATTTTGATGCATTCTGTTCATCTTTGTATGTATTTATTTGTTGTATGTGCAGATTTTTCGTAGATAAGGTTCCAATAAGAGTATACAGAAACGCGAATTACACGAACAATTTCTTCCCTAACGAGAAGCCAATGTACTTATTTTCGAGCATATGGAACGCGGATGATTGGGCTACTCGGGGCGGGTTAGAGAAAACAGACTGGAAAAATGCACCATTTGTATCAACATATATGGATTTCAATGTTGATGCTTGTCAATGGGAAGATCCTTTCCCTTCTTGTGTTTCAACAACTACTCAAAATTGGTGGGATCAATATAATTCTTGGCACCTTTCAAGTGATCAAAAATTGGACTATGCTTGGGTGCAAAGAAATTTAGTCACTTATGATTATTGCCAAGATATTGAGAGATATAAAGTAAAGCCTGAGGAATGTTGGGTAAGTCCATGGGATTAATTACTATTTCAATTTTGATTTAAGAGTGTGTAGATGTTGCATTGTTTTGTCATGTGTTAAAAATCATGAATTCAATTTTATAATAGGGTTACCCTAATAGCTAAGGATTTCAAATATATGCGAATGACTTTTCATTTTGAGTTTA

>SlXTH28

CTTTTCCTAACAGACCGATAGACCTGAACTTTTTTCTTCTGGACTCTGAAATTAATCTATCTTCAAGTTCTAGACAGCAATGTCATCCTTTATGATTGTCTTTTTGATCCTATCTATGCTACTAAACCCAGGGGTTGGTGTCAACTTCACTGATGTTTTCGAGTCCAGCTGGGCACCGGACCATATTGCTGTTGTAGGAGACGAAGTTACTCTCTCCCTTGACAGCGCTTCTGGTAAGACTTCATTAATTCACTTCATATCTTATATTTATGCCTATTCAAGGCAATATAATTACATAGCCATTTGATTAATTATGTTCATATTCACAAAGGCTGCGGATTTGAGTCGAGGTTCAAATATTTGTTCGGGAAAGCCAGTGCACAGATCAAACTAGTTGAAGGAGATTCAGCCGGAACAGTTATTGCATTTTATGTACGTGACAAAAACCACGTCAAATAAATATCAAGATCTAACATATTGTTTCTTCAAACTATAATAACTTATGGCCTAATGCTACTAATTGCTGGAAATATGTGGTTTGTAGATGTCATCAGAAGGAGCTAATCACGACGAACTGGACTTTGAATTTCTTGGGAATGTTTCAGGGGAACCATACCTAGTACAAACAAATATCTACGTGAATGGCAGCGGAGATCGAGAGCAGAGGCACGGTCTGTGGTTCGATCCAACAACGGACTTCCACACTTACTCTTTCTTTTGGAATCATCATTCTATCATGTAAGTAATTCTACTTGAACCATATCTCTAAATATGTTCTTTCCGTGAAACATGTAGAGGTGCAACTCCGTAGGACCGAGGGTGTTACTATTTTAGAATTTTATATTATCAGTGTATATGAGTCAAATCTACGGTAACAATGACTAGTTTTAGTCGACCCCTGAATCTTTCACAGCTTTTCAGTTGATGATATTCCAATTAGAGTGTTCAAAAACAAGGAGAAAAAAGGTGTTCCATATCCGAAAAATCAAGGCATGGGAATCTATGGATCGTTGTGGAATGCAGATGACTGGGCTACACAAGGAGGGAGAGTGAAGACAAACTGGAGCCACTCTCCATTTGTTACGACATTTCGATCGTTCGAGATCGATGCTTGTGATTTGTGTGGTGAGGACACAATTGCTGCAGGTGCAAAATGTGGCAAGTTAGCTAAATTTTTGTGGGATAAACCATCCAAGAATGGGCTAGAAAAGAGCAAAAAACGCCAATTCAAAATGGTTCAAAACAAGTACTTGGTGTATGATTATTGTAAGGATACTGCAAGATTCAATCAAATGCCTAAAGAGTGCTTGTACTAGAGACACCTCGCAATATTGTAGTTTCTAAAAATGTGTATCCAAATTGCTGATCAAAATTTTCTTGTTTGGCTTGTTATTTGACACTCTTTTAACATTTAAGACAGTTCTATGGTCAAAAAATAAATTCAACTGTGCTATGACTTTGAATATTTAATTCAAAT

>SlXTH29

ATGGCAAAAATCATACATTTTAATTCCTTGGTTTTGATGATTATTGCAACAATCACATTTCAATCATATTTAGCCAATGGATGGACATCAAGTAGCATGTATGTCAATTGGGGTGCTCATCATTGTAAACTTTTAGGGGATGATCTTCAACTTGTTCTTGATAAATCTGCAGGCAAGTTACAATTCTTTTGGTGATATATATAATAACTTTTTCGTATATTACACGTAGAATGTCATATGAACGGGTTGAATATATATAATAATTTTTTGTATATGTCTACCTACATAGCTTAAATTAAAGTTAACAGATTCAGATGAACCTAGATACATGCTCTAGATTCAGCTCTAAACTTATTAGTCTAAAGTGAAAAGCGATTGAGCAAATGAAAAATAATATTTATATTTGATTGAAAAAAAATTATAAAATTTAAATTTTTCGAGTTTACTAATTATTGCGGTTTGATTCAACTTCAGGCTCTGGTGCTCAATCGAAAAATTCATTTCTCTTTGGTAGCTTTGAAATGCTTCTCAAGTTGGTACCTAACAACTCTGCTGGAACTGTCACAACATATTATGTAAGTATACTTGTAATCAATTTTAAATTTTGTACATTGACAATATATAACGAGATGTGAACGTTATTTATATATTGACGTTAACATATTTTATTTCATGACAGTTATCTTCTACCGGTACCAAGCATGATGAAATCGATTTCGAGTTTCTAGGAAATATATCAGGACATCCTTATATTATACACACAAATATTTACACCCAAGGTGTTGGAAATAGAGAGCAACAATTCTATCCATGGTTTGATCCAACTGCTGCTTTTCACAATTACACCATTCATTGGAACCCTAACGCCGTTGTGTAAGTCGATCTTTTCAGATCAAATTATAAAATTATTTGAAACTTATGATTTGAAATATATGTTGTAAATGACTTTTATCATATCAGACATGTCGTCAGAATTTGATGTGACATTTTTTTTTATTTTACAGATGGTACATTGATAGTATTCCAATTAGGGTTTTTAGAAACTACCAATCCAAAGGCATTTCATTCCCAAACCAACAAGGGATGGGAGTCTACACTAGTCTATGGAATGCTGATGATTGGGCAACAAGAGGTGGTCTTGTTAAAATTGATTGGACAAATGCACCATTTATTGCAACTTATAGAAATTTTAGACCAAGAGCTTGCTATTGGAATGGACCAATGAGTATTTCCCAATGTGCAATTCCAACAAATTCCAATTGGTGGGCTTCACCTTCATACTATAAATTGAGTGCAAATAAAGTTGGTGAAATGATCTCAATTAGAAGCAAGAATATGATTTATGATTATTGCAAAGATGTGAAAAGATTCAAGGGAGTTATGCCTATTGAGTGCTCATTGCCCCAATACTAA

>SlXTH30

ATGGGTTTTCATCTAATAAGTCTAAGTGCTCTTTTATTATTAACTAGAGTTTTTGAAGGTCTAGCTTTACCATTTGATAAAAAATACAACATTTCTTGGGGGAACAACAATGTTAAGTTATTGAAAAATGGAGAAGAAATTCAGCTATCTCTTGATAAATTTTCTGGTGATCTTACCTTTGTAGTTCTCTTTCTTCTTTGTTTTATACCTTTTACACTTCAATTTTGAAAATGCACGTCTAAACACCTCAACTCGTCTTTATTATATTTCGTGCGTGAACATCCAGTACTGATATAACACATAATTCTTGGAGGTGTCTAGATGATCATTTGATTTGATGGAGTGTTCAACTGACACATTGAAAAAAATTTAAAAAATCTAGACATGCATCTTTAAAATTTGAAGTGTTTAGTTGTTAATTGAGACCAGATTAAAACGTATACCTACATTATGTCATTTAAATAGGGTGATGTGTCAAGTTTTGTCCCTTTACTATGTTCAATTGTTCATTCTCTTTTGTTCACATATTATCTTGTATTTGCCTTTGCTATTAACGTAGCTCTAGAGTGAAATAGTTGGATTCCACTTGTCTCTACTATTTAAAATGACTATCAACTTGGAGCTCTAGTGGGGGTAAAAGGAAAAAAATATAGAGTTTTTTTCATTACGATAGGATAAATTAAAGTTAAAACAAAAAGTTTAACCGATAATAAAATTGTTCGATTTCAAATAGTAAGAGGATAATATTACTTTTCTTCTTTGCTATTGTTTTTTAATTTTAAGTTTTTGTTTTCTTAGAATTCTAAATACCAAATGATTTTTTAAAAATAAATAAATAAATTATGCAGGATGTGGGATTGAGTCCAAACAAAGTTATGGCTCTGGATCATTCAAAATGAGAATAAAGCTACCAAGCAAAGACTCAGCTGGAGTAGTGACAACATTTTATGTAAGAACACCTATATACATTCAATTGTTCACTTCTTGAAGAGTTAAAATAATTTAGTTTTTTACCATTTTACAAATATTCATCAAGTTCGGTAAAAAAGATATTGCGTTCAAGTAGGATTTGAGAAAGGATCGTATCCTAATGGGTGTGATAAAAATATTGCATTATTTTTATAATTCAATATCGTGATTTGAGGTCACACGAAGACAACTATACCATTGCTTCAGGACCCCTCTTCCTTCTAGTACTAGATAAACTCTTTTTAAAAAATAAATAAATGTTAGATTGAAGGTCGCAAACATAAATAGAGTAATATAGACAATGAGAATTCATACAACCGATTTTTCTTTGATGTAGTAGTTATTGAATACTTGATTACATTTAAAACTCTAAAAAACCATTATATTGTGGAAATTTTTGTTTAATTTTAAGTTGCTCTAAAAAAATAATTTTTTGATCTTTTGGCAGCTACATTCACATACAAGCCACCATGATGAATTGGATTTCGAGTTTTTAGGTAATAGAAAAGGGAAACCATACATATTGCAAACAAATGTATTTGCAAATGGTATTGGTGATAGAGAAGAAAGAATTCAACTTTGGTTTGATCCAACAACAAACTTTCATGAGTACTCAATCCTATGGAATTCACATCACATTGTGTAAGTTCATTTTTCTTTTCGGTATTCATTCAGTATCTGATATTCACTTTATAATTTTATTAATTCGAATTTGTACCAAAAAGTCCAATTTTAACACTCTTACTCGAACTTCAAGCCTCTAATTTACGCTTTGATAGTAAATACGTAGTAACGTTCCTTTCCAAAATTAAGAAAAATAGATTAATCTAAAATGTAGCAAGTTAAAAATTAGATCAACCAATAAGTTCTTTCAATTTAATCATTCAGTATCCAAAGCTTACTAGTCTGGTTAATTTGGATCCCCATTAGGTAAGCCTATAAAGGAAAGCAATATAATTTTCACATTTCTTAGAATTTTGTGCTTATTTATCACTCAATTTGGTTGTTTAGATCTTTTCGTGACCTTTGATATTGTATTTATTTAGGAGTAGAGTAAGACGTAAATTTTTTCTATAGCAAAAAGTCTTTCATTTTTAAGTCTTATGAAACCAAGGCTTTTTAAGTAGAAGTCATTGAGAGCCTGTTTGGCTCAGCTTAAAAGCTGGTTTTTGACTTATTTAACTGTTTGGCAATACTCAAAATAGCTTATTTTAAGTTAAAAAAAAATTATTTTAAGCCAAAAGTTAAAAGCTGGGGTAGGGGTGCTTTTTTTTTTTAGCTTATAATCTTTTTAAGTTGACCACATTTTTATGTTTTTTCCCTTAATATTTTTATACAATCTTCAAATTACCCCTATAACCCTAACATCTCTTTCTTCTATTTTTCGCTTTTCACGTTTGGCATAACAACTTCAGCACTTTTATCCAAACGTATAACTGCTTATTTTAAAAATAAGTTTCAGCACTTTCAAAAATACTTTTTTAAAGCTGATTTTACTAAGCCCATCCAAACGGGCCTTGAATCTCAAACATCCTTATGTACCTCTCGATGACATTTACAATTTAATATTTATTGTCTAATTGATTTGTTTATTTGAAGTTTTTTTGTAGATGAAATACCAATTAGGGTTTACAAGAACAAATCATATAGAGGAATTGGATACCCTACACAACCAATGCAATCAGAAGCCACAATATGGAATGGAGAAAGTTGGGCAACAGAAAATGGAAGTCAAAAAATTAATTGGTCAAATTCTCCATTCATAGCTCAATTTCAAGGCTTTAACATTGAAGGTTGCCCTTCTAATTATCATAGTTTAAATTGCAATTCAACAAAGTGGTGGTGGAATTCTAAGAAATTATGGAAATTAACTCTTGATCAAGAAAAATCATATAAAGATATTAGAAGCAAAAATATGATTTATGATTATTGCAAAGATACCAATAGATTTCAAAACATTCCTTTAGAATGTTCAAGTGATTATTAA

>SlXTH31

ATGGCTTCTTTTGAGTTCATGAGTATAATTATTTGTATTTTGATGTATTTTGCTCTGTCACCAATTTATGCTATGGTTGATTTTAATCAATATTATAATCCCTTGTGGGGTCAAAATCATATAACTTATCTTAATCAAAGTACTGAAGTGCAGTTACTTTTGGATCAATCAGGTATATCACTGACAAATTTTTATATTTATTTAATTTAGAATTTACAATTAGTGACAAGTTTTTATATATGTTTAATTTAGAATTAGTGACACATTTCTATATCGATTCAATTTAGAATTTAAATTCAATGACAATATTTTTAATCTGTTTAATTTAAAAGTTTGAGTCAAATAAATTCGATATAAATTCAACTAGATCTATAAATTGCCATAGAATTACCATATATTTAACGGTCCGTATATCTAATTACTTCTCCATCATAAAAATGTACTTGTTTTTCTTTTACATTACAAGAGATTATGTCATTTCAATGGCATTTTAAAATTATTCTAGTTAAGTCACATTTTTTTGGTAGAATAGTAAGTTATATAATATGAAAAAACTCATTTATTTTACACTAGAATTAAATGTAGCAATTTCAATATTGTTTTTTTTTGTTGTTGATGTCCCTTCTTTCCATATTAGTTAGTTATTTTGCTTTTGTTAAAGTAAGAAATTACAAATAAGGTGGACAATTAATATGTGACAATTATTTTTAGAAAAATAATTAATTAACATGGGACAGAAGAGAAGAAGTATTTATTTAGACATTTATTTACGACTTTTATGAAATGATTAATGATGCAGGAGGAGCTGGATTCAAATCGAAAACACAATATAACTCTGGATTATTTACATTAAGAATAAAGATGTCAGATAAAAAGACCGATGGAATGATCACAGCTTTCTACGTATGTTATTAATTTTTTTTCTTTGATAATGTATAAATGAGTACATATATAATTATAAAAATATTTTTTTAACTTAATTTTTATCACAGTTAATTTCAGATGATCAAGATGCACGGGTTAATCATGATGAAATAGACTTTGAATTTATAGGGACTCAAGGAAAATTACAGACAAATATATTTGCTAATGATATGGGTGGTAGAGAACAAGTTTTTCAACTTCCATTTGATCCTTCTCAGGATTTTCATACTTATCAAATTCTTTATACTCCACAAAGAATAGTGTAAGTAATTATAATTTACTTCTTATGTTTGACATATTTTAGTATCTTAATTAGCTTTATTAATTTACGGTTATTTGTTGACTTTTATAAAAAAAAATGTGTGTTATTTTTTCATATACATACATAGATATAAAAGCCAGCTATTTATCCTAAAATAAGCCTCCACAGATAAGTGGTTCTATAATTATTAAATTCTGAGATAGTACTGATCCATCAATATTTAGATTTTCATTCCTCCTTTCATCTAATATAGTATCTTTCAAATCGATATTCAGTAGATTTGCACACCTACTCAATAAAGTGTTAGACCATTAGACGAACCTCATCCCATACCAATAAAGTAGGAGGGAGAATCTTCTTTTTGTTCCTTTATTTTTATTTTTTTTGTTTGATTGATTCATGTATGTTTTTATTTCATAAAAAAGTTGACAATAAATCGATTCATCTAACCAACAACTTGCGATAGAGATACCTAGTGGAAAGTTGGTCCAATTCTCCTTGAAAAGCAATAAATATTGCATTCTAGTGTTTCTTGAATTCTGTTATTAGAATTTCTGACTGTACAACTGTTTGATTGAAAACCGTACATGTACTAATTTGTGTTTGATTTTTATAGGTTTTTTGTGGACAACATACCAATAAGGACATTTGAGAACAACACAAATAGAGGTATCAACTATCCAACAAAATCACTATGGTCAGAAGCAAGCCTATGGATTTCAGATGCTGTGGGTTGGGCTGGATCTGTTGAATGGGGCTATGCACCATTTATAGTTAGTTTTCAAGACTTCAACATTTCTGGTTGCCCTGCTGGTAGTGATTGCTTGCCATCCACAGATTTTAGCCCATGGACTAGGCACAAATTAGCCTCAAGAAGCTTGAATCTTATGAGGAATTTCAGAAAAAAATATATGACTTATGATTATTGTAGCTCTGAGGAAAATAAAAATAGGTACCCAGAATGTGCTTAA

>SlXTH32

CATATATACAAACTTTTGACTTATTTTTGAAAAAACAAATCAAGAAATTTCACTAAAATTGAACCAATTATTTTCAATGGCTTCCTTAGTTCTTTGTTTGGTCATTTTGGCATTTTGCTCTTTACATTATAGTTTGGCTTCTAATAATTTCAATCAAGATTTTGATGTTACATGGGGAGATGGTAGGGCAAAAGTTTTAAACAATGGCAAACTTCTTACTCTTTCCCTTGACAAAGTTTCTGGCTCCGGTGTTAAATCCAAGAAAGAATATTTGTTTGGAAGGATTGATATGCAACTTAAGCTCGTACGTGGAAATTCAGCTGGTACAGTTACTACATATTACGTAAGTATTATCGTACTAGTGATGCATATCATTTGAACTATACTAAGAATTGAAGTCATGTGACAATTTACTATTTTGAATATCATATGAATTTACTAAGAAATTGTTTTATATCAATTTTGTATGTACTATATAGTTATCATCACAAGGGTCAACACATGATGAGATAGATTTTGAATTCTTGGGAAACCTTAGTGGAGATCCTTATATTGTTCATACAAATGTGTATACTCAAGGCAAAGGTGATAAGGAACAACAATTCTACTTATGGTTTGATCCCACTGCTGATTTTCATACCTACTCCATTCTTTGGAATCCACAAACAATTATGTAAGTAAATAAAAATCAATCTCCGCTTACTTTTACTTATCCACTTTAAAAATTTTATATATTTGTTAAAAAAATTAGTATGTGATTGATATAAATATTTTATTATAATATTCATATTTATAGTCTTAAATCTTGAGAAATAATCCCTCCCCTAGAATTCACATCTTTACTTCGTTGGTGACTCGAACTTATAACATTTCTCTTTGATCTTTTGTTGATTCAAGCTTACAACCTTATGGTTGAAAGTGATCATGATGCTTACCATCCGAGCAATCCCATCGTGTCAGTAATGAATTAAAATCTTGATAATAAAACATGAAAAAGTACTTATTTTTTCTTGATTCGTTGAAAGTGACAAAGTAAAAATAAACAGAGAAATAATATATATCCTTAGTAATAATTAATAGAATCAAAAGCTCAAAATATACAAAGTCAATATAAAGATCTCTCTCTTGTTTTTTCCACCATAAAGTTCTACAAATTTAATTTTATCTTAATATTGACTTAATTATTCTTTTTTTTTTGTAGATTTTATGTGGATGGCACACCAATAAGAGTGTTCAAAAACATGGAGTCAAGTGGAGTACCTTACCCAAATAAACAACCTATGAGAGTCTATGCAAGTTTATGGAATGCAGATGATTGGGCCACAAGGGGTGGCCTTGTTAAAACAAATTGGTCCAATGCTCCATTCATAGCTTATTTTAGAAATTTCAAAGACAATAATGCTTGTATTTGGGAATTTGGAAAATCATCATGCACAAATTCAACAAAGTCATGGTTCTATCATGAACTTGATTCTACAAGCCAAGCTAGGTTACAATGGGTGCAAAAGAACTATATGGTTTATAATTATTGTAATGATATTAATAGGTTCCCTCGAGGCCTTCCTCTAGAGTGCGCTTTCAACTCTACGACTAATTAAATCTAGGTTTTCAGACTCTTTTAAAATGTTATCATATGCATGTCAATCTGATGTACACATGCAATCGACATTTTCTGAGAGGAGGGCACTTTCAATTCTACGACTAATTAAATCTAGGTTTTCAGACTCTTTTAAAATATTATCGTATGCGTGTCAATCTGATGTACATATGCAATCGATATTTTCTGAGAGGAGGGCACTTTCAACTCTACGACTAATTAAATCTAGGTTTTC

>SlXTH33

ATGGGCTTCAAATGGACGATGATGTTGGTGTTGTGTGTGTTAATAGGAGGATCAATGGGAGCTAAGCCCAATAAGCCAATTGATGTCCCATTTGGAAGAAATTATGAACCTAGTTGGGCTTTTGATCACATCAAATATTTGAATGGTGGCTCTGAGATCCAGCTCTCCCTCGATAACCGCACCGGTAATATTTTATTTTTCATCGGATGTGTTTGGTACGATTAAAGTTATTAATTGTATTATTTTTGTTGTTAATGTTTATTTTTGCATTACTTTCAGCATGACTAGCTTCTCTACTTCTTTATTTGTTTTACATACGTGATTTTATTTGTTAAGTTTTCCTTTACTTCGAAGGTTTATCGAAAATAATTTTTCCGTCTTCACAAGATAAGGGGTAAGGTTGTTTATACACTACCCTTAACATTCCATCGTATGTATTTGGTATGCCGAAAGTCATTAATCGTATTATATATTGTTAATCTTGATCTTTGCATTGTTTTCAACATGACTTGTTTATTTGTGTTACATACGTGTTTTTTTTGTTATGTTTTACTTGAATCGAGGATTAATCGAAAAACAGTTTCTCCGTTTTCATAAAGTAAAGGCTAAGGTTTTATATACACCACCTTTTTTAGACCTCGATATTATAGTGAATATGTTATTGTTAGTTTTAGTTTTTGTAGAACAATCTTGACTTAACTTTTGATACTAATATTATTATTTACTTTAACATATACTATACTTTTTTTTCCAAAAAGTGTTAGTATTTCTTATGTTAATGCTTCTGATTTTGAATTTGTTTTGGGACATATAGGCACTGGTTTTCAGTCAAAAGGATCTTACCTATTTGGGCACTTTTCTATGCACATAAAGATGGTTGCTGGTGATTCTGCAGGCACTGTCACTGCTTTCTATGTAAGTCAAATTACATATACTAATAGTCAATTAATTGTGTTTAGATCTTAAACTTGTTAAAATTGATCTATATAAATTATACAACAAACATATATTTTGTTATAATGTAAAGGTGTGATAGTCTGTGATAAAGCTAGGGTTTTTATTTAGGGGTATAAAAAACTTATAATAATGTCGCTAGCAAGGATATATATTAATCAAAAAGCAAGGAGCGTCAACATTGATACATATATATATATATATGTGTGTGACTATAAAACTAAAATTTTGACCTATTTATACAATATAATTTCCTAGTGAAGGGATGTCGCTTGACAACCCTCGAGCAAATGTAGCCCCGTCCCTGATGATAGGCCGCTTCGAACAAAATAATAATTCGAAACTATTTGCACAAAACTTTGTGTCACGGGGCTTCTTTGTAGGTTTTACAGCTGAAAAACTATATTCAGAGGTGGATTTAAGCAGATTTAATTTTATCTCATACGCTATATATAACGATAATAATAGCATATCCAATATAATTCTAAAATGTGAGGTCTGAGATATATATATGTTAAAAACTTATTGTTACATAGATACAAAAATTTCCGAACCTGTGAAGTTGTTCTGACGATGTGAATTACTTTGTGTGTATATAGTTGTCTTCTCAAAATTCAGAACATGATGAAATAGACTTCGAGTTCTTGGGGAATAAAACAGGAGAACCATACATTTTACAAACAAATGTATACACAGGAGGGAAAGGTGACAAAGAGCAAAGGATTTACTTATGGTTTGATCCAACAAAAGATTATCACACTTACTCTGTCTTGTGGAATCTTCATCAAATTGTGTAAGTAACTTTTTATTTTTTTATGATCATTTGATAATTGAAAATCGTTTATTCTTTAATTAATTTAGATTCGTGTTATACATTAAAAAAAGAATTTTATTAAGGGGTTTTACATTTCAAATGTCCAACCTGTTTAAATTTAAATGAATTGAATTATATGATTATTATTAATTTAACATAATGAATTGAACTGACATGATTCATCAAACTATTGAGTCAAATGAGTCAAAATATAACCAAACCCAAGTTTACAAACTCAAAATTTGAAGATTGGGAATTATATAAATCTGAAAAAATTAAGGTACTAATTTAAAGAAAATGTCAGTTTCATTCTTTTTTTCTCAAAAAGGCGACAAAAAAAAGGAGGTTTAGTTAGGTCAGGATCGAAGGGTTAAGTTACCATCGTTACTTGAATTATTTTAATCTAAATAAACTTTAGGCACGTTAAATCATGAAAATTTATTAGTTTGACTTATTTTAACATAAAAATAGGGTTTACTTCATAATTTTTGATTTGTTTTCAAATAAATAGTTATTATCTTAAAGTTTCATATACCACAAGTGAAATTTTAGGTCATTATCTTTGCTCTTTATTTCCTTTATAACTAAAGAAAAATACATAATTATATATAAGTTTTTTAAAATATATTTTAATATATTCAAATTTAATTCAATGCTTCCTTTTTTGACAACAGATTTTTTGTGGATGAGTATCCCATAAGAGTGTTCAAGAACAACAAGAACTTAGGTGTCAAATTCCCATTTGACCAATCAATGAAGATATACTCAAGTCTATGGGAAGCAGATGATTGGGCAACAAGAGGTGGACTTGAGAAAATTGATTGGTCAAATGCACCCTTTGTTGCCTCATACAAAGGCTTTCACATTGATGGTTGTGAATCTTCTGTCAATGCCAAATTTTGTGCCAATCAAGGCAAGAGTTGGTGGGATCAAAAGGAATTTCAAGATTTGGACAAAACTCAATGGAGGCTTTTGAGAAGAGTTAGGGACAAATATACAATTTATAACTATTGTACTGATAAAAAGAGGTTCTCTACAACGCCAATAGAGTGTAAGAGGAATAGAGATGTTCCAAGGAATTCAAGAAAGGAAAATTAA

>SlXTH34

AATTATCCCTAGCTATTTCCTCATCCTCAAATCTTTGATATAATCACCAAGAAAAAAAATGAATTATTTCTCTAGATTTATTTTCTTGGCCACTTATTTTATTTACTTATCTCATATTGCATTAGCTTCTATAGTTTCTACAGGAGATTACAATAAAGATTTCTACGTAACATATTCACCTAACCATATAAACACTTCTGCTGATGGCCGTACAAGAAGCTTGATATTTGACAAGGAATCTGGTAATTATTAATTAATTAATTCAAATGCAATATTTATTTATTTTGGCTACGAACACAAAGTTAATTAATTTGATGAATACTTTTTTGTTTGATTAACTTGTTAATCAGGTACAGAGATTGCTTCAAAGGATATGTACTTATTTGGTCAATTTGACATGAAAATTAAGTTGATACCAGGAAATTCAGCAGGCACTGTTGTAGCATTTTATGTAAGCAAAAAAAATGATTAATTATCAAATATTTCTTCAGATTTTTTTTTTTATGATATTGGAATTACGTATATATGCAGTTAGCTTCGGGTCAACCGAATCGCGATGAGATAGATTTTGAATTTCTGGGGAATGTAGATGGAAAACGTTATACTCTTCAAACAAATGTTTATGTTGATGGATTCGACGATAGAGAACAGAGAATCAATTTGTGGTTTGATCCAACACAAGACTACCATACTTATTCTATTCTATGGAACCTTCACCAAATTGTGTAAGCCGTTTGATCTTTTAAATTTTTATCACTGAAATTGGTGATCTAACGTTAAATTTTAGTAAGAGTTCTTAAATATATCCGATAAAAGTATAATATTTTTTGTTTGCATCTCATATACTTCACATGTGCATTTTGTGTGTCACACGTGTAACTCGTGTGGATTGACTATTTTTCGATTTTTGGGCAGGTTCATGGTAGATTGGGTACCTATTAGAACATACAGAAACCATGCAGATAAAGGAGCTAAGTATCCACATTGGCAGCCAATGGAACTCAAAATGAGCCTATGGAATGGAGAAGATTGGGCAACAGATGGTGGAAAAACAAAAATTGATTGGTCAAAATCACCCTTTGTGGCCACATTGGGAAGTTATAAAATTGATGCTTGTGTTTGGAAAGGGAATGCAAGATTTTGCAGAGTAGAAAATGAAAATCATTGGTGGAATAAGGGGCAATCTAGTACTTTGACATGGACACAAAGAAGATTGTTTAAATGGGTTAGAAAGTATCATTTGACATATGATTATTGTATGGATAATAAAAGGTTTCAAAATAATATGCCCATAGAGTGCTCTCTACCAAAATATTAGTCACGTATGGGCTAAATTTTACGTCTCTTTCAATTACGTGAAAAGATTCTTTTCTTTTTAACTTTACGTCCTCTTACATTTGTTCTTTGTATTAAATCAAAAAG

>SlXTH35

TATCCAATTCTAATTCATCCAACAAACTTTGAAATATTAACAGTTAAACATGGCTTCTTCTTCTAAATTAGTACTAGTAATGTGCTTTATGATTAGTGCTTTTGGCATTGCAATTGGGGCCAAGTTTGATCAAGAATTCGACATTACATGGGGTGATGGCAGAGCAAAAATACTTAACAATGGCGACCTCCTTACTCTCTCACTTGACAAAATCTCAGGCTCTGGTTTTCAATCCAAGAATGAATATCTGTTTGGTAAAATTGACATGCAGCTCAAACTTGTCCCAGGAAATTCTGCTGGCACTGTCACTGCTTACTATGTAAGTAATTCCCTTAATTTCTGAATGATTCTTGAGCTATATATATATACATGAATATCCCCTGAAATTGAACTCTTTTATTGCAGTTGTCATCACAAGGACCAACACATGATGAGATAGATTTTGAATTCTTGGGAAATTTAAGTGGTGATCCTTATACTCTCCATACTAATGTATTTAGTCAAGGCAAAGGAAACAGAGAACAACAATTTCATCTCTGGTTTGACCCTACTGCTGATTTCCACACGTATTCCATCACTTGGAATCCGCAACGCATCATGTAAGTAGTTAAACATTCAAACTTTCTTCAAATCTAACACATTCTTTTGGGATAAATCTTGATTTGGGGTTCTCTTATTTTTATGGTTGAAACAGATTTTATGTGGACGGAACGCCAATTAGAGAATACAAGAATAGTGAATCGATTGGAGTTTCATATCCAAAGAACCAACCCATGAGGATATATTCGAGTCTTTGGAATGCTGATGATTGGGCTACAAGAGGAGGCCTTGTTAAGACTGATTGGAGCCAAGCACCCTTTAGTGCTTCTTACAGAAACTTCAGCGCTAATGCTTGCATTCCCACTTCTTCATCTTCTTGCAGTTCCAATTCTGCAGCTTCAACTAGCAATTCATGGTTGAATGAAGAGTTAGATAACACAAGCCAAGAGAGGCTCAAATGGGTGCAGAAGAATTACATGGTTTATAATTACTGCACTGATTCAAAGCGATTTCCACAGGGATTTCCAGCAGACTGTGTTCAGAATAACTGAGCATTTATAATGAAAAAATAGTGTATTACTTTAAAAACTATTGTATTGATTCTTTTTATCTAAGATAAATAAATATGTTGGTTTTCTACCAAATTCGTCTAAGTAATGGATATATATATATATTT

>SlXTH36

ATGGTTAACTTTCAAGCAATTCTTGTTTTCATTAGTTTTTTCTTTTTTGTTAATCAATGTTTAAGTGCAAATGAGGTTCCATTTTACCAAAATTATTATCAAAAATATGGAGGTGACCATCTAACTGTTACTGACCAGGGAAAACAAGTTTGCCTAACTATAGACCAATATACAGGTTTTCTTATTTTAATTAATTAAGCATCAATCTCTAGCTTTTATTAAATTCATATAAATAATATATTTTTTGACTCAATTTTGTTGAATTCAGGTTCTGGATTTATGTCTAACCAACATTTTGGTTCTGGAGATTTTAGCATCGACTTAAAAATACCAAACAAGAACAGTACAGGAGTAATAACAACATTCTACGTACGTACATTTTTTTTTTTATACAAAACGATATATGAATTACACAATCTTATCGATTCATCATCAAAATTGTACGCAATTTATGAATGTTATCGATTAATGAGTGAACATTTACAGTTAACATCACTGCCAATGAATGGAGATCCTGGAATGCATCATGATGAGATTGATTTTGAGTTCCTTGGAGGAGATGGTATATATACATTAAATACAAATATATTTGCAAATGATGGAGGAAGTAGAGAGCAACAATTCAATCTTGATTTTGATCCTACAGAAGATTTCCATACGTATCGAATTCTTTGGAATCAACATCATATCATGTAAGTATTTTAAGTTATACATATCGTCAATATTATTAACTATGTACATGCATATTTATTTATTTAACATTTTTTTTTGAAGATTTTACGCGGATAATGTTCCAATAAGAGTTTTCAAGAACAATACTAATTATGGAGTGAATTTTCCAACACACAAAATGCACATTGAAGCAACCATATGGAATGATACAAATTGGGTTGGAGAAGTAGATTGGAGCCAAGGACCATTCAAAGCTTATTATCGCAATTTTACGATTAATGGATGTCAATATCAAGAATCAAATCGTCAAGAATGCTATAATAACAACTATTATTGGAATACAATTACCAGTCTTAGTCCAAATGAAGTTCAGGAATTTGAAACTGTGAAGGCAGAACAAATGATTTTTAGTTATTGCATGAGGAACAATAGTAGAAATTTTCCAGAATGTATATTAAATTGA

>SlXTH37

AACTATTCAACTCAAATTCATCCAACAAACTTTGAAATCTTAACAGTTAAACATGGCTTCTTCTTCTTCTAAGTTAGTACTTGTAATGTGTTTTATGATTAGTGCTTTTGGCATTGCAATTGGGGCCAAGTTTGATCAAGAATTCGACATTACATGGGGTGATGGCAGAGCAAAAATACTTAACAATGGCGACCTCCTTACTCTCTCACTTGACAAAATCTCAGGCTCTGGTTTTCAATCCAAGAATGAATATCTGTTTGGTAAAATTGACATGCAGCTCAAACTTGTCCCAAGAAATTCTGCTGGCACTGTCACTGCTTACTATGTAAGTAATTCCCTTAATTTCTGAATGATTTTTGAGCTATATATATATACATGAATATCCCCTGAAATTGAACTCTTTTATTGCAGTTGTCGTCACAAGGACCAACACATGATGAGATAGATTTTGAATTCTTGGGAAATTTAAGTGGTGATCCTTATACTCTCCATACTAATGTATTTAGTCAAGGCAAAGGAAACAGAGAACAACAATTTCATCTTTGGTTTGACCCTACTGCTGATTTCCACACTTATGCCATCACTTGGAATCCACAACGCATCATGTAAGTAGTTAAACATTCAAACTTTCTTCAAATCTAACACATTCTTTTGCGATAAATCTTGATTTGGGGTCTCTTATTTTTATGGTTGAAACAGATTTTATGTGGACGGAACGCCAATTAGAGAATACAAGAATAGTGAATCGATTGGAGTTTCATATCCAAAGAACCAACCCATGAGGATATATTCGAGTCTTTGGAATGCTGATGATTGGGCTACAAGAGGAGGCCTTGTTAAGACTGATTGGAGCCAAGCACCCTTTAGTGCTTCTTACAGAAACTTCAGTGCTAATGCTTGTATTCCCACTTCTTCATCTTCTTGCAGTTCCATTTCTGCAACTTCAACAAGCAATTCATGGTTGAATGAAGAGTTAGATAACACAAGCCAAGAGAGGCTCAAATGGGTGCAGAAGAATTACATGGTTTATGATTACTGCACTGATTCAAAGCGATTTCCACAGGGATTTCCAGCAGATTGTGTTCAGAATATCTGAGCATTAATAATGAAAAAATAGTGTATTACTTTAAAAACTATTGTATTGATTCTTTTATTGTTTTGTACCCATCAGAAGAAGATGCAATAATTATTGAGGATTAGAAACATCTTAGTTTTGTACTAAGTTATATAAACAATGAAATAGATACTTTTTTTTCTTCTAA
