# Supplementary material for: Genome-wide characterization of the xyloglucan endotransglucosylase/hydrolase gene family in Solanum lycopersicum L. and gene expression analysis in response to arbuscular mycorrhizal symbiosis
Source: PeerJ. 2023 May 3;11:e15257. doi: 10.7717/peerj.15257 (PMC10163873; doi:10.7717/peerj.15257)
Supplement: Supplemental Information 2 [file peerj-11-15257-s002.docx]

**File S2.** Coding sequences (CDS) of tomato *XTHs.*

>SlXTH1

ATGGGTATCATAAAAGGAGTTTTATTTAGTATTGTTTTGATTAATTTGTCACTTGTTGTATTTTGTGGGTATCCTAGAAGGCCAGTAGATGTGCCCTTTTGGAAAAACTATGAGCCAAGTTGGGCTAGTCACCATATTAAGTTCCTCAATGGTGGTACCACTACTGATCTTATTCTCGACAGATCTTCAGGAGCTGGATTTCAGTCAAAGAAATCATATCTGTTTGGGCATTTCAGTATGAAAATGAGGCTTGTTGGTGGAGACTCAGCTGGTGTTGTCACTGCATTTTACCTGTCATCGAATAATGCAGAGCACGATGAGATAGATTTTGAATTTTTGGGGAACAGAACTGGGCAGCCATACATATTGCAGACAAATGTATTCACAGGAGGAAAAGGAAACAGAGAACAGAGAATATATCTTTGGTTTGATCCAACCAAGGGCTACCATTCTTATTCTGTTCTTTGGAATACATACCTCATTGTGATCTTTGTGGACGACGTTCCAATTAGAGCATTCAAAAATTCGAAAGATCTTGGTGTGAAATTTCCATTCAATCAGCCCATGAAGATATACTCGAGTCTATGGGACGCAGATGATTGGGCCACAAGAGGTGGGCTTGAGAAAACCAATTGGGCCAACGCCCCATTCACCGCGTCATACACATCGTTCCACGTGGATGGATGTGAAGCTGCCACGCCACAAGAAGTCCAAGTTTGTAACACTAAAGGCATGAAATGGTGGGATCAAAAGGCCTTCCAAGATTTAGATGCATTACAGTATAGGAGACTTCGTTGGGTTCGTCAAAAATACACTGTTTATAACTATTGCACTGATAAAGCGAGGTACCCTGTTCCACCACCAGAGTGCACTAAGGACAGAGATATTTAA

>SlXTH2

ATGATCAAAACATCAAGTTGTATATTTACTTTCTTTCTTCTAATATGTTTCTTCGTAGTGGTGGCTTTTGGTGGTACTTTCGACCAAGAATTTGATGTTACATGGGGTTATGGGAGGGTGAAAATACTCGAAAACGGGCAACTTCTTACTCTTTCCCTCGATAGAAGTTCTGGCTCTGGATTTAAGTCTAAACAACAATATATGTTTGCAAAGATTGACATGAAGATCAAACTTGTCCCTGGTAATTCTGCTGGCACCGCTACTACATACTATTTATCTTCGGTGGGGTCAGCTCATGATGAGATTGACTTTGAGTTTCTTGGGAATGTAAGTGGAGAACCATATACTCTTCATACAAATGTGTATGCACAAGGCAAGGGAGATAGAGAGCAACAATTTCATCTTTGGTTTGACCCTACTAAGGATTTCCACACCTACTCTATTCTTTGGAATCCTCGAAATATCATATTTTTGGTAGATGGGACACCAATAAGACAATACAAGAATCTTGAAGCAACAAATGGAATACCTTACCCAAAGAACCAACCAATGTGGTTATACTCAAGTTTATGGAATGCTGAGGAATGGGCAACAAGAGGTGGCCTTGTAAGGACTGATTGGAGTAAAGCCCCTTTTATTGCTTCTTATAGAAACTTCAATGCCCAAACTTCCAAGAATCCCACAGCCAATTCTTGGTTAACTCAATCATTGGACAATGTTGGGTTAACAAGGATGAAATGGGTGCAAAAGAACTATATGATATACAATTATTGCACTGATACTAAACGTTTCCCTCAAGGGTTCCCTCATGAATGCACTCTAAATTAA

>SlXTH3

ATGGCTTCTTCTTCTTCTAAATTAGTACTTGTAATGTGTTTTATGATTAGTGCTTTTGGCATTGCAATTGGGGCCAAGTTTGATCAAGAATTCGACATTACATGGGGTGATGGCAGAGCAAAAATACTTAACAATGGCGACCTCCTTACTCTCTCACTTGACAAAATCTCAGGCTCTGGTTTTCAATCCAAGAATGAATATCTCTTTGGTAAAATTGACATGCAGCTCAAACTTGTCCCAGGAAATTCTGCTGGCACTGTCACTGCTTACTATTTGTCATCACAAGGACCAACACATGATGAGATAGATTTTGAATTCTTGGGAAATTTAAGTGGTGATCCTTATACTCTCCATACTAATGTATTTAGTCAAGGCAAAGGAAACAGAGAACAACAATTTCATCTCTGGTTTGACCCTACTGCTGATTTCCACACGTATTCCATCACTTGGAATCCACAACGCATCATATTTTATGTGGACGGAACGCCAATTAGAGAATACAAGAATAGTGAATCGATTGGAGTTTCATATCCAAAGAACCAACCCATGAGGATATATTCGAGTCTTTGGAATGCTGATGATTGGGCTACAAGAGGAGGCCTTGTTAAGACTGATTGGAGCCAAGCACCCTTTAGTGCTTCTTACAGAAACTTCAGTGCTAATGCTTGCATTCCCACTTCTTCATCTTCTTGCAGTTCCAATTCTGCAGCTTCAACAAGCAATTCATGGTTGAATGAAGAGTTAGATAACACAAGCCAAGAGAGGCTCAAATGGGTGCAGAAGAATTACATGGTTTATGATTACTGCACTGATTCAAAGCGATTTCCACAGGGATTTCCAGCAGATTGTGTTCAGAATATCTGA

>SlXTH4

ATGAAGGGAGTTTTAGTTGCTTTTGTTTTGATTAATTTGTCAATATTGGCAAGTTGTGGGGCTCCAAGGAAGGTAATTGATGTGCCTTTTTGGAACAACTATGAACCAAGTTGGTCTAGTCACCATATTAAGTACCTTAATGGTGGTACTACGGCTGAACTTCTTCTTGACAAATCCTCTGGAACTGGATTTCAATCAAAGAGATCGTATCTATTTGGTCATTTCAGCATGAAAATGAAGCTTGTTGGAGGAGATTCTGCTGGTGTTGTCACTGCTTTTTATTTATCATCGACTAATGCTGAACACGATGAGATAGATTTCGAATTCCTCGGGAATAGAACCGGTCAGCCATACATATTGCAGACTAATGTGTTCACAGGAGGCAAAGGAGACAGAGAACAGAGGATCTATCTTTGGTTTGATCCAACCAAGGACTTTCATTCATATTCTGTTCTTTGGAACACTTACCAAATTGCGATTTTTGTGGATGATGTCCCAATAAGAGTATTCAAGAATTCAAAAGACATAGGAGTGAAATTTCCGTTCAATCAGCCAATGAAGATCTACTCAAGCCTATGGAACGCGGATGATTGGGCTACAAGAGGAGGGTTAGAGAAAACTAATTGGTCTGGGGCGCCATTCATCGCTTCCTATACTTCATTCCACATTGATGGATGTGAGGCTGTCACACCACAAGAGGTACAAGTTTGTAACACCAATGGCATGAAATGGTGGGATCAAAAGGCTTTCCAAGATTTAGATGGCCCTGAATATAGAAAACTTCATAGGGTTAGACAAAATTTCACAATATATAACTATTGTACTGATAGAAAAAGGTACCCTACACTTCCTCTAGAGTGTACAAGGGATAGAGATCTTTAA

>SlXTH5

ATGAAAATATGTCTTTCTGTCTTATTTTTTTTCCATGTTTGGTTTTGTAGAGCTTTTAATGATGTCTCAACAATTCCTTTTAACAAAGGATTCAGCCATCTCTTTGGTGATGGAAATATTCTTCATGCTAACGATGATAACAGCCTTCAACTTCATCTCAACCAAAACACAGGTTCAGGGTTCAAGTCTTCTGACCTTTACAACCATGGTTTCTTCAGTGCTAAAATTAAATTGCCATCAGATTATACTGCAGGAATAGTTGTTGCCTTCTATACGACGAATCAAGATGTATTTAAGAAGACACACGATGAACTAGATTTTGAATTTTTGGGAAATATAAAAGGAAAAGCATGGAGATTTCAAACAAATATGTATGGAAATGGAAGCACACATAGAGGAAGAGAAGAAAGATATACTCTATGGTTTGATCCTTCTAAAGAGTTCCATCGTTATAGTATTTTGTGGACCAACAAAAACATCATATTTTATATAGATGATGTTCCAATTAGAGAAATTGTAAGAAATGATGCAATGGGTGGAGACTACCCATCAAAGCCAATGGGCCTATATGCAACAATTTGGGATGCTTCAGATTGGGCTACTTCAGGTGGAAAATACAAAACAAATTACAAATATGCACCATTTATAGCTGAATTTACTGATTTAGTACTCAATGGATGTGCAATGGACCCATTGGAACAAGTTGTAAACCCTAGTCTTTGTGATGAAAAAGATGTTGAACTCCAAAAGTCGGATTTTTCAAGGATTACATCGAGACAAAGAATGTCCATGAAAAGATTTAGGGCGAAATATATGTACTATTCTTACTGTTACGATTCATTGAGATACTCAGTGCCACCACCAGAGTGCGAGATTGATCCAGTTGAGCAACAACATTTTAAAGAGACTGGAAGGTTGAAGTTTATAAACAAGCACCACGGACATCGTCATCCTAAGAAAACAAAAAGTGAAGTTCTTGATGCTAGGAAGTATGGAAATGAAGATGAAGAGTGA

>SlXTH6

ATGGAATTCCTTCTTTATTTACTTTTATTTTTCTTACTCAATTCAAGATTAATCAATGCTCAAGGTCCCCCTTCACCTGGCTACTATCCTAGTTCTAGGGCACAATCTATAGGATTTAACCAAGGTTTTAGAAACCTTTGGGGTCCTCAACATCAATCATTGGACCAAAGTACCTTAACTATATGGCTTGATAAAAATTCAGGAGGAAGTGGTTTTAAATCTCTAAAGAATTATCGTTCTGGTTATTTTGGGAGTAGTATTAAGCTACAACCTGGTTTTACTGCTGGAATTATTACTTCTTTTTATCTTTCAAATAATCAAGATTATCCGGGGAACCATGATGAAATTGATATTGAATTTCTTGGAACAACACCAAACAAGCCATATACTTTACAAACAAATGTGTATATAAGAGGAAGTGGAGATGGAAATATTATTGGAAGAGAAATGAAATTTCATCTTTGGTTTGATCCAACAAAAGATTATCACAATTATGCAATCCTTTGGGACCCCAATGAGATCATATTTTTTGTCGATGATGTCCCTATTAGAAGATACCCTAAAAAAAATGATGCAACATTTCCACAAAGACCTATGTATGTCTATGGTTCAATTTGGGATGCATCATCTTGGGCAACGGAGGAAGGACGAATTAAAGCGGATTATCGATACCAACCTTTTATCGGAAAATATAGTAATAATTTCAAGGTTGAAGGTTGCGCGGCCTACGAGAGTCCCTCTTGTCGTCGAGCGCCTTCTAGCTCTCCTTCGGGGGGTGGAGGGTTGAGTCGACAACAGATAGAGGCTATGTTGTGGGTGCATAGGAACTATAAGGTGTACGATTATTGTAGGGATCCTAGGAGGGACCATACTCACACACCTGAGTGTTAG

>SlXTH7

ATGGCCACATTGACTTGCTCTTCCTTAAAAAATTCAGCTTTTGTTCTAATATTGGTATATGCCTTGACCTTTTCATTCTCACTAGTAAGTGCACGACCCGCCACTTTTTTACAGGATTTTAAAATCGCTTGGTCCGACTCTCACATCAAACAACTCGATGGCGGCAGGGGAATTCAACTTATTCTCGATCAAAACTCAGGATGTGGATTTGCTTCGAGAAGCAAATACCTGTTTGGACGTGTTAGCATGAAGATCAAGCTCGTTCCAGGTGACTCTGCAGGAACTGTTACCGCCTTTTACATGAATTCGGACACAGATAACGTAAGAGACGAACTTGACTTCGAATTCTTGGGAAACCGGACAGGGCAGCCGTACACTGTTCAAACGAATGTTTATGTCCACGGAAAAGGTGACAAGGAACAAAGGGTTAACCTTTGGTTCGATCCATCCGCTGATTTTCACACATACACCATTTTTTGGAACCATCATCAAGCCGTGTTCTCAGTGGATGGAATACCCATTAGAGTGTACAAGAACAACGAAGCAAAAGGAATCCCATTCCCCAAATTTCAACCCATGGGTGTCTACTCAACATTGTGGGAAGCTGACGACTGGGCTACAAGAGGTGGCTTAGAGAAAATAAATTGGAGCAAATCCCCATTTTACGCATACTACAAGGATTTTGACATTGAAGGATGTGCAATGCCAGGACCAGCAAATTGTGCCTCCAACCCAAGTAATTGGTGGGAAGGACCTTCTTATCAACAACTGAGCCCAGTACAAGCAAGGCAATATCGTTGGGTTCGAATGAATCACATGATCTATGATTATTGCACAGACAAATCGAGAAACCCCGTTCCCCCACCAGAATGTAGGGCCGGAATTTGA

>SlXTH8

ATGGTGAATTTTCTTCTGGAAATTTTTATATTTTGCTATGTTGTTGTATTAGTTTCTGGATTTTCAGAAAATCTCGAAACGTCGTCGTTTAATGAAGGATATTCACAACTTTTTGGTCATGATAATCTTATGGTCATTCAAGATGGAAAATCAGTTCATATTTCTCTAGATGAAAGAACAGGAGCTGGATTTGTGTCACAAGACTTGTACCTTCATGGCTTATTCAGTGCTTCTATTAAATTACCAGAAGATTACACTGCTGGAGTGGTGGTTGCATTTTATATGTCAAATGGAGACATGTTTGAGAAGAATCATGATGAAATTGACTTTGAGTTTTTGGGAAATATTAGAGCAAAAAATTGGAGGATTCAAACTAATATTTATGGAAATGGTAGCACAAATGTTGGTAGAGAAGAAAGATATGGACTTTGGTTTGATCCAACTGAAGATTTTCATACATATACAATTCTTTGGACTGACAGCCACATCATCTTTTATGTAGATAATGTACCTATAAGAGAGATCAAGAGAACACAAGCAATGAGTGAGGACTTCCCTTCTAAGCCAATGTCTTTATATGGTACAATATGGGATGGCTCTAGTTGGGCTACTAATGGGGGTAAATACAAAGTCAATTACAAATATGCCCCTTACGTCGCGAAGTTCTCCGATTTCGTCCTCCATGGATGTGGTGTTGATCCAATTGAATTGTCTCCCAAGTGTGATATAGTCCTGGATTCTGCATCCATCCCAACTAGAATATCCCCTGACCAAAGGCGAAAAATGGAGAGGTTTCGAAACAAGTACTTGCAATATTCATATTGCTATGACCGGACACGATACAATGTTCCTCAATCTGAATGTGTGATTGATCCTAAGGAAGCTAATCGCCTCCGAGGATTCGACCCTATGACCTTTGGTGGTGTCCCTCGTCATCAGAACAAACGACACCACCAAAGGCAATCGAGGAGGGAAGATACGTCCGCGAAATAA

>SlXTH9

ATGTCTTCTAAATTTTCATCAACATTGCTTCTTCTTATTTCAATACTAATGAGTATCCAATTACTAGCCTCAGCTGGTAATTTCTATAGAGATGTAGACATAACTTGGGGCGAAGGACGCGGTAAAATACAAGAAGGCGGTAGAGGCCTTGCCCTATCGCTTGATAAACTTTCTGGCTCTGGCTTTCAATCCAAAAATGAGTACCTTTTTGGAAGATTCGATATGCAACTTAAACTCGTCCCTAAAAACTCTGCTGGCACTGTAACAACTTTCTTCTTATCTTCACAAGGAGAAGGACATGATGAGATCGATTTCGAGTTCTTAGGCAATGTCTCTGGCCAGCCTTACACTATCCATACCAATGTATACACACAAGGAAAAGGAAACAAAGAACAACAATTTCATCTTTGGTTTGATCCAACTGCCGCGTTTCACACTTACACCATCGTCTGGAATCCTCATCGCATAGTGTTCTTAGTAGATAACAGTCCAATTAGAGTGTTCAACAACCATGAAAGCATGGGAATTCCATTTCCCAAGAGTCAAGCAATGAAAGTATACTGCAGTTTATGGAATATTGGGCACTTGCACCATTCACTGCTTATTACAGAAACATTAACATTGATGGTTGTGCAGTATCATCAGGTAACCTCTTCATGTAAGTCCATCGGTTCAATAAACAACGCGAAGCCATGGCAAACACATGAACTTGATGGTAAGGGACGGAATAGACTACGATGGGTGCAGACCAAACACATGGTTTACAATTACTGTGCTGATTCTAAGAGGTTTCCACAAGGCTTTTCTGCTGAATGCAAGAGTTCAAGATTTTAA

>SlXTH10

ATGTTGCTGCAGCTTTCTCTTCTTACACTAGTCTTACTATCCCCTGTTTCCGCTGATAATTTCTACCAAGACGCGGCGGTCACGTTTGGTGACCAGCGCGCTCAGATACAAGATGGAGGGCGCCTTCTCACATTGTCACTTGATAAAATTTCAGGTTCCGGATTTCAGTCTAAGAATGAGTATTTATTCGGAAGGTTCGATATGCAGCTTAAACTCGTACCTGGAAATTCTGCTGGCACTGTCACCACATTCTATTTGTCTTCTCAAGGAGCAGGGCATGATGAAATTGATTTTGAGTTTCTAGGAAATTCATCAGGACTACCTTACACGGTTCATACCAATGTTTACTCTCAAGGAAAAGGCAATAAAGAACAACAATTTCGTCTCTGGTTTGATCCAACTTCGTCGTTCCACACTTACTCTATTGTTTGGAACTCTCAACGGATCATATTTTTGGTGGATAATATCCCAATTAGAGTGTTCAACAACCACGAAGCACTTGGTGTTGCATACCCAAAGAATCAAGCAATGAGAGTTTACGCGAGTCTATGGAATGCTGATGATTGGGCTACACAAGGAGGACGGGTGAAGACAGATTGGTCTATGGCTCCGTTTACAGCTTCTTACAGGAATTTCAATACAAATGCTTGTGTTTGGTCAGCTGCTACGTCTACTTCGTCTTGTGGAGGTTCTAAGACTGAGTCAGTAAACAATGATGAGACATGGCAAACGCAACAACTGAACGCTAATGGAAGAAATAGAATACGATGGGTTCAGCAGAAGTACATGATCTACAATTACTGTGCAGATGCTAATAGGTTCTCTCAAGGCTTTTCTCCTGAATGCAAGCGTTCAAGGTTCTAA

>SlXTH11

ATGTTGCTGCAGCAGCTATCTGTTCTTGCTCTACTTCTCTTGCTATGTCCTGTTTGGGCTGACAATTTCTACCAAGATGCAACGGTTACCTTTGGTGATCAGCGAGCTCAGATACAAGATGGTGGGCGCCTTCTCGCCTTGTCCCTTGACAAAATTTCAGGTTCAGGATTTCAGTCTAAGAATGAATATTTATTTGGAAGGTTCGATATGCAGCTCAAACTAGTACCTGGAAATTCTGCTGGCACTGTCACTACCTTCTATTTGTCTTCTCAAGGAGCAGGGCACGACGAAATTGATTTTGAGTTTCTGGGAAATTCATCAGGCCAACCGTACACGGTTCATACTAATGTCTACTCTCAAGGAAAAGGCAACAAAGAACAACAGTTTCGCCTATGGTTTGATCCCACCTCGTCGTTCCACACCTACTCTATTGTTTGGAACTCTCAACGCATCATATTTTTGGTGGATAATATCCCAATAAGAGTATTCAACAACCACGAAAAGCTTGGTGTTGCATTCCCAAAGAACCAAGCAATGAGAGTTTATGCCAGTTTATGGAATGCTGATGACTGGGCAACACAAGGAGGGCGAGTGAAGACGGATTGGTCAATGGCTCCGTTTACAGCTTCTTACAGGAATTTCAACACAAATGCTTGTGTTTGGTCAGCTGCATCGTCTACTTCGTCCTGTGGAGGCTCTAAGACTGATTCAGTAAACAATGATCAGGCATGGCAAACTCAAGAACTGAACGGTAATGACAGAAATAGGCTTCGATGGGTTCAGCAGAAATACATGATCTACAATTACTGTGCAGATGCTAAAAGGTTCTCTCAAGGCCTTTCTCCTGAATGCAAACGTTCAAGGTTCTAA

>SlXTH12

ATGGGGTCTTTTACCCATTATGGGTTCTTGATGTTAGCACTTTTATTTAGTTCTTGCATGGTTACTTATGGTGGAAATTTTTATCAAGAATTTGACTTCACTTGGGGTGGCAATAGAGCCAAGATTTTCAATGGAGGTCAACTTTTATCTTTATCTTTAGACAAAGTTTCTGGCTCTGGTTTTCAATCAAAAAAAGAACATCTCTTTGGAAGAATTGATATGCAAATCAAACTCGTTGCTGGAAACTCTGCTGGCACTGTCACAACATATTACTTATCTTCTCAAGGACCCACTCATGATGAAATTGACTTTGAGTTCTTGGGAAATGTTACTGGTGAACCTTATATTCTACACACAAATATTTATGCCCAAGGCAAGGGTAACAAAGAGCAACAATTTTACCTTTGGTTTGACCCTACAAAGAACTTCCACACCTACTCCATCATTTGGAAACCCCAACACATCATATTTTTGGTGGACAACACACCAATAAGAGTATACAAAAATGCTGAATCAGTTGGTGTACCATTTCCAAAGAATCAGCCCATGAGGATTTACTCAAGCCTTTGGAATGCTGATGATTGGGCCACAAGAGGAGGCCTAGTAAAAACTGATTGGGCCCAAGCCCCATTCACAGCCTACTATAGAAACTACATGGCCCAAAGCTTTAGCCCATCACAATTTTCTGATCAAAAATGGCAAAATCAAGAACTTGATTCTAATGGCAGAAGAAGACTTAGATGGGTTCAAAAGAATTTCATGATTTATAATTATTGTACTGATATTAAGAGGTTTCCTCAAGGTTTTCCTCCAGAATGTAGAAGATTTTGA

>SlXTH13

ATGGCATTATTTTCATCAAGAAATTCATCAAGATCTAGGTCCTCTCTTCCATATTTGGTGTTTCTCTTAATTGCTGCCTTTTTTGTCTTCAAGGTAGATATACTCATATCTCAGTCTTTTAGTTCAGCCCGTCGCAACCTGGAAAAAACTCCTAATCGTATCGTTGTGAACCCCCAAAAATCATCGGAAGAACGTGTTGTTGACAGCCTCCCTGTAGTTTTAGTAAATGGTACATTTGACCAGCATATTATGATATCATGGGGAGATGACAGAGGAAAAATACTTGAAAATGGAGAGCTTTTAACACTCTCCTTAGACAAGAAGTCTGGATCAGGCTTTCAGTCTAAAAAAGAGTACCTCTTTGCTAAAATTGATATGCAAATTAAGCTCGTCCCTGGAAATTCTGCTGGCACTGTTACTACGTTTTACCTATCATCACAAGGGAACAAGCATGATGAAATAGATTTTGAATTCTTGGGGAATTCAACAGGGAACCCTTATACTCTTCATACAAATGTTTTTAGTTTAGGCAAAGGCAATAGGGAACAACAATTCTTCTTGTGGTTTGATCCAACTGCAGATTATCACACATATTCAATCCTATGGAATTCTAAATGTATTATATTCTATGTTGATGATATACCAATTAGAGAATACAAAAATCCAGAGAGACTTGGTCTTTCATATTTAAAATACCAACCAATGAGACTATACTCAAGTCTATGGAACGCAGATGATTGGGCTACACAAGGTGGTCGTATCAAAACCAATTGGGAACTAGCACCTTTTGTAGCGTCCTACAAAAATTTCACATATGAAGCTTGTATTTATTCAAGATTAACTAGTTCATCTTCATGTGATATCGACTCTCCAACTCCTATCAACAACGCTTGGTTAACATATGAGTTAGATCGAACAAGTCGTGTTAGAATGAAAGCTTTGCAGAAAAAACATATGATTTATGATTATTGCAACGATAAATGGAGATTTCCTAAAGGTCCTGCTCCTGAATGCAAGCTTCTTCAATAA

>SlXTH14

ATGTCAACAATTTTTTTCCTTCCTATTTTTCTTTGTTTTATTTTTCTCCATTCAACTAATGCTAATTATTGGCCAATTTCACCTGGATATTATCCAAGTACAAAATTTAAATCCATGAGTTTTTATCAAGGATTTAAGAATCTCTGGGGTCCTAATCACCAGAGTGTAGATAACAATGGCATTAATATTTGGCTTGATAGAAATTCAGGCAGTGGATTCAAGTCGGTTAAACCGTTTCGATCCGGGTATTTTGGGGCTTCTATTAAACTCCAACCCGGTTATACGGCCGGAGTCATCACCGCTTTCTACCTTTCAAATAATGAAGCACATCCAGGGTTCCATGATGAAGTGGACATAGAATTTCTTGGAACAACATTTGGAAAACCATATACATTACAAACAAATGTATATATTAGAGGAAGTGGTGATGGAAAAATTATAGGAAGAGAAATGAAATTTCATTTGTGGTTTGATCCCACAAAAAATTTTCATCACTATGCTATTTTGTGGAGTCCGAGAGAAATCATATTTTTGGTGGATGATGTACCAATAAGGAGGTATGCAAGGAGAAGTGATGCAACATTTCCATTGAGGCCAATGTGGTTATATGGATCCATATGGGATGCATCTTCATGGGCTACTGAGAATGGAAAATACAAAGCTGATTATAACTACCAACCATTCTATGGAAAATTCACGAATTTCAAGGCGAGTGGTTGCACCGCCTACTCGTCTCGATGGTGTCGACCTGTGTCTGCCTCGCCCTATAGATCCGGTGGCCTTAGCAGGCAACAACGTCAGGCCATGAATTGGGTTCGAAGTCACTATATGGTGTATGATTATTGCAGGGACTTTAAAAGAGATCACTCCCTTACACCAGAATGTTGGCGCAAGTGA

>SlXTH15

ATGGCTTCTCCTATAGCTTATTTTCTTGTACTTAGTGCAATAATTGTTGTACTTTTTTCATCAACACAAGCTGAAGTACAAGGTTCATTTGATGACAATTTTAGTAAAAGTTGTCCAGAAACTCATTTCAAGACTTCTGAAGATGGACAGATCTGGTATTTATCATTGGACAAAAAAGCAGGATGTGGATTTATGACCAAACAGAAATATAGATTTGGGTGGTTTAGCATGAAGTTGAAATTGGTGGGAGGTGACTCTGCTGGTGTTGTGACAGCTTACTATATGTGCACAGAAGATGGAGCAGGACCAACAAGAGATGAATTAGATTTTGAGTTCTTGGGGAATAGGACAGGTGAACCTTATCTTATTCAAACAAATGTGTACAAGAATGGAACTGGTAATCGTGAGATGAGACATGTTTTATGGTTTGACCCCACTGAGGATTTTCACACCTACTCAGTTCTTTGGAATACTCACCAAATTGTGTTTTTCGTGGATAAGGTACCAATAAGAGTGTACAAAAACGCGAATTACACAAACAATTTCTTCCCAAATGAGAAGCCAATGTACTTATTTTCAAGTATATGGAATGCAGATGATTGGGCTACAAGAGGTGGTTTAGAGAAAACAAATTGGAAAAATCAACCATTTGTTTCATCATACAAAGATTTTAGTGTGGATGGTTGTCAATGGGAAGATCCATATCCATCTTGTGTTTCAACAACAACACAAAATTGGTGGGATCAATATGATTCATGGCATTTATCAAGTGATCAAAAATTGGATTATGCTTGGGTACAAAGAAATCTTGTCATTTATGATTATTGTCAAGATACTGAAAGATTTCCAAAAAAACCTGAGGAGTGTTGGTTAAATCCATGGGAATAA

>SlXTH16

ATGGTGAGTTTTAATTGGGTTTTTTCGAGCTTTGTGATGCTGTTTATGGTGGGTTTGGTTAGCTCTGCAAAATTTGAGGAGCTTTATCAACCCAGTTGGGCTTTTGACCATTTGACAACTGAAGGAGAGATTCTTAGAATGAAATTGGATCATCTTTCTGGTACTGGGTTTCAATCTAAGAGCAAATATATGTTTGGGAAAGTTACTGTTCAGATTAAGCTTGTTGAAGGTGACTCTGCTGGAACTGTCACTGCATTCTATATGTCATCAGATGGACCAACCCACAATGAGTTTGATTTTGAATTTTTAGGCAATACAACTGGTGAACCATATACAGTACAAACAAATGTGTATGTCAATGGTGTTGGTAACAGAGAACAGAGATTGAAGCTTTGGTTCGACCCATCGAAGGATTTTCACTCTTATTCCATCATGTGGAATCAACGTCAAGTTGTATTCTTGGTAGATGAAACCCCTGTTCGAGTGCATTCGAATTTGGAGCATAGAGGAATCCCATACCCCAAGGATCAACCAATGGGTGTGTATAGTTCGATTTGGAATGCAGATGATTGGGCTACACAAGGCGGGCTCGTTAAGACTGATTGGTCACACGCGCCCTTCGTAGCATCCTATAAGGGATTTGAGATTAACGGTTGTGAGTGCCCGGCAACTGTTGCAGCTGCTGAGAATACTCGGCGTTGCAGCAGTAATGGGCAGAAGAAGTACTGGTGGGATGAACCTGTTATGTCCGAGTTGAATTTGCACCAGAGTCACCAGCTGATATGGGTCAGGGCGAACCATATGGTTTACGATTATTGCACGGATAGCGCTAGGTTCCCTGTTGCCCCTGTTGAGTGCCAGCACCACCAGCACAAGACGAATCATAACTAG

>SlXTH17

ATGGCTAATTCTCATTTACTTTTAATTTCCATTGTATTAATGGGCAATTTAGTGGCTGTATTAGCAGCTGGTAATTTTAATGACCTTACAGAAATCACTTGGGGTGATGGACGTGGTAAAATATTAGATGGAGGTAAAGGTCTCTCTTTGTCACTTGATAATTATTCCGGGTCGGGTTTTCAATCGAAAAATGAATATCTCTACGGAAGATTCGACATGCAACTCAAACTCGTCCCTAAAAACTCTGCTGGCACTGTCACCACGTTCTTTCTATCGTCACAAGGAGAAGGACACGATGAGATCGATTTCGAGTTCTTGGGAAATGTGACTGGTGAGCCTTATACGGTACACACCAATGTTTATTCTCAAGGAAAAGGAAACAAAGAACAACAATTTCACCTTTGGTTCGATCCAACTGCAGCATTTCACACTTACACCATTGTTTGGAACGCTAACCGCATAGTGTTTTTGGTGGATCAGATTCCAATTAGAGTATACAACAACCATGAGAGCATTGGAATTGCATACCCCAAAAGTCAACCAATGAAAGTCTATTGTAGTTTATGGAATGCAGATGAATGGGCTACACAAGGTGGTAGAGTCAAAACTGATTGGTCACAAGCACCATTTACTGCCTATTATAGGAACATTAACATTGATGGCTGCGTCGTTAAATCAGGCGCTTCCTCATGTGCCTCACGGTCTACTGAATCCACGAACAGTGCTAAGTCGTGGGAGACACATGAGCTTGATGCTAAGGGTCGGAACAGGGTCCGATGGGTACAGAGCAAACATATGGTTTATAATTATTGCGCGGATTCTAAGAGGTTTCCTCAAGGATATTCACAAGAGTGTAAACGATCAAGGTTTTAA

>SlXTH18

ATGGCAAAACTCATAGATTTTAATTCTTTGGTTTTGATGATTATTGCAATAATTGCATTATTTCATTCATATGTAGTCATTGGGATGACATCAAGTAGCATGTATGTTAATTGGGGTGCTCATCATTGTAAACTTCTAGGGGATGATCTTCAACTTGTTCTTGATAAATCTGCAGGCTCTGGTGCTCAATCAAAAAGATCATTTCTTTTTGGTAGCTTTGAAATGCTTATCAAGTTAGTACCCAATAATTCTGCTGGAACTGTCACAACATACTATCTATCTTCTACTGGTACCAAGCATGATGAAATCGATTTCGAGTTTTTAGGAAATATATCAGGACAACCTTATATTATACACACAAATATTTACACCCAAGGTGTTGGAAATAGAGAGCAACAATTTTACCCTTGGTTTGATCCAACTGCTGATTTTCACAACTATACTATTCATTGGAACCCTAACGCCGTTGTATGGTACATTGATAGTATTCCAATTAGGGTTTTTAGAAACTACCAATCCAAAGGCATTCCATTCCCAAACAAACAAGGAATGAGAGTCTACACTAGTCTATGGAATGCAGATGATTGGGCAACAAGAGGTGGTCTTGTTAAAATTGATTGGACAAATGCACCATTTATTGCAACTTATAGAAAATTTAGACCAAGAGCTTGTTATTGGAATGGACCAATGAGTATTTCCCAATGTTCAATTCCTACAAAAACCAATTGGTGGAGTTCACCTACATACAATAAATTGAGTGCAAATAAACTTGGTCAAATGAACTCAATGAGGAGTAAGTATATGATCTATGATTATTGCAAAGATGTGAAAAGATTCAAAGGAGTTATACCTATTGAGTGCTCATTGCCACAATACTAG

>SlXTH19

ATGCAATTCAAAAACACATACACCATGAAGACTACTTTCTTACTTTTCTTGATTCTAAGTTTCTTCTTCTCTGCTTTGGCTGGAAATTTTAACCAAGATTTTGATATTACATGGGGTGATGACCGCGCCAAAATACTCGAAAACGGACAACTTATGACCCTTTCCCTCGATAAAGTCTCTGGCTCTGGTTTTCGATCCAAAAACCAGTATTTGTTTGGAAAGATTGATTTGAAAATCAAACTTGTGCCTGGTAACTCTGCTGGCACCGTTACAACATACTATCTATCTTCAATAGGATCAAGTCATGATGAGATTGACTTTGAGTTTCTTGGGAATTTGAGTGGTGACCCATATATTCTTCATACAAATGTATTCACACAAGGAAAGGGAGATAGAGAGCAACAATTTTATCTTTGGTTTGATCCCACTAAGGACTTTCATACATATTCTATTCTTTGGAATCCTCAAAGCATCATATTTTCAGTAGATGGGACACCAATTAGGCAATTCAAGAATCTAGAATCAAGTGGTATCCCCTATCCAAAGTCACAACCAATGTGGATATACTCAAGTTTATGGAATGCAGATGATTGGGCTACAAGAGGTGGACTTGTCAAAATTGATTGGACTAAAGCCCCATTTATTGCTTCATATACAAATTTCAATGCACAAGCTTGTGTATGGTCTTCAACTTCAACTTCTTCTTCTTGCAATTCTACTACACAAGATTCTTGGCTAAGTGAAAACTTGGATATAACAGGCAAATCAAGGATTAAATGGGTGCAAAATAATTACATGATTTATAATTATTGTAATGATATTAAACGTTTTCCTCAAGGGTTTCCTCTTGAGTGTTCTCTCAATTAG

>SlXTH20

ATGCCTTTTCTATTTTCCTTTAATATTAGACTTATTTTAGTTTTAGTATTTATAAGTTGTATGGTTGTTAAATATTGTGCTAGTAATGATCTTAATCAAGATTTTGATATTACATGGGGAAATGAAAGGGGGAAAATACTAAATAATGGTGAAATACTTACTCTTACACTTGATAATATTTCAGGTTCTGGATTTCAATCAAAGAAGGAATATTTATTTGGGAAAATTGATATGCAAATAAAATTAGTTCAAGGGAACTCTGCTGGCACTGTCACCGCATACTATTTGTCATCACAAGGATCAAGTCATGATGAGATAGATTTTGAGTTTCTTGGTAATTTAAGTGGAGAGCCATATACACTACATACAAATGTGTATACACAAGGCAAAGGTGATAGAGAGCAACAATTTCACTTGTGGTTTGATCCTGCTAATGATTTTCATACTTATTCTATCCTTTGGAATCCACAAACTATTGTATTTTCAGTGGACAATGTACCAATAAGGGAGTTTAAAAATATGGAAAACATTGGAGTTGCATTTCCAAAATCTCAATCAATGAAACTTTATTCAAGTTTATGGAATGCTGATGAATGGGCCACAAGAGGTGGGCTTATCAAGACTGATTGGGCCCAAGCCCCATTTACAGCCTCTTATAGAAATTTCAATGCCAATATTTGCAATAATAATAATAATAATAATGATTCTTGCAAATATTTGGTAGAAAATTTGGATCCTGTGAATGAAGAAAAATTGAGAAGGGTGCAACAAAAATACATGATATATAATTATTGTACTGATAATAAGAGATTTCCTCAAGGTTTTCCTCTAGAGTGTAGTGTTAGTTAA

>SlXTH21

ATGGTGAACTATTATATGTTCTTTTTCATATTTTTGTCTTGTATTCTTGTTTTGGTTTCTGGGTTTTCAAGAAATCTGCCAATTTTAGCTTTTGATGAAGGTTACTCTCATTTATTTGGTGATAATAATCTTATGATCCTTAAAGATGGAAAATCAGTTCATATTTCTCTTGACAAAAGAACAGGGGCTGGATTTGTGTCTCAAGACCTTTATTTTCATGGATTTTTTAGTGCTTCTATTAAGTTACCTGCAGATTATACAGCTGGTGTTGTTGTTGCATTTTATATGTCTAATGGGGATATGTTTGAGAAGAACCATGATGAAATTGATTTTGAGTTTTTGGGAAATATTAGAGGCAAAGACTGGAGAATTCAGACTAATATTTATGGGAATGGTAGCACAAATGTTGGCAGAGAAGAAAGATATGGACTTTGGTTTGATCCTTCTGAAGATTTTCATCAATACAGTATCCTTTGGACTGAGAATTTGATCATCTTTTATGTAGATAATGTCCCCATAAGAGAGATCAAGAGGACAAAAGCCATGGGTGGGGACTTCCCATCTAAGCCAATGTCCTTGATAGCTACAATATGGGATGGTTCTAATTGGGCTACAAATGGTGGAAAATACAAAGTCAATTACAAATACGCCCCGTATATCGCTGAGTTCTCCGATTTCATCCTCCACGGATGCGCGGTTGATCCAATCGAACTGTCATCCAAATGTGACAACACTACGCCAAAAACTCCAACGATCCCTACCGATATCACCCTTGACCAAAGACGAAAGATGGAGAACTTCAGAAAGAAGCAAATGCAATATTCATACTGCTATGACAAGACCAGGTACAAGGTCCCTCCTCCCGAGTGCGTGATCGACCCTAAGGAAGCCGAACGACTCCGAGCCTTTGACCCCGTTACATTTGGAGGATCCCACCACCATCATGGGAGACGACATCACCGGAGCAGACCAAAGTTGAAGGGTGATGATGATGTATCCT

>SlXTH22

ATGGGCAGCTCTCTAGTTCTTTCATTGGCTAATTTGTTGATTATTTCAACAATTGTGTCATTTGGTTCTTTAGTTATGGTTAATGGTATTTTCTCAGATAATATGTACATTAATTGGGGTTCTCATCATTCTTGGATGCAAGGAGATGATCTTCAACTTGTCCTTGATCAATCTTCTGGTTCAGGTGTACAATCAAAAGGAACATTTCTATTTGGAAGTATAGAAATGCAAATTAAATTGGTACCTGGAAATTCTGCTGGAACAGTCACTGCATACTATTTATCCTCAACTGGTGACAAGCATGATGAAATTGACTTTGAGTTTCTAGGAAATGTATCAGGACAACCATATATTATACACACAAATATATTTACTCAAGGTGCTGGAGGCAGGGAACAACAATTTTATCCATGGTTTGATCCAACTGCTGATTATCATAATTATACCATTCATTGGAACCCTAATGCAGTCGTATGGTACGTTGACGATATACCAATTAGAGTCTATAAAAACTATCAGAGTCAAGATATTCCCTATCCGAACGCGCAAGCAATGGGGGTTTACTCTAGCCTTTGGAATGCTGATAGTTGGGCAACTAGAGGTGGTCTTGTCAAATGTGACTGGACCAATGCACCATTTATAGCCAAGTATCGAAATTTCGCCCCACGGGCCTGTGCCTGGAACGGACCTATTAGCATTAGTCAATGTGCAACTCAAACTCCAAGTAACTGGTATACTGCTCCTGAGTATAATCAATTGAGTTACGCGAAACAAGGTCAAATGGAATGGGTTAGGAGCAATTACATGATTTATGATTATTGTAAAGATACGAAGCGATTTAACGGACAATTTCCTGGAGAGTGTTTTAAACCTCAATTTTAA

>SlXTH23

ATGGAGAGCAATGCTTCTTCAATGGCTCGTGTTCTTTTGATTTTATCAGTAATTTTTACCCTTTTTTCATCATCAAATGGTGTAGTTGGAGGTGCATTTGAAGAAAATTTCAGTAAAAGTTGTCCTGGTACACATTTCAAGACTTCTAAAGATGGACAGATCTGGTATCTTACCTTAGACCAAGTATCAGATTGTGGGTTCATAACAAAACAGAGCTATAGATTTGGTTGGTATAGCACAAAGTTGAAATTAGTAGGAGGTGACTCTGCTGGTGTTGTGACAGCATTTTATATGTGCTCAGAAGTAGAGGCAGGGCCATTGAGAGATGAGATAGATTTTGAGTTTTTGGGAAACAGAACAGGGCAGCCTTATCTTATTCAGACAAATGTGTATAACAATGGGAGTGGTGGACGTGAAATGAGGCATCAACTTTGGTTTGATCCTACTCTCGACTTTCATACTTATTCCATTCTTTGGAACTCTCATCAAATTGTATTTTTTGTGGATAAAGTACCAATAAGGGTATACAAGAACGCGAATCACACAAACAATTTCTTTCCAGCCCAGAGGCCGATGTACGTGTTTTCAAGCATATGGAATGCAGATAATTGGGCTACTAGAGGAGGCTTGGACAAGATAAACTGGGAAAATGCACCATTTGTAGCATCTTATAAGGATTTTACCATAGACGCTTGTCCATGGAAAAACCCTTACCCTGCTTGTGCTTCATCCACCACACAGCACTGGTGGGATCAGAATAATACTTGGCACCTATCAAGTAAAGAGAAGATTGATTATGCTTGGGTTCAGAGGAACTTTGTGGTTTATAATTATTGCCAGGATACTGTGAGGAACAAGTACAAGCCTCAAGAGTGTTGGTTAAATCCATTGGACTAA

>SlXTH24

ATGGCTTCTTCTTCTAAGTTAGTACTTGTAATGTGTTTTATGATTAGTGCTTTTGGCATTGCAATTGGGGCCAAGTTTGATCAAGAATTCGACATTACATGGGGTGATGGCAGAGCAAAAATACTTAACAATGGCGACCTCCTTACTCTCTCACTTGACAAAATCTCTGGCTCTGGTTTTCAACCCAAGAATGAATATCTGTTTGGTAAAATTGACATGCAGCTCAAACTTGTCCCAGGAAATTCTGCTGGCACTGTCACTGCTTACTATTTGTCATCACAAGGACCAACACATGATGAAATAGATTTTGAATTCTTGGGAAATTTAAGTGGTGATCCTTATACTCTCCATACTAATGTATTTAGTCAAGGCAAAGGAAACAGAGAACAACAATTTCATCTTTGGTTTGACCCTACTGCTGATTTCCACACGTATTCCATCACTTGGAATCCACAACGCATCATATTTTATGTGGACGGAACGCCAATTAGAGAATACAAGAATAGTGAATCGATTGGAGTTTCATATCCAAAGAACCAACCCATGAGGATATATTCGAGTCTTTGGAATGCAGATGATTGGGCTACAAGAGGAGGACTTGTTAAGACTGATTGGAGCCAAGCACCCTTTAGTGCTTCTTACAGAAACTTCAGTGCTAATGCTTGTATTCCCACTTCTTCATCTTCTTGCAGTTCCATTTCTGCAACTTCAACAAGCAATTCATGGTTGAATGAAGAGTTAGATAACACAAGCCAAGAGAGGCTCAAATGGGTGCAGAAGAATTACATGGTTTATGATTACTGCACTGATTCAAAGCGATTTCCACAGGGATTTCCAGCAGATTGTGTTCAGAATATCTGA

>SlXTH25

ATGGAATTTTTCCTTCATGATAGAAAATTTATATTATCAGCATTCTTGATTTTATGCATGATTATTGTTGTTTCATGTCGAGGTCCAGTGTACAAACCTCCAGAAATCGAAAAATTAACTGATCATTTTAGTCGATTATCGGTTAATCAGAGTTATAATGTGTTTTATGGAGGTTCTAATATTCATATTACAAATAATGGGTCAAGTGCTGAAATTATTTTAGATAAATCTTCAGGTTCTGGACTAATCTCTAAAGAGAAATATTACTATGGTTTCTTTAATGCTGCACTAAAATTGCCTGCTCATTTTACATCAGGAGTTGTTGTTGCCTTTTATATGTCAAACTCAGATGTGTTTCCACACAACCATGATGAAATAGACTTTGAATTGCTTGGACATGAGAAGAGAAGGGATTGGGTTTTACAGACAAATCTTTATGGAAATGGAAGTGTTCACACTGGAAGAGAAGAAAAATTCTACCTATGGTTTGATCCAACTTTGGATTTTCATGATTATACCATCCTTTGGAATAATCATCACATAGTATTTCTCGTGGACAATGTACCAGTAAGAGAAGTGGTTCACAACACAGCAATCTCTTCAGTTTACCCATCAAAGCCAATGTCAACTATATTGACAATATGGGATGGATCAGAATGGGCAACACATGGAGGAAAATACCCTGTAAACTATAATTATGCACCTTTTATAACAACAATCAAAGGTATTGAATTAGAAGGTTGTGTAAAACAACAACAAAATACATGTTCTAAGAGAAGTAGTACTTCAAGTTTGGACCCTGTTGATGGAGAAGGATTTATGAAGTTATCATCACAACAGATGAAAGGATTGGATTGGGCTAGGAGAAAACATATGTTTTACTCATACTGTCAAGATACTAAGAGATACAAAGTTCTACCACCAGAATGCACTTCTGAATAG

>SlXTH26

ATGGATCATCGAGTTCTTTCATTTGTATCAAAATCAATAACACCTTTCTCTCTCCTATTATTACTGTACATTTTTCCGGCGGCTGAGACGGCGGCGAACATGACGTATAAGGCGTTTAATCTGCCGACGATTACTTTCAAAGAAGGATATTCCCCTCTTTTTAGTGATTTCAATATTGAACGATCTCCTGATGATCGAAGCTTTCGTCTCCTCCTTAATAAATTCTCAGGATCGGGTGTTATTTCAACAGAATATTACAATTATGGATTTTTCAGCGCTAGTATAAAGTTACCGGCCATATATACGGCCGGCATCGTCGTCGCTTTCTATACATCAAATGCAGATACATTTGAGAAGAATCATGATGAGTTAGATATTGAGTTTTTGGGGAATGTGAACGGTCAACCATGGAGGTTTCAGACTAACATGTATGGAAATGGCAGTGTTAGCCGTGGTAGAGAAGAGAGGTATAGAATGTGGTTTGATCCAAGCAAGGACTTTCATCAGTACAGCATTCTTTGGACACCAAAAAACATCATATTTTACATTGATGAAACACCACTTAGAGAAATAAATCGCCATCCAGCAATGGGAGGTGACTTTCCAGCAAAACCAATGGCTTTATATGCCACAATTTGGGATGCATCTTCTTGGGCTACAAATGGTGGCAAAGCTAAAGTGGACTATAAATATGAACCTTTTGCAACAGAGCTTAAAGACTTAGTTCTTGAAGGATGCATAGTAGATCCATCTGAGCAAATTCCATCAACAAATTGCACTGACAGGAATGCTAAATTACTTGCTCAAGATTACTCTAACATCACGCCCGAAAGGCGAAACAACATGAAATTTTTTAGGGAAAGATACATGTACTATTCTTATTGTTATGATAACCTTAGGTACCCTGTGCCACCACCAGAATGTGTGATTGTTCAGTCCGAAAGAGATTTGTTTAGGGACAGTGGAAGGCTTAGGCAGAAGATGAAGTTTGGTGGGAGCCACAGCCATACCCAAAGCCACCGGAAACACCGCCCTGGACGGAGCTCTAGGCGGCGGAATAAGGTGGCTGGCGGTGCATCAAAATCTGGCCGACGAGGTTCTGCTGCTGCTGCAATGTGA

>SlXTH27

ATGGCTAATCTCCTCTTAATTGGAGTTGTAATTGCTATGCTATGCTCTGAAATTAAATGTTCATTTGAAGACAACTTTAGTAAAAGTGATTGTCCTGACTCTCACTTCAAGACTTCTGAAGATGGACAGATCTGGTACCTATCATTAGATAACAAAGCAGGTTGTGGATTTATGACCAGACAGAGATATAGATTTGGTTGGTTTAGCATGAAATTGAAATTGGTAGGAGGTGACTCAGCTGGTGTTGTTACAGCTTATTATATGTGCACAGAAGATGGGGCAGGGCCAACTAGAGATGAGCTAGACTTTGAGTTCTTGGGGAATAGGACAGGGGAACCATATCTTATTCAGACAAATGTGTATAAAAATGGTACTGGTGGGCGTGAGATGAGGCATGTTTTATGGTTTGACCCTACTCAAGACTTCCATACATATTCCATTCTTTGGAATTCTCATCAAATTGTATTTTTCGTAGATAAGGTTCCAATAAGAGTATACAGAAACGCGAATTACACGAACAATTTCTTCCCTAACGAGAAGCCAATGTACTTATTTTCGAGCATATGGAACGCGGATGATTGGGCTACTCGGGGCGGGTTAGAGAAAACAGACTGGAAAAATGCACCATTTGTATCAACATATATGGATTTCAATGTTGATGCTTGTCAATGGGAAGATCCTTTCCCTTCTTGTGTTTCAACAACTACTCAAAATTGGTGGGATCAATATAATTCTTGGCACCTTTCAAGTGATCAAAAATTGGACTATGCTTGGGTGCAAAGAAATTTAGTCACTTATGATTATTGCCAAGATATTGAGAGATATAAAGTAAAGCCTGAGGAATGTTGGGTAAGTCCATGGGATTAA

>SlXTH28

ATGTCATCCTTTATGATTGTCTTTTTGATCCTATCTATGCTACTAAACCCAGGGGTTGGTGTCAACTTCACTGATGTTTTCGAGTCCAGCTGGGCACCGGACCATATTGCTGTTGTAGGAGACGAAGTTACTCTCTCCCTTGACAGCGCTTCTGGCTGCGGATTTGAGTCGAGGTTCAAATATTTGTTCGGGAAAGCCAGTGCACAGATCAAACTAGTTGAAGGAGATTCAGCCGGAACAGTTATTGCATTTTATATGTCATCAGAAGGAGCTAATCACGACGAACTGGACTTTGAATTTCTTGGGAATGTTTCAGGGGAACCATACCTAGTACAAACAAATATCTACGTGAATGGCAGCGGAGATCGAGAGCAGAGGCACGGTCTGTGGTTCGATCCAACAACGGACTTCCACACTTACTCTTTCTTTTGGAATCATCATTCTATCATCTTTTCAGTTGATGATATTCCAATTAGAGTGTTCAAAAACAAGGAGAAAAAAGGTGTTCCATATCCGAAAAATCAAGGCATGGGAATCTATGGATCGTTGTGGAATGCAGATGACTGGGCTACACAAGGAGGGAGAGTGAAGACAAACTGGAGCCACTCTCCATTTGTTACGACATTTCGATCGTTCGAGATCGATGCTTGTGATTTGTGTGGTGAGGACACAATTGCTGCAGGTGCAAAATGTGGCAAGTTAGCTAAATTTTTGTGGGATAAACCATCCAAGAATGGGCTAGAAAAGAGCAAAAAACGCCAATTCAAAATGGTTCAAAACAAGTACTTGGTGTATGATTATTGTAAGGATACTGCAAGATTCAATCAAATGCCTAAAGAGTGCTTGTACTAG

>SlXTH29

ATGGCAAAAATCATACATTTTAATTCCTTGGTTTTGATGATTATTGCAACAATCACATTTCAATCATATTTAGCCAATGGATGGACATCAAGTAGCATGTATGTCAATTGGGGTGCTCATCATTGTAAACTTTTAGGGGATGATCTTCAACTTGTTCTTGATAAATCTGCAGGCTCTGGTGCTCAATCGAAAAATTCATTTCTCTTTGGTAGCTTTGAAATGCTTCTCAAGTTGGTACCTAACAACTCTGCTGGAACTGTCACAACATATTATTTATCTTCTACCGGTACCAAGCATGATGAAATCGATTTCGAGTTTCTAGGAAATATATCAGGACATCCTTATATTATACACACAAATATTTACACCCAAGGTGTTGGAAATAGAGAGCAACAATTCTATCCATGGTTTGATCCAACTGCTGCTTTTCACAATTACACCATTCATTGGAACCCTAACGCCGTTGTATGGTACATTGATAGTATTCCAATTAGGGTTTTTAGAAACTACCAATCCAAAGGCATTTCATTCCCAAACCAACAAGGGATGGGAGTCTACACTAGTCTATGGAATGCTGATGATTGGGCAACAAGAGGTGGTCTTGTTAAAATTGATTGGACAAATGCACCATTTATTGCAACTTATAGAAATTTTAGACCAAGAGCTTGCTATTGGAATGGACCAATGAGTATTTCCCAATGTGCAATTCCAACAAATTCCAATTGGTGGGCTTCACCTTCATACTATAAATTGAGTGCAAATAAAGTTGGTGAAATGATCTCAATTAGAAGCAAGAATATGATTTATGATTATTGCAAAGATGTGAAAAGATTCAAGGGAGTTATGCCTATTGAGTGCTCATTGCCCCAATACTAA

>SlXTH30

ATGGGTTTTCATCTAATAAGTCTAAGTGCTCTTTTATTATTAACTAGAGTTTTTGAAGGTCTAGCTTTACCATTTGATAAAAAATACAACATTTCTTGGGGGAACAACAATGTTAAGTTATTGAAAAATGGAGAAGAAATTCAGCTATCTCTTGATAAATTTTCTGGATGTGGGATTGAGTCCAAACAAAGTTATGGCTCTGGATCATTCAAAATGAGAATAAAGCTACCAAGCAAAGACTCAGCTGGAGTAGTGACAACATTTTATCTACATTCACATACAAGCCACCATGATGAATTGGATTTCGAGTTTTTAGGTAATAGAAAAGGGAAACCATACATATTGCAAACAAATGTATTTGCAAATGGTATTGGTGATAGAGAAGAAAGAATTCAACTTTGGTTTGATCCAACAACAAACTTTCATGAGTACTCAATCCTATGGAATTCACATCACATTGTTTTTTTTGTAGATGAAATACCAATTAGGGTTTACAAGAACAAATCATATAGAGGAATTGGATACCCTACACAACCAATGCAATCAGAAGCCACAATATGGAATGGAGAAAGTTGGGCAACAGAAAATGGAAGTCAAAAAATTAATTGGTCAAATTCTCCATTCATAGCTCAATTTCAAGGCTTTAACATTGAAGGTTGCCCTTCTAATTATCATAGTTTAAATTGCAATTCAACAAAGTGGTGGTGGAATTCTAAGAAATTATGGAAATTAACTCTTGATCAAGAAAAATCATATAAAGATATTAGAAGCAAAAATATGATTTATGATTATTGCAAAGATACCAATAGATTTCAAAACATTCCTTTAGAATGTTCAAGTGATTATTAA

>SlXTH31

ATGGCTTCTTTTGAGTTCATGAGTATAATTATTTGTATTTTGATGTATTTTGCTCTGTCACCAATTTATGCTATGGTTGATTTTAATCAATATTATAATCCCTTGTGGGGTCAAAATCATATAACTTATCTTAATCAAAGTACTGAAGTGCAGTTACTTTTGGATCAATCAGGAGGAGCTGGATTCAAATCGAAAACACAATATAACTCTGGATTATTTACATTAAGAATAAAGATGTCAGATAAAAAGACCGATGGAATGATCACAGCTTTCTACTTAATTTCAGATGATCAAGATGCACGGGTTAATCATGATGAAATAGACTTTGAATTTATAGGGACTCAAGGAAAATTACAGACAAATATATTTGCTAATGATATGGGTGGTAGAGAACAAGTTTTTCAACTTCCATTTGATCCTTCTCAGGATTTTCATACTTATCAAATTCTTTATACTCCACAAAGAATAGTGTTTTTTGTGGACAACATACCAATAAGGACATTTGAGAACAACACAAATAGAGGTATCAACTATCCAACAAAATCACTATGGTCAGAAGCAAGCCTATGGATTTCAGATGCTGTGGGTTGGGCTGGATCTGTTGAATGGGGCTATGCACCATTTATAGTTAGTTTTCAAGACTTCAACATTTCTGGTTGCCCTGCTGGTAGTGATTGCTTGCCATCCACAGATTTTAGCCCATGGACTAGGCACAAATTAGCCTCAAGAAGCTTGAATCTTATGAGGAATTTCAGAAAAAAATATATGACTTATGATTATTGTAGCTCTGAGGAAAATAAAAATAGGTACCCAGAATGTGCTTAA

>SlXTH32

ATGGCTTCCTTAGTTCTTTGTTTGGTCATTTTGGCATTTTGCTCTTTACATTATAGTTTGGCTTCTAATAATTTCAATCAAGATTTTGATGTTACATGGGGAGATGGTAGGGCAAAAGTTTTAAACAATGGCAAACTTCTTACTCTTTCCCTTGACAAAGTTTCTGGCTCCGGTGTTAAATCCAAGAAAGAATATTTGTTTGGAAGGATTGATATGCAACTTAAGCTCGTACGTGGAAATTCAGCTGGTACAGTTACTACATATTACTTATCATCACAAGGGTCAACACATGATGAGATAGATTTTGAATTCTTGGGAAACCTTAGTGGAGATCCTTATATTGTTCATACAAATGTGTATACTCAAGGCAAAGGTGATAAGGAACAACAATTCTACTTATGGTTTGATCCCACTGCTGATTTTCATACCTACTCCATTCTTTGGAATCCACAAACAATTATATTTTATGTGGATGGCACACCAATAAGAGTGTTCAAAAACATGGAGTCAAGTGGAGTACCTTACCCAAATAAACAACCTATGAGAGTCTATGCAAGTTTATGGAATGCAGATGATTGGGCCACAAGGGGTGGCCTTGTTAAAACAAATTGGTCCAATGCTCCATTCATAGCTTATTTTAGAAATTTCAAAGACAATAATGCTTGTATTTGGGAATTTGGAAAATCATCATGCACAAATTCAACAAAGTCATGGTTCTATCATGAACTTGATTCTACAAGCCAAGCTAGGTTACAATGGGTGCAAAAGAACTATATGGTTTATAATTATTGTAATGATATTAATAGGTTCCCTCGAGGCCTTCCTCTAGAGTGCGCTTTCAACTCTACGACTAATTAA

>SlXTH33

ATGGGCTTCAAATGGACGATGATGTTGGTGTTGTGTGTGTTAATAGGAGGATCAATGGGAGCTAAGCCCAATAAGCCAATTGATGTCCCATTTGGAAGAAATTATGAACCTAGTTGGGCTTTTGATCACATCAAATATTTGAATGGTGGCTCTGAGATCCAGCTCTCCCTCGATAACCGCACCGGCACTGGTTTTCAGTCAAAAGGATCTTACCTATTTGGGCACTTTTCTATGCACATAAAGATGGTTGCTGGTGATTCTGCAGGCACTGTCACTGCTTTCTATTTGTCTTCTCAAAATTCAGAACATGATGAAATAGACTTCGAGTTCTTGGGGAATAAAACAGGAGAACCATACATTTTACAAACAAATGTATACACAGGAGGGAAAGGTGACAAAGAGCAAAGGATTTACTTATGGTTTGATCCAACAAAAGATTATCACACTTACTCTGTCTTGTGGAATCTTCATCAAATTGTATTTTTTGTGGATGAGTATCCCATAAGAGTGTTCAAGAACAACAAGAACTTAGGTGTCAAATTCCCATTTGACCAATCAATGAAGATATACTCAAGTCTATGGGAAGCAGATGATTGGGCAACAAGAGGTGGACTTGAGAAAATTGATTGGTCAAATGCACCCTTTGTTGCCTCATACAAAGGCTTTCACATTGATGGTTGTGAATCTTCTGTCAATGCCAAATTTTGTGCCAATCAAGGCAAGAGTTGGTGGGATCAAAAGGAATTTCAAGATTTGGACAAAACTCAATGGAGGCTTTTGAGAAGAGTTAGGGACAAATATACAATTTATAACTATTGTACTGATAAAAAGAGGTTCTCTACAACGCCAATAGAGTGTAAGAGGAATAGAGATGTTCCAAGGAATTCAAGAAAGGAAAATTAA

>SlXTH34

ATGAATTATTTCTCTAGATTTATTTTCTTGGCCACTTATTTTATTTACTTATCTCATATTGCATTAGCTTCTATAGTTTCTACAGGAGATTACAATAAAGATTTCTACGTAACATATTCACCTAACCATATAAACACTTCTGCTGATGGCCGTACAAGAAGCTTGATATTTGACAAGGAATCTGGTACAGAGATTGCTTCAAAGGATATGTACTTATTTGGTCAATTTGACATGAAAATTAAGTTGATACCAGGAAATTCAGCAGGCACTGTTGTAGCATTTTATTTAGCTTCGGGTCAACCGAATCGCGATGAGATAGATTTTGAATTTCTGGGGAATGTAGATGGAAAACGTTATACTCTTCAAACAAATGTTTATGTTGATGGATTCGACGATAGAGAACAGAGAATCAATTTGTGGTTTGATCCAACACAAGACTACCATACTTATTCTATTCTATGGAACCTTCACCAAATTGTGTTCATGGTAGATTGGGTACCTATTAGAACATACAGAAACCATGCAGATAAAGGAGCTAAGTATCCACATTGGCAGCCAATGGAACTCAAAATGAGCCTATGGAATGGAGAAGATTGGGCAACAGATGGTGGAAAAACAAAAATTGATTGGTCAAAATCACCCTTTGTGGCCACATTGGGAAGTTATAAAATTGATGCTTGTGTTTGGAAAGGGAATGCAAGATTTTGCAGAGTAGAAAATGAAAATCATTGGTGGAATAAGGGGCAATCTAGTACTTTGACATGGACACAAAGAAGATTGTTTAAATGGGTTAGAAAGTATCATTTGACATATGATTATTGTATGGATAATAAAAGGTTTCAAAATAATATGCCCATAGAGTGCTCTCTACCAAAATATTAG

>SlXTH35

ATGGCTTCTTCTTCTAAATTAGTACTAGTAATGTGCTTTATGATTAGTGCTTTTGGCATTGCAATTGGGGCCAAGTTTGATCAAGAATTCGACATTACATGGGGTGATGGCAGAGCAAAAATACTTAACAATGGCGACCTCCTTACTCTCTCACTTGACAAAATCTCAGGCTCTGGTTTTCAATCCAAGAATGAATATCTGTTTGGTAAAATTGACATGCAGCTCAAACTTGTCCCAGGAAATTCTGCTGGCACTGTCACTGCTTACTATTTGTCATCACAAGGACCAACACATGATGAGATAGATTTTGAATTCTTGGGAAATTTAAGTGGTGATCCTTATACTCTCCATACTAATGTATTTAGTCAAGGCAAAGGAAACAGAGAACAACAATTTCATCTCTGGTTTGACCCTACTGCTGATTTCCACACGTATTCCATCACTTGGAATCCGCAACGCATCATATTTTATGTGGACGGAACGCCAATTAGAGAATACAAGAATAGTGAATCGATTGGAGTTTCATATCCAAAGAACCAACCCATGAGGATATATTCGAGTCTTTGGAATGCTGATGATTGGGCTACAAGAGGAGGCCTTGTTAAGACTGATTGGAGCCAAGCACCCTTTAGTGCTTCTTACAGAAACTTCAGCGCTAATGCTTGCATTCCCACTTCTTCATCTTCTTGCAGTTCCAATTCTGCAGCTTCAACTAGCAATTCATGGTTGAATGAAGAGTTAGATAACACAAGCCAAGAGAGGCTCAAATGGGTGCAGAAGAATTACATGGTTTATAATTACTGCACTGATTCAAAGCGATTTCCACAGGGATTTCCAGCAGACTGTGTTCAGAATAACTGA

>SlXTH36

ATGGTTAACTTTCAAGCAATTCTTGTTTTCATTAGTTTTTTCTTTTTTGTTAATCAATGTTTAAGTGCAAATGAGGTTCCATTTTACCAAAATTATTATCAAAAATATGGAGGTGACCATCTAACTGTTACTGACCAGGGAAAACAAGTTTGCCTAACTATAGACCAATATACAGGTTCTGGATTTATGTCTAACCAACATTTTGGTTCTGGAGATTTTAGCATCGACTTAAAAATACCAAACAAGAACAGTACAGGAGTAATAACAACATTCTACGTACGTACATTTTTTTTTTTATACAAAACGATATATGAATTACACAATCTTATCGATTCATCATCAAAATTGTACGCAATTTATGAATGTTATCGATTAATGAGTGAACATTTACAGTTAACATCACTGCCAATGAATGGAGATCCTGGAATGCATCATGATGAGATTGATTTTGAGTTCCTTGGAGGAGATGGTATATATACATTAAATACAAATATATTTGCAAATGATGGAGGAAGTAGAGAGCAACAATTCAATCTTGATTTTGATCCTACAGAAGATTTCCATACGTATCGAATTCTTTGGAATCAACATCATATCATATTTTACGCGGATAATGTTCCAATAAGAGTTTTCAAGAACAATACTAATTATGGAGTGAATTTTCCAACACACAAAATGCACATTGAAGCAACCATATGGAATGATACAAATTGGGTTGGAGAAGTAGATTGGAGCCAAGGACCATTCAAAGCTTATTATCGCAATTTTACGATTAATGGATGTCAATATCAAGAATCAAATCGTCAAGAATGCTATAATAACAACTATTATTGGAATACAATTACCAGTCTTAGTCCAAATGAAGTTCAGGAATTTGAAACTGTGAAGGCAGAACAAATGATTTTTAGTTATTGCATGAGGAACAATAGTAGAAATTTTCCAGAATGTATATTAAATTGA

>SlXTH37

ATGGCTTCTTCTTCTTCTAAGTTAGTACTTGTAATGTGTTTTATGATTAGTGCTTTTGGCATTGCAATTGGGGCCAAGTTTGATCAAGAATTCGACATTACATGGGGTGATGGCAGAGCAAAAATACTTAACAATGGCGACCTCCTTACTCTCTCACTTGACAAAATCTCAGGCTCTGGTTTTCAATCCAAGAATGAATATCTGTTTGGTAAAATTGACATGCAGCTCAAACTTGTCCCAAGAAATTCTGCTGGCACTGTCACTGCTTACTATTTGTCGTCACAAGGACCAACACATGATGAGATAGATTTTGAATTCTTGGGAAATTTAAGTGGTGATCCTTATACTCTCCATACTAATGTATTTAGTCAAGGCAAAGGAAACAGAGAACAACAATTTCATCTTTGGTTTGACCCTACTGCTGATTTCCACACTTATGCCATCACTTGGAATCCACAACGCATCATATTTTATGTGGACGGAACGCCAATTAGAGAATACAAGAATAGTGAATCGATTGGAGTTTCATATCCAAAGAACCAACCCATGAGGATATATTCGAGTCTTTGGAATGCTGATGATTGGGCTACAAGAGGAGGCCTTGTTAAGACTGATTGGAGCCAAGCACCCTTTAGTGCTTCTTACAGAAACTTCAGTGCTAATGCTTGTATTCCCACTTCTTCATCTTCTTGCAGTTCCATTTCTGCAACTTCAACAAGCAATTCATGGTTGAATGAAGAGTTAGATAACACAAGCCAAGAGAGGCTCAAATGGGTGCAGAAGAATTACATGGTTTATGATTACTGCACTGATTCAAAGCGATTTCCACAGGGATTTCCAGCAGATTGTGTTCAGAATATCTGA
