# Supplementary material for: Genome-wide characterization of the xyloglucan endotransglucosylase/hydrolase gene family in Solanum lycopersicum L. and gene expression analysis in response to arbuscular mycorrhizal symbiosis
Source: PeerJ. 2023 May 3;11:e15257. doi: 10.7717/peerj.15257 (PMC10163873; doi:10.7717/peerj.15257)
Supplement: Supplemental Information 3 [file peerj-11-15257-s003.docx]

**File S3.** Transcript sequences of tomato *XTHs.*

>SlXTH1

TTCCTCACCCCCATTGGGCCATATTCATCATTCTCTAAAAAAAGAAAAAAAGAAAAATACACAAACACTGGTCTCTGATTGGATTTGTTTTTCTCACCATGGGTATCATAAAAGGAGTTTTATTTAGTATTGTTTTGATTAATTTGTCACTTGTTGTATTTTGTGGGTATCCTAGAAGGCCAGTAGATGTGCCCTTTTGGAAAAACTATGAGCCAAGTTGGGCTAGTCACCATATTAAGTTCCTCAATGGTGGTACCACTACTGATCTTATTCTCGACAGATCTTCAGGAGCTGGATTTCAGTCAAAGAAATCATATCTGTTTGGGCATTTCAGTATGAAAATGAGGCTTGTTGGTGGAGACTCAGCTGGTGTTGTCACTGCATTTTACCTGTCATCGAATAATGCAGAGCACGATGAGATAGATTTTGAATTTTTGGGGAACAGAACTGGGCAGCCATACATATTGCAGACAAATGTATTCACAGGAGGAAAAGGAAACAGAGAACAGAGAATATATCTTTGGTTTGATCCAACCAAGGGCTACCATTCTTATTCTGTTCTTTGGAATACATACCTCATTGTGATCTTTGTGGACGACGTTCCAATTAGAGCATTCAAAAATTCGAAAGATCTTGGTGTGAAATTTCCATTCAATCAGCCCATGAAGATATACTCGAGTCTATGGGACGCAGATGATTGGGCCACAAGAGGTGGGCTTGAGAAAACCAATTGGGCCAACGCCCCATTCACCGCGTCATACACATCGTTCCACGTGGATGGATGTGAAGCTGCCACGCCACAAGAAGTCCAAGTTTGTAACACTAAAGGCATGAAATGGTGGGATCAAAAGGCCTTCCAAGATTTAGATGCATTACAGTATAGGAGACTTCGTTGGGTTCGTCAAAAATACACTGTTTATAACTATTGCACTGATAAAGCGAGGTACCCTGTTCCACCACCAGAGTGCACTAAGGACAGAGATATTTAAAATCATAATCAAAATTAAGAGGGACTTTATGAAGAAAAAAAACTTAATATGCTTTATGTGTGAGTATTTTAATGATCCTTAAAACAAAGTGCTTTTAATTGAGCTGTATTTCCCTAATTCTTTTTGAGTGTATCATTATTGGTGGAGTCATGAGGATATTATGTATCTCATGCCAGGCCTTTCATGTCTCTTGTGTTTCATCATCATAACCATAATCATGATTGATGTATTGTAATTTATCGAACTATTTGTTACTTATCTCTTAATTAAAAATATGAATTAATACTTTTTTTATTTAAGATAATTTGACTAAATCCTGAATCTCA

>SlXTH2

TTCCTCACCCCCATTGGGCCATATTCATCATTCTCTAAAAAAAGAAAAAAAGAAAAATACACAAACACTGGTCTCTGATTGGATTTGTTTTTCTCACCATGTCCATGCACTTCAAACACAAAAATATTTAAAACTTATAAAAAAAAACAATACTCAAACAACATGATCAAAACATCAAGTTGTATATTTACTTTCTTTCTTCTAATATGTTTCTTCGTAGTGGTGGCTTTTGGTGGTACTTTCGACCAAGAATTTGATGTTACATGGGGTTATGGGAGGGTGAAAATACTCGAAAACGGGCAACTTCTTACTCTTTCCCTCGATAGAAGTTCTGGCTCTGGATTTAAGTCTAAACAACAATATATGTTTGCAAAGATTGACATGAAGATCAAACTTGTCCCTGGTAATTCTGCTGGCACCGCTACTACATACTATTTATCTTCGGTGGGGTCAGCTCATGATGAGATTGACTTTGAGTTTCTTGGGAATGTAAGTGGAGAACCATATACTCTTCATACAAATGTGTATGCACAAGGCAAGGGAGATAGAGAGCAACAATTTCATCTTTGGTTTGACCCTACTAAGGATTTCCACACCTACTCTATTCTTTGGAATCCTCGAAATATCATATTTTTGGTAGATGGGACACCAATAAGACAATACAAGAATCTTGAAGCAACAAATGGAATACCTTACCCAAAGAACCAACCAATGTGGTTATACTCAAGTTTATGGAATGCTGAGGAATGGGCAACAAGAGGTGGCCTTGTAAGGACTGATTGGAGTAAAGCCCCTTTTATTGCTTCTTATAGAAACTTCAATGCCCAAACTTCCAAGAATCCCACAGCCAATTCTTGGTTAACTCAATCATTGGACAATGTTGGGTTAACAAGGATGAAATGGGTGCAAAAGAACTATATGATATACAATTATTGCACTGATACTAAACGTTTCCCTCAAGGGTTCCCTCATGAATGCACTCTAAATTAATAAGTTATTGTGTTAAAATATCCTCTATATTTATTTTTTCTTGCTAAATTGGAGTGATACGGTAAGAGTTGTTGTTGTGTGAATAAAGATATTATTATAGTTTCCAGTGGTGAAAGTCGAAACAACTTCTCGCAGAAATGGATATGATAAAGATGAGTGTGTATCAAAAGCTTAATGTATTAAACTGTTATTTTTAAAAAAGTTCGTTCTTTATACTATAAAGAAATGTATATTAGCCCACTTGAGAGTGATATTGTTTGATTGTACTTATGAAATAAGTTTGAAATATTGTTTAAGATCTTGACTTGTTCTTGTTATCTCAAAAGTATCGAGGGACACAAGTTATGCTTATCTATAATATATGGTACTCATAA

>SlXTH3

CTAATTCATCCAACAAACTTTGAAATATTAAGAATTAAACATGGCTTCTTCTTCTTCTAAATTAGTACTTGTAATGTGTTTTATGATTAGTGCTTTTGGCATTGCAATTGGGGCCAAGTTTGATCAAGAATTCGACATTACATGGGGTGATGGCAGAGCAAAAATACTTAACAATGGCGACCTCCTTACTCTCTCACTTGACAAAATCTCAGGCTCTGGTTTTCAATCCAAGAATGAATATCTCTTTGGTAAAATTGACATGCAGCTCAAACTTGTCCCAGGAAATTCTGCTGGCACTGTCACTGCTTACTATTTGTCATCACAAGGACCAACACATGATGAGATAGATTTTGAATTCTTGGGAAATTTAAGTGGTGATCCTTATACTCTCCATACTAATGTATTTAGTCAAGGCAAAGGAAACAGAGAACAACAATTTCATCTCTGGTTTGACCCTACTGCTGATTTCCACACGTATTCCATCACTTGGAATCCACAACGCATCATATTTTATGTGGACGGAACGCCAATTAGAGAATACAAGAATAGTGAATCGATTGGAGTTTCATATCCAAAGAACCAACCCATGAGGATATATTCGAGTCTTTGGAATGCTGATGATTGGGCTACAAGAGGAGGCCTTGTTAAGACTGATTGGAGCCAAGCACCCTTTAGTGCTTCTTACAGAAACTTCAGTGCTAATGCTTGCATTCCCACTTCTTCATCTTCTTGCAGTTCCAATTCTGCAGCTTCAACAAGCAATTCATGGTTGAATGAAGAGTTAGATAACACAAGCCAAGAGAGGCTCAAATGGGTGCAGAAGAATTACATGGTTTATGATTACTGCACTGATTCAAAGCGATTTCCACAGGGATTTCCAGCAGATTGTGTTCAGAATATCTGAGCATTAATAATGAAAAAATAGTGTATTACTTTAAAAACTATTGTATTGATTCTTTTATTGTTTTGTACCCATCAGTAGAAGATGCAATAATTATTGAGGATTAGAAACATCTTAGTTTTGTACTAAGTTATATAAACAATGAAATAGATACTTTTTCTTCTTCTAATT

>SlXTH4

ATGAAGGGAGTTTTAGTTGCTTTTGTTTTGATTAATTTGTCAATATTGGCAAGTTGTGGGGCTCCAAGGAAGGTAATTGATGTGCCTTTTTGGAACAACTATGAACCAAGTTGGTCTAGTCACCATATTAAGTACCTTAATGGTGGTACTACGGCTGAACTTCTTCTTGACAAATCCTCTGGAACTGGATTTCAATCAAAGAGATCGTATCTATTTGGTCATTTCAGCATGAAAATGAAGCTTGTTGGAGGAGATTCTGCTGGTGTTGTCACTGCTTTTTATTTATCATCGACTAATGCTGAACACGATGAGATAGATTTCGAATTCCTCGGGAATAGAACCGGTCAGCCATACATATTGCAGACTAATGTGTTCACAGGAGGCAAAGGAGACAGAGAACAGAGGATCTATCTTTGGTTTGATCCAACCAAGGACTTTCATTCATATTCTGTTCTTTGGAACACTTACCAAATTGCGATTTTTGTGGATGATGTCCCAATAAGAGTATTCAAGAATTCAAAAGACATAGGAGTGAAATTTCCGTTCAATCAGCCAATGAAGATCTACTCAAGCCTATGGAACGCGGATGATTGGGCTACAAGAGGAGGGTTAGAGAAAACTAATTGGTCTGGGGCGCCATTCATCGCTTCCTATACTTCATTCCACATTGATGGATGTGAGGCTGTCACACCACAAGAGGTACAAGTTTGTAACACCAATGGCATGAAATGGTGGGATCAAAAGGCTTTCCAAGATTTAGATGGCCCTGAATATAGAAAACTTCATAGGGTTAGACAAAATTTCACAATATATAACTATTGTACTGATAGAAAAAGGTACCCTACACTTCCTCTAGAGTGTACAAGGGATAGAGATCTTTAA

>SlXTH5

TCTCTCAAAGCACATATAAAAACACATAGTGTGGCCTCAGAAAAAAACAGAAACAAAAAAAAATGGATTTCATCAGAATGAAAATATGTCTTTCTGTCTTATTTTTTTTCCATGTTTGGTTTTGTAGAGCTTTTAATGATGTCTCAACAATTCCTTTTAACAAAGGATTCAGCCATCTCTTTGGTGATGGAAATATTCTTCATGCTAACGATGATAACAGCCTTCAACTTCATCTCAACCAAAACACAGGTTCAGGGTTCAAGTCTTCTGACCTTTACAACCATGGTTTCTTCAGTGCTAAAATTAAATTGCCATCAGATTATACTGCAGGAATAGTTGTTGCCTTCTATACGACGAATCAAGATGTATTTAAGAAGACACACGATGAACTAGATTTTGAATTTTTGGGAAATATAAAAGGAAAAGCATGGAGATTTCAAACAAATATGTATGGAAATGGAAGCACACATAGAGGAAGAGAAGAAAGATATACTCTATGGTTTGATCCTTCTAAAGAGTTCCATCGTTATAGTATTTTGTGGACCAACAAAAACATCATATTTTATATAGATGATGTTCCAATTAGAGAAATTGTAAGAAATGATGCAATGGGTGGAGACTACCCATCAAAGCCAATGGGCCTATATGCAACAATTTGGGATGCTTCAGATTGGGCTACTTCAGGTGGAAAATACAAAACAAATTACAAATATGCACCATTTATAGCTGAATTTACTGATTTAGTACTCAATGGATGTGCAATGGACCCATTGGAACAAGTTGTAAACCCTAGTCTTTGT

>SlXTH6

ATGGAATTCCTTCTTTATTTACTTTTATTTTTCTTACTCAATTCAAGATTAATCAATGCTCAAGGTCCCCCTTCACCTGGCTACTATCCTAGTTCTAGGGCACAATCTATAGGATTTAACCAAGGTTTTAGAAACCTTTGGGGTCCTCAACATCAATCATTGGACCAAAGTACCTTAACTATATGGCTTGATAAAAATTCAGGAGGAAGTGGTTTTAAATCTCTAAAGAATTATCGTTCTGGTTATTTTGGGAGTAGTATTAAGCTACAACCTGGTTTTACTGCTGGAATTATTACTTCTTTTTATCTTTCAAATAATCAAGATTATCCGGGGAACCATGATGAAATTGATATTGAATTTCTTGGAACAACACCAAACAAGCCATATACTTTACAAACAAATGTGTATATAAGAGGAAGTGGAGATGGAAATATTATTGGAAGAGAAATGAAATTTCATCTTTGGTTTGATCCAACAAAAGATTATCACAATTATGCAATCCTTTGGGACCCCAATGAGATCATATTTTTTGTCGATGATGTCCCTATTAGAAGATACCCTAAAAAAAATGATGCAACATTTCCACAAAGACCTATGTATGTCTATGGTTCAATTTGGGATGCATCATCTTGGGCAACGGAGGAAGGACGAATTAAAGCGGATTATCGATACCAACCTTTTATCGGAAAATATAGTAATAATTTCAAGGTTGAAGGTTGCGCGGCCTACGAGAGTCCCTCTTGTCGTCGAGCGCCTTCTAGCTCTCCTTCGGGGGGTGGAGGGTTGAGTCGACAACAGATAGAGGCTATGTTGTGGGTGCATAGGAACTATAAGGTGTACGATTATTGTAGGGATCCTAGGAGGGACCATACTCACACACCTGAGTGTTAGGATGAAAAAGATGTTGAACTCCAAAAGTCGGATTTTTCAAGGATTACATCGAGACAAAGAATGTCCATGAAAAGATTTAGGGCGAAATATATGTACTATTCTTACTGTTACGATTCATTGAGATACTCAGTGCCACCACCAGAGTGCGAGATTGATCCAGTTGAGCAACAACATTTTAAAGAGACTGGAAGGTTGAAGTTTATAAACAAGCACCACGGACATCGTCATCCTAAGAAAACAAAAAGTGAAGTTCTTGATGCTAGGAAGTATGGAAATGAAGATGAAGAGTGATTCAAAGTTTTGTTTACTAATAGTAATATTGTTGGTGTGTATATAGATAAAAGGAGGGAAAGAGTGAATTCGTTTATTTTTCTTGGATTATAGATTTTGTTATAGGATATATTGTCAGAGTGAAGGGTGAAAAATGATTATATATAAAGTGTGAACACTGATTGAGTAGTTATATTGTAATATAAATAAAGAGATTGAATTATGTATATTGAATTACTATTAAAGACTCCATACATTTCTTATTCTTCAAATTAAATAATCATATTTCATATAAT

>SlXTH7

ACACCACAACATCCAATTATATAATTAGAAAAAAAACAATGGCCACATTGACTTGCTCTTCCTTAAAAAATTCAGCTTTTGTTCTAATATTGGTATATGCCTTGACCTTTTCATTCTCACTAGTAAGTGCACGACCCGCCACTTTTTTACAGGATTTTAAAATCGCTTGGTCCGACTCTCACATCAAACAACTCGATGGCGGCAGGGGAATTCAACTTATTCTCGATCAAAACTCAGGATGTGGATTTGCTTCGAGAAGCAAATACCTGTTTGGACGTGTTAGCATGAAGATCAAGCTCGTTCCAGGTGACTCTGCAGGAACTGTTACCGCCTTTTACATGAATTCGGACACAGATAACGTAAGAGACGAACTTGACTTCGAATTCTTGGGAAACCGGACAGGGCAGCCGTACACTGTTCAAACGAATGTTTATGTCCACGGAAAAGGTGACAAGGAACAAAGGGTTAACCTTTGGTTCGATCCATCCGCTGATTTTCACACATACACCATTTTTTGGAACCATCATCAAGCCGTGTTCTCAGTGGATGGAATACCCATTAGAGTGTACAAGAACAACGAAGCAAAAGGAATCCCATTCCCCAAATTTCAACCCATGGGTGTCTACTCAACATTGTGGGAAGCTGACGACTGGGCTACAAGAGGTGGCTTAGAGAAAATAAATTGGAGCAAATCCCCATTTTACGCATACTACAAGGATTTTGACATTGAAGGATGTGCAATGCCAGGACCAGCAAATTGTGCCTCCAACCCAAGTAATTGGTGGGAAGGACCTTCTTATCAACAACTGAGCCCAGTACAAGCAAGGCAATATCGTTGGGTTCGAATGAATCACATGATCTATGATTATTGCACAGACAAATCGAGAAACCCCGTTCCCCCACCAGAATGTAGGGCCGGAATTTGAAACTTCTCCTTAATTGTGTATACATGGGGGGGGGGGGGGGGGATGAAATTTGTGCCCATTGATATGGACAGTCACGTTCATGTCTTATCAATGCTTGCTCCGTTGTTATATGTACTAAGATTATAGAAAGAGTGGCCTAAAGTCAAATGTCGTTTTATTGCTGTAATATATTGTTGTCTGTATATGTAAATTGTACGTACACTCTACGAGAGCCAATAATGTTTCTTCCTGTTTGATTG

>SlXTH8

ATGGTGAATTTTCTTCTGGAAATTTTTATATTTTGCTATGTTGTTGTATTAGTTTCTGGATTTTCAGAAAATCTCGAAACGTCGTCGTTTAATGAAGGATATTCACAACTTTTTGGTCATGATAATCTTATGGTCATTCAAGATGGAAAATCAGTTCATATTTCTCTAGATGAAAGAACAGGAGCTGGATTTGTGTCACAAGACTTGTACCTTCATGGCTTATTCAGTGCTTCTATTAAATTACCAGAAGATTACACTGCTGGAGTGGTGGTTGCATTTTATATGTCAAATGGAGACATGTTTGAGAAGAATCATGATGAAATTGACTTTGAGTTTTTGGGAAATATTAGAGCAAAAAATTGGAGGATTCAAACTAATATTTATGGAAATGGTAGCACAAATGTTGGTAGAGAAGAAAGATATGGACTTTGGTTTGATCCAACTGAAGATTTTCATACATATACAATTCTTTGGACTGACAGCCACATCATCTTTTATGTAGATAATGTACCTATAAGAGAGATCAAGAGAACACAAGCAATGAGTGAGGACTTCCCTTCTAAGCCAATGTCTTTATATGGTACAATATGGGATGGCTCTAGTTGGGCTACTAATGGGGGTAAATACAAAGTCAATTACAAATATGCCCCTTACGTCGCGAAGTTCTCCGATTTCGTCCTCCATGGATGTGGTGTTGATCCAATTGAATTGTCTCCCAAGTGTGATATAGTCCTGGATTCTGCATCCATCCCAACTAGAATATCCCCTGACCAAAGGCGAAAAATGGAGAGGTTTCGAAACAAGTACTTGCAATATTCATATTGCTATGACCGGACACGATACAATGTTCCTCAATCTGAATGTGTGATTGATCCTAAGGAAGCTAATCGCCTCCGAGGATTCGACCCTATGACCTTTGGTGGTGTCCCTCGTCATCAGAACAAACGACACCACCAAAGGCAATCGAGGAGGGAAGATACGTCCGCGAAATAA

>SlXTH9

ATGTCTTCTAAATTTTCATCAACATTGCTTCTTCTTATTTCAATACTAATGAGTATCCAATTACTAGCCTCAGCTGGTAATTTCTATAGAGATGTAGACATAACTTGGGGCGAAGGACGCGGTAAAATACAAGAAGGCGGTAGAGGCCTTGCCCTATCGCTTGATAAACTTTCTGGCTCTGGCTTTCAATCCAAAAATGAGTACCTTTTTGGAAGATTCGATATGCAACTTAAACTCGTCCCTAAAAACTCTGCTGGCACTGTAACAACTTTCTTCTTATCTTCACAAGGAGAAGGACATGATGAGATCGATTTCGAGTTCTTAGGCAATGTCTCTGGCCAGCCTTACACTATCCATACCAATGTATACACACAAGGAAAAGGAAACAAAGAACAACAATTTCATCTTTGGTTTGATCCAACTGCCGCGTTTCACACTTACACCATCGTCTGGAATCCTCATCGCATAGTGTTCTTAGTAGATAACAGTCCAATTAGAGTGTTCAACAACCATGAAAGCATGGGAATTCCATTTCCCAAGAGTCAAGCAATGAAAGTATACTGCAGTTTATGGAATATTGGGCACTTGCACCATTCACTGCTTATTACAGAAACATTAACATTGATGGTTGTGCAGTATCATCAGGTAACCTCTTCATGTAAGTCCATCGGTTCAATAAACAACGCGAAGCCATGGCAAACACATGAACTTGATGGTAAGGGACGGAATAGACTACGATGGGTGCAGACCAAACACATGGTTTACAATTACTGTGCTGATTCTAAGAGGTTTCCACAAGGCTTTTCTGCTGAATGCAAGAGTTCAAGATTTTAA

>SlXTH10

ACATTCACAATAACAAATTCTAATTTATCCCTTTAAATCACTACTCTAATTTTCTATAATGTTGCTGCAGCTTTCTCTTCTTACACTAGTCTTACTATCCCCTGTTTCCGCTGATAATTTCTACCAAGACGCGGCGGTCACGTTTGGTGACCAGCGCGCTCAGATACAAGATGGAGGGCGCCTTCTCACATTGTCACTTGATAAAATTTCAGGTTCCGGATTTCAGTCTAAGAATGAGTATTTATTCGGAAGGTTCGATATGCAGCTTAAACTCGTACCTGGAAATTCTGCTGGCACTGTCACCACATTCTATTTGTCTTCTCAAGGAGCAGGGCATGATGAAATTGATTTTGAGTTTCTAGGAAATTCATCAGGACTACCTTACACGGTTCATACCAATGTTTACTCTCAAGGAAAAGGCAATAAAGAACAACAATTTCGTCTCTGGTTTGATCCAACTTCGTCGTTCCACACTTACTCTATTGTTTGGAACTCTCAACGGATCATATTTTTGGTGGATAATATCCCAATTAGAGTGTTCAACAACCACGAAGCACTTGGTGTTGCATACCCAAAGAATCAAGCAATGAGAGTTTACGCGAGTCTATGGAATGCTGATGATTGGGCTACACAAGGAGGACGGGTGAAGACAGATTGGTCTATGGCTCCGTTTACAGCTTCTTACAGGAATTTCAATACAAATGCTTGTGTTTGGTCAGCTGCTACGTCTACTTCGTCTTGTGGAGGTTCTAAGACTGAGTCAGTAAACAATGATGAGACATGGCAAACGCAACAACTGAACGCTAATGGAAGAAATAGAATACGATGGGTTCAGCAGAAGTACATGATCTACAATTACTGTGCAGATGCTAATAGGTTCTCTCAAGGCTTTTCTCCTGA

>SlXTH11

ATGTTGCTGCAGCAGCTATCTGTTCTTGCTCTACTTCTCTTGCTATGTCCTGTTTGGGCTGACAATTTCTACCAAGATGCAACGGTTACCTTTGGTGATCAGCGAGCTCAGATACAAGATGGTGGGCGCCTTCTCGCCTTGTCCCTTGACAAAATTTCAGGTTCAGGATTTCAGTCTAAGAATGAATATTTATTTGGAAGGTTCGATATGCAGCTCAAACTAGTACCTGGAAATTCTGCTGGCACTGTCACTACCTTCTATTTGTCTTCTCAAGGAGCAGGGCACGACGAAATTGATTTTGAGTTTCTGGGAAATTCATCAGGCCAACCGTACACGGTTCATACTAATGTCTACTCTCAAGGAAAAGGCAACAAAGAACAACAGTTTCGCCTATGGTTTGATCCCACCTCGTCGTTCCACACCTACTCTATTGTTTGGAACTCTCAACGCATCATATTTTTGGTGGATAATATCCCAATAAGAGTATTCAACAACCACGAAAAGCTTGGTGTTGCATTCCCAAAGAACCAAGCAATGAGAGTTTATGCCAGTTTATGGAATGCTGATGACTGGGCAACACAAGGAGGGCGAGTGAAGACGGATTGGTCAATGGCTCCGTTTACAGCTTCTTACAGGAATTTCAACACAAATGCTTGTGTTTGGTCAGCTGCATCGTCTACTTCGTCCTGTGGAGGCTCTAAGACTGATTCAGTAAACAATGATCAGGCATGGCAAACTCAAGAACTGAACGGTAATGACAGAAATAGGCTTCGATGGGTTCAGCAGAAATACATGATCTACAATTACTGTGCAGATGCTAAAAGGTTCTCTCAAGGCCTTTCTCCTGAATGCAAACGTTCAAGGTTCTAAATGCAAGCGTTCAAGGTTCTAAGGCGGATATATATAGTATATGAATGTAAAATTATGTTTGTTTCACTTTTCTATTCTTTTAATTTTGATCAGGTAAAAAAAAGAACATAGTGTAATTATTTGTGTATGCAATATATTCTTTATTCTTTTTGTAATCATGAAATAGAAATAATAATGAATTGTTTTCCTGACAAGCAT

>SlXTH12

TCACACACAAGCAAAAAATCATATATTATTCAAAGTTCAAGTAAAAAATTTTAACTACAAATGGGGTCTTTTACCCATTATGGGTTCTTGATGTTAGCACTTTTATTTAGTTCTTGCATGGTTACTTATGGTGGAAATTTTTATCAAGAATTTGACTTCACTTGGGGTGGCAATAGAGCCAAGATTTTCAATGGAGGTCAACTTTTATCTTTATCTTTAGACAAAGTTTCTGGCTCTGGTTTTCAATCAAAAAAAGAACATCTCTTTGGAAGAATTGATATGCAAATCAAACTCGTTGCTGGAAACTCTGCTGGCACTGTCACAACATATTACTTATCTTCTCAAGGACCCACTCATGATGAAATTGACTTTGAGTTCTTGGGAAATGTTACTGGTGAACCTTATATTCTACACACAAATATTTATGCCCAAGGCAAGGGTAACAAAGAGCAACAATTTTACCTTTGGTTTGACCCTACAAAGAACTTCCACACCTACTCCATCATTTGGAAACCCCAACACATCATATTTTTGGTGGACAACACACCAATAAGAGTATACAAAAATGCTGAATCAGTTGGTGTACCATTTCCAAAGAATCAGCCCATGAGGATTTACTCAAGCCTTTGGAATGCTGATGATTGGGCCACAAGAGGAGGCCTAGTAAAAACTGATTGGGCCCAAGCCCCATTCACAGCCTACTATAGAAACTACATGGCCCAAAGCTTTAGCCCATCACAATTTTCTGATCAAAAATGGCAAAATCAAGAACTTGATTCTAATGGCAGAAGAAGACTTAGATGGGTTCAAAAGAATTTCATGATTTATAATTATTGTACTGATATTAAGAGGTTTCCTCAAGGTTTTCCTCCAGAATGTAGAAGATTTTGAGAGGGTTATGTAGTTTTTTTTTTGTTTTTTTTTTTTTGGGTGAAATTCTTTCATGTGTTTGTGGTTTTATTTTGATAGATTGTTAGCCAACTAAAATAAATTAATATGTTTTTTCTTTGTTTTATTTTGTATGTTATTTGAAGGTAGCTAGTAGTTTATTTTGTATCTATTTTATTTGATATCCTTTTTAGTA

>SlXTH13

ATGGCATTATTTTCATCAAGAAATTCATCAAGATCTAGGTCCTCTCTTCCATATTTGGTGTTTCTCTTAATTGCTGCCTTTTTTGTCTTCAAGGTAGATATACTCATATCTCAGTCTTTTAGTTCAGCCCGTCGCAACCTGGAAAAAACTCCTAATCGTATCGTTGTGAACCCCCAAAAATCATCGGAAGAACGTGTTGTTGACAGCCTCCCTGTAGTTTTAGTAAATGGTACATTTGACCAGCATATTATGATATCATGGGGAGATGACAGAGGAAAAATACTTGAAAATGGAGAGCTTTTAACACTCTCCTTAGACAAGAAGTCTGGATCAGGCTTTCAGTCTAAAAAAGAGTACCTCTTTGCTAAAATTGATATGCAAATTAAGCTCGTCCCTGGAAATTCTGCTGGCACTGTTACTACGTTTTACCTATCATCACAAGGGAACAAGCATGATGAAATAGATTTTGAATTCTTGGGGAATTCAACAGGGAACCCTTATACTCTTCATACAAATGTTTTTAGTTTAGGCAAAGGCAATAGGGAACAACAATTCTTCTTGTGGTTTGATCCAACTGCAGATTATCACACATATTCAATCCTATGGAATTCTAAATGTATTATATTCTATGTTGATGATATACCAATTAGAGAATACAAAAATCCAGAGAGACTTGGTCTTTCATATTTAAAATACCAACCAATGAGACTATACTCAAGTCTATGGAACGCAGATGATTGGGCTACACAAGGTGGTCGTATCAAAACCAATTGGGAACTAGCACCTTTTGTAGCGTCCTACAAAAATTTCACATATGAAGCTTGTATTTATTCAAGATTAACTAGTTCATCTTCATGTGATATCGACTCTCCAACTCCTATCAACAACGCTTGGTTAACATATGAGTTAGATCGAACAAGTCGTGTTAGAATGAAAGCTTTGCAGAAAAAACATATGATTTATGATTATTGCAACGATAAATGGAGATTTCCTAAAGGTCCTGCTCCTGAATGCAAGCTTCTTCAATAA

>SlXTH14

TCCATATACACCACCTTCCTTTCAACACTTTTCCATTCATATTTTTATTTTCTTTCTTCATTTCCAAAAAAAAATTTTAAAAAAATGTCAACAATTTTTTTCCTTCCTATTTTTCTTTGTTTTATTTTTCTCCATTCAACTAATGCTAATTATTGGCCAATTTCACCTGGATATTATCCAAGTACAAAATTTAAATCCATGAGTTTTTATCAAGGATTTAAGAATCTCTGGGGTCCTAATCACCAGAGTGTAGATAACAATGGCATTAATATTTGGCTTGATAGAAATTCAGGCAGTGGATTCAAGTCGGTTAAACCGTTTCGATCCGGGTATTTTGGGGCTTCTATTAAACTCCAACCCGGTTATACGGCCGGAGTCATCACCGCTTTCTACCTTTCAAATAATGAAGCACATCCAGGGTTCCATGATGAAGTGGACATAGAATTTCTTGGAACAACATTTGGAAAACCATATACATTACAAACAAATGTATATATTAGAGGAAGTGGTGATGGAAAAATTATAGGAAGAGAAATGAAATTTCATTTGTGGTTTGATCCCACAAAAAATTTTCATCACTATGCTATTTTGTGGAGTCCGAGAGAAATCATATTTTTGGTGGATGATGTACCAATAAGGAGGTATGCAAGGAGAAGTGATGCAACATTTCCATTGAGGCCAATGTGGTTATATGGATCCATATGGGATGCATCTTCATGGGCTACTGAGAATGGAAAATACAAAGCTGATTATAACTACCAACCATTCTATGGAAAATTCACGAATTTCAAGGCGAGTGGTTGCACCGCCTACTCGTCTCGATGGTGTCGACCTGTGTCTGCCTCGCCCTATAGATCCGGTGGCCTTAGCAGGCAACAACGTCAGGCCATGAATTGGGTTCGAAGTCACTATATGGTGTATGATTATTGCAGGGACTTTAAAAGAGATCACTCCCTTACACCAGAATGTTGGCGCAAGTGAAACAATTTTCTTATTTGTATCTTATATTCATCGGATGAACATAACTAAGCGATTTGTTGAAGCTGTGGGATGTATCGATGGTACACGTGTCAATCTTTTATAGGATGAATTTCCTATTTTGTGTGAACTACTTTTTTTTTCTTTTTTGTTATTGGGTATGGGGTTTGGGGTGTGGGGTGTGGGGTCTTGTGATAGGTTCCACTTGTCAATATCAATTGTAAGAGTAGTTGAGTGTCCAAGCTTATAGATTCCAAATAAAAAATCAATAATACAT

>SlXTH15

TTAAGAAGCTTCACTTCTCAAGTTCCTCTTGTGACGTTTTTAATTCCATTTATTTTTTTTAAAATTTTGGGGAGAAAATGGCTTCTCCTATAGCTTATTTTCTTGTACTTAGTGCAATAATTGTTGTACTTTTTTCATCAACACAAGCTGAAGTACAAGGTTCATTTGATGACAATTTTAGTAAAAGTTGTCCAGAAACTCATTTCAAGACTTCTGAAGATGGACAGATCTGGTATTTATCATTGGACAAAAAAGCAGGATGTGGATTTATGACCAAACAGAAATATAGATTTGGGTGGTTTAGCATGAAGTTGAAATTGGTGGGAGGTGACTCTGCTGGTGTTGTGACAGCTTACTATATGTGCACAGAAGATGGAGCAGGACCAACAAGAGATGAATTAGATTTTGAGTTCTTGGGGAATAGGACAGGTGAACCTTATCTTATTCAAACAAATGTGTACAAGAATGGAACTGGTAATCGTGAGATGAGACATGTTTTATGGTTTGACCCCACTGAGGATTTTCACACCTACTCAGTTCTTTGGAATACTCACCAAATTGTGTTTTTCGTGGATAAGGTACCAATAAGAGTGTACAAAAACGCGAATTACACAAACAATTTCTTCCCAAATGAGAAGCCAATGTACTTATTTTCAAGTATATGGAATGCAGATGATTGGGCTACAAGAGGTGGTTTAGAGAAAACAAATTGGAAAAATCAACCATTTGTTTCATCATACAAAGATTTTAGTGTGGATGGTTGTCAATGGGAAGATCCATATCCATCTTGTGTTTCAACAACAACACAAAATTGGTGGGATCAATATGATTCATGGCATTTATCAAGTGATCAAAAATTGGATTATGCTTGGGTACAAAGAAATCTTGTCATTTATGATTATTGTCAAGATACTGAAAGATTTCCAAAAAAACCTGAGGAGTGTTGGTTAAATCCATGGGAATAATTAAATAATTCAACTTAGGTTAAAAAAAATATATATTATGAGAGGGATTAAGGAGTATTTTGATTATTTTATTTTTGATGTTGAGTTATTACTCGAGTATATATATATATATATATATAT

>SlXTH16

CATCTTAAACAAAACATATATTCATAAAATCTCTGAAAATTTAAAAAAAATAAAATGGTGAGTTTTAATTGGGTTTTTTCGAGCTTTGTGATGCTGTTTATGGTGGGTTTGGTTAGCTCTGCAAAATTTGAGGAGCTTTATCAACCCAGTTGGGCTTTTGACCATTTGACAACTGAAGGAGAGATTCTTAGAATGAAATTGGATCATCTTTCTGGTACTGGGTTTCAATCTAAGAGCAAATATATGTTTGGGAAAGTTACTGTTCAGATTAAGCTTGTTGAAGGTGACTCTGCTGGAACTGTCACTGCATTCTATATGTCATCAGATGGACCAACCCACAATGAGTTTGATTTTGAATTTTTAGGCAATACAACTGGTGAACCATATACAGTACAAACAAATGTGTATGTCAATGGTGTTGGTAACAGAGAACAGAGATTGAAGCTTTGGTTCGACCCATCGAAGGATTTTCACTCTTATTCCATCATGTGGAATCAACGTCAAGTTGTATTCTTGGTAGATGAAACCCCTGTTCGAGTGCATTCGAATTTGGAGCATAGAGGAATCCCATACCCCAAGGATCAACCAATGGGTGTGTATAGTTCGATTTGGAATGCAGATGATTGGGCTACACAAGGCGGGCTCGTTAAGACTGATTGGTCACACGCGCCCTTCGTAGCATCCTATAAGGGATTTGAGATTAACGGTTGTGAGTGCCCGGCAACTGTTGCAGCTGCTGAGAATACTCGGCGTTGCAGCAGTAATGGGCAGAAGAAGTACTGGTGGGATGAACCTGTTATGTCCGAGTTGAATTTGCACCAGAGTCACCAGCTGATATGGGTCAGGGCGAACCATATGGTTTACGATTATTGCACGGATAGCGCTAGGTTCCCTGTTGCCCCTGTTGAGTGCCAGCACCACCAGCACAAGACGAATCATAACTAGGTGTGGAGGAAAATTGGAGTTCAGTCTTGCATTGTATAAAAAGATTTAGATAAAAAAGAAAAAAATGACATGAACTCCATGTTACATATTTTTTGTGCCACTAGTATGTAAAAATTTTCATTGTCTCTTTGTTTTAATGTTTATGTTAGTTTAATGAAAATTTTTTTTCTTCGTTGGACC

>SlXTH17

GATCATCATTATTTCTCTTCAACAATAATATTTCAGCTAATTTGAAAAAAAATGGCTAATTCTCATTTACTTTTAATTTCCATTGTATTAATGGGCAATTTAGTGGCTGTATTAGCAGCTGGTAATTTTAATGACCTTACAGAAATCACTTGGGGTGATGGACGTGGTAAAATATTAGATGGAGGTAAAGGTCTCTCTTTGTCACTTGATAATTATTCCGGGTCGGGTTTTCAATCGAAAAATGAATATCTCTACGGAAGATTCGACATGCAACTCAAACTCGTCCCTAAAAACTCTGCTGGCACTGTCACCACGTTCTTTCTATCGTCACAAGGAGAAGGACACGATGAGATCGATTTCGAGTTCTTGGGAAATGTGACTGGTGAGCCTTATACGGTACACACCAATGTTTATTCTCAAGGAAAAGGAAACAAAGAACAACAATTTCACCTTTGGTTCGATCCAACTGCAGCATTTCACACTTACACCATTGTTTGGAACGCTAACCGCATAGTGTTTTTGGTGGATCAGATTCCAATTAGAGTATACAACAACCATGAGAGCATTGGAATTGCATACCCCAAAAGTCAACCAATGAAAGTCTATTGTAGTTTATGGAATGCAGATGAATGGGCTACACAAGGTGGTAGAGTCAAAACTGATTGGTCACAAGCACCATTTACTGCCTATTATAGGAACATTAACATTGATGGCTGCGTCGTTAAATCAGGCGCTTCCTCATGTGCCTCACGGTCTACTGAATCCACGAACAGTGCTAAGTCGTGGGAGACACATGAGCTTGATGCTAAGGGTCGGAACAGGGTCCGATGGGTACAGAGCAAACATATGGTTTATAATTATTGCGCGGATTCTAAGAGGTTTCCTCAAGGATATTCACAAGAGTGTAAACGATCAAGGTTTTAAAAAATCGAATTTAATTTAATTACGTATGGTTCGAAACTCGATGAATATGTAAGCTGGTTATGCGATTGATTGTTTTTGTGTGTGAAATTCTTTATTGTTTTGTTTTGTTGTATTACCAATAAAATAAAAGGTTGCTTATTTATTTTTTTTGATTAAATGATATATGTACTATTCAAGTTTTATA

>SlXTH18

ATGGCAAAACTCATAGATTTTAATTCTTTGGTTTTGATGATTATTGCAATAATTGCATTATTTCATTCATATGTAGTCATTGGGATGACATCAAGTAGCATGTATGTTAATTGGGGTGCTCATCATTGTAAACTTCTAGGGGATGATCTTCAACTTGTTCTTGATAAATCTGCAGGCTCTGGTGCTCAATCAAAAAGATCATTTCTTTTTGGTAGCTTTGAAATGCTTATCAAGTTAGTACCCAATAATTCTGCTGGAACTGTCACAACATACTATCTATCTTCTACTGGTACCAAGCATGATGAAATCGATTTCGAGTTTTTAGGAAATATATCAGGACAACCTTATATTATACACACAAATATTTACACCCAAGGTGTTGGAAATAGAGAGCAACAATTTTACCCTTGGTTTGATCCAACTGCTGATTTTCACAACTATACTATTCATTGGAACCCTAACGCCGTTGTATGGTACATTGATAGTATTCCAATTAGGGTTTTTAGAAACTACCAATCCAAAGGCATTCCATTCCCAAACAAACAAGGAATGAGAGTCTACACTAGTCTATGGAATGCAGATGATTGGGCAACAAGAGGTGGTCTTGTTAAAATTGATTGGACAAATGCACCATTTATTGCAACTTATAGAAAATTTAGACCAAGAGCTTGTTATTGGAATGGACCAATGAGTATTTCCCAATGTTCAATTCCTACAAAAACCAATTGGTGGAGTTCACCTACATACAATAAATTGAGTGCAAATAAACTTGGTCAAATGAACTCAATGAGGAGTAAGTATATGATCTATGATTATTGCAAAGATGTGAAAAGATTCAAAGGAGTTATACCTATTGAGTGCTCATTGCCACAATACTAG

>SlXTH19

ATGCAATTCAAAAACACATACACCATGAAGACTACTTTCTTACTTTTCTTGATTCTAAGTTTCTTCTTCTCTGCTTTGGCTGGAAATTTTAACCAAGATTTTGATATTACATGGGGTGATGACCGCGCCAAAATACTCGAAAACGGACAACTTATGACCCTTTCCCTCGATAAAGTCTCTGGCTCTGGTTTTCGATCCAAAAACCAGTATTTGTTTGGAAAGATTGATTTGAAAATCAAACTTGTGCCTGGTAACTCTGCTGGCACCGTTACAACATACTATCTATCTTCAATAGGATCAAGTCATGATGAGATTGACTTTGAGTTTCTTGGGAATTTGAGTGGTGACCCATATATTCTTCATACAAATGTATTCACACAAGGAAAGGGAGATAGAGAGCAACAATTTTATCTTTGGTTTGATCCCACTAAGGACTTTCATACATATTCTATTCTTTGGAATCCTCAAAGCATCATATTTTCAGTAGATGGGACACCAATTAGGCAATTCAAGAATCTAGAATCAAGTGGTATCCCCTATCCAAAGTCACAACCAATGTGGATATACTCAAGTTTATGGAATGCAGATGATTGGGCTACAAGAGGTGGACTTGTCAAAATTGATTGGACTAAAGCCCCATTTATTGCTTCATATACAAATTTCAATGCACAAGCTTGTGTATGGTCTTCAACTTCAACTTCTTCTTCTTGCAATTCTACTACACAAGATTCTTGGCTAAGTGAAAACTTGGATATAACAGGCAAATCAAGGATTAAATGGGTGCAAAATAATTACATGATTTATAATTATTGTAATGATATTAAACGTTTTCCTCAAGGGTTTCCTCTTGAGTGTTCTCTCAATTAGTGCATGTAAATTAAGTTAATTATTGCATTGTAATTAAAATAATGTACTTAGGATTAGTAGAAATGTTTTGTTTGATTTGAGGAATAAGATGGAGAAATTCCATTTATACCTATTTGATAATTTTTAAT

>SlXTH20

CAATATATTCTCAAATTTTTTATAAATCCACTAAAAATAACAATGCCTTTTCTATTTTCCTTTAATATTAGACTTATTTTAGTTTTAGTATTTATAAGTTGTATGGTTGTTAAATATTGTGCTAGTAATGATCTTAATCAAGATTTTGATATTACATGGGGAAATGAAAGGGGGAAAATACTAAATAATGGTGAAATACTTACTCTTACACTTGATAATATTTCAGGTTCTGGATTTCAATCAAAGAAGGAATATTTATTTGGGAAAATTGATATGCAAATAAAATTAGTTCAAGGGAACTCTGCTGGCACTGTCACCGCATACTATTTGTCATCACAAGGATCAAGTCATGATGAGATAGATTTTGAGTTTCTTGGTAATTTAAGTGGAGAGCCATATACACTACATACAAATGTGTATACACAAGGCAAAGGTGATAGAGAGCAACAATTTCACTTGTGGTTTGATCCTGCTAATGATTTTCATACTTATTCTATCCTTTGGAATCCACAAACTATTGTATTTTCAGTGGACAATGTACCAATAAGGGAGTTTAAAAATATGGAAAACATTGGAGTTGCATTTCCAAAATCTCAATCAATGAAACTTTATTCAAGTTTATGGAATGCTGATGAATGGGCCACAAGAGGTGGGCTTATCAAGACTGATTGGGCCCAAGCCCCATTTACAGCCTCTTATAGAAATTTCAATGCCAATATTTGCAATAATAATAATAATAATAATGATTCTTGCAAATATTTGGTAGAAAATTTGGATCCTGTGAATGAAGAAAAATTGAGAAGGGTGCAACAAAAATACATGATATATAATTATTGTACTGATAATAAGAGATTTCCTCAAGGTTTTCCTCTAGAGTGTAGTGTTAGTTAATTAATTAATTAAATTTAATTAATGTAGGTAGAAAAGTTGGTATAATAATATTAATAGCCTCCTCTAGTTTTGTCTCTTTCAAGTTTATTATATATTGTGGCCTAAGTGATAAGGGAGGACTACTATTGTAATATTTATATGTTTATGTTTTTAATAATGGAAGTTGAAAGTGTTTATGTACTT

>SlXTH21

TGGTGTCTTTTTTTTTTCTCAATCAAAATAAGTCCATTTTTCTCTTCTTCTTCTTCCTCTCTTCTGTGGAGACTGATGAGACTTCATTTTTTCCCCCACTTCTCATGCATTACAACTCTATAGAAAAATACAACAAAAAAGAATTAATCTTTGGGTGTTCATAACTGATAAAAGGTGTAAAGGAAAAAGAAAATACCCAAAAAATAGAAAATAAAAATGGTGAACTATTATATGTTCTTTTTCATATTTTTGTCTTGTATTCTTGTTTTGGTTTCTGGGTTTTCAAGAAATCTGCCAATTTTAGCTTTTGATGAAGGTTACTCTCATTTATTTGGTGATAATAATCTTATGATCCTTAAAGATGGAAAATCAGTTCATATTTCTCTTGACAAAAGAACAGGGGCTGGATTTGTGTCTCAAGACCTTTATTTTCATGGATTTTTTAGTGCTTCTATTAAGTTACCTGCAGATTATACAGCTGGTGTTGTTGTTGCATTTTATATGTCTAATGGGGATATGTTTGAGAAGAACCATGATGAAATTGATTTTGAGTTTTTGGGAAATATTAGAGGCAAAGACTGGAGAATTCAGACTAATATTTATGGGAATGGTAGCACAAATGTTGGCAGAGAAGAAAGATATGGACTTTGGTTTGATCCTTCTGAAGATTTTCATCAATACAGTATCCTTTGGACTGAGAATTTGATCATCTTTTATGTAGATAATGTCCCCATAAGAGAGATCAAGAGGACAAAAGCCATGGGTGGGGACTTCCCATCTAAGCCAATGTCCTTGATAGCTACAATATGGGATGGTTCTAATTGGGCTACAAATGGTGGAAAATACAAAGTCAATTACAAATACGCCCCGTATATCGCTGAGTTCTCCGATTTCATCCTCCACGGATGCGCGGTTGATCCAATCGAACTGTCATCCAAATGTGACAACACTACGCCAAAAACTCCAACGATCCCTACCGATATCACCCTTGACCAAAGACGAAAGATGGAGAACTTCAGAAAGAAGCAAATGCAATATTCATACTGCTATGACAAGACCAGGTACAAGGTCCCTCCTCCCGAGTGCGTGATCGACCCTAAGGAAGCCGAACGACTCCGAGCCTTTGACCCCGTTACATTTGGAGGATCCCACCACCATCATGGGAGACGACATCACCGGAGCAGACCAAAGTTGAAGGGTGATGATGATGTATCCTTTATGTAAAAGAAAAAAGACCCAATCCCATAAGTTTTTCTCTATATAGTCACATGATGATATGATCATTTGAGGTTTTGTTATTTTCACATGTGAATAGAGGATATATTGTTGGGGTATGTATATATGTCATACCTATATAGGGGTCCCAAGCCATGTTCTAGTTGATGTATTTTGTTGTTTGGGCCTTTAATTTATAAATTCAACTTGTTGGTGATTTTGTGTACTTTTTTTGTGACACATTAATAAATGCATGAATAGTACAAAGATTATTTATTATAC

>SlXTH22

ATGGGCAGCTCTCTAGTTCTTTCATTGGCTAATTTGTTGATTATTTCAACAATTGTGTCATTTGGTTCTTTAGTTATGGTTAATGGTATTTTCTCAGATAATATGTACATTAATTGGGGTTCTCATCATTCTTGGATGCAAGGAGATGATCTTCAACTTGTCCTTGATCAATCTTCTGGTTCAGGTGTACAATCAAAAGGAACATTTCTATTTGGAAGTATAGAAATGCAAATTAAATTGGTACCTGGAAATTCTGCTGGAACAGTCACTGCATACTATTTATCCTCAACTGGTGACAAGCATGATGAAATTGACTTTGAGTTTCTAGGAAATGTATCAGGACAACCATATATTATACACACAAATATATTTACTCAAGGTGCTGGAGGCAGGGAACAACAATTTTATCCATGGTTTGATCCAACTGCTGATTATCATAATTATACCATTCATTGGAACCCTAATGCAGTCGTATGGTACGTTGACGATATACCAATTAGAGTCTATAAAAACTATCAGAGTCAAGATATTCCCTATCCGAACGCGCAAGCAATGGGGGTTTACTCTAGCCTTTGGAATGCTGATAGTTGGGCAACTAGAGGTGGTCTTGTCAAATGTGACTGGACCAATGCACCATTTATAGCCAAGTATCGAAATTTCGCCCCACGGGCCTGTGCCTGGAACGGACCTATTAGCATTAGTCAATGTGCAACTCAAACTCCAAGTAACTGGTATACTGCTCCTGAGTATAATCAATTGAGTTACGCGAAACAAGGTCAAATGGAATGGGTTAGGAGCAATTACATGATTTATGATTATTGTAAAGATACGAAGCGATTTAACGGACAATTTCCTGGAGAGTGTTTTAAACCTCAATTTTAA

>SlXTH23

GGATCCAGCCTATCTTCATTCAAAAGGGAAAAAAAAAACCACCATGGAGAGCAATGCTTCTTCAATGGCTCGTGTTCTTTTGATTTTATCAGTAATTTTTACCCTTTTTTCATCATCAAATGGTGTAGTTGGAGGTGCATTTGAAGAAAATTTCAGTAAAAGTTGTCCTGGTACACATTTCAAGACTTCTAAAGATGGACAGATCTGGTATCTTACCTTAGACCAAGTATCAGATTGTGGGTTCATAACAAAACAGAGCTATAGATTTGGTTGGTATAGCACAAAGTTGAAATTAGTAGGAGGTGACTCTGCTGGTGTTGTGACAGCATTTTATATGTGCTCAGAAGTAGAGGCAGGGCCATTGAGAGATGAGATAGATTTTGAGTTTTTGGGAAACAGAACAGGGCAGCCTTATCTTATTCAGACAAATGTGTATAACAATGGGAGTGGTGGACGTGAAATGAGGCATCAACTTTGGTTTGATCCTACTCTCGACTTTCATACTTATTCCATTCTTTGGAACTCTCATCAAATTGTATTTTTTGTGGATAAAGTACCAATAAGGGTATACAAGAACGCGAATCACACAAACAATTTCTTTCCAGCCCAGAGGCCGATGTACGTGTTTTCAAGCATATGGAATGCAGATAATTGGGCTACTAGAGGAGGCTTGGACAAGATAAACTGGGAAAATGCACCATTTGTAGCATCTTATAAGGATTTTACCATAGACGCTTGTCCATGGAAAAACCCTTACCCTGCTTGTGCTTCATCCACCACACAGCACTGGTGGGATCAGAATAATACTTGGCACCTATCAAGTAAAGAGAAGATTGATTATGCTTGGGTTCAGAGGAACTTTGTGGTTTATAATTATTGCCAGGATACTGTGAGGAACAAGTACAAGCCTCAAGAGTGTTGGTTAAATCCATTGGACTAATATTAAACATTAGAAAAAATTCATTGGTGAGAGAAAAGTCATAGTAGTATTTTGATCTTGATTTTGAGTGTATATATTGGACGATTGAGCCATTGTAGATCCATCGATTGGTATATTGTAAACCATCGGCATTACTACTATTGGAAAGTGAAAAGAGTGGTTCACTATATTTACAAATGTTATAACGATATTCTTAATGTAATCAGTATGTTTTATTCAAGGAAGTTAAGATTTCAATT

>SlXTH24

CAAATTCATCCAACAAACTTTGAAATCTTAACAGTTAAACATGGCTTCTTCTTCTAAGTTAGTACTTGTAATGTGTTTTATGATTAGTGCTTTTGGCATTGCAATTGGGGCCAAGTTTGATCAAGAATTCGACATTACATGGGGTGATGGCAGAGCAAAAATACTTAACAATGGCGACCTCCTTACTCTCTCACTTGACAAAATCTCTGGCTCTGGTTTTCAACCCAAGAATGAATATCTGTTTGGTAAAATTGACATGCAGCTCAAACTTGTCCCAGGAAATTCTGCTGGCACTGTCACTGCTTACTATTTGTCATCACAAGGACCAACACATGATGAAATAGATTTTGAATTCTTGGGAAATTTAAGTGGTGATCCTTATACTCTCCATACTAATGTATTTAGTCAAGGCAAAGGAAACAGAGAACAACAATTTCATCTTTGGTTTGACCCTACTGCTGATTTCCACACGTATTCCATCACTTGGAATCCACAACGCATCATATTTTATGTGGACGGAACGCCAATTAGAGAATACAAGAATAGTGAATCGATTGGAGTTTCATATCCAAAGAACCAACCCATGAGGATATATTCGAGTCTTTGGAATGCAGATGATTGGGCTACAAGAGGAGGACTTGTTAAGACTGATTGGAGCCAAGCACCCTTTAGTGCTTCTTACAGAAACTTCAGTGCTAATGCTTGTATTCCCACTTCTTCATCTTCTTGCAGTTCCATTTCTGCAACTTCAACAAGCAATTCATGGTTGAATGAAGAGTTAGATAACACAAGCCAAGAGAGGCTCAAATGGGTGCAGAAGAATTACATGGTTTATGATTACTGCACTGATTCAAAGCGATTTCCACAGGGATTTCCAGCAGATTGTGTTCAGAATATCTGAGCATTAATAATGAAAAAATAGTGTATTACTTTAAAAACTATTGTATTGATTCTTTTATTGTTTTGTACCCATCAGAAGAAGATGCAATAATTATTGAGGATTAGAAACATCTTAGTTTTGTACTAAGTTATATAAACAATGAAATAGATACTTTTTTTTCTTCTAA

>SlXTH25

CTATTTTTTTCACTCCATAAATAGCCCAATTTTGCCCCACACATTCTCATTATTACAAATAATAAAATAAAAAATGGAATTTTTCCTTCATGATAGAAAATTTATATTATCAGCATTCTTGATTTTATGCATGATTATTGTTGTTTCATGTCGAGGTCCAGTGTACAAACCTCCAGAAATCGAAAAATTAACTGATCATTTTAGTCGATTATCGGTTAATCAGAGTTATAATGTGTTTTATGGAGGTTCTAATATTCATATTACAAATAATGGGTCAAGTGCTGAAATTATTTTAGATAAATCTTCAGGTTCTGGACTAATCTCTAAAGAGAAATATTACTATGGTTTCTTTAATGCTGCACTAAAATTGCCTGCTCATTTTACATCAGGAGTTGTTGTTGCCTTTTATATGTCAAACTCAGATGTGTTTCCACACAACCATGATGAAATAGACTTTGAATTGCTTGGACATGAGAAGAGAAGGGATTGGGTTTTACAGACAAATCTTTATGGAAATGGAAGTGTTCACACTGGAAGAGAAGAAAAATTCTACCTATGGTTTGATCCAACTTTGGATTTTCATGATTATACCATCCTTTGGAATAATCATCACATAGTATTTCTCGTGGACAATGTACCAGTAAGAGAAGTGGTTCACAACACAGCAATCTCTTCAGTTTACCCATCAAAGCCAATGTCAACTATATTGACAATATGGGATGGATCAGAATGGGCAACACATGGAGGAAAATACCCTGTAAACTATAATTATGCACCTTTTATAACAACAATCAAAGGTATTGAATTAGAAGGTTGTGTAAAACAACAACAAAATACATGTTCTAAGAGAAGTAGTACTTCAAGTTTGGACCCTGTTGATGGAGAAGGATTTATGAAGTTATCATCACAACAGATGAAAGGATTGGATTGGGCTAGGAGAAAACATATGTTTTACTCATACTGTCAAGATACTAAGAGATACAAAGTTCTACCACCAGAATGCACTTCTGAATAGACGGAATATGACAGTCTGGTGGACACAGTATCTCGCATTAGCAAGAACGACTCTTCCCTTATGGAGGAACATCTGTTTGACTAAATTCATTCTATGATGTAAAAACTTATTGGTCTGAGTTCCTAACATGTTCAAGAATCTTACTTGGAGGTATTCAAATGAAATAATTTCTTTTTCTCTTTATATTTTCGAGTTTGCTAATCAAATTGATAAAGTCTAAAGATGCTAAATTGTGCAATAATTGAACTTGATTGCATTCTCCAAATATTATGTACTATATAATGGGCATATATAAATAAACATTTAAG

>SlXTH26

ATGGATCATCGAGTTCTTTCATTTGTATCAAAATCAATAACACCTTTCTCTCTCCTATTATTACTGTACATTTTTCCGGCGGCTGAGACGGCGGCGAACATGACGTATAAGGCGTTTAATCTGCCGACGATTACTTTCAAAGAAGGATATTCCCCTCTTTTTAGTGATTTCAATATTGAACGATCTCCTGATGATCGAAGCTTTCGTCTCCTCCTTAATAAATTCTCAGGATCGGGTGTTATTTCAACAGAATATTACAATTATGGATTTTTCAGCGCTAGTATAAAGTTACCGGCCATATATACGGCCGGCATCGTCGTCGCTTTCTATACATCAAATGCAGATACATTTGAGAAGAATCATGATGAGTTAGATATTGAGTTTTTGGGGAATGTGAACGGTCAACCATGGAGGTTTCAGACTAACATGTATGGAAATGGCAGTGTTAGCCGTGGTAGAGAAGAGAGGTATAGAATGTGGTTTGATCCAAGCAAGGACTTTCATCAGTACAGCATTCTTTGGACACCAAAAAACATCATATTTTACATTGATGAAACACCACTTAGAGAAATAAATCGCCATCCAGCAATGGGAGGTGACTTTCCAGCAAAACCAATGGCTTTATATGCCACAATTTGGGATGCATCTTCTTGGGCTACAAATGGTGGCAAAGCTAAAGTGGACTATAAATATGAACCTTTTGCAACAGAGCTTAAAGACTTAGTTCTTGAAGGATGCATAGTAGATCCATCTGAGCAAATTCCATCAACAAATTGCACTGACAGGAATGCTAAATTACTTGCTCAAGATTACTCTAACATCACGCCCGAAAGGCGAAACAACATGAAATTTTTTAGGGAAAGATACATGTACTATTCTTATTGTTATGATAACCTTAGGTACCCTGTGCCACCACCAGAATGTGTGATTGTTCAGTCCGAAAGAGATTTGTTTAGGGACAGTGGAAGGCTTAGGCAGAAGATGAAGTTTGGTGGGAGCCACAGCCATACCCAAAGCCACCGGAAACACCGCCCTGGACGGAGCTCTAGGCGGCGGAATAAGGTGGCTGGCGGTGCATCAAAATCTGGCCGACGAGGTTCTGCTGCTGCTGCAATGTGATAAGTTGTATAACTAAGGTATACAAGGTGAATATTATGGTTTGTAAAAAGTATATTACACTCCTATAGTTGGTATACATACTCATTAGTGGTATAAATATTAATGTGGTGGCTGGGTGAAGAGTAAAAACCACATCCATTAATTTTTATGAGCAATGGTTTTCTTGGTTTCTTCACTATGTATATTGGCACTTCATATTCATCCAAGCAATGTTATGATGAATGGTTGCATGGTTTTCGCAGCTATTTGTCA

>SlXTH27

ATGGCTAATCTCCTCTTAATTGGAGTTGTAATTGCTATGCTATGCTCTGAAATTAAATGTTCATTTGAAGACAACTTTAGTAAAAGTGATTGTCCTGACTCTCACTTCAAGACTTCTGAAGATGGACAGATCTGGTACCTATCATTAGATAACAAAGCAGGTTGTGGATTTATGACCAGACAGAGATATAGATTTGGTTGGTTTAGCATGAAATTGAAATTGGTAGGAGGTGACTCAGCTGGTGTTGTTACAGCTTATTATATGTGCACAGAAGATGGGGCAGGGCCAACTAGAGATGAGCTAGACTTTGAGTTCTTGGGGAATAGGACAGGGGAACCATATCTTATTCAGACAAATGTGTATAAAAATGGTACTGGTGGGCGTGAGATGAGGCATGTTTTATGGTTTGACCCTACTCAAGACTTCCATACATATTCCATTCTTTGGAATTCTCATCAAATTGTATTTTTCGTAGATAAGGTTCCAATAAGAGTATACAGAAACGCGAATTACACGAACAATTTCTTCCCTAACGAGAAGCCAATGTACTTATTTTCGAGCATATGGAACGCGGATGATTGGGCTACTCGGGGCGGGTTAGAGAAAACAGACTGGAAAAATGCACCATTTGTATCAACATATATGGATTTCAATGTTGATGCTTGTCAATGGGAAGATCCTTTCCCTTCTTGTGTTTCAACAACTACTCAAAATTGGTGGGATCAATATAATTCTTGGCACCTTTCAAGTGATCAAAAATTGGACTATGCTTGGGTGCAAAGAAATTTAGTCACTTATGATTATTGCCAAGATATTGAGAGATATAAAGTAAAGCCTGAGGAATGTTGGGTAAGTCCATGGGATTAATTACTATTTCAATTTTGATTTAAGAGTGTGTAGATGTTGCATTGTTTTGTCATGTGTTAAAAATCATGAATTCAATTTTATAATAGGGTTACCCTAATAGCTAAGGATTTCAAATATATGCGAATGACTTTTCATTTTGAGTTTA

>SlXTH28

CTTTTCCTAACAGACCGATAGACCTGAACTTTTTTCTTCTGGACTCTGAAATTAATCTATCTTCAAGTTCTAGACAGCAATGTCATCCTTTATGATTGTCTTTTTGATCCTATCTATGCTACTAAACCCAGGGGTTGGTGTCAACTTCACTGATGTTTTCGAGTCCAGCTGGGCACCGGACCATATTGCTGTTGTAGGAGACGAAGTTACTCTCTCCCTTGACAGCGCTTCTGGCTGCGGATTTGAGTCGAGGTTCAAATATTTGTTCGGGAAAGCCAGTGCACAGATCAAACTAGTTGAAGGAGATTCAGCCGGAACAGTTATTGCATTTTATATGTCATCAGAAGGAGCTAATCACGACGAACTGGACTTTGAATTTCTTGGGAATGTTTCAGGGGAACCATACCTAGTACAAACAAATATCTACGTGAATGGCAGCGGAGATCGAGAGCAGAGGCACGGTCTGTGGTTCGATCCAACAACGGACTTCCACACTTACTCTTTCTTTTGGAATCATCATTCTATCATCTTTTCAGTTGATGATATTCCAATTAGAGTGTTCAAAAACAAGGAGAAAAAAGGTGTTCCATATCCGAAAAATCAAGGCATGGGAATCTATGGATCGTTGTGGAATGCAGATGACTGGGCTACACAAGGAGGGAGAGTGAAGACAAACTGGAGCCACTCTCCATTTGTTACGACATTTCGATCGTTCGAGATCGATGCTTGTGATTTGTGTGGTGAGGACACAATTGCTGCAGGTGCAAAATGTGGCAAGTTAGCTAAATTTTTGTGGGATAAACCATCCAAGAATGGGCTAGAAAAGAGCAAAAAACGCCAATTCAAAATGGTTCAAAACAAGTACTTGGTGTATGATTATTGTAAGGATACTGCAAGATTCAATCAAATGCCTAAAGAGTGCTTGTACTAGAGACACCTCGCAATATTGTAGTTTCTAAAAATGTGTATCCAAATTGCTGATCAAAATTTTCTTGTTTGGCTTGTTATTTGACACTCTTTTAACATTTAAGACAGTTCTATGGTCAAAAAATAAATTCAACTGTGCTATGACTTTGAATATTTAATTCAAAT

>SlXTH29

ATGGCAAAAATCATACATTTTAATTCCTTGGTTTTGATGATTATTGCAACAATCACATTTCAATCATATTTAGCCAATGGATGGACATCAAGTAGCATGTATGTCAATTGGGGTGCTCATCATTGTAAACTTTTAGGGGATGATCTTCAACTTGTTCTTGATAAATCTGCAGGCTCTGGTGCTCAATCGAAAAATTCATTTCTCTTTGGTAGCTTTGAAATGCTTCTCAAGTTGGTACCTAACAACTCTGCTGGAACTGTCACAACATATTATTTATCTTCTACCGGTACCAAGCATGATGAAATCGATTTCGAGTTTCTAGGAAATATATCAGGACATCCTTATATTATACACACAAATATTTACACCCAAGGTGTTGGAAATAGAGAGCAACAATTCTATCCATGGTTTGATCCAACTGCTGCTTTTCACAATTACACCATTCATTGGAACCCTAACGCCGTTGTATGGTACATTGATAGTATTCCAATTAGGGTTTTTAGAAACTACCAATCCAAAGGCATTTCATTCCCAAACCAACAAGGGATGGGAGTCTACACTAGTCTATGGAATGCTGATGATTGGGCAACAAGAGGTGGTCTTGTTAAAATTGATTGGACAAATGCACCATTTATTGCAACTTATAGAAATTTTAGACCAAGAGCTTGCTATTGGAATGGACCAATGAGTATTTCCCAATGTGCAATTCCAACAAATTCCAATTGGTGGGCTTCACCTTCATACTATAAATTGAGTGCAAATAAAGTTGGTGAAATGATCTCAATTAGAAGCAAGAATATGATTTATGATTATTGCAAAGATGTGAAAAGATTCAAGGGAGTTATGCCTATTGAGTGCTCATTGCCCCAATACTAA

>SlXTH30

ATGGGTTTTCATCTAATAAGTCTAAGTGCTCTTTTATTATTAACTAGAGTTTTTGAAGGTCTAGCTTTACCATTTGATAAAAAATACAACATTTCTTGGGGGAACAACAATGTTAAGTTATTGAAAAATGGAGAAGAAATTCAGCTATCTCTTGATAAATTTTCTGGATGTGGGATTGAGTCCAAACAAAGTTATGGCTCTGGATCATTCAAAATGAGAATAAAGCTACCAAGCAAAGACTCAGCTGGAGTAGTGACAACATTTTATCTACATTCACATACAAGCCACCATGATGAATTGGATTTCGAGTTTTTAGGTAATAGAAAAGGGAAACCATACATATTGCAAACAAATGTATTTGCAAATGGTATTGGTGATAGAGAAGAAAGAATTCAACTTTGGTTTGATCCAACAACAAACTTTCATGAGTACTCAATCCTATGGAATTCACATCACATTGTTTTTTTTGTAGATGAAATACCAATTAGGGTTTACAAGAACAAATCATATAGAGGAATTGGATACCCTACACAACCAATGCAATCAGAAGCCACAATATGGAATGGAGAAAGTTGGGCAACAGAAAATGGAAGTCAAAAAATTAATTGGTCAAATTCTCCATTCATAGCTCAATTTCAAGGCTTTAACATTGAAGGTTGCCCTTCTAATTATCATAGTTTAAATTGCAATTCAACAAAGTGGTGGTGGAATTCTAAGAAATTATGGAAATTAACTCTTGATCAAGAAAAATCATATAAAGATATTAGAAGCAAAAATATGATTTATGATTATTGCAAAGATACCAATAGATTTCAAAACATTCCTTTAGAATGTTCAAGTGATTATTAA

>SlXTH31

ATGGCTTCTTTTGAGTTCATGAGTATAATTATTTGTATTTTGATGTATTTTGCTCTGTCACCAATTTATGCTATGGTTGATTTTAATCAATATTATAATCCCTTGTGGGGTCAAAATCATATAACTTATCTTAATCAAAGTACTGAAGTGCAGTTACTTTTGGATCAATCAGGAGGAGCTGGATTCAAATCGAAAACACAATATAACTCTGGATTATTTACATTAAGAATAAAGATGTCAGATAAAAAGACCGATGGAATGATCACAGCTTTCTACTTAATTTCAGATGATCAAGATGCACGGGTTAATCATGATGAAATAGACTTTGAATTTATAGGGACTCAAGGAAAATTACAGACAAATATATTTGCTAATGATATGGGTGGTAGAGAACAAGTTTTTCAACTTCCATTTGATCCTTCTCAGGATTTTCATACTTATCAAATTCTTTATACTCCACAAAGAATAGTGTTTTTTGTGGACAACATACCAATAAGGACATTTGAGAACAACACAAATAGAGGTATCAACTATCCAACAAAATCACTATGGTCAGAAGCAAGCCTATGGATTTCAGATGCTGTGGGTTGGGCTGGATCTGTTGAATGGGGCTATGCACCATTTATAGTTAGTTTTCAAGACTTCAACATTTCTGGTTGCCCTGCTGGTAGTGATTGCTTGCCATCCACAGATTTTAGCCCATGGACTAGGCACAAATTAGCCTCAAGAAGCTTGAATCTTATGAGGAATTTCAGAAAAAAATATATGACTTATGATTATTGTAGCTCTGAGGAAAATAAAAATAGGTACCCAGAATGTGCTTAA

>SlXTH32

CATATATACAAACTTTTGACTTATTTTTGAAAAAACAAATCAAGAAATTTCACTAAAATTGAACCAATTATTTTCAATGGCTTCCTTAGTTCTTTGTTTGGTCATTTTGGCATTTTGCTCTTTACATTATAGTTTGGCTTCTAATAATTTCAATCAAGATTTTGATGTTACATGGGGAGATGGTAGGGCAAAAGTTTTAAACAATGGCAAACTTCTTACTCTTTCCCTTGACAAAGTTTCTGGCTCCGGTGTTAAATCCAAGAAAGAATATTTGTTTGGAAGGATTGATATGCAACTTAAGCTCGTACGTGGAAATTCAGCTGGTACAGTTACTACATATTACTTATCATCACAAGGGTCAACACATGATGAGATAGATTTTGAATTCTTGGGAAACCTTAGTGGAGATCCTTATATTGTTCATACAAATGTGTATACTCAAGGCAAAGGTGATAAGGAACAACAATTCTACTTATGGTTTGATCCCACTGCTGATTTTCATACCTACTCCATTCTTTGGAATCCACAAACAATTATATTTTATGTGGATGGCACACCAATAAGAGTGTTCAAAAACATGGAGTCAAGTGGAGTACCTTACCCAAATAAACAACCTATGAGAGTCTATGCAAGTTTATGGAATGCAGATGATTGGGCCACAAGGGGTGGCCTTGTTAAAACAAATTGGTCCAATGCTCCATTCATAGCTTATTTTAGAAATTTCAAAGACAATAATGCTTGTATTTGGGAATTTGGAAAATCATCATGCACAAATTCAACAAAGTCATGGTTCTATCATGAACTTGATTCTACAAGCCAAGCTAGGTTACAATGGGTGCAAAAGAACTATATGGTTTATAATTATTGTAATGATATTAATAGGTTCCCTCGAGGCCTTCCTCTAGAGTGCGCTTTCAACTCTACGACTAATTAAATCTAGGTTTTCAGACTCTTTTAAAATGTTATCATATGCATGTCAATCTGATGTACACATGCAATCGACATTTTCTGAGAGGAGGGCACTTTCAATTCTACGACTAATTAAATCTAGGTTTTCAGACTCTTTTAAAATATTATCGTATGCGTGTCAATCTGATGTACATATGCAATCGATATTTTCTGAGAGGAGGGCACTTTCAACTCTACGACTAATTAAATCTAGGTTTTC

>SlXTH33

ATGGGCTTCAAATGGACGATGATGTTGGTGTTGTGTGTGTTAATAGGAGGATCAATGGGAGCTAAGCCCAATAAGCCAATTGATGTCCCATTTGGAAGAAATTATGAACCTAGTTGGGCTTTTGATCACATCAAATATTTGAATGGTGGCTCTGAGATCCAGCTCTCCCTCGATAACCGCACCGGCACTGGTTTTCAGTCAAAAGGATCTTACCTATTTGGGCACTTTTCTATGCACATAAAGATGGTTGCTGGTGATTCTGCAGGCACTGTCACTGCTTTCTATTTGTCTTCTCAAAATTCAGAACATGATGAAATAGACTTCGAGTTCTTGGGGAATAAAACAGGAGAACCATACATTTTACAAACAAATGTATACACAGGAGGGAAAGGTGACAAAGAGCAAAGGATTTACTTATGGTTTGATCCAACAAAAGATTATCACACTTACTCTGTCTTGTGGAATCTTCATCAAATTGTATTTTTTGTGGATGAGTATCCCATAAGAGTGTTCAAGAACAACAAGAACTTAGGTGTCAAATTCCCATTTGACCAATCAATGAAGATATACTCAAGTCTATGGGAAGCAGATGATTGGGCAACAAGAGGTGGACTTGAGAAAATTGATTGGTCAAATGCACCCTTTGTTGCCTCATACAAAGGCTTTCACATTGATGGTTGTGAATCTTCTGTCAATGCCAAATTTTGTGCCAATCAAGGCAAGAGTTGGTGGGATCAAAAGGAATTTCAAGATTTGGACAAAACTCAATGGAGGCTTTTGAGAAGAGTTAGGGACAAATATACAATTTATAACTATTGTACTGATAAAAAGAGGTTCTCTACAACGCCAATAGAGTGTAAGAGGAATAGAGATGTTCCAAGGAATTCAAGAAAGGAAAATTAA

>SlXTH34

AATTATCCCTAGCTATTTCCTCATCCTCAAATCTTTGATATAATCACCAAGAAAAAAAATGAATTATTTCTCTAGATTTATTTTCTTGGCCACTTATTTTATTTACTTATCTCATATTGCATTAGCTTCTATAGTTTCTACAGGAGATTACAATAAAGATTTCTACGTAACATATTCACCTAACCATATAAACACTTCTGCTGATGGCCGTACAAGAAGCTTGATATTTGACAAGGAATCTGGTACAGAGATTGCTTCAAAGGATATGTACTTATTTGGTCAATTTGACATGAAAATTAAGTTGATACCAGGAAATTCAGCAGGCACTGTTGTAGCATTTTATTTAGCTTCGGGTCAACCGAATCGCGATGAGATAGATTTTGAATTTCTGGGGAATGTAGATGGAAAACGTTATACTCTTCAAACAAATGTTTATGTTGATGGATTCGACGATAGAGAACAGAGAATCAATTTGTGGTTTGATCCAACACAAGACTACCATACTTATTCTATTCTATGGAACCTTCACCAAATTGTGTTCATGGTAGATTGGGTACCTATTAGAACATACAGAAACCATGCAGATAAAGGAGCTAAGTATCCACATTGGCAGCCAATGGAACTCAAAATGAGCCTATGGAATGGAGAAGATTGGGCAACAGATGGTGGAAAAACAAAAATTGATTGGTCAAAATCACCCTTTGTGGCCACATTGGGAAGTTATAAAATTGATGCTTGTGTTTGGAAAGGGAATGCAAGATTTTGCAGAGTAGAAAATGAAAATCATTGGTGGAATAAGGGGCAATCTAGTACTTTGACATGGACACAAAGAAGATTGTTTAAATGGGTTAGAAAGTATCATTTGACATATGATTATTGTATGGATAATAAAAGGTTTCAAAATAATATGCCCATAGAGTGCTCTCTACCAAAATATTAGTCACGTATGGGCTAAATTTTACGTCTCTTTCAATTACGTGAAAAGATTCTTTTCTTTTTAACTTTACGTCCTCTTACATTTGTTCTTTGTATTAAATCAAAAAG

>SlXTH35

TATCCAATTCTAATTCATCCAACAAACTTTGAAATATTAACAGTTAAACATGGCTTCTTCTTCTAAATTAGTACTAGTAATGTGCTTTATGATTAGTGCTTTTGGCATTGCAATTGGGGCCAAGTTTGATCAAGAATTCGACATTACATGGGGTGATGGCAGAGCAAAAATACTTAACAATGGCGACCTCCTTACTCTCTCACTTGACAAAATCTCAGGCTCTGGTTTTCAATCCAAGAATGAATATCTGTTTGGTAAAATTGACATGCAGCTCAAACTTGTCCCAGGAAATTCTGCTGGCACTGTCACTGCTTACTATTTGTCATCACAAGGACCAACACATGATGAGATAGATTTTGAATTCTTGGGAAATTTAAGTGGTGATCCTTATACTCTCCATACTAATGTATTTAGTCAAGGCAAAGGAAACAGAGAACAACAATTTCATCTCTGGTTTGACCCTACTGCTGATTTCCACACGTATTCCATCACTTGGAATCCGCAACGCATCATATTTTATGTGGACGGAACGCCAATTAGAGAATACAAGAATAGTGAATCGATTGGAGTTTCATATCCAAAGAACCAACCCATGAGGATATATTCGAGTCTTTGGAATGCTGATGATTGGGCTACAAGAGGAGGCCTTGTTAAGACTGATTGGAGCCAAGCACCCTTTAGTGCTTCTTACAGAAACTTCAGCGCTAATGCTTGCATTCCCACTTCTTCATCTTCTTGCAGTTCCAATTCTGCAGCTTCAACTAGCAATTCATGGTTGAATGAAGAGTTAGATAACACAAGCCAAGAGAGGCTCAAATGGGTGCAGAAGAATTACATGGTTTATAATTACTGCACTGATTCAAAGCGATTTCCACAGGGATTTCCAGCAGACTGTGTTCAGAATAACTGAGCATTTATAATGAAAAAATAGTGTATTACTTTAAAAACTATTGTATTGATTCTTTTTATCTAAGATAAATAAATATGTTGGTTTTCTACCAAATTCGTCTAAGTAATGGATATATATATATATTT

>SlXTH36

ATGGTTAACTTTCAAGCAATTCTTGTTTTCATTAGTTTTTTCTTTTTTGTTAATCAATGTTTAAGTGCAAATGAGGTTCCATTTTACCAAAATTATTATCAAAAATATGGAGGTGACCATCTAACTGTTACTGACCAGGGAAAACAAGTTTGCCTAACTATAGACCAATATACAGGTTCTGGATTTATGTCTAACCAACATTTTGGTTCTGGAGATTTTAGCATCGACTTAAAAATACCAAACAAGAACAGTACAGGAGTAATAACAACATTCTACGTACGTACATTTTTTTTTTTATACAAAACGATATATGAATTACACAATCTTATCGATTCATCATCAAAATTGTACGCAATTTATGAATGTTATCGATTAATGAGTGAACATTTACAGTTAACATCACTGCCAATGAATGGAGATCCTGGAATGCATCATGATGAGATTGATTTTGAGTTCCTTGGAGGAGATGGTATATATACATTAAATACAAATATATTTGCAAATGATGGAGGAAGTAGAGAGCAACAATTCAATCTTGATTTTGATCCTACAGAAGATTTCCATACGTATCGAATTCTTTGGAATCAACATCATATCATATTTTACGCGGATAATGTTCCAATAAGAGTTTTCAAGAACAATACTAATTATGGAGTGAATTTTCCAACACACAAAATGCACATTGAAGCAACCATATGGAATGATACAAATTGGGTTGGAGAAGTAGATTGGAGCCAAGGACCATTCAAAGCTTATTATCGCAATTTTACGATTAATGGATGTCAATATCAAGAATCAAATCGTCAAGAATGCTATAATAACAACTATTATTGGAATACAATTACCAGTCTTAGTCCAAATGAAGTTCAGGAATTTGAAACTGTGAAGGCAGAACAAATGATTTTTAGTTATTGCATGAGGAACAATAGTAGAAATTTTCCAGAATGTATATTAAATTGA

>SlXTH37

AACTATTCAACTCAAATTCATCCAACAAACTTTGAAATCTTAACAGTTAAACATGGCTTCTTCTTCTTCTAAGTTAGTACTTGTAATGTGTTTTATGATTAGTGCTTTTGGCATTGCAATTGGGGCCAAGTTTGATCAAGAATTCGACATTACATGGGGTGATGGCAGAGCAAAAATACTTAACAATGGCGACCTCCTTACTCTCTCACTTGACAAAATCTCAGGCTCTGGTTTTCAATCCAAGAATGAATATCTGTTTGGTAAAATTGACATGCAGCTCAAACTTGTCCCAAGAAATTCTGCTGGCACTGTCACTGCTTACTATTTGTCGTCACAAGGACCAACACATGATGAGATAGATTTTGAATTCTTGGGAAATTTAAGTGGTGATCCTTATACTCTCCATACTAATGTATTTAGTCAAGGCAAAGGAAACAGAGAACAACAATTTCATCTTTGGTTTGACCCTACTGCTGATTTCCACACTTATGCCATCACTTGGAATCCACAACGCATCATATTTTATGTGGACGGAACGCCAATTAGAGAATACAAGAATAGTGAATCGATTGGAGTTTCATATCCAAAGAACCAACCCATGAGGATATATTCGAGTCTTTGGAATGCTGATGATTGGGCTACAAGAGGAGGCCTTGTTAAGACTGATTGGAGCCAAGCACCCTTTAGTGCTTCTTACAGAAACTTCAGTGCTAATGCTTGTATTCCCACTTCTTCATCTTCTTGCAGTTCCATTTCTGCAACTTCAACAAGCAATTCATGGTTGAATGAAGAGTTAGATAACACAAGCCAAGAGAGGCTCAAATGGGTGCAGAAGAATTACATGGTTTATGATTACTGCACTGATTCAAAGCGATTTCCACAGGGATTTCCAGCAGATTGTGTTCAGAATATCTGAGCATTAATAATGAAAAAATAGTGTATTACTTTAAAAACTATTGTATTGATTCTTTTATTGTTTTGTACCCATCAGAAGAAGATGCAATAATTATTGAGGATTAGAAACATCTTAGTTTTGTACTAAGTTATATAAACAATGAAATAGATACTTTTTTTTCTTCTAA
