# Supplementary material for: Genome-wide characterization of the xyloglucan endotransglucosylase/hydrolase gene family in Solanum lycopersicum L. and gene expression analysis in response to arbuscular mycorrhizal symbiosis
Source: PeerJ. 2023 May 3;11:e15257. doi: 10.7717/peerj.15257 (PMC10163873; doi:10.7717/peerj.15257)
Supplement: Supplemental Information 4 [file peerj-11-15257-s004.docx]

**File S4.** Amino acid sequences of tomato *XTHs.*

>SlXTH1

MGIIKGVLFSIVLINLSLVVFCGYPRRPVDVPFWKNYEPSWASHHIKFLNGGTTTDLILDRSSGAGFQSKKSYLFGHFSMKMRLVGGDSAGVVTAFYLSSNNAEHDEIDFEFLGNRTGQPYILQTNVFTGGKGNREQRIYLWFDPTKGYHSYSVLWNTYLIVIFVDDVPIRAFKNSKDLGVKFPFNQPMKIYSSLWDADDWATRGGLEKTNWANAPFTASYTSFHVDGCEAATPQEVQVCNTKGMKWWDQKAFQDLDALQYRRLRWVRQKYTVYNYCTDKARYPVPPPECTKDRDI

>SlXTH2

MIKTSSCIFTFFLLICFFVVVAFGGTFDQEFDVTWGYGRVKILENGQLLTLSLDRSSGSGFKSKQQYMFAKIDMKIKLVPGNSAGTATTYYLSSVGSAHDEIDFEFLGNVSGEPYTLHTNVYAQGKGDREQQFHLWFDPTKDFHTYSILWNPRNIIFLVDGTPIRQYKNLEATNGIPYPKNQPMWLYSSLWNAEEWATRGGLVRTDWSKAPFIASYRNFNAQTSKNPTANSWLTQSLDNVGLTRMKWVQKNYMIYNYCTDTKRFPQGFPHECTLN

>SlXTH3

MASSSSKLVLVMCFMISAFGIAIGAKFDQEFDITWGDGRAKILNNGDLLTLSLDKISGSGFQSKNEYLFGKIDMQLKLVPGNSAGTVTAYYLSSQGPTHDEIDFEFLGNLSGDPYTLHTNVFSQGKGNREQQFHLWFDPTADFHTYSITWNPQRIIFYVDGTPIREYKNSESIGVSYPKNQPMRIYSSLWNADDWATRGGLVKTDWSQAPFSASYRNFSANACIPTSSSSCSSNSAASTSNSWLNEELDNTSQERLKWVQKNYMVYDYCTDSKRFPQGFPAD

>SlXTH4

MKGVLVAFVLINLSILASCGAPRKVIDVPFWNNYEPSWSSHHIKYLNGGTTAELLLDKSSGTGFQSKRSYLFGHFSMKMKLVGGDSAGVVTAFYLSSTNAEHDEIDFEFLGNRTGQPYILQTNVFTGGKGDREQRIYLWFDPTKDFHSYSVLWNTYQIAIFVDDVPIRVFKNSKDIGVKFPFNQPMKIYSSLWNADDWATRGGLEKTNWSGAPFIASYTSFHIDGCEAVTPQEVQVCNTNGMKWWDQKAFQDLDGPEYRKLHRVRQNFTIYNYCTDRKRYPTLPLECTRDRDL

>SlXTH5

MKICLSVLFFFHVWFCRAFNDVSTIPFNKGFSHLFGDGNILHANDDNSLQLHLNQNTGSGFKSSDLYNHGFFSAKIKLPSDYTAGIVVAFYTTNQDVFKKTHDELDFEFLGNIKGKAWRFQTNMYGNGSTHRGREERYTLWFDPSKEFHRYSILWTNKNIIFYIDDVPIREIVRNDAMGGDYPSKPMGLYATIWDASDWATSGGKYKTNYKYAPFIAEFTDLVLNGCAMDPLEQVVNPSLCDEKDVELQKSDFSRITSRQRMSMKRFRAKYMYYSYCYDSLRYSVPPPECEIDPVEQQHFKETGRLKFINKHHGHRHPKKTKSEVLDARKYGNEDEE

>SlXTH6

MEFLLYLLLFFLLNSRLINAQGPPSPGYYPSSRAQSIGFNQGFRNLWGPQHQSLDQSTLTIWLDKNSGGSGFKSLKNYRSGYFGSSIKLQPGFTAGIITSFYLSNNQDYPGNHDEIDIEFLGTTPNKPYTLQTNVYIRGSGDGNIIGREMKFHLWFDPTKDYHNYAILWDPNEIIFFVDDVPIRRYPKKNDATFPQRPMYVYGSIWDASSWATEEGRIKADYRYQPFIGKYSNNFKVEGCAAYESPSCRRAPSSSPSGGGGLSRQQIEAMLWVHRNYKVYDYCRDPRRDHTHTPEC

>SlXTH7

MATLTCSSLKNSAFVLILVYALTFSFSLVSARPATFLQDFKIAWSDSHIKQLDGGRGIQLILDQNSGCGFASRSKYLFGRVSMKIKLVPGDSAGTVTAFYMNSDTDNVRDELDFEFLGNRTGQPYTVQTNVYVHGKGDKEQRVNLWFDPSADFHTYTIFWNHHQAVFSVDGIPIRVYKNNEAKGIPFPKFQPMGVYSTLWEADDWATRGGLEKINWSKSPFYAYYKDFDIEGCAMPGPANCASNPSNWWEGPSYQQLSPVQARQYRWVRMNHMIYDYCTDKSRNPVPPPECRAGI

>SlXTH8

MVNFLLEIFIFCYVVVLVSGFSENLETSSFNEGYSQLFGHDNLMVIQDGKSVHISLDERTGAGFVSQDLYLHGLFSASIKLPEDYTAGVVVAFYMSNGDMFEKNHDEIDFEFLGNIRAKNWRIQTNIYGNGSTNVGREERYGLWFDPTEDFHTYTILWTDSHIIFYVDNVPIREIKRTQAMSEDFPSKPMSLYGTIWDGSSWATNGGKYKVNYKYAPYVAKFSDFVLHGCGVDPIELSPKCDIVLDSASIPTRISPDQRRKMERFRNKYLQYSYCYDRTRYNVPQSECVIDPKEANRLRGFDPMTFGGVPRHQNKRHHQRQSRREDTSAK

>SlXTH9

MSSKFSSTLLLLISILMSIQLLASAGNFYRDVDITWGEGRGKIQEGGRGLALSLDKLSGSGFQSKNEYLFGRFDMQLKLVPKNSAGTVTTFFLSSQGEGHDEIDFEFLGNVSGQPYTIHTNVYTQGKGNKEQQFHLWFDPTAAFHTYTIVWNPHRIVFLVDNSPIRVFNNHESMGIPFPKSQAMKVYCSLWNIGHLHHSLLITETLTLMVVQYHQVTSSCKSIGSINNAKPWQTHELDGKGRNRLRWVQTKHMVYNYCADSKRFPQGFSAECKSSRF

>SlXTH10

MLLQLSLLTLVLLSPVSADNFYQDAAVTFGDQRAQIQDGGRLLTLSLDKISGSGFQSKNEYLFGRFDMQLKLVPGNSAGTVTTFYLSSQGAGHDEIDFEFLGNSSGLPYTVHTNVYSQGKGNKEQQFRLWFDPTSSFHTYSIVWNSQRIIFLVDNIPIRVFNNHEALGVAYPKNQAMRVYASLWNADDWATQGGRVKTDWSMAPFTASYRNFNTNACVWSAATSTSSCGGSKTESVNNDETWQTQQLNANGRNRIRWVQQKYMIYNYCADANRFSQGFSPECKRSRF

>SlXTH11

MLLQQLSVLALLLLLCPVWADNFYQDATVTFGDQRAQIQDGGRLLALSLDKISGSGFQSKNEYLFGRFDMQLKLVPGNSAGTVTTFYLSSQGAGHDEIDFEFLGNSSGQPYTVHTNVYSQGKGNKEQQFRLWFDPTSSFHTYSIVWNSQRIIFLVDNIPIRVFNNHEKLGVAFPKNQAMRVYASLWNADDWATQGGRVKTDWSMAPFTASYRNFNTNACVWSAASSTSSCGGSKTDSVNNDQAWQTQELNGNDRNRLRWVQQKYMIYNYCADAKRFSQGLSPECKRSRF

>SlXTH12

MGSFTHYGFLMLALLFSSCMVTYGGNFYQEFDFTWGGNRAKIFNGGQLLSLSLDKVSGSGFQSKKEHLFGRIDMQIKLVAGNSAGTVTTYYLSSQGPTHDEIDFEFLGNVTGEPYILHTNIYAQGKGNKEQQFYLWFDPTKNFHTYSIIWKPQHIIFLVDNTPIRVYKNAESVGVPFPKNQPMRIYSSLWNADDWATRGGLVKTDWAQAPFTAYYRNYMAQSFSPSQFSDQKWQNQELDSNGRRRLRWVQKNFMIYNYCTDIKRFPQGFPPECRRF

>SlXTH13

MALFSSRNSSRSRSSLPYLVFLLIAAFFVFKVDILISQSFSSARRNLEKTPNRIVVNPQKSSEERVVDSLPVVLVNGTFDQHIMISWGDDRGKILENGELLTLSLDKKSGSGFQSKKEYLFAKIDMQIKLVPGNSAGTVTTFYLSSQGNKHDEIDFEFLGNSTGNPYTLHTNVFSLGKGNREQQFFLWFDPTADYHTYSILWNSKCIIFYVDDIPIREYKNPERLGLSYLKYQPMRLYSSLWNADDWATQGGRIKTNWELAPFVASYKNFTYEACIYSRLTSSSSCDIDSPTPINNAWLTYELDRTSRVRMKALQKKHMIYDYCNDKWRFPKGPAPECKLLQ

>SlXTH14

MSTIFFLPIFLCFIFLHSTNANYWPISPGYYPSTKFKSMSFYQGFKNLWGPNHQSVDNNGINIWLDRNSGSGFKSVKPFRSGYFGASIKLQPGYTAGVITAFYLSNNEAHPGFHDEVDIEFLGTTFGKPYTLQTNVYIRGSGDGKIIGREMKFHLWFDPTKNFHHYAILWSPREIIFLVDDVPIRRYARRSDATFPLRPMWLYGSIWDASSWATENGKYKADYNYQPFYGKFTNFKASGCTAYSSRWCRPVSASPYRSGGLSRQQRQAMNWVRSHYMVYDYCRDFKRDHSLTPECWRK

>SlXTH15

MASPIAYFLVLSAIIVVLFSSTQAEVQGSFDDNFSKSCPETHFKTSEDGQIWYLSLDKKAGCGFMTKQKYRFGWFSMKLKLVGGDSAGVVTAYYMCTEDGAGPTRDELDFEFLGNRTGEPYLIQTNVYKNGTGNREMRHVLWFDPTEDFHTYSVLWNTHQIVFFVDKVPIRVYKNANYTNNFFPNEKPMYLFSSIWNADDWATRGGLEKTNWKNQPFVSSYKDFSVDGCQWEDPYPSCVSTTTQNWWDQYDSWHLSSDQKLDYAWVQRNLVIYDYCQDTERFPKKPEECWLNPWE

>SlXTH16

MVSFNWVFSSFVMLFMVGLVSSAKFEELYQPSWAFDHLTTEGEILRMKLDHLSGTGFQSKSKYMFGKVTVQIKLVEGDSAGTVTAFYMSSDGPTHNEFDFEFLGNTTGEPYTVQTNVYVNGVGNREQRLKLWFDPSKDFHSYSIMWNQRQVVFLVDETPVRVHSNLEHRGIPYPKDQPMGVYSSIWNADDWATQGGLVKTDWSHAPFVASYKGFEINGCECPATVAAAENTRRCSSNGQKKYWWDEPVMSELNLHQSHQLIWVRANHMVYDYCTDSARFPVAPVECQHHQHKTNHN

>SlXTH17

MANSHLLLISIVLMGNLVAVLAAGNFNDLTEITWGDGRGKILDGGKGLSLSLDNYSGSGFQSKNEYLYGRFDMQLKLVPKNSAGTVTTFFLSSQGEGHDEIDFEFLGNVTGEPYTVHTNVYSQGKGNKEQQFHLWFDPTAAFHTYTIVWNANRIVFLVDQIPIRVYNNHESIGIAYPKSQPMKVYCSLWNADEWATQGGRVKTDWSQAPFTAYYRNINIDGCVVKSGASSCASRSTESTNSAKSWETHELDAKGRNRVRWVQSKHMVYNYCADSKRFPQGYSQECKRSRF

>SlXTH18

MAKLIDFNSLVLMIIAIIALFHSYVVIGMTSSSMYVNWGAHHCKLLGDDLQLVLDKSAGSGAQSKRSFLFGSFEMLIKLVPNNSAGTVTTYYLSSTGTKHDEIDFEFLGNISGQPYIIHTNIYTQGVGNREQQFYPWFDPTADFHNYTIHWNPNAVVWYIDSIPIRVFRNYQSKGIPFPNKQGMRVYTSLWNADDWATRGGLVKIDWTNAPFIATYRKFRPRACYWNGPMSISQCSIPTKTNWWSSPTYNKLSANKLGQMNSMRSKYMIYDYCKDVKRFKGVIPIECSLPQY

>SlXTH19

MQFKNTYTMKTTFLLFLILSFFFSALAGNFNQDFDITWGDDRAKILENGQLMTLSLDKVSGSGFRSKNQYLFGKIDLKIKLVPGNSAGTVTTYYLSSIGSSHDEIDFEFLGNLSGDPYILHTNVFTQGKGDREQQFYLWFDPTKDFHTYSILWNPQSIIFSVDGTPIRQFKNLESSGIPYPKSQPMWIYSSLWNADDWATRGGLVKIDWTKAPFIASYTNFNAQACVWSSTSTSSSCNSTTQDSWLSENLDITGKSRIKWVQNNYMIYNYCNDIKRFPQGFP

>SlXTH20

MPFLFSFNIRLILVLVFISCMVVKYCASNDLNQDFDITWGNERGKILNNGEILTLTLDNISGSGFQSKKEYLFGKIDMQIKLVQGNSAGTVTAYYLSSQGSSHDEIDFEFLGNLSGEPYTLHTNVYTQGKGDREQQFHLWFDPANDFHTYSILWNPQTIVFSVDNVPIREFKNMENIGVAFPKSQSMKLYSSLWNADEWATRGGLIKTDWAQAPFTASYRNFNANICNNNNNNNDSCKYLVENLDPVNEEKLRRVQQKYMIYNYCTDNKRFPQGFPLECSVS

>SlXTH21

MVNYYMFFFIFLSCILVLVSGFSRNLPILAFDEGYSHLFGDNNLMILKDGKSVHISLDKRTGAGFVSQDLYFHGFFSASIKLPADYTAGVVVAFYMSNGDMFEKNHDEIDFEFLGNIRGKDWRIQTNIYGNGSTNVGREERYGLWFDPSEDFHQYSILWTENLIIFYVDNVPIREIKRTKAMGGDFPSKPMSLIATIWDGSNWATNGGKYKVNYKYAPYIAEFSDFILHGCAVDPIELSSKCDNTTPKTPTIPTDITLDQRRKMENFRKKQMQYSYCYDKTRYKVPPPECVIDPKEAERLRAFDPVTFGGSHHHHGRRHHRSRPKLKGDDDVSFM

>SlXTH22

MGSSLVLSLANLLIISTIVSFGSLVMVNGIFSDNMYINWGSHHSWMQGDDLQLVLDQSSGSGVQSKGTFLFGSIEMQIKLVPGNSAGTVTAYYLSSTGDKHDEIDFEFLGNVSGQPYIIHTNIFTQGAGGREQQFYPWFDPTADYHNYTIHWNPNAVVWYVDDIPIRVYKNYQSQDIPYPNAQAMGVYSSLWNADSWATRGGLVKCDWTNAPFIAKYRNFAPRACAWNGPISISQCATQTPSNWYTAPEYNQLSYAKQGQMEWVRSNYMIYDYCKDTKRFNGQFPGECFKPQF

>SlXTH23

MESNASSMARVLLILSVIFTLFSSSNGVVGGAFEENFSKSCPGTHFKTSKDGQIWYLTLDQVSDCGFITKQSYRFGWYSTKLKLVGGDSAGVVTAFYMCSEVEAGPLRDEIDFEFLGNRTGQPYLIQTNVYNNGSGGREMRHQLWFDPTLDFHTYSILWNSHQIVFFVDKVPIRVYKNANHTNNFFPAQRPMYVFSSIWNADNWATRGGLDKINWENAPFVASYKDFTIDACPWKNPYPACASSTTQHWWDQNNTWHLSSKEKIDYAWVQRNFVVYNYCQDTVRNKYKPQECWLNPLD

>SlXTH24

MASSSKLVLVMCFMISAFGIAIGAKFDQEFDITWGDGRAKILNNGDLLTLSLDKISGSGFQPKNEYLFGKIDMQLKLVPGNSAGTVTAYYLSSQGPTHDEIDFEFLGNLSGDPYTLHTNVFSQGKGNREQQFHLWFDPTADFHTYSITWNPQRIIFYVDGTPIREYKNSESIGVSYPKNQPMRIYSSLWNADDWATRGGLVKTDWSQAPFSASYRNFSANACIPTSSSSCSSISATSTSNSWLNEELDNTSQERLKWVQKNYMVYDYCTDSKRFPQGFPADCVQNI

>SlXTH25

MEFFLHDRKFILSAFLILCMIIVVSCRGPVYKPPEIEKLTDHFSRLSVNQSYNVFYGGSNIHITNNGSSAEIILDKSSGSGLISKEKYYYGFFNAALKLPAHFTSGVVVAFYMSNSDVFPHNHDEIDFELLGHEKRRDWVLQTNLYGNGSVHTGREEKFYLWFDPTLDFHDYTILWNNHHIVFLVDNVPVREVVHNTAISSVYPSKPMSTILTIWDGSEWATHGGKYPVNYNYAPFITTIKGIELEGCVKQQQNTCSKRSSTSSLDPVDGEGFMKLSSQQMKGLDWARRKHMFYSYCQDTKRYKVLPPECTSE

>SlXTH26

MDHRVLSFVSKSITPFSLLLLLYIFPAAETAANMTYKAFNLPTITFKEGYSPLFSDFNIERSPDDRSFRLLLNKFSGSGVISTEYYNYGFFSASIKLPAIYTAGIVVAFYTSNADTFEKNHDELDIEFLGNVNGQPWRFQTNMYGNGSVSRGREERYRMWFDPSKDFHQYSILWTPKNIIFYIDETPLREINRHPAMGGDFPAKPMALYATIWDASSWATNGGKAKVDYKYEPFATELKDLVLEGCIVDPSEQIPSTNCTDRNAKLLAQDYSNITPERRNNMKFFRERYMYYSYCYDNLRYPVPPPECVIVQSERDLFRDSGRLRQKMKFGGSHSHTQSHRKHRPGRSSRRRNKVAGGASKSGRRGSAAAAM

>SlXTH27

MANLLLIGVVIAMLCSEIKCSFEDNFSKSDCPDSHFKTSEDGQIWYLSLDNKAGCGFMTRQRYRFGWFSMKLKLVGGDSAGVVTAYYMCTEDGAGPTRDELDFEFLGNRTGEPYLIQTNVYKNGTGGREMRHVLWFDPTQDFHTYSILWNSHQIVFFVDKVPIRVYRNANYTNNFFPNEKPMYLFSSIWNADDWATRGGLEKTDWKNAPFVSTYMDFNVDACQWEDPFPSCVSTTTQNWWDQYNSWHLSSDQKLDYAWVQRNLVTYDYCQDIERYKVKPEECWVSPWD

>SlXTH28

MSSFMIVFLILSMLLNPGVGVNFTDVFESSWAPDHIAVVGDEVTLSLDSASGCGFESRFKYLFGKASAQIKLVEGDSAGTVIAFYMSSEGANHDELDFEFLGNVSGEPYLVQTNIYVNGSGDREQRHGLWFDPTTDFHTYSFFWNHHSIIFSVDDIPIRVFKNKEKKGVPYPKNQGMGIYGSLWNADDWATQGGRVKTNWSHSPFVTTFRSFEIDACDLCGEDTIAAGAKCGKLAKFLWDKPSKNGLEKSKKRQFKMVQNKYLVYDYCKDTARFNQMPKECLY

>SlXTH29

MAKIIHFNSLVLMIIATITFQSYLANGWTSSSMYVNWGAHHCKLLGDDLQLVLDKSAGSGAQSKNSFLFGSFEMLLKLVPNNSAGTVTTYYLSSTGTKHDEIDFEFLGNISGHPYIIHTNIYTQGVGNREQQFYPWFDPTAAFHNYTIHWNPNAVVWYIDSIPIRVFRNYQSKGISFPNQQGMGVYTSLWNADDWATRGGLVKIDWTNAPFIATYRNFRPRACYWNGPMSISQCAIPTNSNWWASPSYYKLSANKVGEMISIRSKNMIYDYCKDVKRFKGVMPIECSLPQY

>SlXTH30

MGFHLISLSALLLLTRVFEGLALPFDKKYNISWGNNNVKLLKNGEEIQLSLDKFSGCGIESKQSYGSGSFKMRIKLPSKDSAGVVTTFYLHSHTSHHDELDFEFLGNRKGKPYILQTNVFANGIGDREERIQLWFDPTTNFHEYSILWNSHHIVFFVDEIPIRVYKNKSYRGIGYPTQPMQSEATIWNGESWATENGSQKINWSNSPFIAQFQGFNIEGCPSNYHSLNCNSTKWWWNSKKLWKLTLDQEKSYKDIRSKNMIYDYCKDTNRFQNIPLECSSDY

>SlXTH31

MASFEFMSIIICILMYFALSPIYAMVDFNQYYNPLWGQNHITYLNQSTEVQLLLDQSGGAGFKSKTQYNSGLFTLRIKMSDKKTDGMITAFYLISDDQDARVNHDEIDFEFIGTQGKLQTNIFANDMGGREQVFQLPFDPSQDFHTYQILYTPQRIVFFVDNIPIRTFENNTNRGINYPTKSLWSEASLWISDAVGWAGSVEWGYAPFIVSFQDFNISGCPAGSDCLPSTDFSPWTRHKLASRSLNLMRNFRKKYMTYDYCSSEENKNRYPECA

>SlXTH32

MASLVLCLVILAFCSLHYSLASNNFNQDFDVTWGDGRAKVLNNGKLLTLSLDKVSGSGVKSKKEYLFGRIDMQLKLVRGNSAGTVTTYYLSSQGSTHDEIDFEFLGNLSGDPYIVHTNVYTQGKGDKEQQFYLWFDPTADFHTYSILWNPQTIIFYVDGTPIRVFKNMESSGVPYPNKQPMRVYASLWNADDWATRGGLVKTNWSNAPFIAYFRNFKDNNACIWEFGKSSCTNSTKSWFYHELDSTSQARLQWVQKNYMVYNYCNDINRFPRGLPLECAFNSTTN

>SlXTH33

MGFKWTMMLVLCVLIGGSMGAKPNKPIDVPFGRNYEPSWAFDHIKYLNGGSEIQLSLDNRTGTGFQSKGSYLFGHFSMHIKMVAGDSAGTVTAFYLSSQNSEHDEIDFEFLGNKTGEPYILQTNVYTGGKGDKEQRIYLWFDPTKDYHTYSVLWNLHQIVFFVDEYPIRVFKNNKNLGVKFPFDQSMKIYSSLWEADDWATRGGLEKIDWSNAPFVASYKGFHIDGCESSVNAKFCANQGKSWWDQKEFQDLDKTQWRLLRRVRDKYTIYNYCTDKKRFSTTPIECKRNRDVPRNSRKEN

>SlXTH34

MNYFSRFIFLATYFIYLSHIALASIVSTGDYNKDFYVTYSPNHINTSADGRTRSLIFDKESGTEIASKDMYLFGQFDMKIKLIPGNSAGTVVAFYLASGQPNRDEIDFEFLGNVDGKRYTLQTNVYVDGFDDREQRINLWFDPTQDYHTYSILWNLHQIVFMVDWVPIRTYRNHADKGAKYPHWQPMELKMSLWNGEDWATDGGKTKIDWSKSPFVATLGSYKIDACVWKGNARFCRVENENHWWNKGQSSTLTWTQRRLFKWVRKYHLTYDYCMDNKRFQNNMPIECSLPKY

>SlXTH35

MASSSKLVLVMCFMISAFGIAIGAKFDQEFDITWGDGRAKILNNGDLLTLSLDKISGSGFQSKNEYLFGKIDMQLKLVPGNSAGTVTAYYLSSQGPTHDEIDFEFLGNLSGDPYTLHTNVFSQGKGNREQQFHLWFDPTADFHTYSITWNPQRIIFYVDGTPIREYKNSESIGVSYPKNQPMRIYSSLWNADDWATRGGLVKTDWSQAPFSASYRNFSANACIPTSSSSCSSNSAASTSNSWLNEELDNTSQERLKWVQKNYMVYNYCTDSKRFPQGFPADCVQNN

>SlXTH36

MVNFQAILVFISFFFFVNQCLSANEVPFYQNYYQKYGGDHLTVTDQGKQVCLTIDQYTGSGFMSNQHFGSGDFSIDLKIPNKNSTGVITTFYVRTFFFLYKTIYELHNLIDSSSKLYAIYECYRLMSEHLQLTSLPMNGDPGMHHDEIDFEFLGGDGIYTLNTNIFANDGGSREQQFNLDFDPTEDFHTYRILWNQHHIIFYADNVPIRVFKNNTNYGVNFPTHKMHIEATIWNDTNWVGEVDWSQGPFKAYYRNFTINGCQYQESNRQECYNNNYYWNTITSLSPNEVQEFETVKAEQMIFSYCMRNNSRNFPECILN

>SlXTH37

MASSSSKLVLVMCFMISAFGIAIGAKFDQEFDITWGDGRAKILNNGDLLTLSLDKISGSGFQSKNEYLFGKIDMQLKLVPRNSAGTVTAYYLSSQGPTHDEIDFEFLGNLSGDPYTLHTNVFSQGKGNREQQFHLWFDPTADFHTYAITWNPQRIIFYVDGTPIREYKNSESIGVSYPKNQPMRIYSSLWNADDWATRGGLVKTDWSQAPFSASYRNFSANACIPTSSSSCSSISATSTSNSWLNEELDNTSQERLKWVQKNYMVYDYCTDSKRFPQGFPADCVQNI
