# Supplementary material for: Genome-wide characterization of the xyloglucan endotransglucosylase/hydrolase gene family in Solanum lycopersicum L. and gene expression analysis in response to arbuscular mycorrhizal symbiosis
Source: PeerJ. 2023 May 3;11:e15257. doi: 10.7717/peerj.15257 (PMC10163873; doi:10.7717/peerj.15257)
Supplement: Supplemental Information 5 [file peerj-11-15257-s005.docx]

**File S5.** Amino acid sequences of *XTH* genes from *S. lycopersicum, N. tabacum, P. axillaris, S. tuberosum* and *A. thaliana* used for phylogenetic analysis*.*

>SlXTH1

MGIIKGVLFSIVLINLSLVVFCGYPRRPVDVPFWKNYEPSWASHHIKFLNGGTTTDLILDRSSGAGFQSKKSYLFGHFSMKMRLVGGDSAGVVTAFYLSSNNAEHDEIDFEFLGNRTGQPYILQTNVFTGGKGNREQRIYLWFDPTKGYHSYSVLWNTYLIVIFVDDVPIRAFKNSKDLGVKFPFNQPMKIYSSLWDADDWATRGGLEKTNWANAPFTASYTSFHVDGCEAATPQEVQVCNTKGMKWWDQKAFQDLDALQYRRLRWVRQKYTVYNYCTDKARYPVPPPECTKDRDI

>SlXTH2

MIKTSSCIFTFFLLICFFVVVAFGGTFDQEFDVTWGYGRVKILENGQLLTLSLDRSSGSGFKSKQQYMFAKIDMKIKLVPGNSAGTATTYYLSSVGSAHDEIDFEFLGNVSGEPYTLHTNVYAQGKGDREQQFHLWFDPTKDFHTYSILWNPRNIIFLVDGTPIRQYKNLEATNGIPYPKNQPMWLYSSLWNAEEWATRGGLVRTDWSKAPFIASYRNFNAQTSKNPTANSWLTQSLDNVGLTRMKWVQKNYMIYNYCTDTKRFPQGFPHECTLN

>SlXTH3

MASSSSKLVLVMCFMISAFGIAIGAKFDQEFDITWGDGRAKILNNGDLLTLSLDKISGSGFQSKNEYLFGKIDMQLKLVPGNSAGTVTAYYLSSQGPTHDEIDFEFLGNLSGDPYTLHTNVFSQGKGNREQQFHLWFDPTADFHTYSITWNPQRIIFYVDGTPIREYKNSESIGVSYPKNQPMRIYSSLWNADDWATRGGLVKTDWSQAPFSASYRNFSANACIPTSSSSCSSNSAASTSNSWLNEELDNTSQERLKWVQKNYMVYDYCTDSKRFPQGFPAD

>SlXTH4

MKGVLVAFVLINLSILASCGAPRKVIDVPFWNNYEPSWSSHHIKYLNGGTTAELLLDKSSGTGFQSKRSYLFGHFSMKMKLVGGDSAGVVTAFYLSSTNAEHDEIDFEFLGNRTGQPYILQTNVFTGGKGDREQRIYLWFDPTKDFHSYSVLWNTYQIAIFVDDVPIRVFKNSKDIGVKFPFNQPMKIYSSLWNADDWATRGGLEKTNWSGAPFIASYTSFHIDGCEAVTPQEVQVCNTNGMKWWDQKAFQDLDGPEYRKLHRVRQNFTIYNYCTDRKRYPTLPLECTRDRDL

>SlXTH5

MKICLSVLFFFHVWFCRAFNDVSTIPFNKGFSHLFGDGNILHANDDNSLQLHLNQNTGSGFKSSDLYNHGFFSAKIKLPSDYTAGIVVAFYTTNQDVFKKTHDELDFEFLGNIKGKAWRFQTNMYGNGSTHRGREERYTLWFDPSKEFHRYSILWTNKNIIFYIDDVPIREIVRNDAMGGDYPSKPMGLYATIWDASDWATSGGKYKTNYKYAPFIAEFTDLVLNGCAMDPLEQVVNPSLCDEKDVELQKSDFSRITSRQRMSMKRFRAKYMYYSYCYDSLRYSVPPPECEIDPVEQQHFKETGRLKFINKHHGHRHPKKTKSEVLDARKYGNEDEE

>SlXTH6

MEFLLYLLLFFLLNSRLINAQGPPSPGYYPSSRAQSIGFNQGFRNLWGPQHQSLDQSTLTIWLDKNSGGSGFKSLKNYRSGYFGSSIKLQPGFTAGIITSFYLSNNQDYPGNHDEIDIEFLGTTPNKPYTLQTNVYIRGSGDGNIIGREMKFHLWFDPTKDYHNYAILWDPNEIIFFVDDVPIRRYPKKNDATFPQRPMYVYGSIWDASSWATEEGRIKADYRYQPFIGKYSNNFKVEGCAAYESPSCRRAPSSSPSGGGGLSRQQIEAMLWVHRNYKVYDYCRDPRRDHTHTPEC

>SlXTH7

MATLTCSSLKNSAFVLILVYALTFSFSLVSARPATFLQDFKIAWSDSHIKQLDGGRGIQLILDQNSGCGFASRSKYLFGRVSMKIKLVPGDSAGTVTAFYMNSDTDNVRDELDFEFLGNRTGQPYTVQTNVYVHGKGDKEQRVNLWFDPSADFHTYTIFWNHHQAVFSVDGIPIRVYKNNEAKGIPFPKFQPMGVYSTLWEADDWATRGGLEKINWSKSPFYAYYKDFDIEGCAMPGPANCASNPSNWWEGPSYQQLSPVQARQYRWVRMNHMIYDYCTDKSRNPVPPPECRAGI

>SlXTH8

MVNFLLEIFIFCYVVVLVSGFSENLETSSFNEGYSQLFGHDNLMVIQDGKSVHISLDERTGAGFVSQDLYLHGLFSASIKLPEDYTAGVVVAFYMSNGDMFEKNHDEIDFEFLGNIRAKNWRIQTNIYGNGSTNVGREERYGLWFDPTEDFHTYTILWTDSHIIFYVDNVPIREIKRTQAMSEDFPSKPMSLYGTIWDGSSWATNGGKYKVNYKYAPYVAKFSDFVLHGCGVDPIELSPKCDIVLDSASIPTRISPDQRRKMERFRNKYLQYSYCYDRTRYNVPQSECVIDPKEANRLRGFDPMTFGGVPRHQNKRHHQRQSRREDTSAK

>SlXTH9

MSSKFSSTLLLLISILMSIQLLASAGNFYRDVDITWGEGRGKIQEGGRGLALSLDKLSGSGFQSKNEYLFGRFDMQLKLVPKNSAGTVTTFFLSSQGEGHDEIDFEFLGNVSGQPYTIHTNVYTQGKGNKEQQFHLWFDPTAAFHTYTIVWNPHRIVFLVDNSPIRVFNNHESMGIPFPKSQAMKVYCSLWNIGHLHHSLLITETLTLMVVQYHQVTSSCKSIGSINNAKPWQTHELDGKGRNRLRWVQTKHMVYNYCADSKRFPQGFSAECKSSRF

>SlXTH10

MLLQLSLLTLVLLSPVSADNFYQDAAVTFGDQRAQIQDGGRLLTLSLDKISGSGFQSKNEYLFGRFDMQLKLVPGNSAGTVTTFYLSSQGAGHDEIDFEFLGNSSGLPYTVHTNVYSQGKGNKEQQFRLWFDPTSSFHTYSIVWNSQRIIFLVDNIPIRVFNNHEALGVAYPKNQAMRVYASLWNADDWATQGGRVKTDWSMAPFTASYRNFNTNACVWSAATSTSSCGGSKTESVNNDETWQTQQLNANGRNRIRWVQQKYMIYNYCADANRFSQGFSPECKRSRF

>SlXTH11

MLLQQLSVLALLLLLCPVWADNFYQDATVTFGDQRAQIQDGGRLLALSLDKISGSGFQSKNEYLFGRFDMQLKLVPGNSAGTVTTFYLSSQGAGHDEIDFEFLGNSSGQPYTVHTNVYSQGKGNKEQQFRLWFDPTSSFHTYSIVWNSQRIIFLVDNIPIRVFNNHEKLGVAFPKNQAMRVYASLWNADDWATQGGRVKTDWSMAPFTASYRNFNTNACVWSAASSTSSCGGSKTDSVNNDQAWQTQELNGNDRNRLRWVQQKYMIYNYCADAKRFSQGLSPECKRSRF

>SlXTH12

MGSFTHYGFLMLALLFSSCMVTYGGNFYQEFDFTWGGNRAKIFNGGQLLSLSLDKVSGSGFQSKKEHLFGRIDMQIKLVAGNSAGTVTTYYLSSQGPTHDEIDFEFLGNVTGEPYILHTNIYAQGKGNKEQQFYLWFDPTKNFHTYSIIWKPQHIIFLVDNTPIRVYKNAESVGVPFPKNQPMRIYSSLWNADDWATRGGLVKTDWAQAPFTAYYRNYMAQSFSPSQFSDQKWQNQELDSNGRRRLRWVQKNFMIYNYCTDIKRFPQGFPPECRRF

>SlXTH13

MALFSSRNSSRSRSSLPYLVFLLIAAFFVFKVDILISQSFSSARRNLEKTPNRIVVNPQKSSEERVVDSLPVVLVNGTFDQHIMISWGDDRGKILENGELLTLSLDKKSGSGFQSKKEYLFAKIDMQIKLVPGNSAGTVTTFYLSSQGNKHDEIDFEFLGNSTGNPYTLHTNVFSLGKGNREQQFFLWFDPTADYHTYSILWNSKCIIFYVDDIPIREYKNPERLGLSYLKYQPMRLYSSLWNADDWATQGGRIKTNWELAPFVASYKNFTYEACIYSRLTSSSSCDIDSPTPINNAWLTYELDRTSRVRMKALQKKHMIYDYCNDKWRFPKGPAPECKLLQ

>SlXTH14

MSTIFFLPIFLCFIFLHSTNANYWPISPGYYPSTKFKSMSFYQGFKNLWGPNHQSVDNNGINIWLDRNSGSGFKSVKPFRSGYFGASIKLQPGYTAGVITAFYLSNNEAHPGFHDEVDIEFLGTTFGKPYTLQTNVYIRGSGDGKIIGREMKFHLWFDPTKNFHHYAILWSPREIIFLVDDVPIRRYARRSDATFPLRPMWLYGSIWDASSWATENGKYKADYNYQPFYGKFTNFKASGCTAYSSRWCRPVSASPYRSGGLSRQQRQAMNWVRSHYMVYDYCRDFKRDHSLTPECWRK

>SlXTH15

MASPIAYFLVLSAIIVVLFSSTQAEVQGSFDDNFSKSCPETHFKTSEDGQIWYLSLDKKAGCGFMTKQKYRFGWFSMKLKLVGGDSAGVVTAYYMCTEDGAGPTRDELDFEFLGNRTGEPYLIQTNVYKNGTGNREMRHVLWFDPTEDFHTYSVLWNTHQIVFFVDKVPIRVYKNANYTNNFFPNEKPMYLFSSIWNADDWATRGGLEKTNWKNQPFVSSYKDFSVDGCQWEDPYPSCVSTTTQNWWDQYDSWHLSSDQKLDYAWVQRNLVIYDYCQDTERFPKKPEECWLNPWE

>SlXTH16

MVSFNWVFSSFVMLFMVGLVSSAKFEELYQPSWAFDHLTTEGEILRMKLDHLSGTGFQSKSKYMFGKVTVQIKLVEGDSAGTVTAFYMSSDGPTHNEFDFEFLGNTTGEPYTVQTNVYVNGVGNREQRLKLWFDPSKDFHSYSIMWNQRQVVFLVDETPVRVHSNLEHRGIPYPKDQPMGVYSSIWNADDWATQGGLVKTDWSHAPFVASYKGFEINGCECPATVAAAENTRRCSSNGQKKYWWDEPVMSELNLHQSHQLIWVRANHMVYDYCTDSARFPVAPVECQHHQHKTNHN

>SlXTH17

MANSHLLLISIVLMGNLVAVLAAGNFNDLTEITWGDGRGKILDGGKGLSLSLDNYSGSGFQSKNEYLYGRFDMQLKLVPKNSAGTVTTFFLSSQGEGHDEIDFEFLGNVTGEPYTVHTNVYSQGKGNKEQQFHLWFDPTAAFHTYTIVWNANRIVFLVDQIPIRVYNNHESIGIAYPKSQPMKVYCSLWNADEWATQGGRVKTDWSQAPFTAYYRNINIDGCVVKSGASSCASRSTESTNSAKSWETHELDAKGRNRVRWVQSKHMVYNYCADSKRFPQGYSQECKRSRF

>SlXTH18

MAKLIDFNSLVLMIIAIIALFHSYVVIGMTSSSMYVNWGAHHCKLLGDDLQLVLDKSAGSGAQSKRSFLFGSFEMLIKLVPNNSAGTVTTYYLSSTGTKHDEIDFEFLGNISGQPYIIHTNIYTQGVGNREQQFYPWFDPTADFHNYTIHWNPNAVVWYIDSIPIRVFRNYQSKGIPFPNKQGMRVYTSLWNADDWATRGGLVKIDWTNAPFIATYRKFRPRACYWNGPMSISQCSIPTKTNWWSSPTYNKLSANKLGQMNSMRSKYMIYDYCKDVKRFKGVIPIECSLPQY

>SlXTH19

MQFKNTYTMKTTFLLFLILSFFFSALAGNFNQDFDITWGDDRAKILENGQLMTLSLDKVSGSGFRSKNQYLFGKIDLKIKLVPGNSAGTVTTYYLSSIGSSHDEIDFEFLGNLSGDPYILHTNVFTQGKGDREQQFYLWFDPTKDFHTYSILWNPQSIIFSVDGTPIRQFKNLESSGIPYPKSQPMWIYSSLWNADDWATRGGLVKIDWTKAPFIASYTNFNAQACVWSSTSTSSSCNSTTQDSWLSENLDITGKSRIKWVQNNYMIYNYCNDIKRFPQGFP

>SlXTH20

MPFLFSFNIRLILVLVFISCMVVKYCASNDLNQDFDITWGNERGKILNNGEILTLTLDNISGSGFQSKKEYLFGKIDMQIKLVQGNSAGTVTAYYLSSQGSSHDEIDFEFLGNLSGEPYTLHTNVYTQGKGDREQQFHLWFDPANDFHTYSILWNPQTIVFSVDNVPIREFKNMENIGVAFPKSQSMKLYSSLWNADEWATRGGLIKTDWAQAPFTASYRNFNANICNNNNNNNDSCKYLVENLDPVNEEKLRRVQQKYMIYNYCTDNKRFPQGFPLECSVS

>SlXTH21

MVNYYMFFFIFLSCILVLVSGFSRNLPILAFDEGYSHLFGDNNLMILKDGKSVHISLDKRTGAGFVSQDLYFHGFFSASIKLPADYTAGVVVAFYMSNGDMFEKNHDEIDFEFLGNIRGKDWRIQTNIYGNGSTNVGREERYGLWFDPSEDFHQYSILWTENLIIFYVDNVPIREIKRTKAMGGDFPSKPMSLIATIWDGSNWATNGGKYKVNYKYAPYIAEFSDFILHGCAVDPIELSSKCDNTTPKTPTIPTDITLDQRRKMENFRKKQMQYSYCYDKTRYKVPPPECVIDPKEAERLRAFDPVTFGGSHHHHGRRHHRSRPKLKGDDDVSFM

>SlXTH22

MGSSLVLSLANLLIISTIVSFGSLVMVNGIFSDNMYINWGSHHSWMQGDDLQLVLDQSSGSGVQSKGTFLFGSIEMQIKLVPGNSAGTVTAYYLSSTGDKHDEIDFEFLGNVSGQPYIIHTNIFTQGAGGREQQFYPWFDPTADYHNYTIHWNPNAVVWYVDDIPIRVYKNYQSQDIPYPNAQAMGVYSSLWNADSWATRGGLVKCDWTNAPFIAKYRNFAPRACAWNGPISISQCATQTPSNWYTAPEYNQLSYAKQGQMEWVRSNYMIYDYCKDTKRFNGQFPGECFKPQF

>SlXTH23

MESNASSMARVLLILSVIFTLFSSSNGVVGGAFEENFSKSCPGTHFKTSKDGQIWYLTLDQVSDCGFITKQSYRFGWYSTKLKLVGGDSAGVVTAFYMCSEVEAGPLRDEIDFEFLGNRTGQPYLIQTNVYNNGSGGREMRHQLWFDPTLDFHTYSILWNSHQIVFFVDKVPIRVYKNANHTNNFFPAQRPMYVFSSIWNADNWATRGGLDKINWENAPFVASYKDFTIDACPWKNPYPACASSTTQHWWDQNNTWHLSSKEKIDYAWVQRNFVVYNYCQDTVRNKYKPQECWLNPLD

>SlXTH24

MASSSKLVLVMCFMISAFGIAIGAKFDQEFDITWGDGRAKILNNGDLLTLSLDKISGSGFQPKNEYLFGKIDMQLKLVPGNSAGTVTAYYLSSQGPTHDEIDFEFLGNLSGDPYTLHTNVFSQGKGNREQQFHLWFDPTADFHTYSITWNPQRIIFYVDGTPIREYKNSESIGVSYPKNQPMRIYSSLWNADDWATRGGLVKTDWSQAPFSASYRNFSANACIPTSSSSCSSISATSTSNSWLNEELDNTSQERLKWVQKNYMVYDYCTDSKRFPQGFPADCVQNI

>SlXTH25

MEFFLHDRKFILSAFLILCMIIVVSCRGPVYKPPEIEKLTDHFSRLSVNQSYNVFYGGSNIHITNNGSSAEIILDKSSGSGLISKEKYYYGFFNAALKLPAHFTSGVVVAFYMSNSDVFPHNHDEIDFELLGHEKRRDWVLQTNLYGNGSVHTGREEKFYLWFDPTLDFHDYTILWNNHHIVFLVDNVPVREVVHNTAISSVYPSKPMSTILTIWDGSEWATHGGKYPVNYNYAPFITTIKGIELEGCVKQQQNTCSKRSSTSSLDPVDGEGFMKLSSQQMKGLDWARRKHMFYSYCQDTKRYKVLPPECTSE

>SlXTH26

MDHRVLSFVSKSITPFSLLLLLYIFPAAETAANMTYKAFNLPTITFKEGYSPLFSDFNIERSPDDRSFRLLLNKFSGSGVISTEYYNYGFFSASIKLPAIYTAGIVVAFYTSNADTFEKNHDELDIEFLGNVNGQPWRFQTNMYGNGSVSRGREERYRMWFDPSKDFHQYSILWTPKNIIFYIDETPLREINRHPAMGGDFPAKPMALYATIWDASSWATNGGKAKVDYKYEPFATELKDLVLEGCIVDPSEQIPSTNCTDRNAKLLAQDYSNITPERRNNMKFFRERYMYYSYCYDNLRYPVPPPECVIVQSERDLFRDSGRLRQKMKFGGSHSHTQSHRKHRPGRSSRRRNKVAGGASKSGRRGSAAAAM

>SlXTH27

MANLLLIGVVIAMLCSEIKCSFEDNFSKSDCPDSHFKTSEDGQIWYLSLDNKAGCGFMTRQRYRFGWFSMKLKLVGGDSAGVVTAYYMCTEDGAGPTRDELDFEFLGNRTGEPYLIQTNVYKNGTGGREMRHVLWFDPTQDFHTYSILWNSHQIVFFVDKVPIRVYRNANYTNNFFPNEKPMYLFSSIWNADDWATRGGLEKTDWKNAPFVSTYMDFNVDACQWEDPFPSCVSTTTQNWWDQYNSWHLSSDQKLDYAWVQRNLVTYDYCQDIERYKVKPEECWVSPWD

>SlXTH28

MSSFMIVFLILSMLLNPGVGVNFTDVFESSWAPDHIAVVGDEVTLSLDSASGCGFESRFKYLFGKASAQIKLVEGDSAGTVIAFYMSSEGANHDELDFEFLGNVSGEPYLVQTNIYVNGSGDREQRHGLWFDPTTDFHTYSFFWNHHSIIFSVDDIPIRVFKNKEKKGVPYPKNQGMGIYGSLWNADDWATQGGRVKTNWSHSPFVTTFRSFEIDACDLCGEDTIAAGAKCGKLAKFLWDKPSKNGLEKSKKRQFKMVQNKYLVYDYCKDTARFNQMPKECLY

>SlXTH29

MAKIIHFNSLVLMIIATITFQSYLANGWTSSSMYVNWGAHHCKLLGDDLQLVLDKSAGSGAQSKNSFLFGSFEMLLKLVPNNSAGTVTTYYLSSTGTKHDEIDFEFLGNISGHPYIIHTNIYTQGVGNREQQFYPWFDPTAAFHNYTIHWNPNAVVWYIDSIPIRVFRNYQSKGISFPNQQGMGVYTSLWNADDWATRGGLVKIDWTNAPFIATYRNFRPRACYWNGPMSISQCAIPTNSNWWASPSYYKLSANKVGEMISIRSKNMIYDYCKDVKRFKGVMPIECSLPQY

>SlXTH30

MGFHLISLSALLLLTRVFEGLALPFDKKYNISWGNNNVKLLKNGEEIQLSLDKFSGCGIESKQSYGSGSFKMRIKLPSKDSAGVVTTFYLHSHTSHHDELDFEFLGNRKGKPYILQTNVFANGIGDREERIQLWFDPTTNFHEYSILWNSHHIVFFVDEIPIRVYKNKSYRGIGYPTQPMQSEATIWNGESWATENGSQKINWSNSPFIAQFQGFNIEGCPSNYHSLNCNSTKWWWNSKKLWKLTLDQEKSYKDIRSKNMIYDYCKDTNRFQNIPLECSSDY

>SlXTH31

MASFEFMSIIICILMYFALSPIYAMVDFNQYYNPLWGQNHITYLNQSTEVQLLLDQSGGAGFKSKTQYNSGLFTLRIKMSDKKTDGMITAFYLISDDQDARVNHDEIDFEFIGTQGKLQTNIFANDMGGREQVFQLPFDPSQDFHTYQILYTPQRIVFFVDNIPIRTFENNTNRGINYPTKSLWSEASLWISDAVGWAGSVEWGYAPFIVSFQDFNISGCPAGSDCLPSTDFSPWTRHKLASRSLNLMRNFRKKYMTYDYCSSEENKNRYPECA

>SlXTH32

MASLVLCLVILAFCSLHYSLASNNFNQDFDVTWGDGRAKVLNNGKLLTLSLDKVSGSGVKSKKEYLFGRIDMQLKLVRGNSAGTVTTYYLSSQGSTHDEIDFEFLGNLSGDPYIVHTNVYTQGKGDKEQQFYLWFDPTADFHTYSILWNPQTIIFYVDGTPIRVFKNMESSGVPYPNKQPMRVYASLWNADDWATRGGLVKTNWSNAPFIAYFRNFKDNNACIWEFGKSSCTNSTKSWFYHELDSTSQARLQWVQKNYMVYNYCNDINRFPRGLPLECAFNSTTN

>SlXTH33

MGFKWTMMLVLCVLIGGSMGAKPNKPIDVPFGRNYEPSWAFDHIKYLNGGSEIQLSLDNRTGTGFQSKGSYLFGHFSMHIKMVAGDSAGTVTAFYLSSQNSEHDEIDFEFLGNKTGEPYILQTNVYTGGKGDKEQRIYLWFDPTKDYHTYSVLWNLHQIVFFVDEYPIRVFKNNKNLGVKFPFDQSMKIYSSLWEADDWATRGGLEKIDWSNAPFVASYKGFHIDGCESSVNAKFCANQGKSWWDQKEFQDLDKTQWRLLRRVRDKYTIYNYCTDKKRFSTTPIECKRNRDVPRNSRKEN

>SlXTH34

MNYFSRFIFLATYFIYLSHIALASIVSTGDYNKDFYVTYSPNHINTSADGRTRSLIFDKESGTEIASKDMYLFGQFDMKIKLIPGNSAGTVVAFYLASGQPNRDEIDFEFLGNVDGKRYTLQTNVYVDGFDDREQRINLWFDPTQDYHTYSILWNLHQIVFMVDWVPIRTYRNHADKGAKYPHWQPMELKMSLWNGEDWATDGGKTKIDWSKSPFVATLGSYKIDACVWKGNARFCRVENENHWWNKGQSSTLTWTQRRLFKWVRKYHLTYDYCMDNKRFQNNMPIECSLPKY

>SlXTH35

MASSSKLVLVMCFMISAFGIAIGAKFDQEFDITWGDGRAKILNNGDLLTLSLDKISGSGFQSKNEYLFGKIDMQLKLVPGNSAGTVTAYYLSSQGPTHDEIDFEFLGNLSGDPYTLHTNVFSQGKGNREQQFHLWFDPTADFHTYSITWNPQRIIFYVDGTPIREYKNSESIGVSYPKNQPMRIYSSLWNADDWATRGGLVKTDWSQAPFSASYRNFSANACIPTSSSSCSSNSAASTSNSWLNEELDNTSQERLKWVQKNYMVYNYCTDSKRFPQGFPADCVQNN

>SlXTH36

MVNFQAILVFISFFFFVNQCLSANEVPFYQNYYQKYGGDHLTVTDQGKQVCLTIDQYTGSGFMSNQHFGSGDFSIDLKIPNKNSTGVITTFYVRTFFFLYKTIYELHNLIDSSSKLYAIYECYRLMSEHLQLTSLPMNGDPGMHHDEIDFEFLGGDGIYTLNTNIFANDGGSREQQFNLDFDPTEDFHTYRILWNQHHIIFYADNVPIRVFKNNTNYGVNFPTHKMHIEATIWNDTNWVGEVDWSQGPFKAYYRNFTINGCQYQESNRQECYNNNYYWNTITSLSPNEVQEFETVKAEQMIFSYCMRNNSRNFPECILN

>SlXTH37

MASSSSKLVLVMCFMISAFGIAIGAKFDQEFDITWGDGRAKILNNGDLLTLSLDKISGSGFQSKNEYLFGKIDMQLKLVPRNSAGTVTAYYLSSQGPTHDEIDFEFLGNLSGDPYTLHTNVFSQGKGNREQQFHLWFDPTADFHTYAITWNPQRIIFYVDGTPIREYKNSESIGVSYPKNQPMRIYSSLWNADDWATRGGLVKTDWSQAPFSASYRNFSANACIPTSSSSCSSISATSTSNSWLNEELDNTSQERLKWVQKNYMVYDYCTDSKRFPQGFPADCVQNI

>NtXTH1

MNNFSTLIFFVTAFIYLFHITLASIVSTGDFNKDFIVPWSPNHVNTSADGHTRSLIFDKESGSGIASNDTYLFGQFDMKIKLIPGNSAGTVVAFYLTSYQPNRDEVDFEFLGNVPGKPYTLQTNVYVDGLDDREQRINLWFDPTQDFHTYSILWNLHQIVFMVDRVPIRTYRNHADKGAKYPRWQPMALQISIWNGESWATDGGKTKIDWSKAPFVASLGNYTIDACVWKGNARFCRGESENNWWNKEKFSTLTWTQRRLFKWVRKYHLTYDYCMDNQRFQNNLPIECSLPKY

>NtXTH2

MKLKLVGGDSAGVVTAYYMCTEDGAGPTRDEVDFEFLGNRTGEPYLIQTNVYKNGTGGREMRHVLWFDPTEDFHSYSLLWNSHQLVFFVDEVPIRVYKNANYTNNFFPNEKPMYLFSSIWNADDWATRGGLEKTDWKNAPFVSTYKDFSVDGCQWEDPFPTCVSTTTKNWWDQYNSWHLSSDQKLNYAWVQRNLVIYDYCQDTKRYPEKPEECWLSPWD

>NtXTH3

MANLLLIAVLIAIYCSLSQAEVKGSFDDNFSKSCPESHFKTSEDGQIWYLSLDHKAGCGFMTRQKYRFGWFSMKLKLVGGDSAGVVTAYYMCTEDGAGPTRDEVDFEFLGNRTGEPYLIQTNVYKNGTGGREMRHVLWFDPTEDFHSYSLLWNSHQLVFFVDEVPIRVYKNTNYTNNFFPNEKPMYLFSSIWNADDWATRGGLEKTDWKNAPFVSTYKDFSVDGCQWEDPFPSCVSTTTENWWDQYNSWHLSSDQKLDYAWVQRNLVIYDYCQDTERYPEKPEECWLSPWD

>NtXTH4

MERMSSSIPKFLLIIALITVLFTLTQAEVQGSFDDNFSKSCPETHFKTSEDGQIWYLSLDKKAGCGFMTRQKYRFGWFSMKLKLVGGDSAGVVTAYYMCTEDGAGPTRDELDFEFLGNRTGEPYTIQTNVYKNGTGNREMRHILWFDPTEDFHTYSILWNTHQIVFFVDRVPIRVYKNANYTNNFFPNEKPMYLFSSIWNADDWATRGGLEKTNWKNQPFVSSYKDFSVDGCQWKDPFPACVSTTTKNWWDQYNSWHLSSDQKMDYAWVQRNLVTYDYCQDTERFPKKPEECWLNPWD

>NtXTH5

MEKMASSIPKILLIIALITVLFSLTQAEVQGSFDDNFSKSCPETHFKTSEDGQIWYLSLDKKAGCGFMTKQKYRFGWFSMKLKLVGGDSAGVVTAYYMCTEDGAGPTRDELDFEFLGNRTGEPYTIQTNVYKNGTGNREMRHILWFDPTEDFHTYSILWNTHQIVFFVDRVPIRVYKNANYTNNFFPNEKPMYLFSSIWNADDWATRGGLEKTNWKNQPFVSSYKDFSVDGCQWKDPFPACVSTTTKNWWDQYNSWHLSSDQKMDYAWVQRNLVTYDYCQDTERFPKKPEECWLNPWE

>NtXTH6

MERNASSMADLFFTAALMAALFSSSHAELIKGAFENNFSKSCPGTHFKTSQDGQIWYLTLDQISDCGFITKQSYRFGWFSTKLKLVGGDSAGVVTAFYMCSEVEAGPLRDEIDFEFLGNRTGQPYLIQTNVYNNGSGGREMRHLLWFDPTQDFHTYSILWNSHQIVFFVDKVPIRVYKNANHTNNFFPAERPMYVFSSIWNADNWATRGGLDKINWTSAPFIASYKDFILDACQWKDPFPACVSTTTQHWWDQYNAWHLSSKQKIDYAWVQRNFVVYDYCQDSVRNRYKPQECWLSALD

>NtXTH7

MERNMGDLLLFAALVATLFSSSHAQLIKGAFENTFSKSCPGTHFKTSQDGQIWYLTLDQVSDCGFITKQSYRFGWFSTKLKLVGGDSAGVVTAFYMCSEVEAGPLRDEIDFEFLGNRTGQPYLIQTNVYNNGSGGREMRHLLWFDPTQDFHTYSILWNSHQIVFFVDKVPIRVYKNANHTNNFFPAERPMYVFSSIWNADNWATRGGLDKINWTSAPFVASYKEFTLDACQWKDPFPACVSTTTQHWWDQYNAWHLSSKQKIDYTWVQRNFVVYDYCQDSVRNRYKPQECWLSPLD

>NtXTH8

MKQVIEYRCLLILGCGFASKSKYLFGRVSMKIKLVPGDSAGTVTAFYMNSDTDNVRDELDFEFLGNRSGQPYTVQTNVYVHGKGDKEQRINLWFDPSADFHTYTILWNHHHTVFYVDAVPIRVYKNNEAKGIPFPKFQPMGVYSTLWEADDWATRGGLEKINWSKSPFYAYYKDFDIEGCAMPGPANCASNPRNWWEGANYQQLSAVEARQYRWVRTNHMIYDYCTDKSRNPVPPPECVAGI

>NtXTH9

MISSSLKYSTVIPILLYALTFSSSVSARPATFLQDFKVAWADSHIKQIDGGKAIQLILDQNSGCGFASKSKYLFGRVSMKIKLVPGDSAGTVTAFYMNSDTDNVRDELDFEFLGNRSGQPYTVQTNVYVHGKGDKEQRINLWFDPSADFHTYTILWNHHHTVFYVDAVPIRVYKNNEAKGIPFPKFQPMGVYSTLWEADDWATRGGLEKINWSRSPFYAYYKDFDIEGCAMPGPANCASNPRNWWEGANYQQLSAVEAKQYRWVRMNHMIYDYCTDKSRNPVTPPECVAGI

>NtXTH10

MGKLTSLKYSAAILILLYALTFSFSVSARPATFLQDFKVSWAYSHIKQIDGGRAIQLILDQNSGCGFASKSKYLFGRVSMKIKLVPGDSAGTVTAFYMNSDTDNVRDELDFEFLGNRSGQPYTVQTNVYVHGKGDKEQRVNLWFDPSADFHTYTILWNHHHAVFYVDAVPIRVYKNNEAKGIPFPKFQPMGVYSTLWEADDWATRGGLEKINWSKSPFYAYYKDFDIEGCAMPGPANCASNPRNWWEGANYQQLSAVEARQYRWVRMNHMIYDYCTDKSRNPVTPPECVAGI

>NtXTH11

MARLTSLKYSAAILILLYALTFSFSVSARPATFLQDFKVSWSDSHIKQIDGGRAIQLILDQNSGCGFASKSKYLFGRVSMKIKLVPGDSAGTVTAFYMNSDTDNVRDELDFEFLGNRSGQPYTVQTNVYVHGKGDKEQRVNLWFDPSADFHTYTILWNHHHAVFYVDAVPIRVYKNNEAKGIPFPKFQPMGVYSTLWEADDWATRGGLEKINWSKSPFYAYYKDFDIEGCAMPGPANCASNPRNWWEGANYQQLSAAEARQYRWVRMNHMIYDYCTDKSRNPVTPPECVAGI

>NtXTH12

MVSFPMEFKWVFLGISLMLVGLVSSSRFEELYQPSWATDHLTNEGEILRMKLDNLSGAGFSSKNKYMFGKVTVQIKLVEGDSAGTVTAFYMSSEGPTHNEFDFEFLGNTTGEPYSVQTNVYVNGVGNREQRLNLWFDPSNEFHSYSILWNQHRVVFLVDETPVRVHSNLEHKGIPFPKDQAMGVYSSIWNADDWATQGGRVKTDWSHAPFIASYRGFEIDGCECPATVAAAENSKRCSSSAEKRYWWDEPTMSELSLHQSHQLIWVRANHMVYDYCTDTARFPVAPVECQHHQHKTRN

>NtXTH13

MVSFPMEFKCVFLGISLIMVGLVSSSRFEELYQPSWATDHLTNEGEILRMKLDNLSGAGFSSKNKYMFGKVTVQIKLVEGDSAGTVTAFYMSSEGPTHNEFDFEFLGNTTGEPYSVQTNVYVNGVGNREQRLNLWFDPSKEFHSYSILWNQRRVVFLVDDTPIRVHSNLEHKGIPFPKDQAMGVYSSIWNADDWATQGGRVKTDWSHAPFIASYRGFEIDGCECPATVAAAENSKRCSSSAVKRYWWDEPVMSELSLHQSHQLIWVRANHMVYDYCTDTARFPVAPVECQHHQHKFHN

>NtXTH14

MPSSMIVFLILAMLLNTGVGVNFAEVFESSWAPDHITVVGDQVMLTLDNASGCGFQSKNKYLFGKASVQIKLVGGDSAGTVIAFYMSSEGANHDELDFEFLGNVSGEPYLVQTNVYANGTGDREQRHSLWFDPTTDFHTYSFFWNHHTIIFSVDDIPIRVFQNKENKGVAYPKNQGMGIYGSLWNADDWATQGGRVKTNWSHSPFVATFRAFEIDACDLSGEDTVAAGAKCGKLAECWWDKPAVKQLNKSKKRQFKMVQSKHLVYDYCKDTARFTQMPKECLD

>NtXTH15

MRRKSCMLTTVPWLPLKHSLARWVGVNFTEVFESSWSPDHITVVGDQVMLTLDNASGCGFQSKNKYMFGKASAQIKLVDGDSAGTVIAFYMSSEGANHDELDFEFLGNVSGEPYLVQTNVYANGTGDREQRHSLWFDPTADFHTYSFFWNHHTIIFSVDDIPIRVFKNTEKKGVAYPKNQGMGVYGSLWNADDWATQGGRVKTNWSHSPFVATFRAFEIDACDLSGEDTVAAGAKCGKLAQCWWDKPAMRELNKSKKRQFKMVQSKHLVYDYCKDTARFTQMPKECLD

>NtXTH16

MGMNMLLVCVLFVVGAMAAAPKKPMDVPFGRNYENTWAPDHVKYFNGGSEIQLFLDNRTGTGFQSKGSYLFGHFAMHIKMVAGDSAGTVTAFYLSSQNNEHDEIDFEFLGNKTGEPYVVQTNIYTGGKGDKEQRIYLWFDPTKDYHTYSVLWNLHQIVFFVDEYPIRTFKNSKDLGVKFPFDQPMKIYSSLWEADDWATRGGLEKIDWSNAPFVASYKGFHIDGCEASVNAKLCANQGKKWWDQKEFQDLDKQQWRLLRRVRDKYTIYNYCTDKKRFATLPKECRRNRDVPRKSSKKSP

>NtXTH17

MGFKWMNMLLFCALFVVGAMAAAPKKPMDVPFGRNYENSWAPDHVKYFNGGSEIQLFLDNRTGTGFQSKGSYLFGHFAMHIKMVAGDSAGTVTAFYLSSQNNEHDEIDFEFLGNKTGEPYVVQTNVYTGGKGDKEQRIYLWFDPTKDYHTYSVLWNLHQIVFFVDEYPIRTFKNSKDLGVKFPFDQPMKIYSSLWEADDWATRGGLEKIDWSNAPFVASYKGFHIDGCEASVNAKYCSNQGKKWWDQKEFQDLDKQQWRLLRRVRDKYTIYNYCTDKKRFATMPKECRRNRDVPRKSSKKSP

>NtXTH18

MGLKGLLFSIVLINLSLLGLCGYPRKPVDVPFWKNYEPSWASHHIKYLNGGSTADLVLDRSSGAGFQSKKSYLFGHFSMKLRLVGGDSAGVVTAFYLSSNNAEHDEIDFEFLGNRTGQPYILQTNVFTGGKGDREQRIYLWFDPTKGYHSYSVLWNTFQIVIFVDDVPIRAFKNSKDLGVKFPFNQPMKIYSSLWDADDWATRGGLEKTDWSNAPFTASYTSFHVDGCEAATPQEVQVCNTKGMRWWDQKAFQDLDALQYRRLRWVRQKYTIYNYCTDRKRYPTLPPECTKDRDI

>NtXTH19

MGVKGLLFSIVLINLSLLGLCGYPRKPVDVPFWKNYEPSWASHHIKYLSGGSTVDLVLDRSSGAGFQSKKSYLFGHFSMKLKLVGGDSAGVVTAFYLSSNNAEHDEIDFEFLGNRTGQPYILQTNVFTGGKGDREQRIYLWFDPTKGYHSYSVLWNTFQIVIFVDDVPIRAFKNSKDLGVKFPFNQPMKIYSSLWDADDWATRGGLEKTDWSNAPFTASYTSFHVDGCEAATPQEVQVCNTKGMRWWDQKAFQDLDALQYRRLRWVRQKYTIYNYCTDRKRYPTLPPECTKDRDI

>NtXTH20

MQLKLVPGNSAGTVTTFFLSSQGAGHDEIDFEFLGNVSGQPYTVHTNVYSQGKGNKEQQFHLWFDPTAAFHTYSIIWNAQKIIFLVDNSPIRVYNNHESAGIPFPKSQPMKVYCSLWNADEWATQGGRVKTDWTHAPFTAYYRNFNIDGCAVTSGASSCKSTDSANNARPWQNQELDAKGRNRLRWVQSRHMVYNYCADSKRFPQGFSHECKRSRFL

>NtXTH21

MSPRFSFKMLILPIVMASLWAAASAGNFYNLADITWGEGRGKITEGGRGLSLSLDKLSGSGFQSKNEYLFGRFDMQLKLVPGNSAGTVTTFFLSSQGAGHDEIDFEFLGNVSGQPYTVHTNVYSQGKGNKEQQFHLWFDPTAAFHTYSIIWNAQKIIFLVDNSPIRVYNNHESNGIPFPKIQPMKVYCSLWNADEWATQGGRVKTDWTHVPFTAYYRNFNIDGCAVTSGTSSCKSTDSANNARPWQNQELDAKGRNRLRWVQSRHMVYNYCADSKRFPQGFSHECKRSRFL

>NtXTH22

MASHLFLISILMGSLVAASANFNNLAEITWGEGRGKITEGGKGLSLSLDKLSGSGFQSKNEYLFGRFDMQLKLVPGNSAGTVTTFFLSSQGEGHDEIDFEFLGNTTGEPYTVHTNVYSQGKGNKEQQFHLWFDPTAAFHTYTIVWNSNRIVFLVDNIPIRVYNNHENNGIPFPKSQPMKVYCSLWNADEWATQGGRVKTDWTHAPFTAYYRNFKIDGCAVTSGASSCKSTDSAGNAKAWQNQELDAKGRNRVRWVQSRHMVYNYCADKKRFPQGYSHECKSSRF

>NtXTH23

MASHFLLISILMGSLVVASANFNNLAEITWGEGRGKITEGGKGLSLSLDKLSGSGFQSKNEYLFGRFDMQLKLVPGNSAGTVTTFFLSSQGKGHDEIDFEFLGNTTGEPYTVHTNVYSQGKGNKEQQFHLWFDPTAAFHTYTIVWNANRILFLVDNIPIRVYNNHESNGIPFPKSQPMKVYCSLWNADEWATQGGRVKTDWTHAPFTAYYRNFKIDGCAVTSGASSCKSTDSAGNAKAWQNHELDAKGRNRVRWVQSRHMVYNYCADKKRFPQGYSHECKSSRF

>NtXTH24

MASKFSSVMLLLCIIMSIQLLAASAGNFYRDAVITWGEGRGKIQEGGRGLALTLDKLSGSGFQSKNEYLFGRFDMQLKLVPGNSAGTVTTFFLSSQGEGHDEIDFEFLGNVSGQPYTVHTNVYTQGKGNKEQQFHLWFDPTAAFHTYTIVWNPHRIVFLVDNSPIRVYNNHESIGIPFPKSQAMRVYCSLWNADEWATQGGRVKTDWTLAPFTAYYRNINIDGCAVLSGTSSCKSSNSANNAKPWQTHELDGKGRNRLRWVQSRHMVYNYCADSKRFPQGFSAECKSSRF

>NtXTH25

MASKFSSAMLLLCILMSIQLLAASAGNFYRDTVITWGEGRGKIQEGGRGLALTLDKLSGSGFQSKNEYLFGRFDMQLKLVPGNSAGTVTTFFLSSQGEGHDEIDFEFLGNVSGQPYTVHTNVYTQGKGNKEQQFHLWFDPTAAFHTYTIVWNPHRIVFLVDNSPIRVYNNHENIGIPFPKSQAMRVYCSLWNADEWATQGGRVKTDWTLAPFTAYYRNINIDGCAVLSGTSSCKSSNSANNAKPWQTHELDGKGRNRLRWVQSRHMVYNYCADSKRFPQGFSEECKRSRF

>NtXTH26

MSLSSASSRIPKMFLQLSVLAVFLLCTACADNFYQDATVTWGDQRAHIQEGGRLLTLSLDKISGSGFQSKSEFLFGRFDMQLKLIPGNSAGTVTTFYLSSQGAGHDEIDFEFLGNSSGQPYTVHTNVYSQGKGNKEQQFHLWFDPTTSFHTYSIIWNAQRIIFLVDNIPIRVYNNHEALGVAFPKNQAMRVYASLWNADDWATQGGRVKTDWSMAPFTASYRNFNTNACVWSAASSTSSCGGSKSTDSANNDQTWQTQELDANGRNRLRWVQQKYMTYNYCTDAQRFNQVIPPECKRSRF

>NtXTH27

MGSRIFLVLALVFSSCMVSYGGNFFQEFDFTWGGNRAKIFNGGQLMSLSLDKVSGSGFQSKKEYLFGRIDMQIKLVAGNSAGTVTTYYLSSQGPTHDEIDFEFLGNVTGEPYILHTNIYAQGKGNKEQQFYLWFDPTKNFHTYSIIWKPQHIIFLVDNTPIRVYKNAESIGVPFPKNQPMRIYSSLWNADDWATRGGLVKTDWSKAPFTAYYRNFNSQTFSSSQFSNEKWQNQELDANGRRRLRWVQRNFMIYNYCTDFKRFPQGFPPECKRF

>NtXTH28

MARFSSSSSRSRSSLPYIVLLFVAALFVFKIDVIISQTFSSARRNLENTPNRILVKSKSQETDDSIPVVLVNGTFHRHFILSWGDDRGKIHENGELLTLSLDKQSGSGFQSKKEYLFAKIDMQIKLVPGNSAGTVTTFYLSSQGNKHDEIDFEFLGNSTGNPYTLHTNIFSLGQGNREQQFFLWFDPTADYHTYSILWNPKCIIFYVDGTPIREFKNAEKIGVPFLKYQPMRLYSSLWNADDWATQGGRVKTNWKLAPFIASYKNFTYEACIYSRLTSSSSCNINSPPFGNNAWLTHELDRRSRAKMKILQKKHMIYDYCKDKWRFPKGPAPECKLQ

>NtXTH29

MARFSSSSSRSRSSLPYIILLFVAALFVFKIDVIISQSFSSARRNLENTPNHILVKSKSQETDDSIPVVLVNGTFHRHFILSWGDDRGKIHENGELLTLSLDKLSGSGFQSKKEYLFAKIDMQIKLVPGNSAGTVTTFYLSSQGNKHDEIDFEFLGNSTGNPYTLHTNIFSLGQGNREQQFFLWFDPTADYHTYSILWNPKCIIFYVDGTPIREYKNAEKIGVPFPKYQPMRLYSSLWNADDWATQGGRIKTNWKLAPFIASYKNFTYDACIYSRLTSSSSCNINSPPFGNDSWLTHELDRRSRAKMKILQKKHMIYDYCNDKWRFPKGPAPECKLQ

>NtXTH30

MMKTSSCMFSFLFLSFLVLVALAENFNQEFDVTWGDGRVKILENGQLLTLSLDKTSGSGFRSKRQYMFGKIDMKIKLVPGNSAGTVTTYYLSSLGPTHDEIDFEFLGNLSGDPYILHTNVFVQGKGEREQQFYLWFDPTKDFHTYSILWNPRSIIFSVDGTPIRQFKNLEASRGIPYPKNQPMWIYSSLWDAEDWATRGGLVKTDWSKAPFIASYRNFNAQACVWSSGSTSSCSINSTANSWITESLDNSGQARIKWVQKNYMVYNYCTDTKRFPQGFPLECSLN

>NtXTH31

MMKTSISCIISFLFLSFLLVVMAALAGDFNQEFDVTWGDGRVKILENGQLLTLSLDKTSGSGFRSKRQYMFGKIDMKIKLVPGNSAGTVTTYYLSSLGPTHDEIDFEFLGNLSGDPYILHTNVFTQGKGDREQQFYLWFDPTKDFHTYSILWNPRSIIFSVDGTPIRQFKNLETSMGIPYPKNQPMWIYSSLWDAEDWATRGGLVKTDWSQAPFVASYRNFNAQACVWSSGSTSSCSRNSTANSWITESLDNSGQARIKWVQKNYMVYNYCTDIKRFPQGFPLECSLN

>NtXTH32

MMKSFLFQMMFLVVAFAGNFNQNFDITWGDGRAKILENGQLLTLSLDKTSGSGFRSKNQYLFGKIDLKIKLVPGNSAGTVTTYYLSSIGSSHDEIDFEFLGNLSGDPYILHTNVFTQGKGNREQQFYLWFDPTKYFHTYSILWNPQSIIFSVDGTPIRQFKNLEASGIPYPKNQPMWIYSSLWNADDWATRGGLVKTDWSKAPFIASYRNYNAQACVWSSTSSSSCSPNNSTENSWLSESLDNTGQSKIKWVQNNYMIYNYCTDTKRFPQGFPPECSLN

>NtXTH33

MMKSFLFLMIFLVVALAGNFNKDFDITWGDGRAKILENGQLLTLSLDKTSGSGFRSKNQYLFGKIDLKIKLVPGNSAGTVTTYYLSSIGSSHDEIDFEFLGNLSGDPYILHTNVFTQGKGNREQQFYLWFDPTKDFHTYTILWNPQSIIFSVDGTPIRQFKNLEASGIPYPKNQPMWIYSSLWNADDWATRGGLVKTDWSKAPFIASYRNYNAQACVWSSSSSSSCTSNSSTGNSWLSESLDSTGQSRIKWVQSNYMIYNYCTDTKRFPQGFPPECSLN

>NtXTH34

MSSFSSKLVLALIVSAFAIAIAGTIDENFEITWGEGRAKMLNNGELLTLSLDKISGSGFQSKNEYLFGKIDMQLKLVPGNSAGTVTAYYLSSQGPTHDEIDFEFLGNLSGDPYTLHTNVFSQGKGNREQQFHLWFDPTADFHTYSILWNPQRIIFYVDGTPIREYKNAESIGVSYPKKQPMRIYSSLWNADDWATRGGLIKTDWSKAPFSASYRNFKSATSTSAATSNSWLNEELDNTSQERLKWVQKNYMVYNYCNDSKRFPQGFPADCAM

>NtXTH35

MASLLAQYLVFLALCSLQYHSLAYNNFNQDFDVTWGDGRAKVLNNGKLLTLSLDKASGSGIQSKREYLFGRIDMQLKLVRGNSAGTVTTYYLSSQGATHDEIDFEFLGNLSGDPYIIHTNVYTQGKGDKEQQFYLWFDPTAGFHTYSILWNPQTIIFYVDGTPIRVFKNMKSRGIPYPNKQPMRVYASLWNADDWATRGGLIKTDWSNAPFIASFRNFKANACVWEFGKSSCNSSTNPWFFQELDSTSQAKLQWVQKNYMVYNYCTDIKRFPQGFPLECNFNSTTS

>NtXTH36

MASLLVQCLNFLALCSLQYHILASSNFNQDFDVTWGDGRAKVLNNGKLLTLSLDKASGSGIQSKREYLFGRIDMQLKLVRENSAGTVTTYYLSSQGATHDEIDFEFLGNLSGDPYIIHTNVYTQGKGDKEQQFYLWFDPTAGFHTYSILWNPQTIIFYVDGTPIRVFKNMKSSGVPYPTNQPMRVYASLWNADDWATRGGLIKTDWSKAPFIASFRNFKANACVWEFGKSSCNSSTNSTKPWFFQELDSTSQARLQWVQKNYMVYNYCTDIKRFPQGLPQECNFNSTTS

>NtXTH37

MAKFIAFNSLVLIIATFAFHCAIVNAKISSSMYINWGAHHCQMLGDDLQLVLDKSAGSGAQSKRTFLFGSFEMLIKLVPNNSAGTVTTYYLSSTGTKHDEIGFEFLGNVSGQPYIIHTNIYTQGVGNKEQQFYPWFDPTADFHNYTIHWNPNAVVWYIDGIPIRVFRNYQLKGIPFPNQQGMRIYSSLWNADEWATRGGRDKIDWTNAPFIATYRKFRPRACYWNGPLSIVQCAIPTKSNWWNFPLYSKLSAPKVDQMNSIRSKYMIYDYCKDTTRFKGVMPTECTLPQN

>NtXTH38

MAKFIAFNSLVLIIATIAFHCAIVNGKISSSMYVNWGAHHCQMLGDDLQLVLDKSAGSGAQSKRTFLFGSFEMLIKLVPNNSAGTVTTYYLSSTGTKHDEIDFEFLGNVSGQPYILHTNIYTQGVGNREQQFYPWFDPTADFHNYTIHWNPNAVVWYVDGIPIRVFRNYQFKGIPYPNQQGMRIYSSLWNADEWATRGGRDKIDWTNAPFIATYRKFRPRACYWNGPLSIVQCAIPTKSNWWNSPLYSKLSAPKVDQMNSIRSKYMIYDYCKDTTRFKGVMPIECSLPQY

>NtXTH39

MAKFVAFNSLVLIIATIAFHCAIVNGKISSSMYVNWGAHHCQMLGEDLQLVLDKSAGSGAQSKRTFLFGSFEMLIKLVPNNSAGTVTTYYLSSTGTKHDEIDFEFLGNVSGQPYILHTNIYTQGVGNREQQFYPWFDPTADFHNYTIHWNPNAVVWYVDSIPIRVFRNYQLKGIPFPNQQGMRIYSSLWNADEWATRGGRDKIDWTNAPFIAKYRKFRPRACYWNGPLSIVQCAIPTKSNWWNSPLYSKLSAPKVDQMNSIRSKYMIYDYCKDTTRFKGVTPTECSLPQN

>NtXTH40

MAKFITFSLVLIIATFAFRCTLVNGKISSSMYINWGAHHCKMQGDDLQLVLDKSAGSGAQSKRTFLFGSFEMLIKLVPNNSAGTVTTYYLSSTGTKHDEIDFEFLGNVSGQPYIIHTNIYTQGVGNKEQQFYPWFDPTADFHNYTIHWNLNAVVWYVDGIPIRVFRNYELKGIPFPNQQGMRIYSSLWNADEWATRGGRDKIDWTNAPFIATYRNFRPRACYWNGPLSIGQCAIPTKSNWWNSPLYNKLSAPKVDQMNSIRSKYMIYDYCKDTKRFKGVTPTECSLPQN

>NtXTH41

MFKIMASSRLLSLANLFILAIAFHLVSVNGMFSDNMYIGWGAHHSWMQGNDLQLVLDQSSGSGVQSKGAFLFGSIQMQIKLVPGNSAGTVTAYYLSSTGDKHDEIDFEFLGNVSGHPYIIHTNIFTQGAGGREQQFYPWFDPTADYHNYTIHWNPSAVVWYVDDIPIRVYKNYQSQGILYPNAQGMGVYSSLWNADNWATRGGLDKIDWTNAPFIAKYRNFAPRACPWYGPGSISHCAAPTPNNWYTSPEYSQLSYAKQGQMNWVRNNYMIYDYCKDTTRFNGQIPGECFKPQF

>NtXTH42

MFKIMASSRLLSLSNLFILAIAFHLVSVNGMFSDNMYINWGAHHSWMQGNDLQLVLDQSAGSGVQSKGAFLFGSIEMQIKLVPGNSAGTVTAYYLSSTGDKHDEIDFEFLGNVSGQPYIIHTNIFTQGAGGREQQFYPWFDPTADYHNYTIHWNPSAVVWYVDGIPIRVYKNYQSQGILYPNAQGMKVYSSLWNADNWATRGGLDKIDWTNAPFIAKYRNFAPRACPWYGPGSIRQCAAPTPNNWYTSYEYSQLSYAKQGQMNWVRNNYMIYDYCKDKTRFNGQIPGECFKPQI

>NtXTH43

MAIFFLHFLLLLIVVPSTNAGYWPPSPGYYPSSKFRSMSFYQGFRNLWGPNHQNVDNNGINIWLDRNSGSGFKSIKPFRSGYFGASIKLQPGYTAGVITAFYLSNNEAHPGYHDEVDIEFLGTTFGKPYTLQTNVYIRGSGDGKIVGREMKFHLWFDPTKEFHHYAILWSPREIIFLVDDVPIRRYARKSIATFPLRPMWLYGSIWDASSWATEDGKYKADYRYQPFYGKFTNFKASGCTAYSSRWCHPVSASPSRSGGLTRQQRQAMNWVHSHYLAYDYCRDSKRDHSLTPECWR

>NtXTH44

MSIFFLPFLLFLIVLPSTNAGYWPPSPGYYPSSKFKSMSFYQGFKNLWGPNHQNVDNNGINIWLDRNSGSGFKSIKPFRSGYFGASIKLQPGYTAGVITAFYLSNNEAHPGYHDEVDIEFLGTTFGKPYTLQTNVYIRGSGDGKIIGREMKFHLWFDPTKDFHHYAILWSPREIIFLVDDVPIRRYARKSIATFPLRPMWLYGSIWDASSWATEDGKYKADYRYQPFYGKFTNFKASGCTAYSSRWCHPVSASPSRSGGLTRQQRQAMNWVHSHYLAYDYCRDSKRDHSLTPECWR

>NtXTH45

MANLFLLSLLLIFLFNSSNAQGPLSPGYYPSSKVQSLGFNQGFRNLWGPQHQSLDQSALTIWLDKTSGGSGFKSLENYRSGYFGTSVKLQPGYTAGIITSFYLSNNQDYPGNHDEIDIEFLGTTPNKPYTLQTNVYIRGSGDGNIIGREMKFHLWFDPTKAYHNYAILWDPNEIIFFVDDVPIRRYPRKNDATFPQRPMYVYGSIWDASSWATEEGRIKADYRYQPFVGKYNNFKIAGCTANENPWCGRSPSSSPSRAGGLSRQQIAAMLWVQRNYKVYDYCRDPRRDHTHTPEC

>NtXTH46

MALFLLSLLLLFLFNSSNAQGPPSPGYYPSSKVQSLGFSQCFRNLWGPQHQSLDQSALTIWLDKTTGGSGFKSLKNYRSGYFGTSVKLQPGYTAGIITSFYLSNNQDYPGNHDEIDIEFLGTTPNKPYTLQTNVYIRGSGDGNIIGREMKFHLWFDPTQAYHNYAILWNPNEIIFFVDDVPIRRYPRKNDATFPQRPMYVYGSIWDASSWATEEGRIKADYRYQPFIGKYNNFKIAGCTANENPWCGRSPSSSSSRAGGLSRQQMAAMLWVQRNYKVYDYCRDPRRDHTHTPEC

>NtXTH47

MDFFHHNKTFLLSQFLIFCMIVVVSCRGPVYKPPEVEKLTDHFSRLSVNQGYNVFFGGANVRMTNNGSSADLILDKSSGSGLISKEKYYYGFFNAALKLPAHFTSGVVIAFYMSNSDVFPHNHDEIDFELLGHDKRRDWVLQTNLYGNGSVHTGREEKFYLWFDPTLDFHDYTILWNNHHIVFLVDNVPIREVVHNTAISSVYPSKPMSVIATIWDGSEWATHGGKYPVNYQYAPFVTSMKEVELEGCVRQQNTSATSTCFRRSTSSLDPVDGEEFMKLSQQQMTGLDWVRRKHMFYSYCQDTNRYKVLPPECTSN

>NtXTH48

MEFYHQHKTCLFSGFLIFCMIAVASSLGPIYTPPEAERLTDRFSRLSVNQGYNVFFGGANVRLTNNGSNADLILDKSSGSGLVSRDKYYYGFFNAALKLPANFTSGVVVAFYLSNQNIFPHNHDELDFELLGYDKRRDWVLQTNIYGNGSVSTGREEKFYLWFDPTQDFHDYSILWNNHHILFLVDNVPVREVVNNTTISSVYPSKPMSIYATIWDGSQWATRGGKYPVNYTYAPFVTSIKGVELEGCVSEQNASAASACARRSTSSLDPVDGEEFVKLSQQQMTGLDWARRKHMFYSYCQDTRRYKVLPPECTAT

>NtXTH49

MEFFHQHNTLLLSEFLIFCMISVASSLGPIYTPPEVERLTDRFSRLSVNQGYNMFFGGVNVRLTNNGSSADLILDKSSGSGLVSRDKYYYGFFNAALKLPANFTSGVVVAFYLSNQNIFPHDHDELDFELLGYDKRRDWVLQTNNYGNGSVSTGREGKFYLWFDPTQDFHDYTILWNNHHILFLVDNVPVREVVHNTAISSVYPSKPMSIYVTIWDGSQWATRRGKYPVNYTYAPFVTSIKGVELEGCVSEQNGSAATACARRSTSSLDPVDGEEFVKLSQQQMMGLDWARRKHMFYSYCQDTRRYKVLPPECTAT

>NtXTH50

MDYRVLSSLSKSLTPFSLLMLLYIFPAAETATATTAKAFNLSTITFEEGYSPLFSDFNIERSPDDTSFRLLLNRFSGSGVISTEYYNYGFFSASIKLPAIYTAGIVVAFYTSNVDTFEKNHDELDIEFLGNVNGQPWRFQTNLYGNGSVSRGREERYRMWFDPSNDFHHYSILWTPKNIIFYVDETPIREVNRNPAMGGDFPSKPMSLYATIWDASSWATNGGKAKVDYKHEPFATEFKDLVLEGCIVDPIEQISSTNCTDRIARLLSQNYSIMTPERRKSMKWFRERYMYYSYCYDNIRYPVPPPECVIVQSERDLFKDSGRLRQKMKFGGSHSHRKHRPGRSSRRRNRAAGGGSSKSGQAAAM

>NtXTH51

MDFIRKKICLSVFLFFHVWFSTALNVSTIPFSDGFSHLFGEGNILHATDDKSLQLHLNQRTGSGFKSSDLYNHGFFSAKIKLPSDYTAGIVVAFYTTNGDLFTKTHDELDFEFLGNIRGKAWRFQTNMYGNGSTSRGREERYYLWFDPSKEFHRYSILWTNKNIIFYIDDVPIREIVRNDAMGGDYPSKPMGLYATIWDASDWATSGGKYKTNYKYAPFIAEFTDLVLNGCAMDPLEQVVNNPSCDEKDDELQKADFSRITPRQRMAMKRFRSKYMYYSYCYDSLRYSVPPPECEIDPIEQQHFKETGRLKFNKHHHRHPKRTKSQVLDARNYGNQDEE

>NtXTH52

MDFIRKKICLSVFLFFHVCFITADAALNVSTIPFSDGFSHLFGEGNILHATDDKSLQLHLNQRTGSGFKSSDLYTHGFFSAKIKLPSDYTAGIVVAFYTTNGDLFTKTHDELDFEFLGNIRGKAWRFQTNMYGNGSTSRGREERYYLWFDPSKEFHRYSILWTIKNIIFYIDDVPIREIVRNDAMGGDYPSKPMGLYATIWDASDWATSGGKYKTNYKYAPFIAEFTDLVLNGCAMDPLEQVVNNPSCDEKDDELQKADFSRITPRQRMAMKRFRSKYMYYSYCYDSLRYSVPPPECEIDHVEQQHFKETGRLKFNKHGHHRHAKRTRSQVLDARNHGNQDEE

>NtXTH53

MVNYHLVTFIFFSVVELVYGSSRNLPILAFDEGYSHLFGDDNVMILKDGKSAHISLDERTGAGFVSQDLYLHGFFSASIKLPADYTAGVVVAFYMSNVDMFEKNHDEIDFEFLGNIRGKDWRIQTNIYGNGSTSVGREERYGLWFDPSEDFHHYSILWTENFIIFYVDNVPIREIKRTEAMGGDFPSKPMSLYATIWDGSGWATNGGKYKVNYKYAPYIAKFSDFVLHGCAVDPIELSSKCDTAPKTASIPTGITPDQRRKMEKFRKKQMQYSYCYDKTRYKVPPPECVIDPKEAERLRAFDPVTFGGSRHHHGKQHRRSRSRAEGDISFL

>NtXTH54

MVNYHLVIFIFFSVVELVYGSSRNLPILAFDEGYSHLFGDNNLMILKDGKSAHISLDERTGAGFVSQDLYLHGFFSASIKLPADYTAGVVVAFYMSNVDMFEKNHDEIDFEFLGNIRGKDWRIQTNIYGNGSTSFGREERYGLWFDPSEDFHHYSILWTENFIIFYVDNVPIREIKRTEAMGGDFPSKPMSLYATIWDGSGWATNGGKYKVNYKYAPYIAKFSDFVLHGCAVDPIELSSKCDTAPKTSSIPTGITPDQRRKMENFRKKQMQYSYCYDKTRYKVPPTECVIDPKEAERLRVFDPVTFGGSRHHHGKRHSRSRSRAEGDVSFL

>NtXTH55

MVNFRLEIFILCSFLVLVCGSSKQLQTLPFDEGYSQLFGHDNLMVLEDGKSVHLSLDERTGAGFVSQDLYLHGYFSASIKLPADYTAGVVVAFYMSNGDMFEKNHDEIDFEFLGNIRAKKWRIQTNIYGNGSTNVGREERYGLWFDPSEDFHQYSILWTESQIIFYVDNIPIREIKRTKAMGGDFPSKPMSLYATIWDGSSWATNGGKYKVNYKYAPYVAKFSDFILHGCAVDPIELSPKCDTTPNSASIPTSISPDQRRKMESFRKKYLQYSYCYDRTRYNVPLSECVIDPKEADRLRGFDPVTFGGVQRHHSKRHHQRQSRREDTSSE

>NtXTH56

MVNFRLGIFILCSFLVLVSGSSKKLQTLPFDEGYSQLFGHDNLMVLEDGKSVHISLDERTGAGFVSQDLYLHGYFSASIKLPADYTAGVVVAFYMSNGDMFEKSHDEIDFEFLGNIRAKNWRIQTNIYGNGSTNVGREERYGLWFDPSEDFHQYTILWTESQIIFYVDNIPIREIKRTKAMGGDFPSKPMSLYATIWDGSSWATNGGKYKVNYKYAPYVAKFSDFVLHGCAVDPIELSPKCDTAPKSAFVPTGISPDQRRKMESFRKKYLQYSYCYDRTRYNVPLSECVIDPKEADRLQGFDPVTFGGVQRHHSKRRRQRQSRREDASSE

>AtXTH33

MKIMWETAVVFCLCSLSLVSSHSRKFTTPNVTRLTDQFSKIAIENGFSRRFGAHNIQVNGSLAKLTLDKSSGAGLVSKNKYHYGFFSARLKLPAGFASGVVVAFYLSNAETYPKSHDEIDIELLGRSRRDDWTIQTNVYANGSTRTGREEKFYFWFDPTQAFHDYTLIWNSHHTVFLVDNIPVRQFPNRGAFTSAYPSKPMSLYVTVWDGSEWATKGGKYPVNYKYAPFVVSVADVELSGCSVNNGSSTGSGPCTKSGGSISSLDPVDGQDFATLSKNQINAMDWARRKLMFYSYCSDKPRYKVMPAECN

>AtXTH8

METERRIITSCSAMTALFLFMTALMASSSIAATPTQSFEDNFNIMWSENHFTTSDDGEIWNLSLDNDTGCGFQTKHMYRFGWFSMKLKLVGGDSAGVVTAYYMCSENGAGPERDEIDFEFLGNRTGQPYIIQTNVYKNGTGNREMRHSLWFDPTKDYHTYSILWNNHQLVFFVDRVPIRVYKNSDKVPNNDFFPNQKPMYLFSSIWNADDWATRGGLEKTDWKKAPFVSSYKDFAVEGCRWKDPFPACVSTTTENWWDQYDAWHLSKTQKMDYAWVQRNLVVYDYCKDSERFPTLPWECSISPWA

>AtXTH28

MGFITRFLVFMSLFTSLVSGFALQKLPLIQFDEGYTQLFGDQNLIVHRDGKSVRLTLDERTGSGFVSNDIYLHGFFSSSIKLPADYSAGVVIAFYLSNGDLYEKNHDEIDFEFLGNIRGREWRIQTNIYGNGSTHLGREERYNLWFDPTEDFHQYSILWSLSHIIFYVDNVPIREVKRTASMGGDFPAKPMSLYSTIWDGSKWATDGGKYGVNYKYAPYVSQFTDLILHGCAVDPTEKFPSCKDEAVQNLRLASEITESQRNKMEIFRQKHMTYSYCYDHMRYKVVLSECVVNPAEAKRLRVYDPVTFGGIPHGHRRGKHRSRSRLARTESI

>AtXTH30

MSKSSYNHIFILILCLCLRSSSAFTNLNTLSFEESLSPLFGDANLVRSPDDLSVRLLLDRYTGSGFISSNMYQHGFYSSMIKLPADYTAGVVVAFYTSNGDVFEKTHDELDIEFLGNIKGKPWRFQTNLYGNGSTHRGREERYRLWFDPSKEFHRYSILWTPHKIIFWVDDVPIREVIRNDAMGADYPAKPMALYATIWDASDWATSGGKYKANYKFAPFVAEFKSFSLDGCSVDPIQEVPMDCSDSVDFLESQDYSSINSHQRAAMRRFRQRFMYYSYCYDTLRYPEPLPECVIVPAEKDRFKETGRLKFGGTEARERRRNRRQQRRPEIEIESDPDDRKLL

>AtXTH17

MKLSCGTSFAFLLLFLLAAQSVHVYAGSFHKDVQIHWGDGRGKIHDRDGKLLSLSLDKSSGSGFQSNQEFLYGKAEVQMKLVPGNSAGTVTTFYLKSPGTTWDEIDFEFLGNISGHPYTLHTNVYTKGTGDKEQQFHLWFDPTVNFHTYCITWNPQRIIFTVDGIPIREFKNPEAIGVPFPTRQPMRLYASLWEAEHWATRGGLEKTDWSKAPFTAFYRNYNVDGCVWANGKSSCSANSPWFTQKLDSNGQTRMKGVQSKYMIYNYCTDKRRFPRGVPAECT

>AtXTH27

METLSRLLVFMSLFSGLVSGFALQNLPITSFEESYTQLFGDKNLFVHQDGKSVRLTLDERTGSGFVSNDYYLHGFFSASIKLPSDYTAGVVVAFYMSNGDMYEKNHDEIDFEFLGNIREKEWRVQTNIYGNGSTHSGREERYNLWFDPTEDFHQYSILWSDSHIIFFVDNVPIREVKRTAEMGGHFPSKPMSLYTTIWDGSKWATNGGKYGVNYKYAPYIARFSDLVLHGCPVDPIEQFPRCDEGAAEDMRAAQEITPSQRSKMDVFRRRLMTYSYCYDRARYNVALSECVVNPAEAQRLRVYDPVRFGGIPRRHRNGKHRSKRSRVDGTESI

>AtXTH4

MTVSSSPWALMALFLMVSSTMVMAIPPRKAIDVPFGRNYVPTWAFDHQKQFNGGSELQLILDKYTGTGFQSKGSYLFGHFSMHIKLPAGDTAGVVTAFYLSSTNNEHDEIDFEFLGNRTGQPAILQTNVFTGGKGNREQRIYLWFDPSKAYHTYSILWNMYQIVFFVDNIPIRTFKNAKDLGVRFPFNQPMKLYSSLWNADDWATRGGLEKTNWANAPFVASYKGFHIDGCQASVEAKYCATQGRMWWDQKEFRDLDAEQWRRLKWVRMKWTIYNYCTDRTRFPVMPAECKRDRDA

>AtXTH10

MTLINRSKPFVLLVGFSIISSLLLWVSQASVVSSGDFNKDFFVTWSPTHVNTSNDGRSRTLKLDQESGASFSSIQTFLFGQIDMKIKLIRGSSQGTVVAYYMSSDQPNRDEIDFEFLGNVNGQPYILQTNVYAEGLDNREERIHLWFDPAKDFHTYSILWNIHQIVFMVDQIPIRLYRNHGEKGVAYPRLQPMSVQASLWNGESWATRGGHDKIDWSKGPFVASFGDYKIDACIWIGNTSFCNGESTENWWNKNEFSSLTRVQKRWFKWVRKYHLIYDYCQDYGRFNNKLPKECSLPKY

>AtXTH21

MVSSTLLVMSISLFLGLSILLVVHGKDFNQDIDITWGDGRGNILNNGTLLNLGLDQSSGSGFQSKAEYLYGKVDMQIKLVPGNSAGTVTTFYLKSQGLTWDEIDFEFLGNVSGDPYIVHTNVYTQGKGDREQQFYLWFDPTAAFHNYSILWNPSHIVFYIDGKPIREFKNLEVLGVAYPKNQPMRMYGSLWNADDWATRGGLVKTNWSQGPFVASFMNYNSENACVWSIVNGTTTTSPCSPGDSTSSSSSSTSEWFSQRGMDSSSKKVLRWVQRKFMVYNYCKDKKRFSNGLPVECTAKNKNTKS

>AtXTH32

MGNSLISLLSIFHLLVLWGSSVNAYWPPSPGYWPSSKVGSLNFYKGFRNLWGPQHQRMDQNALTIWLDRTSGSGFKSVKPFRSGYFGANIKLQPGYTAGVITSLYLSNNEAHPGFHDEVDIEFLGTTFGKPYTLQTNVYIRGSGDGKIIGREMKFRLWFDPTKDFHHYAILWSPREIIFLVDDIPIRRYPKKSASTFPLRPMWLYGSIWDASSWATEDGKYKADYKYQPFTAKYTNFKALGCTAYSSARCYPLSASPYRSGGLTRQQHQAMRWVQTHSMVYNYCKDYKRDHSLTPECWR

>AtXTH16

MGRILNRTVLMTLLVVTMAGTAFSGSFNEEFDLTWGEHRGKIFSGGKMLSLSLDRVSGSGFKSKKEYLFGRIDMQLKLVAGNSAGTVTAYYLSSEGPTHDEIDFEFLGNETGKPYVLHTNVFAQGKGNREQQFYLWFDPTKNFHTYSLVWRPQHIIFMVDNVPIRVFNNAEQLGVPFPKNQPMKIYSSLWNADDWATRGGLVKTDWSKAPFTAYYRGFNAAACTVSSGSSFCDPKFKSSFTNGESQVANELNAYGRRRLRWVQKYFMIYDYCSDLKRFPQGFPPECRKSRV

>AtXTH3

MDYMRIFSVFVVTLWIIRVDARVFGGRGIEKFVTFGQNYIVTWGQSHVSTLHSGEEVDLYMDQSSGGGFESKDAYGSGLFEMRIKVPSGNTGGIVTAFYLTSKGGGHDEIDFEFLGNNNGKPVTLQTNLFLNGEGNREERFLLWFNPTKHYHTYGLLWNPYQIVFYVDNIPIRVYKNENGVSYPSKPMQVEASLWNGDDWATDGGRTKVNWSYSPFIAHFRDFALSGCNIDGRSNNVGACESSNYWWNAGNYQRLSGNEQKLYEHVRSKYMNYDYCTDRSKYQTPPRECY

>AtXTH31

MALSLIFLALLVLCPSSGHSQRSPSPGYYPSSRVPTSPFDREFRTLWGSQHQRREQDVVTLWLDKSTGSGFKSLRPYRSGYFGASIKLQPGFTAGVDTSLYLSNNQEHPGDHDEVDIEFLGTTPGKPYSLQTNVFVRGSGDRNVIGREMKFTLWFDPTQDFHHYAILWNPNQIVFFVDDVPIRTYNRKNEAIFPTRPMWVYGSIWDASDWATENGRIKADYRYQPFVAKYKNFKLAGCTADSSSSCRPPSPAPMRNRGLSRQQMAALTWAQRNFLVYNYCHDPKRDHTQTPEC

>AtXTH11

MRGSDQKILLMVMVVVAVVAAAQGQEETTGFVTWGNNYYQTWGHQALVINKTSELQLTLDKNSGSGFESQLIYGSGYFNVRIKAPQTTSTGVITSFYLISRSSRHDELCFQILGKNGPPYLLNTNMYLYGEGGKDQRFRLWFDPTKDYHSYSFLWNPNQLVFYVDDTPIRVYSKNPDVYYPSVQTMFLMGSVQNGSIIDPKQMPYIAKFQASKIEGCKTEFMGIDKCTDPKFWWNRKQLSSKEKTLYLNARKTYLDYDYCSDRQRYPKVPQECGSYT

>AtXTH9

MVGMDLFKCVMMIMVLVVSCGEAVSGAKFDELYRSSWAMDHCVNEGEVTKLKLDNYSGAGFESRSKYLFGKVSIQIKLVEGDSAGTVTAFYMSSDGPNHNEFDFEFLGNTTGEPYIVQTNIYVNGVGNREQRLNLWFDPTTEFHTYSILWSKRSVVFMVDETPIRVQKNLEEKGIPFAKDQAMGVYSSIWNADDWATQGGLVKTDWSHAPFVASYKEFQIDACEIPTTTDLSKCNGDQKFWWDEPTVSELSLHQNHQLIWVRANHMIYDYCFDATRFPVTPLECQHHRHL

>AtXTH1

MEYLSIFGFVSVLYLIIRVDARAYEVNGIDQSKVGFDDNYVVTWGQNNVLKLNQGKEVQLSLDHSSGSGFESKNHYESGFFQIRIKVPPKDTSGVVTAFYLTSKGNTHDEVDFEFLGNKEGKLAVQTNVFTNGKGNREQKLALWFDPSKDFHTYAILWNPYQIVLYVDNIPVRVFKNTTSQGMNYPSKPMQVVVSLWNGENWATDGGKSKINWSLAPFKANFQGFNNSGCFTNAEKNACGSSAYWWNTGSYSKLSDSEQKAYTNVRQKYMNYDYCSDKVRFHVPPSECKWNN

>AtXTH2

MNRIRYCFELVSVLFLMFTANARARGRGAIDFDVNYVVTWGQDHILKLNQGKEVQLSMDYSSGSGFESKSHYGSGFFQMRIKLPPRDSAGVVTAFYLTSKGDTHDEVDFEFLGNRQGKPIAIQTNVFSNGQGGREQKFVPWFDPTTSFHTYGILWNPYQIVFYVDKVPIRVFKNIKKSGVNYPSKPMQLVASLWNGENWATSGGKEKINWAYAPFKAQYQGFSDHGCHVNGQSNNANVCGSTRYWWNTRTYSQLSANEQKVMENVRAKYMTYDYCSDRPRYPVPPSECRWNQ

>AtXTH15

MGPSSSLTTIVATVLLVTLFGSAYASNFFDEFDLTWGDHRGKIFNGGNMLSLSLDQVSGSGFKSKKEYLFGRIDMQLKLVAGNSAGTVTAYYLSSQGATHDEIDFEFLGNETGKPYVLHTNVFAQGKGDREQQFYLWFDPTKNFHTYSIVWRPQHIIFLVDNLPIRVFNNAEKLGVPFPKSQPMRIYSSLWNADDWATRGGLVKTDWSKAPFTAYYRGFNAAACTASSGCDPKFKSSFGDGKLQVATELNAYGRRRLRWVQKYFMIYNYCSDLKRFPRGFPPECKKSRV

>AtXTH29

MRDSIYLLWIDNRLVVIIMMVMMVSCRCVLGLENINPIFFDEGLSHLFGEGNLIRSPDDRSVRLLLDKYTGSGFISSSMYQHGFFSSLIKLPGAYTAGIVVAFYTSNGDVFVKDHDELDIEFLGNLEGKPWRFQTNMYGNGSTNRGREERYRLWFDPSKEFHRYSILWTPTKIIFWVDDVPIREILRKEEMNGDYPQKPMSLYATIWDASSWATSGGKFGVDYTFSPFVSEFKDIALDGCNVSDSFPGENNNNNIGNYNNINCSVSDQFLMSNDYSTISPKQATAMRRFRERYMYYSYCYDTIRYSVPPPECVIVTAEKNRFRDTGRLKFGGSHPKVHKARKKRRRNRSTPVVSADL

>AtXTH23

MAMISYSTIVVALLASFMICSVSANFQRDVEITWGDGRGQITNNGDLLTLSLDKASGSGFQSKNEYLFGKIDMQIKLVAGNSAGTVTAYYLKSPGSTWDEIDFEFLGNLSGDPYTLHTNVFTQGKGDREQQFKLWFDPTSDFHTYSILWNPQRIIFSVDGTPIREFKNMESQGTLFPKNQPMRMYSSLWNAEEWATRGGLVKTDWSKAPFTASYRGFNEEACVVINGQSSCPNVSGQGSTGSWLSQELDSTGQEQMRWVQNNYMIYNYCTDAKRFPQGLPRECLAA

>AtXTH14

MACFATKQPLLLSLLLAIGFFVVAASAGNFYESFDITWGNGRANIFENGQLLTCTLDKVSGSGFQSKKEYLFGKIDMKLKLVAGNSAGTVTAYYLSSKGTAWDEIDFEFLGNRTGHPYTIHTNVFTGGKGDREMQFRLWFDPTADFHTYTVHWNPVNIIFLVDGIPIRVFKNNEKNGVAYPKNQPMRIYSSLWEADDWATEGGRVKIDWSNAPFKASYRNFNDQSSCSRTSSSKWVTCEPNSNSWMWTTLNPAQYGKMMWVQRDFMIYNYCTDFKRFPQGLPKECKL

>AtXTH26

MAGLQAKTLMFVLAAALATLGRTFVEADFSKNFIVTWGKDHMFMNGTNLRLVLDKSAGSAIKSKVAHLFGSVEMLIKLVPGNSAGTVAAYYLSSTGSTHDEIDFEFLGNATGQPYTIHTNLYAQGKGNREQQFRPWFNPTNGFHNYTIHWNPSEVVWFVDGTPIRVFRNYESEGIAYPNKQGMKVFASLWNAEDWATQGGRVKTNWTLAPFVAEGRRYKARACLWKGSVSIKQCVDPTIRSNWWTSPSFSQLTASQLTKMQKIRDGFMIYDYCKDTNRFKGVMPPECSKKQF

>AtXTH24

MSPFKIFFFTTLLVAAFSVSAADFNTDVNVAWGNGRGKILNNGQLLTLSLDKSSGSGFQSKTEYLFGKIDMQIKLVPGNSAGTVTTFYLKSEGSTWDEIDFEFLGNMSGDPYTLHTNVYTQGKGDKEQQFHLWFDPTANFHTYSILWNPQRIILTVDDTPIREFKNYESLGVLFPKNKPMRMYASLWNADDWATRGGLVKTDWSKAPFMASYRNIKIDSKPNSNWYTQEMDSTSQARLKWVQKNYMIYNYCTDHRRFPQGAPKECTTSS

>AtXTH18

MKLSCGTSFAFLIMFLFAAQSMHVYAGSFHKDVQIHWGDGRGKVRDRDGKLLSLSLDKSSGSGFQSNQEFLYGKAEVQMKLVPGNSAGTVTTFYLKSPGTTWDEIDFEFLGNLSGHPYTLHTNVYTKGSGDKEQQFHLWFDPTVNFHTYCITWNPQRIIFTVDGIPIREFKNSESIGVPFPTKQPMRLYASLWEAEHWATRGGLEKTDWSKAPFTAFYRNYNVEGCVWANGKSSCPANSSWFTQQLDSNGQTRMKGVQSKYMVYNYCNDKRRFPRGVPVECS

>AtXTH19

MKSFTFLILFLFAAQSISVYAGSFHKDVKIHWGDGRGKIHDNQGKLLSLSLDKSSGSGFQSNQEFLYGKAEVQMKLVPGNSAGTVTTFYLKSPGTTWDEIDFEFLGNISGHPYTLHTNVYTKGSGDKEQQFHLWFDPTANFHTYCITWNPQRIIFTVDGIPIREFMNAESRGVPFPTKQPMRLYASLWEAEHWATRGGLEKTDWSKAPFTAYYRNYNVEGCVWVNGKSVCPANSQWFTQKLDSNGQTRMKGVQSKYMVYNYCSDKKRFPRGVPPECS

>AtXTH7

MVVSLFSSRNVFYTLSLCLFAALYQPVMSRPAKFEDDFRIAWSDTHITQIDGGRAIQLKLDPSSGCGFASKKQYLFGRVSMKIKLIPGDSAGTVTAFYMNSDTDSVRDELDFEFLGNRSGQPYTVQTNVFAHGKGDREQRVNLWFDPSRDFHEYAISWNHLRIVFYVDNVPIRVYKNNEARKVPYPRFQPMGVYSTLWEADDWATRGGIEKINWSRAPFYAYYKDFDIEGCPVPGPADCPANSKNWWEGSAYHQLSPVEARSYRWVRVNHMVYDYCTDKSRFPVPPPECSAGI

>AtXTH5

MGRLSSTLCLTFLILATVAFGVPPKKSINVPFGRNYFPTWAFDHIKYLNGGSEVHLVLDKYTGTGFQSKGSYLFGHFSMHIKMVAGDSAGTVTAFYLSSQNSEHDEIDFEFLGNRTGQPYILQTNVFTGGAGNREQRINLWFDPSKDYHSYSVLWNMYQIVFFVDDVPIRVFKNSKDVGVKFPFNQPMKIYSSLWNADDWATRGGLEKTNWEKAPFVASYRGFHVDGCEASVNAKFCETQGKRWWDQKEFQDLDANQYKRLKWVRKRYTIYNYCTDRVRFPVPPPECRRDRDI

>AtXTH20

MVSFCGRRFAFLIIFLFAAQYERVYAGSFHKDVQIHWGDGRGKILDNVGNLLSLSLDKFSGSGFQSHQEFLYGKVEVQMKLVPGNSAGTVTTFYLKSPGTTWDEIDFEFLGNISGHPYTLHTNVYTKGTGDKEQQFHLWFDPTVDFHTYCIIWNPQRVIFTIDGIPIREFKNSEALGVPFPKHQPMRLYASLWEAEHWATRGGLEKTDWSKAPFTAFYRNYNVDACVWSNGKSSCSANSSWFTQVLDFKGKNRVKWAQRKYMVYNYCTDKKRFPQGAPPECS

>AtXTH12

MAAFATKQSPLLLASLLILIGVATGSFYDSFDITWGAGRANIFESGQLLTCTLDKTSGSGFQSKKEYLFGKIDMKIKLVPGNSAGTVTAYYLSSKGETWDEIDFEFLGNVTGQPYVIHTNVFTGGKGNREMQFYLWFDPTADFHTYTVLWNPLNIIFLVDGIPIRVFKNNEANGVAYPKSQPMKIYSSLWEADDWATQGGKVKTDWTNAPFSASYRSFNDVDCCSRTSIWNWVTCNANSNSWMWTTLNSNQLGQLKWVQKDYMIYNYCTDFKRFPQGLPTECNLN

>AtXTH13

MAAFTTKQSLLLLSLLLLISLSAGSFYDNFDITWGNGRANIVESGQLLTCTLDKISGSGFQSKKEYLFGKIDMKMKLVAGNSAGTVTAYYLSSKGETWDEIDFEFLGNVTGQPYVLHTNVFTGGKGNREMQFYLWFDPTADFHTYTVLWNPLNIIFLVDGIPIRVFKNNEANGVAYPKSQPMKIYSSLWEADDWATQGGKVKTDWTNAPFSASYKSFNDVDCCSRTSLLNWVTCNANSNSWMWTTLNSNQYGQMKWVQDDYMIYNYCTDFKRFPQGLPTECNLN

>AtXTH25

MDRSTFILSLLFTLTVSTTTLFSPVFAGTFDTEFDITWGDGRGKVLNNGELLTLSLDRASGSGFQTKKEYLFGKIDMQLKLVPGNSAGTVTAYYLKSKGDTWDEIDFEFLGNLTGDPYTMHTNVYTQGKGDREQQFHLWFDPTADFHTYSVLWNPHHIVFMVDDIPVREFKNLQHMGIQYPKLQPMRLYSSLWNADQWATRGGLVKTDWSKAPFTASYRNFRADACVSSGGRSSCPAGSPRWFSQRLDLTAEDKMRVVQRKYMIYNYCTDTKRFPQGFPKECRH

>AtXTH22

MAITYLLPLFLSLIITSSVSANFQRDVEITWGDGRGQIKNNGELLTLSLDKSSGSGFQSKNEYLFGKVSMQMKLVPGNSAGTVTTLYLKSPGTTWDEIDFEFLGNSSGEPYTLHTNVYTQGKGDKEQQFKLWFDPTANFHTYTILWNPQRIIFTVDGTPIREFKNMESLGTLFPKNKPMRMYSSLWNADDWATRGGLVKTDWSKAPFTASYRGFQQEACVWSNGKSSCPNASKQGTTTGSWLSQELDSTAQQRMRWVQRNYMIYNYCTDAKRFPQGLPKECLAA

>AtXTH6

MAKIYSPSFPGTLCLCIFTLLTLMFIRVSARPATFVEDFKAAWSESHIRQMEDGKAIQLVLDQSTGCGFASKRKYLFGRVSMKIKLIPGDSAGTVTAFYMNSDTATVRDELDFEFLGNRSGQPYSVQTNIFAHGKGDREQRVNLWFDPSMDYHTYTILWSHKHIVFYVDDVPIREYKNNEAKNIAYPTSQPMGVYSTLWEADDWATRGGLEKIDWSKAPFYAYYKDFDIEGCPVPGPTFCPSNPHNWWEGYAYQSLNAVEARRYRWVRVNHMVYDYCTDRSRFPVPPPECRA

>PeaxiXTH1

MRIKIPKNRTIGGVITAFYVIQDDQLASGNHDEIDFEFIGTEGRLQTNLFANDMGGREQTFLLPFDPSEDYHTYQILYSPHHIVWFVDNIPIRAFQNNTMRGVNYPSRPMWAESSLWYSNAVNWAGPIDWNQEPFIAHYQDFNISTCPSYQPVGSCIMSPAVLKTWNTWNRPKVDLHQLQLMWNFRQKHMIYDYCKHRGARYPECPHRAN

>PeaxiXTH2

MGFRWMILVCVLFIVCCGGAMAGKPKTPTDVPFGRNYEPSWAFDHIKYLNGGSEIQLYLDNRTGEPYILQTNVYTGGKGDKEQRIYLWFDPTKEYHTYSVLWNLHQILYSSLWEADDWATRGGLEKIDWSNAPFVASYKGFHIDGCEASVNAKYCETQGKRWWDQQDYQDLDKYQWRLLRRVRDKYTIYNYCIDKKRFAKVPPECKANRDVPRTSKKSP

>PeaxiXTH6

MATLTIKSSAVFLMLYALTFSFSVSARPATFLQDFKVTWSDSHIKQIDGGRAVQLILDQNSGMILYFLVSVLAFRCGFASKSKYLFGRVSMKIKLVPGDSAGTVTAFYMNSDTDNVRDELDFEFLGNRSGQPYTVQTNVYVHGKGDKEQRVNLWFDPSADFHTYSILWNHNHTIFSVDEVPIRVFKNNEARGIPYPKFQPMGVYSTLWEADDWATRGGLEKIDWSKAPFYAYYKDFDIEGCPLPGPAYCASNSRNWWEGANYQQLNAVEARRYRWVRMNHMIYDYCTDKSRNPVTPPECLDGI

>PeaxiXTH15

MASNFSSAMLLLSMLMGTIISASAGNFYKDVVITWGEGRGKIQEGGRGLALTLDKLSGSGFQSKNEYLFGRFDMQLKLVPGNSAGTVTTFFLSSQGEGHDEIDFEFLGNVSGQPYTVHTNVYTQGKGNKEQQFHLWFDPTAAFHTYTIVWNPHRIVFLVDNSPIRVFNNHESIGIPFPKTQAMKVYCSLWNADEWATQGGRVKTDWALAPFTAYYRNINIDGCAVSSGTSSCKSGASANNAKPWQTHELDGKGRNRLRWVQSRHMVYNYCADSKRFPGGFSEECKRSRF

>PeaxiXTH25

MASSLKLLLVKCLMLSVFGIAIAGKLLDQEFEITWGDGRAKILDNGELLTLTLDKTSGSGFQSKNEYLFGKIDMQIKLVPGNSAGTVTAYYLTSQEGPTHDEIDFEFLGNLSGDPYTLHTNVFSQGKGNREQQFHLWFDPTADFHTYSILWNPQRIIFYVDGTPIREFKNPESLGVPYPKNQPMRIHSSLWNGEAWATRGGLVKTDWNQAPFTASYRNYNANACVPNSPSSCNSNSSSWFNEELDKTSQERLKWVQNNYMIYNYCSDSKRFPQGLPPECAM

>PeaxiXTH10

MKNFCRLICFATVFIYLFQVSLASIVSTGDFNKDFFVTWSPNHVNTSADGHSRSMIFDKESGSGIASNDMYLFGQFDMKIKLIPGNSAGTVVAFYLTSDQPNHDEVDFEFLGNVPGKPYTLQTNVYVDGLDDREQRINLWFDPTEDFHTYSILWNLHQIVFMVDWVPIRTYRNHADKGAKYPRWQPMALKISLWNGESWATDGGKTKIDWSNAPFVATLGNYSIDACIWKGNARFCRAESESNWWNKDNFSTLTSTQRRLYKWVRENHLTYDYCIDNERFQNKLPIECSLPKY

>PeaxiXTH9

MVSFSMAFSCVFLGICIMMVGFVSSARFEELFEPSWALDHLTNEGEVLRMKLDSSSGAGFSSKSKYMFGKVTVQIKLVEGDSAGTVTAFYMSSEGPTHNEFDFEFLGNTTGEPYLVQTNVYVNGVGNREQRLNLWFDPSKDFHSYSILWNQRQVVFLVDDTPVRVHSNLEHKGIPFPKDQAMGVYSSIWNADDWATQGGLIKTDWSHAPFVASYKGFEIDGCECPSTVAAVENTRKCSSSAEKRYWWDEPTMSELSLHQSHQLIWVRANHMVYDYCTDTARFPVAPVECQHHRHN

>PeaxiXTH24

MSSYTSSKLVLLVICLMVSAFGIANADKFDKEFDITWGEGRAKILNNGDLLTLSLDKISGSGFQSKNEYLFGKIDMQLKLVPGNSAGTVTAYYLSSQGPTHDEIDFEFLGNLSGDPYTLHTNVFSQGKGNREQQFHLWFDPTADFHTYSILWNPQRIIFYVDGTPIREFKNPESLGVPYPKNQPMRIYSSLWNADDWATRGGLVKTDWTQAPFTASYRNFNANACVPNSPSSSACNSNSASSSSWLNEELDNTSQERMKWVQKNYMVYNYCSDSKRFPQGFPAECAQNS

>PeaxiXTH5

MGIKAILVGFVLINLSILGSGWAPRKPVDVPFLKNYEPTWGSHHIKFLNGGTTAELLLDKASGAGFQSKKSYLFGHFSMRMKLVGGDSAGVVSAFYLSSNNAEHDEIDFEFLGNRTGQPYILQTNVFTGGKGDREQRIYLWFDPTKDFHSYSVLWNTFQIIIFVDNVPIRVFKNSKDIGVKFPFNQPMKIYSSLWNADDWATRGGLEKTDWSTAPFTASYTSFHIDGCEAVTPQQVQVCNTNGMKWWDQKAFQDLDGPQYRRLRWVREKFTIYNYCTDRKRYPTLPPECTRDRDL

>PeaxiXTH16

MPYVSSSRMLTMFMQLSVLVVFLLCTASADNFHQDTAITYGDQRVQILDGGRRLTLSLDKFSGSGFQSKNEFLFGRFDMQLKLVPGNSAGTVTTFYLSSQGAGHDEIDFEFLGNSSGQPYTVHTNVYSQGKGNKEQQFHLWFDPTSSFHTYSIVWNPQRIIFLVDNIPIRVYNNNEALGVAFPKNQAMRVYASLWNADDWATQGGRVKTDWSMAPFTASYRNFNTSACVWSAASSTSSCGGTDSVNNNQAWQTQELDANGRNRLRWVQQKYMIYNYCADISRFPQGLSPECKRSRF

>PeaxiXTH7

MATLTIFSLKKSAVFLVLYALAFSFSVRCGFGSKSKYLFGRVSMKIKLVPGDSAGTVTAFYMNSDTDNVRDELDFEFLGNRSGQPYTVQTNVYVHGKGDKEQRVNLWFDPSADFHTYTILWNHHHCVFYVDGVPIRVYKNNEARGIPYPKFQPMGVYSTLWEADDWATRGGIEKIDWSKAPFYAYYKDFDIEGCPMPGPAVCASNSRNWWEGTNYQQLNAVEARQYRWVRTNHMIYDYCTDKSRNPVTPPECLAGI

>PeaxiXTH26

MANFIAFSLVLVIVTVAFPLSLVNGKIASSMYVNWGKHHCSMQGDDLQLVLDKSSGSSFGTKSSGAQSKRSFLFGSFEMQLKLVANNSAGTVTTYYLSSTGTKHDEIDFEFLGNISGQPYILHTNIYAQGVGNREQQFYPWFDPTADFHTYTIHWNPNAVVWYIDGIPIRVYRNYQSQGIPFPNQQGMRVYTSLWNADSWATRGGLVKIDWTSAPFVATYRKFRPRACYWNGPSSISQCALPTKTNWWTSPVYSKLSAAKVDQMNSIRSKYMIYDYCKDTKRFNGQMPKECSLPQN

>PeaxiXTH8

MERKAPSITYFLLTAALTATLFSSTQAEVQGSFDDNFSKSCPETHFKTSEDGQTWYLSLDKKAGCGFMTKQKYRFGWFSMKLKLVGGDSAGVVTAYYMCTEDGAGPTRDELDFEFLGNRTGQPYLIQTNVYKNGTGNREMRHVLWFDPTEDFHTYSVLWNSHQIVFFVDRVPIRVYKNANYTNNFFPNEKPMYLFSSIWNADDWATRGGLEKTDWKNQPFVSTYKDFSVDGCQWEDPYPACVSTTTKNWWDQYNSWHLSSDQKLDYAWVQRNLVIYDYCQDTERFPKKPEECWLSPWD

>StXTH7

MTTLTSPSLKYSAFVLIVLYALTFSFSLVSARPATFLQDFKIAWSDSHIKQLDGGRGIQLILDQNSGCGFASRSKYLFGRVSMKIKLVPGDSAGTVTAFYMNSDTDTVRDELDFEFLGNRTGQPYTVQTNVYVHGKGDKEQRVNLWFDPSADFHTYTIFWNHHQAVFSVDGIPIRVYKNNEAKGIPFPKFQPMGVYSTLWEADDWATRGGLEKINWSKSPFYAYYKDFDIEGCAMPGPANCASNPSNWWEGPAYQQLSPVQARQYRWVRMNHMIYDYCTDKSRNPVPPPECRAGI

>StXTH6

MKITFLFFLILSFLFLVVALAGNFNQDFDITWGDGRAKILENGQLMTLSLDKASGSGFRSKNQYLFGKIDLKIKLVPGNSAGTVTTYYLSSIGSSHDEIDFEFLGNLSGDPYILHTNVFTQGKGDREQQFYLWFDPTKDFHTYSILWNPQSIIFSVDGTPIRQFKNMESSGIPYPKSQPMWIYSSLWNADDWATRGGLVKTDWTKAPFIASYTNFNAQACVWSSTSTASSCNSTTQNSWLSENLDITGQSRIKWVQNNYMIYNYCNDIKRFPQGFPRECSLN

>StXTH5

MGFKWMMMLVLCVLIGGSMGAKPNKPIDVPFGRNYEPSWAFDHIKYLNGGSEIQLSLDNRTGTGFQSKGSYLFGHFAMHIKMVAGDSAGTVTAFYLSSQNSEHDEIDFEFLGNKTGEPYILQTNVYTGGKGDKEQRIYLWFDPTKDYHTYSVLWNLYQIVFFVDEYPIRVFKNNKNLGIKFPFDQPMKIYSSLWEADDWATRGGLEKIDWSNAPFVASYKGFHIDGCESSVNAKFCANQGKSWWDQKEFQDLDTTQWRLLRRVRDKYTIYNYCTDKKRFSTMPKECKRNRDVPRNS

>StXTH9

MASLVLCLVSFAFCFLHYSLASNNFNQDFDVTWGDGRAKVLNNGKLLTLSLDKVSGSGVKSKKEYLFGRIDMQLKLVRGNSAGTVTTYYLSSQGSTHDEIDFEFLGNLSGDPYIVHTNVYTQGKGDKEQQFYLWFDPTADFHTYSILWNPQTIIFYVDSTPIRVFKNMKSSGVPYPNNQPMRVYASLWNADDWATRGGLIKTNWSNAPFIASFRNFKDNNACIWEFRKSSCTNSTKSWFSQELDSTSQARLQWVQKNYMVYNYCNDINRFPQGIPLECTFNSTTS

>StXTH3

MASSSKLVLVMCFMVSAFGIAIGNTLDQEFEITWGDGRAKILNNGDLLTLSLDKISGSGFQSKNEYLFGKIDMQLKLVSGNSAGTVTAYYLSSQGPTHDEIDFEFLGNLSGDPYTLHTNVFSQGKGNREQQFHLWFDPTADFHTYSITWNPQRIIFYVDGTPIREYKNSESMGVSYPKNQPMRIYSSLWNADDWATRGGLVKTDWSQAPFSASYRNFSANACIPSSSSSCNSNTTTSTSNSWLNEELDSTSQERLKWVQKNYMVYNYCTDSKRFPQGFPVDCVQNN

>StXTH1

MGTIKGVLFSIVLINLSLVGFCGYPRRPVDVPFWKNYEPSWASHHIKFLNGGATTDLILDRSSGAGFQSKKSYLFGHFSMKMRLVGGDSAGVVTAFYLSSNNAEHDEIDFEFLGNRTGQPYILQTNVFTGGKGNREQRIFLWFDPTKGYHSYSVLWNTYLIVIFVDDVPIRAFKNSKDLGVKFPFNQPMKIYSSLWDADDWATRGGLEKTDWSNAPFTASYTSFHVDGCEAATPQEVQVCNTKGMKWWDQKAFQDLDALQYRRLRWVRQKYTVYNYCTDKARYPVPPPECTKDRDI

>StXTH8

MPSLFSFNIRLILIPVFISCMVVEYCASNDLNQDFDITWGNERGEILNNGEILTLTLDNISGSGFESKKEYLFGKIDMQIKLVQGNSAGTVTAYYLSSKGSNHDEIDFEFLGNVSGEPYTLHTNVYTQGKGEREQQFHLWFDPTNDFHIYSILWNPQTIVFSVDNVPIREFKNMENIGVAFPKSQSMKLYSSLWNADEWATMGGLIKTDWAQAPFTASYRNFNANICNNNNNSCKFLVENLDPVNEEKLRRVQQKYMIYNYCTDNKRFPQGFPPECSAT

>StXTH12

MSTIFFLPIFLSFIFLHSTNANYWPISPGYYPSTKFKSMSFYQGFKNLWGPNHQSVDNNGINIWLDRNSGSGFKSVKPFRSGYFGASIKLQPGYTAGVITAFYLSNNEAHPGFHDEVDIEFLGTTFGKPYTLQTNVYIRGSGDGKIIGREMKFHLWFDPTKDFHHYAILWSPREIIFLVDDVPIRRYARRSDATFPLRPMWLYGSIWDASSWATENGKYKADYNYQPFFGKFTNFKASGCTAYSSRWCRPVSASPYRSGGLSRQQRQAMNWVRSHYMVYDYCRDFKRDHSLTPECWRK

>StXTH13

MVNYYMFFFIFLSCILVLVSGFSRNLPILAFDEGYSHLFGDNNLMILKDGKSVHISLDKRTGAGFVSQDLYFHGFFSASIKLPADYTAGVVVAFYMSNGDMFEKNHDEIDFEFLGNIRGKDWRIQTNIYGNGSTSVGREERYGLWFDPSEDFHQYSILWTENLIIFYVDNVPIREIKRTKAMGGDFPSKPMSLIATIWDGSNWATNGGKYKVNYKYAPYIAEFSDFVLHGCAVDPIELSSKCENTTPKTPSIPTGITLDQRRKMENFRKKQMQYSYCYDKTRYKVPPPECVIDPKETERLRAFDPVTFGGSHHHHGKQHRRSRSKLKDDDVSFM

>StXTH2

MIERLQLTSLQINGDPGIRHDEIDFEFLGGDGKYTLNTNIFANDGGSREQQFNLEFDPTTDFHTYRILWNQYHIVFYADDVPIRVFKNNTNYGVNYPTNKMHIEATIWNDTNWVGAVDWSQGPFKAYYRDFSINGCQYQESNPQECYNNNYYWNTITNLSPDEVQKYEDVKAEQMTFSYCMRNNSMNFPECMLN

>StXTH4

MNHFPRFIFLATFLIYLSHIALASIVSTGDYNKDFYVTYSPSHINTSVDGRTRNLIFDKESGTEIATKDMYLFGQFDMKIKLIPGNSAGTVVAFYLASGQPNRDEVDFEFLGNVAGKPYTLQTNVYVDGFDDREQRINLWFDPTQDYHTYSILWNLHQIV

>StXTH16

MGVYSSIWNADDWATQGGLVKTDWSHAPFVASYKGFEINGCACPATVASAENTRRCSSNGQKKYWWDEPVMSELNVHQSHQLIWVRANHMVYDYCTDTARFPVAPVECQHHQHKTNHN
