# Supplementary material for: Genome-wide characterization of the xyloglucan endotransglucosylase/hydrolase gene family in Solanum lycopersicum L. and gene expression analysis in response to arbuscular mycorrhizal symbiosis
Source: PeerJ. 2023 May 3;11:e15257. doi: 10.7717/peerj.15257 (PMC10163873; doi:10.7717/peerj.15257)
Supplement: Supplemental Information 6 [file peerj-11-15257-s006.docx]

**File S6.** Promoter sequences of tomato *XTHs* used for *cis*-acting regulatory elements analysis*.*

>SlXTH1

TGAATGATCAAAAATACTATTACTTATTGATTAAAATATAAATTAATTTGTTTTAACTCTTTTAAGTATAAAAAACTTAAAATTAAACAAATTTTTTTCATGTTAACCAATTTAAAGGTATTTCAAATATTTTTATTTTAAAAAGAAAGTGTTTCCCCGCATTTATTTGCAAAACACATCAAGAATCTTTTTTCAACTTCGACACTTTTATTCAAACATATGAATAATTATTTCAAATATAATTTTTAGCACTTTAAAAATCTTTTTTTAATTCAATCTAAATAGGCTCTTAATAATTTTTTAAATTAATTAGACTTATTTTTAAATTTAATAATTATTTATAAAAAAATCGTATAAAATCGAAAAAAACAAAAGCACGCGCTATTAGGTCGAGTGAGATGGATGGGGTCATAAAATTTTGCTCCTCGGTCTGAGGGTGACAAGCCTTTTCTCTGATACGGGCATGTGCATGTCCCCGTTAATTACTCCCCCAATGTGCAATTACCCACTAACTCTAACCCCTCTTTTGGACAATTATTTGAAAGGCTTTAATTTAATTATTTTTTTGTTTTTCATTCCATCTATACTTATATTAAAGTTGAATCAAATTTAGAATTACACTTGTATTTAGCACTAAAGTGCTATATAATAAAAAATGACTATGTACTCAAGAAAAATTAAATTTGAAATCAACAGAAGAGTCATAATTTTTAATAAAGAAATTTAAAATTTATAAAAATAAACACAAAAAATGTCTCAAAGGAGATTAGATATCTATTAGAATATTATTGTAATAAAATATAAATAATATAATTTTGCATATTCGAAATTTCTGATTAAGGACGAAAGAATAATCGTGGCTGCACAATAACCTTTGTTGGTGAAAGGACAAATTTCAACCACCCAAAATCTGAAAAATCTAACTTTGTTTCAACTTTCAACCACAAGTCCAACTCAGTCCCTTTTACACCTATAAATAACCAGTCACTACACTTCCATT

>SlXTH2

NGCTATTATTATTATATATATATATATATATATATATATATATATATATATAATCTTAAACCTTATATGTGTATATATTGTAATTGTTTCACAAAGGAAGTTCAAATAAACCTTGTATCCAATAAGCTTCCCTCATGACTTATGATGTTCATTATAAATTTTTATTAGTGTATTGGTATTTGTCTTCTTAATTAATGAAAGTTAACTTGTTATGGTATGTCAATTTTCGTTTTTACTATGTTACAAATTAATTATACTATAGTATTCTGATTAGGATGTTATGTGAAAATTTATAATTTAAAATATATTGGCGATAAGTTAGATGACAAAAACTTATATTAAAAATATAATATTATTATATAAATTTAATGTAAAAGAAAAATATTAAAACTATTGAGAGAATAAATTTCTGAAAAGCCTATATTATGGATGTCATTGTGTTATGATATAAGAAGAGGATATTTAATTTATAGAGTTCCAAAATTTTTCTTTAAGAAAATAAGTAATTAAATGTGGAAGAGAATTATATTTTTCTTTTGGGAAAAGTTCAAATATTTATGGTAATACTTTGACTTTTATTTAAGGAAAAAATAAAACTTAAAATATGTTTTAAAAAAAATTTAGGGCAAAACCCTAACAGATAATACGAGCTCACTTCTGTTTTCTTCCTAGATATATTTCTCCCCCACCTATCGAAATAAAAAAAAATCCCCTTTACCAAGTTCAGTACGAAATCATTCTAAAAAATGTATTTTATAAGTACCAAACACCTATAAAAAGGTTGAAATGAAATTTTTGTCTATAATTATGTTGTAAAATTTTATGTTTTGTCGGTCCATAAAACTTTAAAATGTCTCAAAAATAGGTTTAGGAGCCAAAAAAATCAACGAAAAAGAAATCCAACCCCCTCTACCTCCCCCCCCCCCTCTTCCCCAAATAAAAAAATAATAATTGTGTCTTTCTCCCAACACTACTATATATAATGCCTCTAATTAACCAT

>SlXTH3

AACACAAAAATATATATGATCCAATGATAGAAATAAAATGAAAATATAATAACAAGAATTTTACGTGAAAATTCTTTTAAATAAGTGAAAATTTATGAGTTAAGAGGAATAACTGATATTATTATAGTAAGAAATTTTATACTGTATAGTCACGAATACAATACTCAAAGTGATAACAATACATTAAAAAGCTCGCACTCTATTTTTTTTATATAGTCTATGTAATACCTCACGTTGCTCTCAAAATTTTTTTTTCTAACTTAATGTGTTCACAAATGAGCAAGAATGTTCTATTTATAAAAGGAGAAAATCATACCTATGTCACTACTGACATAGGTAAATGCACTAAAACCAAAAGAAGTTTGCAAATTTTATAAATTTTCAACTACCAAATCTTTTATTTTCAACTCTTATCATTATTCCTTATAAACAATCACTTGTAGCAATTGAATAACAAAAATTTGGTAAAAAAATATAAAATGAACTATATATGATGTTTGTACTTTTAAAAAATGTTATAATTTCTCTATTTTGTAAAATAGAAATACGATTTGTATATATGCTCTACTTCTCATATTTAACCGGTGAAATTTATATTATGTATTATGTTGTAATTTAGTGTAAACATATAATATGATAAATTTTTTAATTTCTTCTTGGATTTGACTTATTCTTTTCCATCTTCATTTGTAGGAGCGCAGAACAGGCAATGACGATCAAATAAAAGAAAAATACTAGGACTACGCGTACGCCTATTTGGTATGGTGTGTACTTTAATACCACAATCTATAAATTAAAGTTGAACTAATTCCAATGAAAATTATAAGAGTGAGAGTTGTAAATTGGCTGTCACTTTTTTTCCTGTCGCCAGCTCAATATTCATTTTTTTTTCTTCTACTTTCTGTTTTACTTTGAACCTTCCTCCTTATTCCCCCACACGCTTGGGCTACTAAAGTGGGGTGGTATAAACCCCTATAAAACCCCATATAACTATCCAACT

>SlXTH4

ATATTTTGTTATAATTTTTTATCAAAAAATTACGTAACTATACTGACGTATAATAGTTAGTTATGCTCCATAACTACAGTTTAATTCAATTTTTACAAATAAAAAAATATAATTTTTTATCTATTACATTCTCAATTTATTTAGAAAATTAATTTTTCCAAAAGGGATAAAATCACTCTTAACAAATAAATTAATTAATAAAAATAAAATAATAAATTTAATATATTAATAATTATTTCCAAAAGTAATAGTTATTTATTGATTGAGGGTAACAAGAAACATGTGCATAACACCATAGATTGCTCTTCTAATGTGCATCAAACAAAGGGCTTCAACTTATTTATGAGTATTTTGGGACAGTTACACTCTTACAATTGGAACTCTAAATATAGCACCAAAATATCTCACTTGCTTCATTGTTATATTGACACATGACTAGTGTTCTTTTTCAATGCACTACTTCCTCTAGTTTATATTAAATAAATTATTGACCACAGAAAATAAATAACGCGTTATTTTTTTAATAATTTTCTTGAACTTTAATAATTCATTTACTTTAGCCATAGAAGAAGTCTTTAACAATTCATTTATGCATAATACGTGAACACATGCTCTTTCACTTAATTTGTGTTGGTATTTGTGTTTTTATTTTTAAATTTGATAAGTAGATACTTAAACTTATATTGAATTGAACAAGTGGATGCATATAACATATCACACGTGAAATATCATGTAGGATATATGAATTACCACAAAAAACGTCATGTAAGATGCTTATGTCTACTAACATATTAAAAATTGCTGAATATAAATGCAAGTTAAAATTAAATTAAAAAATATTTTTATGTATCATTCAATTCATTTAATATGAACCAAAATGATTGAGTAGTATTCAAAATCCCCTTCTATAAATAATGAATCATATCTCTATTAACTTTGCCTCATCCATATGACCAATATCTCATTCAACAAAAACAAAGGAAGATAGTAAGTAATGAAT

>SlXTH5

ATTCTTTCGATCCTTCTCTGCAAATAGAACGATGAAATGAGTTAAATAATTATTGCTATCATTGAGTTAAAAGAAAAATAAATAAAAAAATAAAAGGAAGAAAGAAAGAATAAGCTTGGAAATGATTACAACTTTAATGTAAAAAAATCATAACACAAATAGACTAACGTTAGTTGTACATTTGTTTTTATTTCAAATATAAGTATGATTCTTATCTTGTTAATAACCTATGAATTTGTTGAGAATGTAAAATCAACATTTCAAATTGATATATCATAGTGACGTCTGACGTGTCATACACTACACTTGTAAAGGCATTATGAACTTATCAAAATTAATTTTTTTAGCCTTTTTTATTATTATGAAATTAATTTATTAAAAATAATGCAGTTTGATATATCTTGCCGGAAAAGCAACTAACAACTATAAATCGGAAATGCATTAAATAATGTTATCCAAATATAGAATAAGTTTAATCATAGACTACAAAAACAATAAGCAAATGTAATTCCACAAATAAAATATATAAAAAAAAATGATATATCGAGAATTTAATTTTATAAGGTAGAAAATTATTTATTATAGATATTTAAAATAACGATAGTAATGTGGCAAGATACCCTCGACCAATTAAATAATATGAATAAAAGAACTATCTATTCAAGTAAATCGTAATGCTATTTTAATAAAAAGAAAAAGTGACTAGCTTACTTACGATCTCTAGTATTACAACACTCGAATATTTATTAATTAGTACTTTTTTATTATAATCTTCATTCTTTAAATATTTCTATCTAGGATCATATATATTTTTAATCAGTTCGGAATATTTTATGTCCAACCAAACCACCTCCTATCTCCATTTTTTTAAACCATAAACTCAAATAAAAAACAAAATCTATTTAAATGCTTGTATATTTCCAAAAATACCCCTACAAACCTTGTTCATAACGTAACCAACCAACACAAATATAAAAACAATTTATCCAAAACACCCCCT

>SlXTH6

TCGTAATATTTTACCTTCTACGTGTATTTTCTTTTCGATTATTTTTTTTTCTAAATTTATTGTTTTATACTGATTTTCTCATTAATTTGGTTAGCTGCATTTGAAGAAAAGTCTTTTGAAAATAACCTCTCTATCTTTACGAGCTAAGAATTAGGTATCAATTTGTAACAAATATGTATTACTCCAAAATAGTTATATATAAATTTAAAATATTTTTCATCGTATCGAACAATAAGCGTGTAAAAAGGAATGAAGAAATTAAAAAAGAAATGGAAAAATAAGAATGCCACATTATGGCATTTTCCTTCCATCTTCTTGTTTTGTTAGCTTCCCATAACTAGTAGAAATCCCAACCAACTAAATTTGTTTTTACAAGGGAATATTAAAAATAAAACTTTAAAAATTTGGAGAAAAACTATATAGATATCTCAAAATATGTAATATAAACATTTGTTTTGACGCAATTAATTCGTAATCGATTTCACTAACGTGAATAAGTAATCACTTATAAGTTAAAAACAATAGTTACTAATCAAAAAAAATATATATATAGTGAAAATTAAACTCACACTTATTCCATAAGAAATTAAATCTTCACTAAATTACTAGATGTTATAAGCTTTAGTATTTTATTGACATATATATGATTTTATTCAATTCAAAATAATAGTAAGATTTGTGTACGATTTATACTCTTCAGACACTAATCTGTTGAAGCTACATTAAGTATATTGTTATTCTAGCGTCTTGATTTAATACATATTATATTTATATTCATAAAAAAAAAACTTTTTTTTTATATTTGTCATTTTGTGTAACTCCCATCAACCATTCTTATCAATTCAATTTCTTTTAGTAGTAGGACCCTTTTATAATCTCCTTATATACCCCTCTCTATATGTTCATCTCCCTTCAATTCTACAACAAAGTGTACTAACACTCACTCAACACCAAAGTACCATTAAAACAACTTTTTGGATTTTATATCCAAACAATTATT

>SlXTH7

CAATTCCACATCTTGACTTGAGTGTTTTTAGGTAAAAGTCGTCCAAGTTTGATTCCAAGTTAGACAATATCATTTTCATTTTATATTAAGGATATTATTTGATTGTTTTAGCTTAATAATTGATATTTGAATCTTCTCCATTTCAAATTATTGCTTATATTGTTTATGTTTCAAATGCCCAATTCTAAATATGTGAAGGTGTTGAATTATACATTAATTATAAAATTAAAACTAGATCTCCTCCATATAAATAATATTGAAAAAATTCTGACTTCAGACTTGGATATGTCATTTTTTTAATCACTATCATATCAAACACACCTTTTTAGAAGTCTTTAAAATGAGTAATAATGTACAATTTGGCATCCATACATAGGGTACACCTAGCTAGATGTTATTTATGTCACCTAAATTTGAATGTTGTAATAATAAAAGTAGTTATTTGATGACTAAAGACTAACCTTAATAAAAGTAATGGAGACACCATTCAAAGAGAGATCGTTGTCTTCGATCGATGAAGATTACAATCAATACGGTTGACGTTGGAAAAAATAAAGAAAAAAAATATTTGAAAGTTAAAATTATGCTTGAATACAAATTTCCTCCTCTTTAAAAAAGGTTGATTTATTTATTTAGTATATGCAAATAAATTCTTTGTTGTTATATTTGCAAATAATGAAATTGATACCGGGTACATCCAATTAGGCTTTGAAAAAGTCAAAAGTCATTTATATAAAGTAATTAGAAGACCGTAGATTAGATAGGACCTCTGGTTTAGTAGCTACTAATTGCAATAAACATGAAAGTAAAAATATGGTGATATGACAGAAGAAGGGAAAGAATTATTCTGGATTAATTGTAGGGTTTGGCATAGAGAGAGGGTCCATTGACGGCTTGCTATTAGCTGTAAATTGTAATATGAAAAAATGAAAATGGAATTTCTTTCGTCGTTTTCTTTTATACTTACTCATATATAAACCTCACAAAATTCCACACACTA

>SlXTH8

TGAAATGCGTTTGTATTTATATAAAGCGAGAAAAAGTTGTATATATACATATATTTTCGTTCCCTCTCTCAGATCTCGCTTGCTTCTCTCACTTATACAAACAGAAAAGAAATGTATAAATTGTGTTTTCGTTTGTATAAAGCGAGAGAAAATGTATATACACATGGAAATATATACATCTTTGTCCTATACACTTATAATTATACAATACAAATATTCTTATGCCTAGTTTTCTTTTGTCTTTTACGCTTTCTCATTTTACACAAACACAAGTTATACCGAAACAGAAACAGTTTATAAACAACTATTTTATTTTATATATATATATATATTATATATATATCGAAATATACATATTTATGTTTGCTATGTAGCGCAATTACGTAACATAGTTGTAATATACAAATATGATTTTTGTGTTTGTTATATGTGAAAACTGCTTTTAAAAGAAATCTAAAGCATGATTTCGCTTACTTCTTCCGTCTTAATTTATTTGTCCGCTCTTTACTTAGCGAAAAACTTTTAAAAAATCTGAAATTTACGACCTGATATATTAAAATTAAAAAGTTATTACAATAAGAAAAATAAAATATTTCGTATCATTTCTCTCCAATCAAAACAAACCACATAAAAGGAAATTTATTCTTTTTTTAGTTTCTTTTGTCTCGTGTATATATATGTACCGTGTGTATATTATTATCCATTTACTTATATTTTTCCCTTCTTTTATCACAAGCCACTAAATTTGAAAAAAATAATAATAATAATAATAACTCCTTTTTTATATATATATATATATATTTTGTCGTTTCTCTTTTCCCAAATTCCAACTGATTTATTTGTCCATATTATATGAACAATATACAGCTCATAAAAACACTTTTTCCCCTCTCTCTTTTGCTTATATTATACGACACTACAAAAAAAAAATTAAATAAATACTCACAAAACTTTATAATAAGCTTCAACTTCCCTTTTTTTAAAAAAATAATTACAAAAA

>SlXTH9

TTCGTTTTGGATGAAATTATAATGTTTGGTCAAAATTATATTTTTTTGGCACATGACTCTGACATTGTTACATATTAAAAATTTGTTATGCTAATATATCCATGCATTATTTTTCATGTAGTTTTGGACCTATGTCGTATTAAGCACCGTCATGAATTATTTTGTACCAAATGTTTGGTATAATCATTTATTGCTGGTTGAGATGCTTCCATAAATGGTTTGCATGGTGAGTTCAACCTATTTATTTTAAGTCATGTAAAATAAAATTTCAAAAAATACTTATAAAATAATCTCACTTGAAATTTAGTTATATGTTTTTCTATTATTGTTATTGTTCGAATTAATTTTAACGTATATCAATCATTTCATTTAGTACTTGTTATTTTCTATCAGCATAAGTATCGAATATTTCTATCTAACAAAACTTAAGCAAATGAAAAACAAACCTTTTTTGAATCTGAACAAAAATCTTCATAATTCTCTTTACTTCATTAAATATTAGATCACCCTTTGAATTATCGGTTACAATTACAAATAAGTAATCGCACCAAGAATGCTGGTAACTATTCTCCGTTTTAGAGCTAGGTCTGAACAAATTTATTTTTCAGACTACGACATATCGAATCTCAATGATATACATATCATATAAATTGAAATTTTGCCTGACACCTCATCTTTAGAAGTCTGTCAGTTCTACTTGATCTGTTCTTATTAGCTCGCCCTTCCGTCAAGGGCATCACAAGTACATTTTCAAAAAAAAAAAAAAAAAAAAGAACCCCACACGCATAGAATAATTCTTGTATGTATCCAATGAATTTAAATAAACTAAGGTTTTCCAGCTCATAGCTAGCCCTTTGCCCATATTTCTCTCATTAAAAAAAAGGATCCTTCCTATTATATTTTACTTCACATTGCACCTATAAAAAGACAACATTTAACCAAACCAATAACATCAAAATTTTACATTATTTCTTTAACAATTTCATCATCTTAGAAGATC

>SlXTH10

AATAAAGTCAGACGCATATCACCGACATTGTGGTCAATTCTGAAACCAAAAAACATGATTTTGCATCGGTACCGAATTATTTTTTAATCTTTTATTCCTTTGTAGATCTAGAATGTTTGAATGGTTCTGATTATACGCGGCATCCACCTAGAAATTAATATTAATGGACGGATTCGAAATTTAAAGTCGTAATTATAATTTTAAACTTGTAGCTTCATATTTGTTATTTCATCTGATATGATTTATTTGTCTTGTTTAATAATTTTTTTACATAACATATTTAAGATTTGTAACATTCAACATATCTTTTATTTAAAATGATTATAAAATTCAAAAATATAAATTTATGCTCCGCTTTAAAAATATTAAATTATGAACCTGATATTGCAAAGGGTGAATACAATTAGCGTGTTAGGTAGAATAATGTCAATTTTGGGTTATTGGTGTCACCCAACCCATATGGATCTAATCCGGAGTGGTTCAAACATATTAGTGGTTTAAAGTAAAAATTAATTGGGATTTAAATTTAAATTGTTAATAACTTTTATATAATTGATTTTTTTAATTTTTAATTAATAATATAATTATTTTTATTTTAAATTATTTTTCATGAAATATAGTACTTCAAATATGTCATTAATTTCTTCATCTTCATGAAAAAATTTACTTTTTTTACAAATAGTATTCAATATTGTAAAAAATAATAACTTATAGTCCAATATTATTAAAAATAATACCATCTAAATTTAAAAGTCTATAACATATGTATTTTTTTTAACACTGTCGAGTGAGGTTTGATCTAATAGCTGCCCCCACCCACCATATACTTCCGAAACCGCAGCCCCCTTAATATCTCCCAAGGCCAAACAAACGCGTTACATTTCAATCCTTTCAATTCACAGCTATTTTGCTTCATTTTATTCAATTTAATTCCCCTTCTTCGATTCTTCCTCATCCCTTTATCAAACTTTACTATATATATTCTCATCTCAAAACACAC

>SlXTH11

CGAGTGTTACAATCTCTATGATTTCTCTGAATTCGCCTTCTCAAATATAAATTTAAGGGCTTTAAAGCTTGATCTTGAATTTGTGTTGATCTTGAACTTAAACTTGAACTTTATCTTAACTTTTACTTAAATTCAAGGGCTTCAAGCTTGTTCTTGAATTTGATGGTCTTGATTTTGATCCTCAAGGGCTTCGAGCTTGTTCTTGAACTCGATGGTGTTGATCTTGATCGTTCTTAAAACACAAGTGCTTGAATTCTTAAACTTGTAGCTTTCTAGAGAATTAATAGCGTTCGTACAACAAATTTTCTCTAGCTTCTTATTTAAAAGAATTTTCTCTCCCTTTTTTGAATTATGAGGATCCCTATTTATAGTTTTAGAATAGAAGAGTTACAATTTTAACATAATTCCCTTCGGCCAATCAGATTTAAGTGACATGACATATGAGTTTTATTGAGCGGGCTTGTCATCTTTGACACATGTCATGATTCTATTGGCCTCTTATTTGACTTGACATGCCACATCATTTGCCCCGCGTGGCACCAAGATAGTCTCCAAAATGAGTGAAATTGGTTCATCTATTGAGCCCAAATCAATTATTTTAACTTTAATTAAATCCATATTTATTGGACTTGAATAATTAGCATATTTATGAACTTAATAGAACCTACTCATTGTATAAATTTATATTTAATAAATTTTAAATGATTACACCATTTTTTTAAACACACTCTACCAAAGCGTGTTATTAGTTAAAATAAATATCAATGTTTTCCTTTTTGGGTAATTGGTGTCCCCACTCCCCATACAAACGCGTTACATTTAAATCCTTTCAATTCACAGCTATTTGCTTCATTTAATTTAATAATTCCCCTCCTTCCTCTTCCCTTCTATCAACTCTCCTCTCTCTCATATATATATAATATATATCCTCATTTATGTCCCAATTTCAAACATACATTCCCAAAAACAACTAACTACATATTCCTTAAGATTTCCTATA

>SlXTH12

CGATTAAGTAAATCTTTATCTTATTTATCTTTGAAAAAAATTCAATTACTTTTCATTCATTGTCCTTTTTGCTAACAAGTAGCAAATGGGTAAAATGACAGGATGTTGCCAGTAGTAAAAGTTGTTGGTCTTATCAACAACAACAAGGTCATAAGAAACTTGCTAACAAATTAAACCAAGTGTTAACAATAAAGGGTACCATTTAATAAAATAAATACTAGTACTTCTCATCTACTTATCCTGCCCTTTGAGTTATTATACTTTTGTCAGTACAATATAATTGATCTGGTTGGCTTCCACTTACTACTCCAATCATATAATAGAGCAAAAGAGTGTATAGGCCATACCAAATTCTTGAATCATTCTTGCAAACTTTATCTCTACTTCTTCAAACCCCCACCCCCACACTTCTTTCAATAATAACATTTCAGATAATTTACGTGCATCTTGATTATTTTATTTAAATATGTATTTTTTTTTGTTGCTCGACATGTTCTCATTAAGAAATTCAAATTATATATATATAATAAGCAGATTAAGAAGTAAAGGAGATACGGTTACGAATAATTTTGATCTTATTGATAAAATGTAATTTTTCAATTCAAATAAACCCCCTGCGTCCCTAGCTTCTACCTAACAATAGCAGAAATATTGAGTAACTTTATCCATTAAGACTATAACAAATTGAACTATTGCAATTTATATCGTAATTTTATTTATTTCATTGACCACTAAATCACGTCTTTAACTGACTCTATTTTTTATTATTCCTAACAAACACCAAAAAATTCTAAAGCATCCAATACCAAAACAAAAAAGGCTCCAGCTCTTATGTATGCTAAGCTGGCACCAGTTGTTGCCCATGCATCCCCCCCCCCCCCTCCAAAACCCCCACCCACCCACCCAAAAACTCAAGAAGTTCACAAGATTTTTGCTCTTTTCTTTATAGCTATAAGCTCCTTCTACTCACTATAAATACTTGCTCCCTTTAAATCTCATT

>SlXTH13

TGTTCTTTTTTCACCAAATTAAGGAAAAAACATTTAACATTTTTGGAATTATTTTTTTTCATGTGGGAACTAATATGAATTAAAGTCAAACATGATTAGAAAAATATTATTTATCTTATTAAAAAGTAAGAGGATGAGTCCCCACTAACCTACAACAACAAAAAATAAATAAATTAAAAATTAAGGGAAAGTAGTATTTTTTTTTTAATTTTGATTTCTCAATAGTTGATAATTTTCATCTTGTTTCTGGATATATATGACTCAAAATATTTCATTTTAATTAATCAAAATAGTGCATTAGGTCTATGTAATAATTTTTTTAATTTTTTCTAATTCTAAATAGGATTTTTTTATTGGGTGGCAAGTACTACACATAAATAAATTGATGATTAAATAAGATTAATCATATACTATAAATGAGTAGAAACTAAAATTTATCAATAACGAAAAGCAAAAAAATTGTTATTAACTCAATAATGAAAGAAAAATAAATAGAATTCTCCCTTCATTGTACATACAAAAAAATAATTAATGATTAAATTAGCTTAATCAAATATTACAAAAACGAAAATTAATATTTTATCAATAACCAAAAAAGCAAAATTTGTTATTATTAACTCAACAAATACATTTTTTTTTAAAAAAAGAGTTCTTGTCATGATGTGGACAACAAATAGCATATTCCATGTTGTAAAGAAAAGAATATTAAAATAATAATACTAATTAATTATTAAAAGTACAAAAAAAAATATATGTAATATTATATTTAAATATGAACATTTGACCACAAAAAAAATTAGGTGATGATGATTGAAGCCAGCAAGAAAGAAGGAATGGACATAGTTGTTTGCCAATATGTTCCCACTACCAAAGATGAAAAAAAAATATAATAATATAGTAATTAATCAATGAACAAAAAAAATCCAATATTTTTTATAGATTATATTATGAGTAATACAAAAACAAGAATGCAAGAGTTTTCAAAGATCAAATTAGTAAA

>SlXTH14

TATTACTATAAATATGAAAAATATAATTTTCAGAAAAAAGGAATTCAACTGAATTCCTTGAAATCATGTAGCTTTGCCACTGACTTTGACACTAACTTAACTCACATGTTCAAAAATTATTATACAAATCTTACTTTATACAAAAAGAGAAAATATCTTACTGTAAAACCAGGAAAAAACAAATAAAATAAAATAAAATAAAATAAAATAATGAAGTAAGTCTTACTACATGTAAAGCTGATGTGGGGTTAATAAATAATAAGTAACCTTTTATATTTGAAAATAAAATTATAAAAGAATTACTTATGTTGAAGTTTTAGTTTTTTATGATTAAACAGTAAAAATACATCAATAATTTTATTTAATGATGAATATACGATATATTATTAAGAATATCTCGTTAGCTAGGGGATATTTAGTGACAAATTATCAACGTACGTCATATATAATTTCTTATTCAGTTAAGCTCATACGAATAAAAAAAAAAAAAAAAATACTATAAAGACGAATTAGTGATGGATCATCAAAAATCCCGTTAGCTAATTCTTAACGATGAATTTTATAGCTAATTCCTTTTTTAATATAGTAAAATATATAATATACATATTGATCGAATATCAGGTAAGAAATAAAAAAAAATACTATAATAAAAATGAATTATAGCATGATTTCTTCACTACTATATTCCTTTTCATATATGTTACTTTAGTCATACGTACATCGAAAACAACCGCTCTAACTTCATACAATAGAAGAATAAGACTGCACACACGCTACCATCCACATTGACGAATTAGCGATAGATCATCAATAATCCTATTTCCGACGACTTCCAATTCTTACTACATTTAGTCATTCGTCAGATATCAAACACCGAATAAAAAATATATTTTTTAAAAAAAAATCTACATAATATAAATTTATAATATTATGTCATGTTGGGGTCCACACTCATCTTGTTATTTATATCAAAATGTATTACCATGCTTTCTTCCCTTCT

>SlXTH15

AAATTTCAAAACGAATATCGATCTTCGGTTAGAGCAAAAGGGTTGTATTGTTATTATTGGAGCAACAAACAAATCAATTAGTGTATTATAGAGGACCAATCAAAAGAGCAAAATCCAGTTGTCAAATTTAACTAGTTTTTTCTATTATTTATAAGACAAATCAAATAGAGTCATGTTCACATATGCAGGCATATTAGACCCCACTCACATTTTTCTTTAACAAAAATTCAGTGAAGTTCATGGGGGCATTATGGTCATTTCCACCTCACTCATCTTTCTGTCAATTGCTTTCTCCAGCTGTCGGTCGTTTGGATTATATGTAATCGTGGGATAAATCCCTAAATTGCCCTTCTAAAAATTTAAACTTATATTTGCCTCTTTATAATGAACTATTAACTTTTGTAAAACAGAACAGCTATGTGTAACGTGTTTTTTGAGACATCTTCATTAAATCACGTAATAATATATTTTATATTGGCTAAATACTGTATGAAAACAAGTTTTTTCCGTAGTTGAACGTCATTTTAATTTTTCTTTCGTGCCCTTTGCTTTGGTTATCAAATTGTTTGTTGTCGCCACTACTTCTTGTTTCATTATTTATATATCTGATTTGATATGTTTTACTTAATTTAGGATTTATGATAAATAATCTTTTTAATGATAAAATTATGTATATTTTATCTTTTTTTAAATTCTATTTATAAAATTAAATCAAATATATTATTGAAAAAACTATTATTCTCAAAATAGGTGAACATAACTTTTTGGACAAACTTCAATGTCTCCTACTAAAAGGAGAGGATTTTTTTTATTTTTTTATTTATATACTAAGGTTAAAGTAGTATAAACACTAGAAACAATACAAAAGCAACAAACATGGAAAAAAAAATTCATTTCCATTTTTGCCCTTGACATGACCAAAACTAGTGATCAATGATCTGTACTTCCTTTATTTTATTTTATTTATTTATATTTCTTCTCTAAAAGGATTCAAACTTA

>SlXTH16

NNNNNNNNNNNNNNNNNNNNNNNNNNNNNNNNNNNNNNNNNNNNNNNNNNNNNNNNNNNNNNNNCGCCCCCCTGGACCCCCCGACTCGCTGACCGGGCAGCGAGACCCCCGTCCTTTCTGTTTGTAGCGGGTCCGATTCAAGGCATTCAACAATATTAATCATAAAAAATACAATATTGCCATAATACATAAATATGTCTTATAATATGGATTTAACGATACGCCAACATATAAACTTCAACCAAACATATAAAATTGATCAAATAAATACATATGAAATACGTGTGTACTTATAAACCTTTCTTGTACAAATTAAAAGAAGTGAGTGTCTTTTTTCACTACACGCGAATAGAATACTTAGCTTTTACAAAGGAAAAAGAATTTCCCTTTTTTTAAAAGAAAAAAAATCTGCTAAAGCCAAAATTAATAACAACTGTACACTGCCATAACCCAGCTGTAATATTCCACGTGTCAATTTCTTATTAGTCAAGTACAAGTACTCATCCTTTTCTCAACAGTGACTACACCTACTGTGCTGTTCCCCACCATGGGACCAACTTTTTTTAAATTCCAGCTAAGCCAATTACTGCTAACGGCTATACTCCTTCCGTACGTTATTATCTTGTCTATTTTATTTTTAAAGTTAAATGATAATAATTTTAATTAGTTTTTTAAGATATATATTTTTGATGATGTTAATATGTAAATTTTTTTTTGTAATTTATAGTACTTTTTATATAATTTTATTAGTATATATTTATTTATTAAAATACTAAATTAATATAATTTAATTTAACTTTAAAAATTAACCAAATTAACTTTTGAAACACGACAATGACACACCAGACGGAGGGAGTAATATTTTTTAATTTTTTCACTATCTGACACATCGACTATCTTGTCCTACGTGGACAATGACGTGTGACTTAAGTGTTTGTTGACGTGGCAGTATGATTTCTCATTTTTGCTATAAAATGCACTCCATTTCACCTTTCTTTTT

>SlXTH17

TCAAAAGCTATCAAATAATCTTTTCGGGAGATAATTATTTAGTAAGTGACTATTTCTACGAGCATTTTAAACTACGATTTAAATATCTTTTTTCACCAAACTAAAAATCTTGTAAAAATGTTAGATAAAAAATTAAAATATAACAATTAGCAAGTCATTAGCATCATCACAAGACAATCGATTGAGTAAACATTAGATCTAATCTAACATTATCCAGAAGAATAAGTTTTTTGAGATTTATATTTACATAACTTTTTTAAATATAGAAATAAAATCTAAATGGTAAACTAAAAAAAAAAAATCTGACTAAGATTTTGGTAGTGCTTCTGACAGTTATTTATTAGCTAAATTGCAAAAGAGACTTTCCTTAATACCACAAACATTACAAAATCGCTATTCATGGAACATAAGTGCTTTAATTTTAAGCTAAAACATAATGGAAAAACCTCACGTATTCACATGACATTTTCATATAATAATAATATTAATATTAATATTAATATTAATATTAATAGCTGTATCATAATAGGTAACGACTACTATAATTCACAAGGGGAAAATGTATCTTCAGATGAGCTCATTTAAATTAAAATATTAAAGATGGAGTGGAGTCAGATAGGATCGAGAGGTACATTCAAAAAAAAAAATTGATAAAAAATTACGCTATTAATATATAGAAAATGTCAAATTTTCTTTGGCTTCTTTATAAATTTACTTCACATTTTTAACCGTCTTATAGAAAATTCTGACTTTAACATATGATAATATAAACCATATACTAATAATCTATTATCAAAAACAGAAATAACGCGCAAACATTATTCTAGCTTGCAATGAGAATTATGAATAAATTAAAGTTGTTATTTAATTTCCAGCTCAATAATTATTGTAACCCAATTGCCCATTTTCCTATCATGTTCATAAAGGAATAAATAATTAATAATTAATAATTCCCTTCTTTATAGTCCACCTATAAAAAGACTACTAAACCAATCCAAAA

>SlXTH18

ATCATATCTGAATTTCTAGCTGCAGTCCTACGTACAAATTCTAATTTCACAACACACGCGTTACATATATTATCCGTTGTGATCCAATAAACTCTTATAATTTTATCATTCGAATGACATTGTCATCAAAACTAAAAATGTGTGTACCATTTAATTTTTAATATTCCTTATTATTAAAGTTGTTCATTTTTCTCCTATATCTCGATATATAATAAATGAAAAAATGATAGAAATTCGACATTTAAGTTCTCTTATAACCATTATCCCCTATTAGTTTTATAAATTCCTAAAATCTCTCATTTCAAGCATCAGATTAATGTATAAGAGCATCAAATTAATGTATCTTGCGTATCAGATTAATGTATCTCACGCAACAGATTAGTGTATCATATATAAAATGTACATCATATTAGTGTATCATGTAAAAAATATAATGCATCTTACTTAACGATTAATGTATCTCGCACATCAAATTAATGTATTGGCGCTTATATTATTGTATCATTTTGAGAGATTTTTGTAATTATCAACTTATAAGGAACAAATGATAATTTTTTCTTTAAAGTAAGTGATTTCTGTTTTTTGCCCTAAAACTATATAAAATTCACTTTAAGTTTAAAATGCACCTTTTTTTTTTCAAAAGCTTGTAACTATAAATGTCAATCATCAACTAAAAGTTGTTTCCCAAAATACAAAACATATATATTAAACTTCAATTTAAATGACTTTGTAACCATAAACTTCAATCATCAATTTAAAGAAATATTCAAACACCATTAAATGCAACATATACATGTAACCAAATTTCTTGATTAATTGTCAACTGCATCTACTTGGTATTAGTTATTATGTCAACCTCAAACATGGTCAGCAGTACAAAATACATAAAATTACACATACTAATTCAAAGTTATTTTTTTAAAAATAAAATAAAATTATGCTATATAAATCCACCATAGTCTTATAACAAATGTACAAATCACAAAAAATAATTTTAAAG

>SlXTH19

AAATTGTGACTTTTATAAAAGGTTGTGACCTTTTCGAACGATTGTAACTTTTTTAAAGAGTTGTGAACTTTTCGATAAGATACAATAAGTATTTGTTGACACTACTACGCTTTGTTGTATGTAAATAGAAGATTTTCCTCTCATTTTAAAATAACGAAAATTTTGAGATTTTTTCTTCTTCTTCTACACAACTAAATATTTGTGTACTTTCTTCTTATTAAGTGACTCTGACACCATTATTTTTGTATAATACATTTGTGAATAGAATTGTTCCATCATGAGAGGATTTATTCCTTTAAACCTCAGGTAATAATAGAGGGGGAAAATTTCCTCTAAGATACACTAGACTTACTTTTTTCCTTTTGTTTCATTCTATTGTTATAGATTATAACTAGTATATTTTTTACATTCGTTTGTTTATACTTATGATACTAATTATTATATTTGAGTATATGTTTTAAATTTTACTTTTTATGTTTCTACAAAGTTATGTGTTGGTACATATAAATATTATTTATTAATTTAAAAATTTAATTTCTCTTGTTGATGTTCATCTAATTTGCCAAAAATATCAAATAGTAACACTTCCTGGAGGCAAAATGATAAATTATAAAATCAAATTAAAAATGATTAATTGAAAGATAGATTATAACATGTTATAATTATCATTTTCCTTTACATTGTTAATATTTATTACTACGTATTCTTTTATGTAAGATAATATGTATAGTGTTTTAATTTTAATGACGGATAGATTTTAAATATTATTTTATAAATTTGGCAATATCAAATTCTCGCGAGAAGCGCCAATAATTTTACTAATTTCATATAGTTATAAATTCGTAATGAATTATGATCTCTAGTTTTAGGGGCCAAAATGAGACATGTACAAAGAAAACTCACCAACCCCCACACAAACTAGTCCCCACACATCATATATATCTCAAGGCCCTCCATCTATAACATAATAATATATTCTATATAAAGCCTAATTGTGTAC

>SlXTH20

GGATGACACGTAGCTAAATGTTGTTGATGAGTCATAAAATGGTGAAATATAAGTCAGAGTAATCAAGTGATCACTCGGCACTGGCATCGAATCGTTTGATATTTATATTAAAATTTAGTTAAATTTAAAATTTTACAAAAAAAATTTTATATAAAAATAAAATATTTTTTAATAAAATAATTTCATATTTAAAAAGTTGTATATTCATTCTTAATTCAGAATGAAGAAATATTTATCATATCGTTTTTCTTACTTCATCAACAAGTTTAATATTACTCACTTTTGTTTTGTACGTAATTTGTATCTAAATTTTATTTCTATCTTATAAAATAAAGAGAATACTTTTCTAAAACGTAAAAAATGAATAATAAAAGCAAAATTGAAAGCAATACATATGAAATGTTGAAAAATAAAAGTATTCTAAATGAAAAATATATTTTTTTAAATTTAATTTGGTATTTGGTAATGAATATAAAAAAAAAGTGCACGTAATCTTACAGAATCAAAGTCCAAATGTTGTAACATGCCATTTTTTAATTAATTGTATTACCTTCCAAGTCCTTTTATTTTTCAAACATAAAATGGATTAATATTATTTTACCATTAAAAAAATAAAAATTAAGTATAAATTTTTTTCAAAAAATTACTCATAACTAATAATTGATTTATAATATATCACCAATTTATTACTAAATAAAATTAATACCTAATTCTATTATTTAGCTTCGAAATTTATCCATCATTAATTCTTATTTTTATAGTAGTGCTCATTCATTCAAAAATATATTAGTTTTAAAAAAATTGACAATTTTTTTAATTTCATAAACTTACCTTATCTCCTCATCTACATTTTACTAATTAATAAATACTAAATAGCAAGCTGTTGCCAACTCTTTATTTTTCAGATGGCATTCATTGAACCACCTTTTGTGGACACAAATTTGTCTTTTTTTTCATCTAAAATTCACTATAAAAAGGCAAATTATATTCATTCCACTTT

>SlXTH21

TCTATCCTTGCCATTTATCAAAACAAGGATGGAGAGAAAAAAATACATTTCATTTTAGTGCTAATTTTACATCTTTCATGCTTTTCTTGTTGTCCATTTTTAAACCAGTTTTTATTTTATAGACTTATTATTACAAATTTCCTTATTGTCTCAAAAAGAAAAAAAAAACAGTTGGCAACTATTATAAAATTAAAAAAAAACACTTTTTAAATTTAAAATTTTACCCTAGTGTTTTTTGAAAATACAACACCCAAAAAATAAAAGAAATTCCAGAATAATTAATTCCTTGCATTAGTTGTTTATCTTAGTTGTAAAATGTAAAAGCCAAAAAAAAGGATGAATCAATTTTCCTTTTTCCCCAATTTCTAGCCTCTATTTTTTTTAAAAAAATAAAATATCTTTTCTTGTAGCCACTTAATTTTTAAAAAATACAAAGAGAGAATCAACAAAAGAAAATTAATCCATTTTCACAGCCAGAAACCCCCCCCCCCCCCCCCCACTAATTTCATAGGAATCTAATAAAACTATAAAAGGCTCTTTTTTTTAAGAGAAGTTTATATTTAAAGATTCCAAACTATCACTATTTATATATTAGAACACATTTTTAAGTTGATATCACCAATTATAAGTTTTTTTTTCCTCATATCACCATCTATTTAATCTAATGTGTTTTTAATACATAAATAATGCTAATTTAGATCGAACAAGTTATCTGATAATTGAAATTTTAACCAACTAAACTATTAAACTATTAAAATTTCTATGAACAAGTGATGACTCCATATAATTAAATGAAATTATGTATATATTGATTTATAGTAAACACTCGTTACAACTCTATTTAAAAGAAAAATACTCCCAAACAAAAAATACTTTTTTTAAACCAAAAACTCAAAATCCAAACTTTTTTTAATTACTAATTTTTTATTTTGTGAGTCAAAAACTCATACCTAAAAGTCTCACCACCCCATAAAATATAGCCTCTCTTCCCTTTTTTTTT

>SlXTH22

AATTCATTTTGGTGAGCATAAATCATCTCACAAGATGATAAAATTTAAGTTGAATTATTTTTAAATAAAAAAGATACTATTATTCCTTTTGAAATTAAAAAAAAAAAACACATCACGTAATTTGTTCAATACATAGCATGTAACAATTCTGCAGAGCGGACCCACATTCATACGTAAGGTGCACGTGCATCTGTTAACTTTGAAAAAATTAATATATGTATATATGTATATCTACAATATAGTTAAACAGATACGAAAAATATAAAATAATCTTTATACCACGAATACAAGCTAGAAGAACTATCATCATGAGATCACGATTTAACTTTTTCTTCTAATAAAATTCATATTCAATATAAATTCAAGTAGAGTCGATAAATTCCGAACAAAAATTAAAACCAAGGTCTAGCTTATTATTAGGCCGGTCAGCCGGCTAAGTCGAGTCTTTCCAATAGTCTAATGCACGTTATTACATAATTCTCAAGCCGCGTCCCAACTGTATATGCTTTGCCACTTCTCTCTCTTTTTACGTATTGTCGTATTTCATCTGTCGTTTAATATTCATATTAAAGTTCAGTCATATTCAAAATTATATATCGTAAGTCTCATTTTCTGAAATTGACGTTCCCAATACAATTATTTTTATTTTCAATATTCATATACGATAAATGAATTTCAATTATTCCACCTCGATTTACGTGTTAGCTTCCATATTATTCATATAATTTCATGTTCCTATTGCTTAAAATTTAAAATACTTTTTCCAACCCCCTCCACCTAATTATTTCCCCTTGATCCATCTGTACTAAGCAAATCAAATCAAAGACGTGATCAAATTAAATATAATAATACATTTTTCAAGGGGGTTTTCTTGGAGTAGTATTATTTGATTATGAACTATATATAAAGATTAATAAGTATGTCATATTCCTAAGAACCAAAACACATTCATTAATTTTTTTCAAGAAATTTAAGTTAAATTATCCCATTTATGTTCAAA

>SlXTH23

AAATTACTTACTTTTATTAAAATTCAAATATATTGAGTAGAGTACTCGTCATCTCTAAAGATTTATATTTTAAAAAACTTAATTGTCAAAAATATTCTATGAACTTATTAAAATAATCTGAATTTGTTCTCCATCTGTCTGTAAGATTTCAATTGTTTTTTTATATTATTTTTTTGATTTAGGGAAATTGGAGTAAGATAGATAGATACAATCTTCTAGTTTAATTAATTTGCTAGTTATTTGAATTTTCACTTTTTTCTCATCTTATAATTTTCTCTCCTACTAACCCTGCATCTTTCTTTTCATTTATTTTTTAAAAAAAGTTCAATTGATAGAAGAAAATATTTTTTATCTTAAAATTAACATTAAAGACAAAGATTTAACAAGTGGAAGGAGGATTTTTTTAAAATTATTTTCAATAGAGTAGTTTTTTCCCCTAGGAGAGAAAACATAAATTATGTGATGTTTGGATTTTGGTATAAATTTAGACTTAAGGAGATTATTTTCTTATATTGCATTTTAGGAGAATGCATTATTTACTATACCATTCATGTAGTAAAAAATTTCAAAAATGATTCGAATTTGAACGCTAGGTTAGTTTTGAGCATATGACTTTGTCGTAGCGCTGTTAGTGTAAAAAATAACTTTATATTAATATTAATAAGATTTTTAATCTATATATAATGTAATTTTTCAACAACTAACATCCATTTATATCGTAGTTTTACCTACAATTAAATTGTTTTGGTTTCTGAAAAAACCATTGGATTGATATTTTTGAAAAGCATTCCATTTTTAGAATACCAAATGGTGGTTCTCAATTTTTGAAATATCTTTCTAGTCCAAATAGGAAAATATTCTCAAATGAAAGCAGTTTCTTGTTCGTTCTTTTTTGTCCTTGGCCTTATATTTCGTCTCTCGAGGTAAAAAAATTATTATTTCCGATTTTGCCCTTAGGTAACAAACCGCGTATAATTAATAAAACGTTGTCATTTTTAAG

>SlXTH24

TAGCAAAAAAATTACTAACATGATGTGGAGATTCGAGATGTCAAATAAGAATAATTGATGAACAAGAACAAGAGAAAATTGAACAAACCCAAAAGTCACAAATATACAAATATAAGGAATTAACGAGTCTAACGCTATTTTTATTTAACTATAATTCTAATTGATAATGAACTCAAAAGATTTTGAGTTTTATTTTAATTCTAAAAAATAGTTAAATTTCAAATGAATTAGGGTGGGTCGCTAGGTTTTTTTTTTTATGAAGGGGACCTATTATTTCGAATTTAAACAAAAATTAATATATTTAAGAAAAGAAAAAAAGAAATTCCCGTAAGGAATCAAAGATGGGAACATTCTATTACAAGCAGTGTTCTTTTAATTGTTTTAGCGTTTATCTTTTCATTTTTTATATAGATATATTTTGTAAAAAAATTTAATATAGCCGTGTCACGTGATACACTTTAGGTATAAATATATTTTCCCCTGCTAATTAGGTAGGAAAATAACATTGATATAACACGAATGTGATACACCTCTCATTGTGGTGCTATTTGTTTGAAATAAATAAAAATTCACTTTTGAACAATATAAATATGTAATAATTCACCATAATAATTTAAAAATGTTTGAGATAAAATTTTAAAATGAATTTATTCATTTTCCCACAAAATGCATTTAGGAAGGTGTACATAAAAAAAAAAATGAAATGGTCAATCTGAAAAAAGGTGCAAAAATTACACGATCGTAGCAGGAGTAGTGTTGGATTAGTAAAAGGCCGCGGAATATCATATATGGGCTCATGTCTGTCACTTAGCTTTGATGCCGTCATTTTAATCCTGTTTCCAGCTCAAAATCTTTATTTTTTCCCTTGTTTTCTGTTTTACGTTGAACCTTCCTCTTTACTTCCTCAAGCCTAACCCCACACGCTCTGCTCTACCTACTTCTTTATTGTTGAGGTGGGGGTGGTATAAACCCTTATAAAACCCCATATAACTATCCAACT

>SlXTH25

TACCATATATATAAGTAAAATGATACACAAATAATTAAATAACATCTTTTGGACACTCTTATAACACAACAAGTTGTTATAGCCTGGTGATTTTAGATGTTTGGAATGAGGTAGTGTTCACATGTTTGAAAATTTCACAAGTCTTATTATTGGACATCCTTTATGAAATTTTTAACTCCGCCAATAAATCACATAACTCGCCTACGGTGGCTCCACCTACAAACGTGACTGTAGAGGCGTAGCTTAATGGTTGTCAAGGTTTCATTAGAAAAAAAATATCGTAATAATACACCAAATGATTAAATAACATAATTTGAACACACTTATAATATAGCCTAATAATTTTAAATGTTTAAAATGAAATAGTGTTCACGTGTTTGAAAATCTCACACGTACTATTACTAAACACCCTTATAAAAATTTTAACTCCGCCCAATAAATATATGGAATACAGGCACATTAAATATATTAACTTTTAATCTAGCATCAAAGAATATTGATCCAAATTCATGACCCCATTAGTCAATCAGTCCAATCTCAATTCAAAAAAATAAGAAAAAAAAAACCCTAATAAAATAGTACATGAATATGTCAATAAATATATAGTATTCATTATTGCTTTTACCTAACAATCAATAAAAGTCAGCAACTTTTGTTGTAGCATGGCTCCTACTTGAACTCAAATTGGTCCCCCAACACAAATAACAATAATAAATTTTTCATTCTTAATTAAAAATTTTACTGAATCAATTAGTAAACATGACATTATCTGTATGACGGATTATTTAACCCTTCAATATAATTTTTTTTTAAAATTTAGTGAAACAAATATCGAATATTGAAATAAAGGAAAAAAGGTCTTTCCAAAAACATTTCTTTCTCTATAGCACATGAGCTTTCTAATCAGTGTCCACAAATCTATTAATATTATTTAATTAGATTCTTTATAAGACAATTACATACAGCTTGCTACAACTAAGCACAATGTTTTTTTTCATCT

>SlXTH26

CGATCATTTAAAGAAAAGCAAATCTAGCTATGTACTATTTAATCATGAATAGAGAAGAAATGAGATTGAATAAAATGTAGAAGGACATGACTCATGACTAAATTACTTAAATAATGTGCGATAGATATTCACATATTGAGATTTGATTAAATTTATTTACATATTGTAGGATTCATTTTTAATGCGAATATTTAATTTAAAGATGAGTTAATCTCAATTATGTAGTATATATGAGATTACACTCTTAACTTTCATGACCATATTTAAATTTGATATTTTGATTGATATGTATGGAGGAGTTATCTATTATCTTAAATTAATTTAACCTAATAATAACATTATCTTTGGAACCAAAACTCGATTTGATTTTACAATCCTTTACACATAAAAGAATTTTCTTTTTAGAAAACATTATCTTCTATTTCTATTTAAGTTATAAATAGTCATAATAGGAGTATATCATTTTCATCAAACTTGCAATATTACATTCCAATTCAACTATGTTTTGGTTATTAAATAGAACTTTCTTTCATATTACTCCACCATCCCAAAAAAGTATGATCCTATCAAAAATTAAGTCTAATAAAAAAAAGTTAATAAGTACATGAATTAGTGTAGTTTATTAAATTTATCAAATATTTATCGAACTTTTCTTTTAAAAACAAGAATATATATGGAAAAACATGTTGATTTTTACTTTATTCCCTTTTTATAAAAATATATCACGTGGCAATCTAGTAAATAAACCCAACTACGAGGACTATTCTATATTCCACCCCTCAAAACCGTAACCTGCCAAATCTCTCTGTTTCCTCCTCTCTTTCTTCATGGCTGCATATTCCGTTACCATTTTTGCGAATCGAAGAATCCAAAAGAAGAAGAAAATGAAAAGATTACAAAAATTTTTATAATTAAGAGAGAAGAAGAGAAAAAATCTAACGTATATATATGTATATATAGTAAAAAATTTCTATACAGAATTACAAAAAAAATAATAATC

>SlXTH27

GCAAATATAATCCCCAAAGACAATAATAGACGCGGCTAAAAAATATGACAAGGATAAATATGAATAATATTTTAAAATTGAGGGGAAAATATGAATAAATTAATGAGTAATTATTTACCTTAGCAAAATCCAGTTGTTAAATCGAGAAGTTTATATTATAAAAACTAAACACTAATCTAATGCAGTCATGTTCATTTAAAGAAGGCATCTTAGTGCTGACTTTCTTACAACTTGTCATTTATTTTTTCTAATTTTTCTTTTTTATAAACGAGTGAACATATTACTCCATTCATTCGTTTCAATTTATTTCTTTTATTTTTCGCTTTATTTTACTTTAAAAAATTATTTTTTTAGTTCTAACTTTATATTTAAAATATTTAAAATTGTAAAATTAAATAATATTTTAATATGTTATATAATCTTTTTAAAATTTGTATCTATTAAAAATTATACAAACAAAATAAAGTGTGGAGTACTTAACTCCTCTTATCAAGTTATGTTGCATTCCTTCCTTTTTAATCTATTTCTAAAGGAATGCTACCTTTTCTAATTCGAAACAACTTTTTTTATTTTTTTTTATATTTAGCAAAATGTTTTATAACCACAAATAACTCTAATTTATTTTTAAATTACAAAATTTTAAATGTTACATCAATAATATCACATATTTAACCATCTTTGTTTTATAAGGGCCAGAATCTTACTTATTCGAAATTATTTTTTTTTAAAGAAGGAATGTTGCTTCTCGTCATATAAGCTTACAAAAATAAATATAAAAGACAATCATAAATATCAAATATAGTAACATGAGTCTTAAGCATTAAAAAACAACCTTTGGTTTATTAAAGATGTTAAACAAACATAAAATATTTTATTTCCACACTTTGCCATTAATATGAGTGAAAAGGACCATTTATTAGCCAAGAATCCTCAGTTATAATTACTCTTCCAAGAAGAATTTCAATTGAAGAAAACAAAAAAGGAGAAAAAAAATTGTTCA

>SlXTH28

GGCCTGGCAGCTTAACTCATTCCTTCTTCCTATGGCCAGTGTGTAGCCGCCAGGGCTTAACTCTAGTATGCGTAACAGGAGGTATATTGATTGGGTGAGTCATATGAAGAGATTGCAGTACTATTTAAATGTCGCAGAATTTCAGATTTTTGTTTTGACTTAGCACTTATCACCATGATATATTTTTAAGTAACCTGAATTTTCTATATCAAAGAGTTCAAAGGTGATTTCATCTTTCTCACCCTGTAATCAGTACTTCCCATGACTATGTTAAGGATAGAGGTGTCCAGTCATCTTCAAATCCTGGATCCACCTTTACTTGGACCCGTAATGTGCTTGCCGTTTGACTTTGGCTATTTTTGAAGATACTTCATCTAGTTTCTCACTTATAACTAATGGAATTTACTCACTGCAATTCAACCAATTTGAAATCCCCCTAACTTTATGAATAAGTAACGGAGTGCTTATTATCCTACTACTCTTCTGGGGATTTGTATTGCAGGTTGGAATAATTTATCCTTGTTTATTCCCTTCTTCATTCAGTCACAAAATCATAGTGCGTTTTCTGATACTTTAGTGGGTGGGTTGTTGCCATTCATAACTTTTGGCAGTATATATATACTGTATTGTTTGCTTCACTCTAACTACCTAATAACTAACTTCCCACTTGTTCCTCCCTCCCATAATGTCTTCTAATAAAGACTGTCATTGAAGTTTCCACTGTAGAATATTTACTATAAAAACTTGTAGTTAGTGCCTTAGTGGAGTGCAGCAAAGAATCAGTTTATTTGGTAAAGTAAGAATATATATAGTAGTAAACAAGAGAAGAAAAAGGCTGCTTTGCTGACAAACAGTCCACTGTACCATGGCTGCCACCAAAACATATTCTCTTGCTTTTTGTACTCCATGATACTAATATAAAAAATATATTGTTTAGTAATACTATGTTAACAACTTTTATGTAAATAAAACTAGAGTGGCTCCAAGTTCTTGATTGGCG

>SlXTH29

TTAAATAATTTGTTATTTAAAATTTTAAGACAAAAGTAAATTTTTTTCGATTGTATTCTTACTCGTAGTTGTTCTTAAAGACTACAAACACTTTAATAGAACAAGTAAATGAAGAGATATTATCTTGAGATATAAATGAGGGTACAATAGTCAAACTTCTCTCCTTATTAATATTTTTTATAGAGCACGTAAAAGAAAAACACACAAATAATTTTAAACGAATGAAGTATTTCAACACATAACAAGATCCTCAAAACGCATATATACTGACAGAGATAGAGCCAAAATTTCTACTTTACGATGATAATTGAAATCTTAGAGTGAACCTTTATATATCAAGTGGTAAAATTGGGTTTTACATTTCATATTTGTACATATTTTAAAAGTTATTAAATACATATATATGATTTAAGCAAAAATTATTAGATTCGATCAAATCGTATGATTACACCATCATCAAACCAAAAGAGCACATACTCTATGAATTTTCGATATGCCTTATATTAAAGTTCTTCATTGTTCTACTAGATTTCGACATACTATTTTATATAAGTGGAAAACGTACCTTCAAAAGCTTATCATTTCATTTTTTTTTACATATTTTTATTAGTCCGCTCTCCGTCAGAAATGTCACACAACTTTTTACATAATAGTGTAACCATAAATGTCAATCATCAAAGTAAAAGTGGTCTCCTAAAATACTATATTAAACTTCAATTATCATCACAAATAATCCTTTTTTTTCTTGCTAAAAAATACTAATTAAATACAACATACATTTAACCAAATTTCTTGATTAATTTGCCAACTACATCCACACGGTATTTTGGAATGTCAACTTCAACATGGTCAGCACAAAATTCATAAAAACAAGTTTACAATATAGTAATTGAAAAGTTCCTTTTTTTTTAAATTTATTTTTTTTGCTATATAAACCCTACATAGCCTAAGAAAAAATATACAATTTTCAAGTGTGATCACTCACAATAATTAATTAAAG

>SlXTH30

ATGATCGCGATTTCGAGGGAAAGAAGAGTGATTTTTATGCTCGGTTACCTGTTGAATCGAACTCAATGGATGTGGATCGAGTGCTCAGGGAGCATGATCGGCATATCCAGGAAGTTGTTGAACGCGCCTAGGTTGAGAAGAAAATGAGAGCTAAAGTTCAGGTTGAAGGCTGAACGGAAAACTTGGTGGACAAAGCTCGAATCAGTGACTTACAGGTCACACAGAAATAACTTTATCGTTACTTCAAGACTCCCTTCAGACCTGAAGGCTGAAGTAAGCAAATTAAATTACAAATTTAGATCATTTATATTCATCAAGGAACACTCTTTATGTAATTTGCAATCTTGCTTTGAAAACTTTTTACTCTTTTGAGCCGATGGTTTATCAGAAATCGTTTATCTACCCCATGAACATATGGCTAAAATATGCATATATGCACATCATACCTCTCTAAACGCCACTTGTGATATTACACATTTACATTTAGTATGTTGTTGTTCTTATAACCGTAAAGTCTTTTTGTTACTTATAACAGTAAAGTAACTATAGTGAGTATAGTTAAATAATCTCTTTGTTATAAAACTAGGTGAAAAAAGAAATTTCGCTTAAAAGGGTCCAAACTATAAAGAAGTAAAAACAGAAAGAAGTCAGGGATTCAACATCTATATATATATTATGCTTTAGACATACAATACAATTTTTCATCGAACCCCCTTGTGCTATTCTGATTCTGCCCCTAGTTATAAAGGTAAATTTTAGTTACCTTAATGAGAAAAAATACTCTTTTTTTTTGTTAAAGTAGATAAAATTCAGTAAATTTACTATGAGTAAAGTTCAGTTACTTTAATATAAAACTTTTATCATAGCTATAAATTTTTCAGTTATTAAAAGAATATTTATTTATCTAATTACTAAATTTAGGTTACTTTAGAACTCTTCCCCTATTAATTCAACACAACTACAAGAAACAAGTCTCAGTGCAATTTTGTTAAATTACTGA

>SlXTH31

AGGGATGTAGAAGCAAAAAATGTAATTTTGATTCAAAAACACCCATAGAAAAGTGTTAAACGTTGTTTGGTGTGTAAAAAATGATTTTGCAAAATCGGAGTTAAAAAAAGGGCATGAATGAGTATTTTCTTTAACAATAATGACATAAATAAGCCAAACTTTTATCGAATGACATAAATAAGCCCTTTTTAATAGTTTGATGACATAGTTGAGTCTTTTCCCTTTAATAAATAATAAATAGTACGGGTATTATTATTATATTATTATTCTTTGTTTTATTAAATTTAATGCGTTGAGAAATGTGTTAGTTGATAAATAGTAGCAAAGATAAAATAGACATAATAGGTAAGTTATCTCTTAATTTTCTAAATTGAACAAATATTATTAGATAACTATTTTTAATATAATGAACATCTGTTGTTGAACATACGAAGACATTATAAATATAGATTTCACATCATTATGTAAATTAGCAGTTTTTTTTCCTAATTTAAACAAAATAATTTTATTTGTTTAATATTTGAATTCGGCCAAAACAAATCTTTTATACTCAAAATCTTCATGTATATATCCAAATTACTTTTAAAAAATTTCTTAATAAGACAATGGCAAAACTAATTATTACTTCATCTTCATTTATGTATCCAAATTATTTTTAAAATAATTCATAATTAAGTAAGCAAGTATTCATGCTGACGAATATAATTCAAATAACTTAGTTATAATCTTTAACCATACATTCAATCAAGGAAGTAAGTTAGGAATATTAATTAAACTCTCATTTTATATCATCAATTTTTATCTAATTAACTATATATGGTAGGTTGATATTATTATTATTAATGGTGCTTAACACAATCTCTTAATTTAAAATTTGCATAGATTAACATACCATAAAAAACACTCACCATAATTATTTGGCATTATAAATATGATGAATACTTAATTAGCAGTCTGTAAAATTGTCATTAGTTTAATGGCAAGTTGTATTTCTCTCTCA

>SlXTH32

AAAGATCTTTATAGTAAAAAATATAAAATATATAATATATGGTTTTTCAACTAGCTAGAAAGTAATATGTGAAAAGAAAATAAGGAAATATTACTCATTGGTTCTTAGAAATTTCTACATGAGCCAATAGAATATCCTAGAGTTTTCCCCTTCCCACTTGCCTATGTATTTCCGATATATTATTATTTTTTTATATTGAAATTTGATTTTTTAAATTTTAAATTTTTTATGTATAAATATTTTCCAAATAAATATTGGCATTATGAATTACTTTTTGTTTCTTAGATGAACTAATTCTGTGAATATTTGTCTATATTATTGGTGTATACATATATATATTGCATAATATTAAAATTTTGCCACATCCAAAAGAAAAGTAGGTGAAGAGTTATATTAGCCATTAGATTAAGTCCTTTTATCTTGTTAGATAATAAGTACTTAATTAGTTTTACCAACCTATCTATTTAAAACCTCAAAAGTTAATGTTTTTTAGGGTTCCTACTATTTTACTCTTGCGATTTAAATTTAAAATTTTTAATTAAAAATAAATTATTCATTATATAATTTTTGTAAAAATATATTATTAAATTATAAAGACTTACGATAATAATAACATATTCAGTAAAATTTTATAAAGTGAAAACGGTTATAACTCATTTTTCAAAATCTATCAACTACCTCAAGGCAATATAAAAATGGTGAAGGTGTTTATCTTCTCCAAGAAAACTAAAAAGTCCAAAAAAAATCATGTCAACTTTATGAATAAGAAGAATTTGTAGTTTTGTTTACATGATGATGTGTAATGATGACTAAACTATTTTATAAACTTTGCTAAACCATCAAATTAAGCCACATCAGATACATTAGGGCCCACACACTATTATAGTGGATGCCACCTGTCCTACTCAATTACCAAAATAGCCCCTCCAATTTCTATGAACTTTCTTTAGGTCCTAACAATTTTCACCCCTATATATAGTATATGGGATATACAAAGTTA

>SlXTH33

GACTTTCGAGATATAATTAATTACATAATCAATTCGTCATTAATTCTGCCTCTTGAATCCTGTGACTATAACTAATTTTTGTAATTGTGATGTTGGGTCGGTTTAAATGCATTTTGACTAATTTTATAAGCTAATTTTTATTTTTATAAAATTGTTGTACGTAATTTATTTTGTCTTTTTATTTCTTAAAAGAGTGTTTGTATTTAAAATCTCATAATTTTCTATATTGACCAAACAGATACCTTAAGTGCCACTTTTAAATTTATGCTATTGAGTGATGTTCTTACTGGGTATACCAAATACTTGTTGTTTAGTGTGGTGGATAACAATAATCACTTAATGATAAGAAATAAAATTAATTTATTTTGTATTTGATTAGAAGTATTAATTAGCAAATCTCAAAATTATCTATAGCCAATTATATTAAAATAATGAAATTTGTCATTAACTTATAAGAAATTCAAATTTAGGAGGTCGAGATAACTTTGTCTTTCCTATACTTATGGGTGGAGTATATATTTTTTATCGAAAAATTATGTAGTGTTTGAATATTACAATGTAATTATTATAAAATATTTAATTTTGCAAATTCTTAATATAATTAGGAAATAAAATTGTTCACTTTTCATCAAATTTCTAACTATGTTATTGCATATATGCTCACCCCACGATTCCTTAAAAATCACTTATTATGAAACGAGAGAATTAATTAGCCAGCTAGTATTGGGACTGCCGAAGTTGCAGTAACTTCCACGTTAGAGTATCTACACTATATATAATGGCATAGAATTAGTACATTTTCTCATCCCAAAACCAAAAAATAAATAAATTCTCTTAGTAGGATTCTATTTATTTTAAAGAATGCTCTTCATTAATTGAGCTTTCTCATTTTTTATTTGAAAGTCGTAACAACATTTTAATTCGCATAATTTATAGTTATTATTAGAGATTTTCTTTATATTCGATTTCTACTCATCGACTCGAAAATAATCGAAACATT

>SlXTH34

TATTTGCAAGTGTATATACAGTTTTCTCACTTTATACAAACAAAAACACAATTTATACATTTCGTTTCTGTTTGTATAAGTGAGAGAAGCGACGGTGGCGAGCAAGATTAGGGAGAATGGCGAGCGAGATCTAGAAAAGGGGAGAGAGGAGAATAAAAATATATGTATTTATACAATTTTCTTTCGTTTTATACAAACACAAATAAATTTTATATATTTGTGTTTGTATAAGGTTATATAATTTTTTAAAATTTTTTTGGATTTGAGGAGATATTTTAAGAAAGTTGAAAGAAATTGAAAGATAGCTAACAAATTTGTATAAAAATAGATAGTGGGCCACAATCAATTAAAAATAATTGATTTGGGAAGAAATTTTAGAGATAACCGAAAGAAAATGAGAAGAAAAGATAGGGAATGAATTTGGATAAAAATGAATCTTTCGCTTGATGAATGAGTTCGAACCAAATGACAAAAATTGTACAAAATTTGCTACGAAATACAAATGTTTTAAACAGTATCTATAGAATTTATTTTAATTTAAATATTTGCTATTATGTATGATTTTTCATAAATTAAAAATAGGCCACTTATTGCTAATTTAATCAATAAATAATCTTGATGTGTCATAATTTCATAAATGTTATTAAGCGCGCTAATTATTGTCCTTTTTAAAACATAAATAAGAGAAATTCAAAGTAGGGCGTTAGGCCCATTTCTGCCGAATCAAATGATAAAAAAATGAAATGTTTGCTGCGACTATATTGATTAAAGGAAGCTTCATGACTTTATTCTTAAAATACACATCTAGCCAAATTTAAGTCGGTTTTGATTTTAAGTCATAAATTTTATTGAGCCGTTTTTGATAATAGCTAACATATCCTTTACTTCTTATTAAAGATATTCTTGCTTAAACAAAACTCTTATTGGTTCTCCTCTTTCTTGTCTCACCAGCTTAATTCCCATACAATTCCACCTATATATATTTATATAATATATTTTA

>SlXTH35

TGTAATCTTTTGTTGAGACTTGAAGTTTAATAAAATGGAGCTAATTCTATGGAGGTGTAGATCGTGGTTATTACATTTAAAAGTTGGGGTTTTTCGCAATAAAATATTGTGTTATTATTTTATTTGCTTCACAAAATGTAACTGTTATAATATGCAAAGGAACATATAAGATAAACTTGTTCCTTAGGCAATTTTGAGGGTCAGAATTCCAACAAAATATGTCTCTGAAATTTGGGACATAGGTTAGGAAGATACTTATGCATTTTCCCTTAATTATATAATTATATTTGTGCAATCAAAAAATATATTTTTAAGTACACTATCATCAAATCATGCCAAATAAGCAAATCATTATAATTACTATACTATCTAATTACTAGGTTATACTCCCTATAGTTCGTATTAACTAAATTTTTGAGAGGATGTACCCTATTAAGAAATATATTTTAGGACATAAATTGAACTGTCACTTTATATTTTTGACCTTTTAGTTATTTTTAAATTATTTTAAAAAATAAAAATAATTTAAAGCAAAATTACAAATATACACAATTATCTCTCTTAAAATTATCAGCTATTTAAATATAATTTGACGGTTGGAAATATCCACCCTCCCTCTACTTGTTCATTTTTTAAAATAAATTTAAATTTTTTTACTTATTATATCTTCGACTATATATTTTTTTTTTTTAAAAATAAAATTTATTGAAAATTTTAATTGTTTTATTCATTCACTTATTGTAAATTAATTACATCAATAATTAACACAGTAGCCAAATAATTAGGAGTGAAAGTATAATATATGGTCTGTCTGTCACTTAGCTTTGATGCCGTCACTTTAATCCTGTTTCCAGCTCAAAATCTTTATTTTTTTCCTTGTTTTCTGTTTTACTCAACCTTCCTCTTTGCTTCCTCAAGCCTAACCCCACACGCTCTGCTCTACCTACTTCTTTATTGCTGAGGTTGGGGTGGTATAAACCCCTATAAAACCCCATATAAC

>SlXTH36

AGAAGCAAAGATACTGTATATGGTATTTTAGGGGTGTATACATTGCACGTTTCCCTATATTAACATTTTCTTATGCAAATGTTTTACTCCCCTAAATATCATTTTCCCACTTCCAAAAATTCAATAACTCCTCTCAATTAATATGATACAATTTTACTAAATATTATATTTAAAAATAAATATTTTGCAATTTATGATTTAAAATAAGTCATAAATATTTGTATGACTATAAAATATCTCATTAAAGTTAAATTAATGCTATATATCTCAGACTCTCAGTCAACTTTTCATGTTGTTCTTTTTTAAAGTCAACTTGACTCATTTTCAAAGTCAAATTAAATTACATTAATTTAATATTTTAAACAAAAAATACTTTTTTTCAAAGTTAAATTAAATTGCATTTTTTGTATATTAATATGATGAAAAATACATCATGTAGAATTTAGTAAAAGTTATTATAATTTGACTCTAAAAATCAATATGATAAACATACATTTTATAACGTTAGTCAAAATTATTATAATTTGAGTTTTAAAAAATAAAAAATAATGACAAAATAGACCAAAAAAAGTTATAAATGCAGAAATATGATAAAAAATAATTTGACTTTAAAAAAAGGGAAAAACAATAACAAAATAAAAAAAAAGTATTAAATGCAGAAATATGATAAAAAATACATCATACAACGTCAAAATTATTATAGTTTGACTGATGAAAAACACATTTTCTAATGATAGTCAAATTTTTTATTATTTGACTTTTAAAAAAGGAAAAACAATGACAAAACAGACCAAAAAAAAGTATTAAATGCAGAAATATGATTAAAAAAAATATAATTTGACTTTTAAAAAAGGAAAAACAATTACAAAATAGACCAAAAAAAAAGTATTAAATGCAGAAATATGACTAAAAATACATCATTCAATGTTTTCTTCCACCTAAATTTTCTATAAATTGAATTTGTGTCATTGTTCAACCAAACAAATTATCAAAACAAACA

>SlXTH37

TTGTAATCTTTTGTTGAGACTTGAAGTTTAATAAAGTGGAGTTAAATTCTACAGAGGTGTAGGTCGTGGTTTTTACCCTTAAAAGTTGAGGTTTTCCGCGATAAAATATTATGTTATTATTTTATTTGTTTCACAAAACGTAACTGCTGTAATCTACAAATGAACATATGAAATAAACATGTTCCTTAGACAATTTTAGGGGTAAGAATTCCAACAAAGTGTGTCTCTAAAAAATTTCAAACATAGATAGGAGAGTACTTGTGTATTTTTCCTTAATTATATAATTATATTTGTGCAATCAAAAAAATATTTTTAAGTACACTATCAATAAATCATGCCAAATAAGCAAATCATTATAATTACTATACTATCTAATTACTAGGTTATACTCCTTATAGTTCGTATTAACTAAATTTTCGAGAGGATGCACCCCATTAAGAAATATAATTTAGGACATAAATTGAACTATCACTTTATATTTTTGACCTTTTAGTTATTTTTAAATTATCTTAAAAAATAAAAATAATTTAAAGCAAAATTACAAATATAAGCAATACTCTCTCTTAAACTTATCAGCTATGTAAATATAATTTGACGGTTGGAAATATCCACCCTCCCTCTACTTGTTCATTTTTTAAAATAAATTTAAAAAGTTTACTTATTATATTTTTAATTAAATATTTTTTAAAAAATAAACTTTATTGAAAATTTTAATTGTTTTGATCATTCACTAATAGTAAATTTATTATATCAATAATTAACACATTTAAAAGTAGCCAAATAATTAGGAGTGAGAGTATAATATATGGTCTGTCTGTCACTTAGCTTTGATGCCGTCACTTTAATCCTGTTTCCAGCTCAAAATCTTTATTTTTTCCCTTGTTTTCTGTTTTACTTAACCTTCCTCTTTGCTTCCTCAAGCCTAACCCCACACGCTCTGCTCTACCTACTTCTTTATTGCTGAGGTTGGGGTGGTATAAATCCCTATAAAACCCCATAT
