# Supplementary material for: Genome-wide characterization of the xyloglucan endotransglucosylase/hydrolase gene family in Solanum lycopersicum L. and gene expression analysis in response to arbuscular mycorrhizal symbiosis
Source: PeerJ. 2023 May 3;11:e15257. doi: 10.7717/peerj.15257 (PMC10163873; doi:10.7717/peerj.15257)
Supplement: Supplemental Information 9 [file peerj-11-15257-s009.docx]

| Gene | Forward (5´---3’) | Reverse (5´---3’) | PCR product size |
| --- | --- | --- | --- |
| *XTH2* | GGGTTAACAAGGATGAAATGGG | CACACAACAACAACTCTTACCG | 189 |
| *XTH3* | CAAAGCGATTTCCACAGGG | TTGCATCTTCTACTGATGGG | 137 |
| *XTH6* | GGGATGCATCATCTTGGGC | AAGAGGGACTCTCGTAGGC | 126 |
| *XTH7* | AGACAAATCGAGAAACCCCG | GCACAAATTTCATCCCCCC | 107 |
| *XTH9* | CTTAGGCAATGTCTCTGGCC | ATGGTGTAAGTGTGAAACGCG | 126 |
| *XTH14* | CTTACACCAGAATGTTGGCG | GTGTACCATCGATACATCCC | 113 |
| *XTH17* | ACATTAACATTGATGGCTGCG | AACCTCTTAGAATCCGCGC | 184 |
| *XTH21* | GAGCAGACCAAAGTTGAAGGG | TAGAACATGGCTTGGGACCC | 200 |
| *XTH35* | ACATTACATGGGGTGATGGC | ATAGTAAGCAGTGACAGTGCC | 179 |
| *EF-α* | GGCGAGCATGATTTTGAGTC | CAAAAATCCGAGCCACCAT | 180 |

**Table S1.** Primer pairs used in this study
