# Supplementary material for: Genome-wide characterization of the xyloglucan endotransglucosylase/hydrolase gene family in Solanum lycopersicum L. and gene expression analysis in response to arbuscular mycorrhizal symbiosis
Source: PeerJ. 2023 May 3;11:e15257. doi: 10.7717/peerj.15257 (PMC10163873; doi:10.7717/peerj.15257)
Supplement: Supplemental Information 10 [file peerj-11-15257-s010.docx]

**Table S2.** Chromosomal coordinates, signal peptide and subcellular location of xyloglucan endotransglucosylase/hydrolase (*XTH*) genes family in tomato (*Solanum lycopersicum* L.).

| Name | SGN  I.D. |  |  |  |
| --- | --- | --- | --- | --- |
|  |  | Chromosome Coordinates | Signal peptide sequence | Subcellular location |
| *SlXTH1* | Solyc01g099630.2.1 | SL2.50ch01:89810092..89812819 | VFCGY | Plasma membrane |
| *SlXTH2* | Solyc07g009380.2.1 | SL2.50ch07:4435429..4437750 | AFGGT | Plasma membrane |
| *SlXTH3* | Solyc03g093130.2.1 | SL2.50ch03:54305583..54306831 | AIGAK | Plasma membrane |
| *SlXTH4* | Solyc11g065600.1.1 | SL2.50ch11:51118385..51120727 | ASCGA | Plasma membrane |
| *SlXTH5* | Solyc01g081060.2.1 | SL2.50ch01:80388141..80391373 | CRAFN | Extracellular |
| *SlXTH6* | Solyc11g066270.1.1 | SL2.50ch11:52001274..52003378 | INAQG | Extracellular |
| *SlXTH7* | Solyc02g091920.2.1 | SL2.50ch02:53134100..53135731 | VSARP | Plasma membrane |
| *SlXTH8* | Solyc04g008210.1.1 | SL2.50ch04:1894160..1896580 | GFSEN | Plasma membrane |
| *SlXTH9* | Solyc12g011030.1.1 | SL2.50ch12:3892612..3893717 | ASAGN | Plasma membrane |
| *SlXTH10* | Solyc07g056000.2.1 | SL2.50ch07:63901549..63902824 | VSADN | Plasma membrane |
| *SLXTH11* | Solyc12g017240.1.1 | SL2.50ch12:6426369..6427448 | VWADN | Plasma membrane |
| *SlXTH12* | Solyc09g092520.2.1 | SL2.50ch09:71577901..71579975 | TYGGN | Plasma membrane |
| *SlXTH13* | Solyc07g006850.1.1 | SL2.50ch07:1712267..1716538 | ----------- | Plasma membrane |
| *SlXTH14* | Solyc09g008320.2.1 | SL2.50ch09:1783996..1786766 | TNANY | Extracellular |
| *SlXTH15* | Solyc03g031800.2.1 | SL2.50ch03:4282559..4284737 | TQAEV | Plasma membrane |
| *SlXTH16* | Solyc07g052980.2.1 | SL2.50ch07:61401256..61403204 | VSSAK | Plasma membrane |
| *SlXTH17* | Solyc07g055990.2.1 | SL2.50ch07:63891570..63893537 | VLAAG | Plasma membrane |
| *SlXTH18* | Solyc12g007260.1.1 | SL2.50ch12:1680869..1682934 | ---------- | Plasma membrane |
| *SlXTH19* | Solyc05g046290.2.1 | SL2.50ch05:58485924..58488638 | ALAGN | Plasma membrane |
| *SlXTH20* | Solyc07g006870.2.1 | SL2.50ch07:1722228..1724125 | CASND | Plasma membrane |
| *SlXTH21* | Solyc01g005120.2.1 | SL2.50ch01:104191..107414 | VSGFS | Plasma membrane |
| *SlXTH22* | Solyc12g007270.1.1 | SL2.50ch12:1685022..1687362 | ---------- | Plasma membrane |
| *SlXTH23* | Solyc02g080160.2.1 | SL2.50ch02:44460578..44462486 | SNGVV | Plasma membrane |
| *SlXTH24* | Solyc03g093120.2.1 | SL2.50ch03:54289827..54291069 | AIGAK | Plasma membrane |
| *SlXTH25* | Solyc05g005680.2.1 | SL2.50ch05:506438..508385 | VSCRG | Plasma membrane |
| *SlXTH26* | Solyc08g076080.2.1 | SL2.50ch08:60127904..60132098 | AETAA | Extracellular |
| *SlXTH27* | Solyc10g005350.2.1 | SL2.50ch10:258489..260485 | IKCSF | Plasma membrane |
| *SlXTH28* | Solyc03g098430.2.1 | SL2.50ch03:60736089..60737565 | GVGVN | Plasma membrane |
| *SlXTH29* | Solyc12g007250.1.1 | SL2.50ch12:1676397..1677809 | ANGWT | Plasma membrane |
| *SlXTH30* | Solyc05g053700.1.1 | SL2.50ch05:63744036..63746991 | GLALP | Plasma membrane |
| *SlXTH31* | Solyc06g083400.1.1 | SL2.50ch06:48806186..48808342 | IYAMV | Plasma membrane |
| *SlXTH32* | Solyc07g006860.2.1 | SL2.50ch07:1717942..1719773 | SLASN | Plasma membrane |
| *SlXTH33* | Solyc11g040140.1.1 | SL2.50ch11:39807936..39810801 | SMGAK | Plasma membrane |
| *SlXTH34* | Solyc01g106650.2.1 | SL2.50ch01:94434107..94435528 | ALASI | Plasma membrane |
| *SlXTH35* | Solyc03g093080.2.1 | SL2.50ch03:54267479..54268694 | AIGAK | Plasma membrane |
| *SlXTH36* | Solyc11g017450.1.1 | SL2.50ch11:8400274..8401408 | CLSAN | Extracellular |
| *SlXTH37* | Solyc03g093110.2.1 | SL2.50ch03:54284652..54285913 | AIGAK | Plasma membrane |
