# Supplementary material for: Genome-wide characterization of the xyloglucan endotransglucosylase/hydrolase gene family in Solanum lycopersicum L. and gene expression analysis in response to arbuscular mycorrhizal symbiosis
Source: PeerJ. 2023 May 3;11:e15257. doi: 10.7717/peerj.15257 (PMC10163873; doi:10.7717/peerj.15257)
Supplement: Supplemental Information 11 [file peerj-11-15257-s011.docx]

| **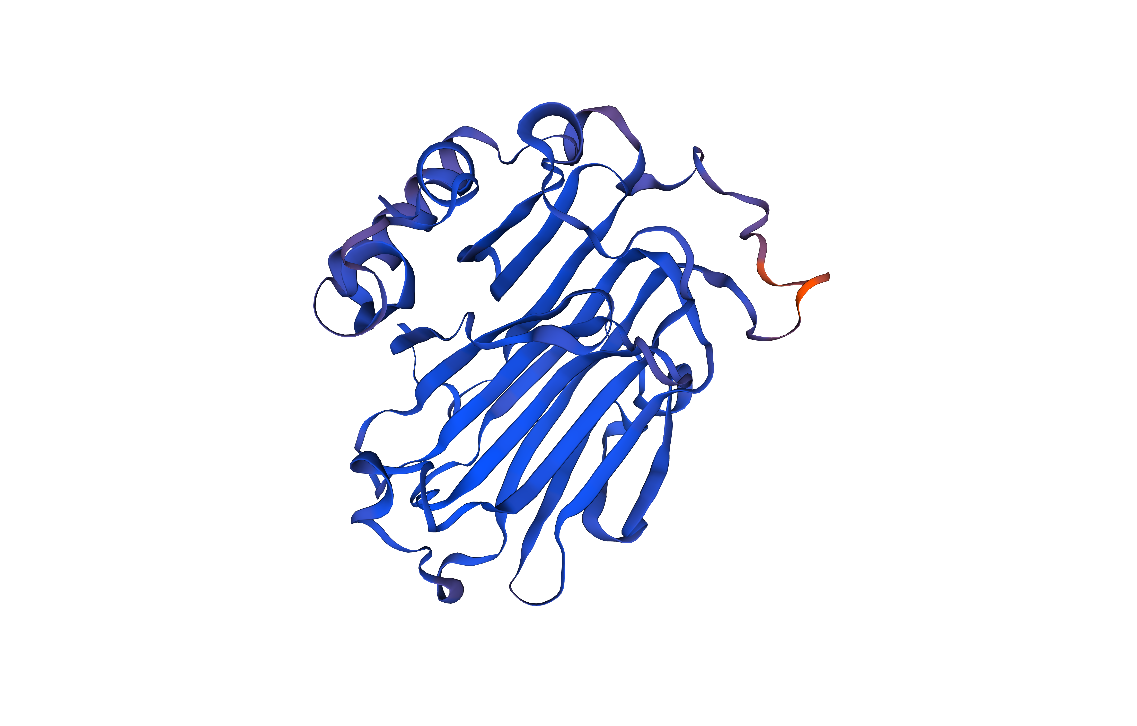**  **SlXTH1** | **Template** | **1umz.1.A** |
| --- | --- | --- |
|  | **Seq Identity (%)** | **76.01** |
|  | **GMQE** | **0.87** |
|  | **QMEANDisCo** | **0.90±0.05** |
|  | **Ligand** | **1.- BGC-BGC-BGC-XYS-XYS-GAL**  **2.- NAG-NAG-BMA** |
| 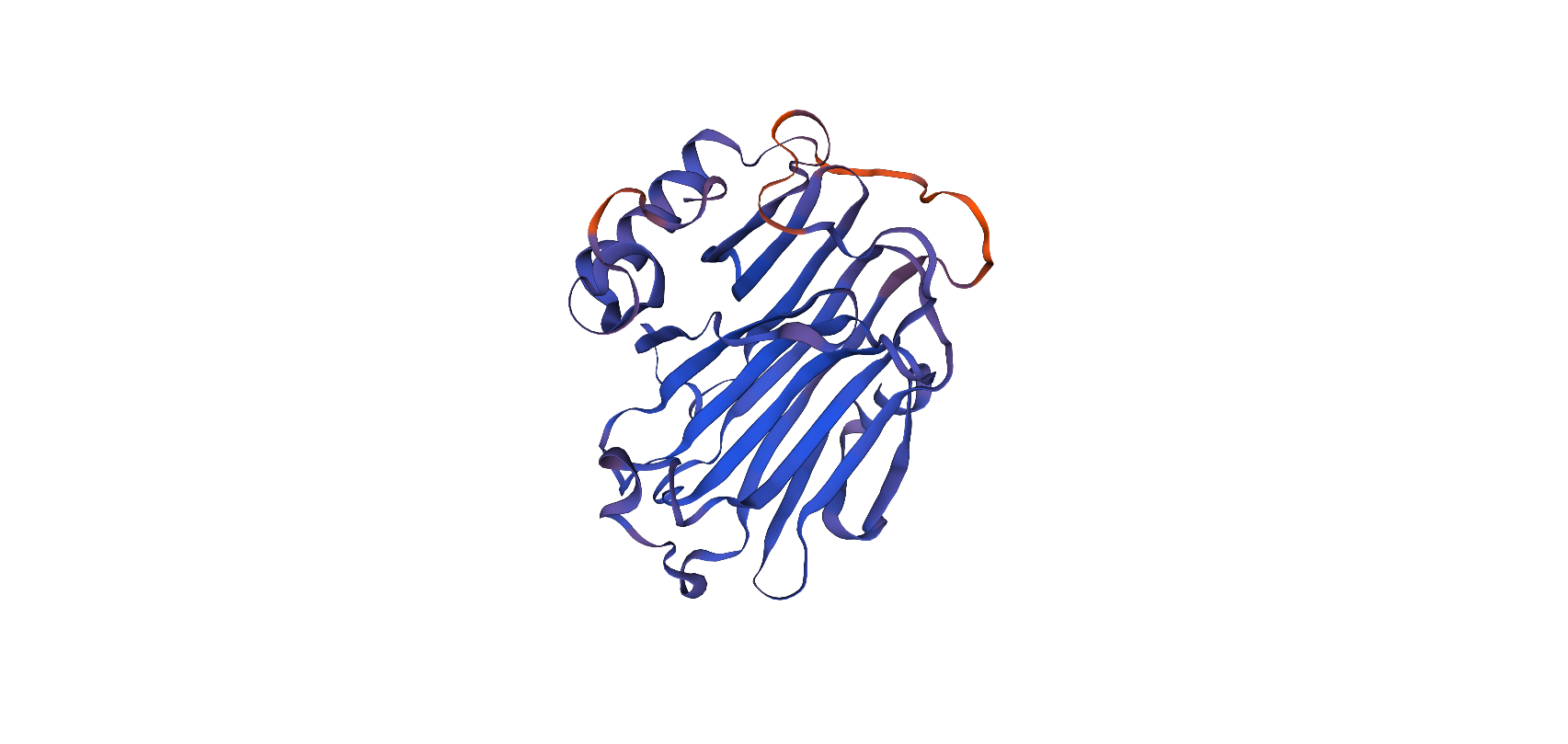  **SlXTH2** | **Template** | **1un1.2.A** |
|  | **Seq Identity (%)** | **54.29** |
|  | **GMQE** | **0.80** |
|  | **QMEANDisCo** | **0.82± 0.05** |
|  | **Ligand** | **1.- BGC-BGC-BGC-XYS-XYS-GAL**  **2.- NAG-NAG-BMA** |

**Table S3.** Predicted 3D structure of the SlXTH proteins

| **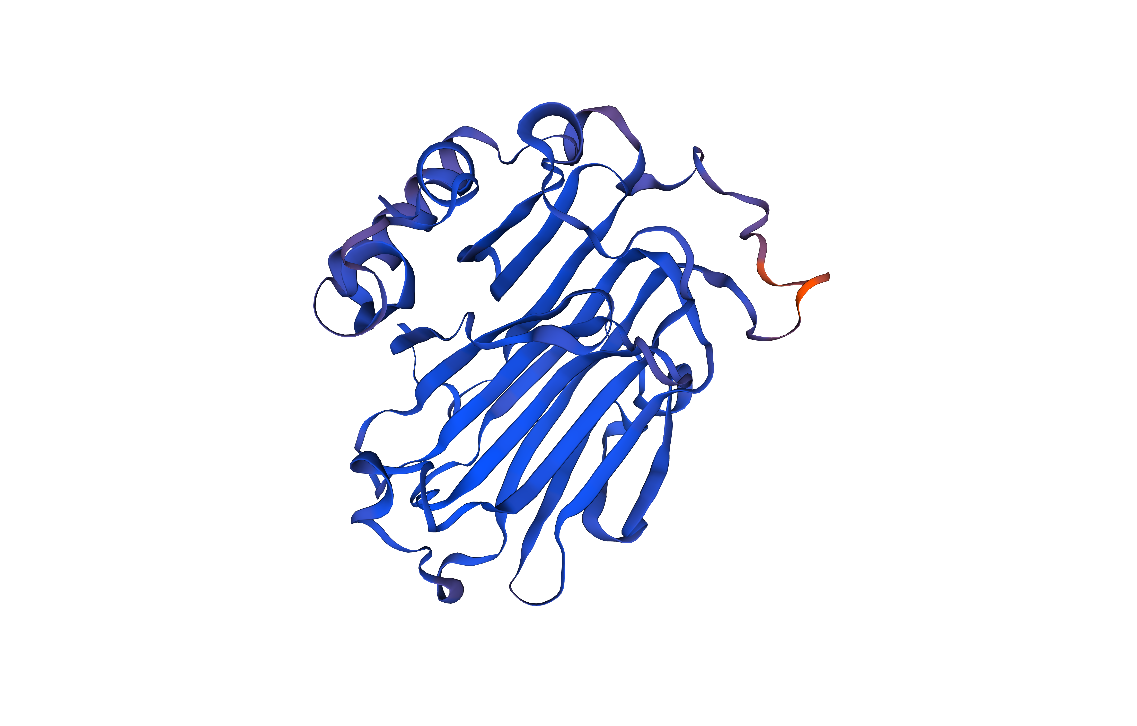**  **SlXTH1** | **Template** | **1umz.1.A** |
| --- | --- | --- |
|  | **Seq Identity (%)** | **76.01** |
|  | **GMQE** | **0.87** |
|  | **QMEANDisCo** | **0.90±0.05** |
|  | **Ligand** | **1.- BGC-BGC-BGC-XYS-XYS-GAL**  **2.- NAG-NAG-BMA** |
| 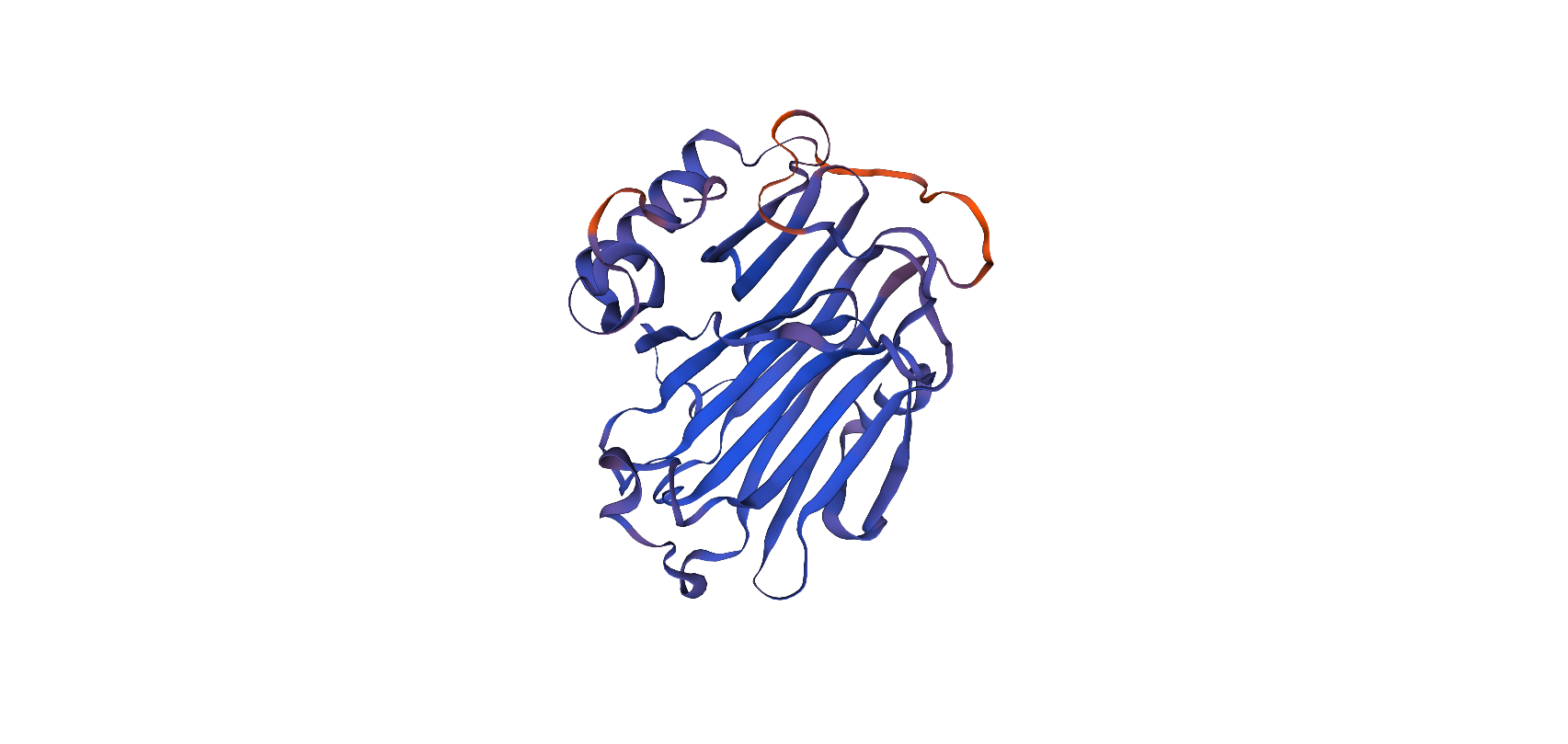  **SlXTH2** | **Template** | **1un1.2.A** |
|  | **Seq Identity (%)** | **54.29** |
|  | **GMQE** | **0.80** |
|  | **QMEANDisCo** | **0.82± 0.05** |
|  | **Ligand** | **1.- BGC-BGC-BGC-XYS-XYS-GAL**  **2.- NAG-NAG-BMA** |
| **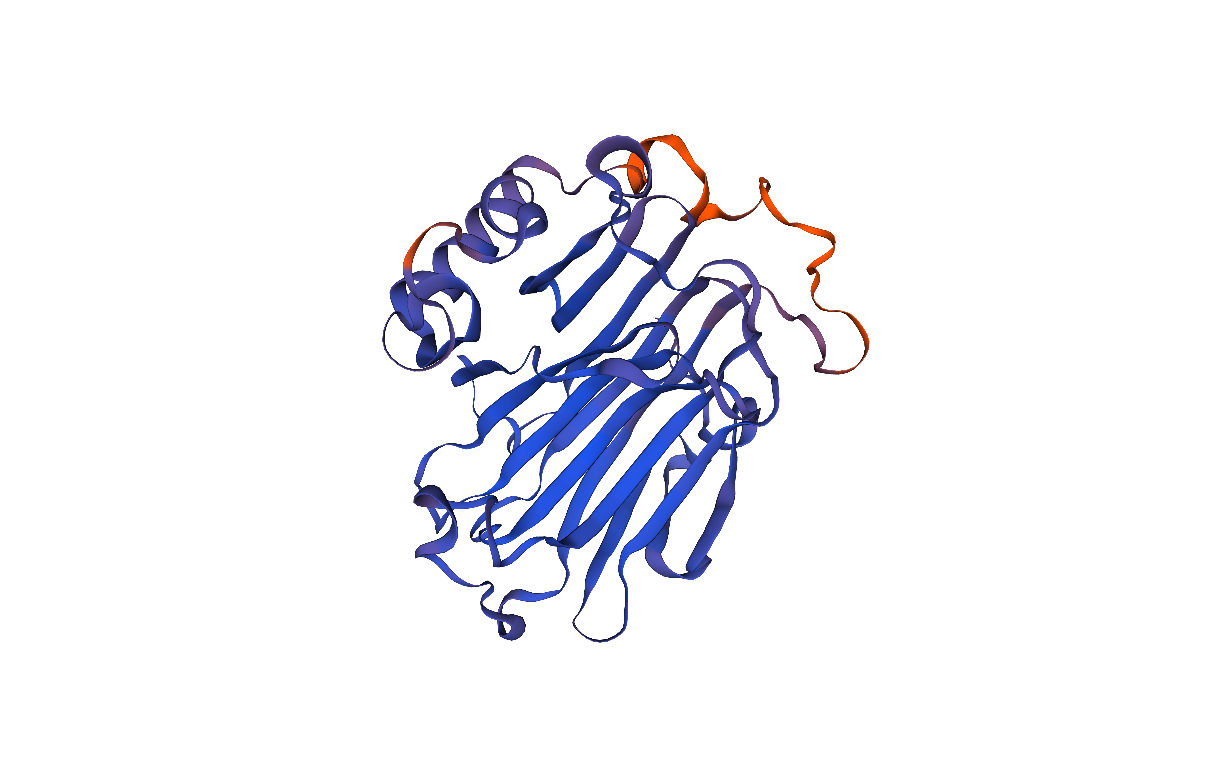**  **SlXTH3** | **Template** | **1umz.1.A** |
|  | **Seq Identity (%)** | **52.22** |
|  | **GMQE** | **0.82** |
|  | **QMEANDisCo** | **0.82 ± 0.05** |
|  | **Ligand** | **1.- BGC-BGC-BGC-XYS-XYS-GAL**  **2.- NAG-NAG-BMA** |
| 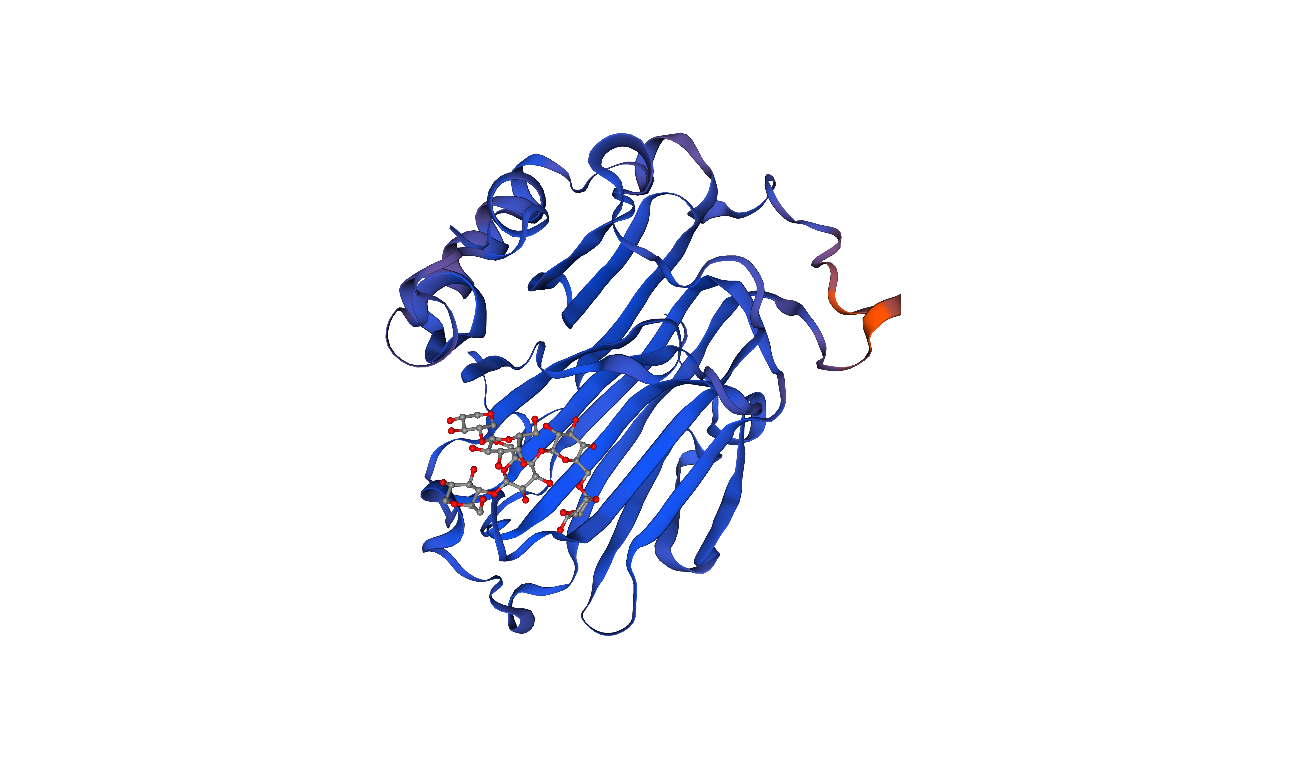  **SlXTH4** | **Template** | **1umz.1.A** |
|  | **Seq Identity (%)** | **77.32** |
|  | **GMQE** | **0.87** |
|  | **QMEANDisCo** | **0.89 ± 0.05** |
|  | **Ligand** | **1.- BGC-BGC-BGC-XYS-XYS-GAL**  **2.- NAG-NAG-BMA** |
| **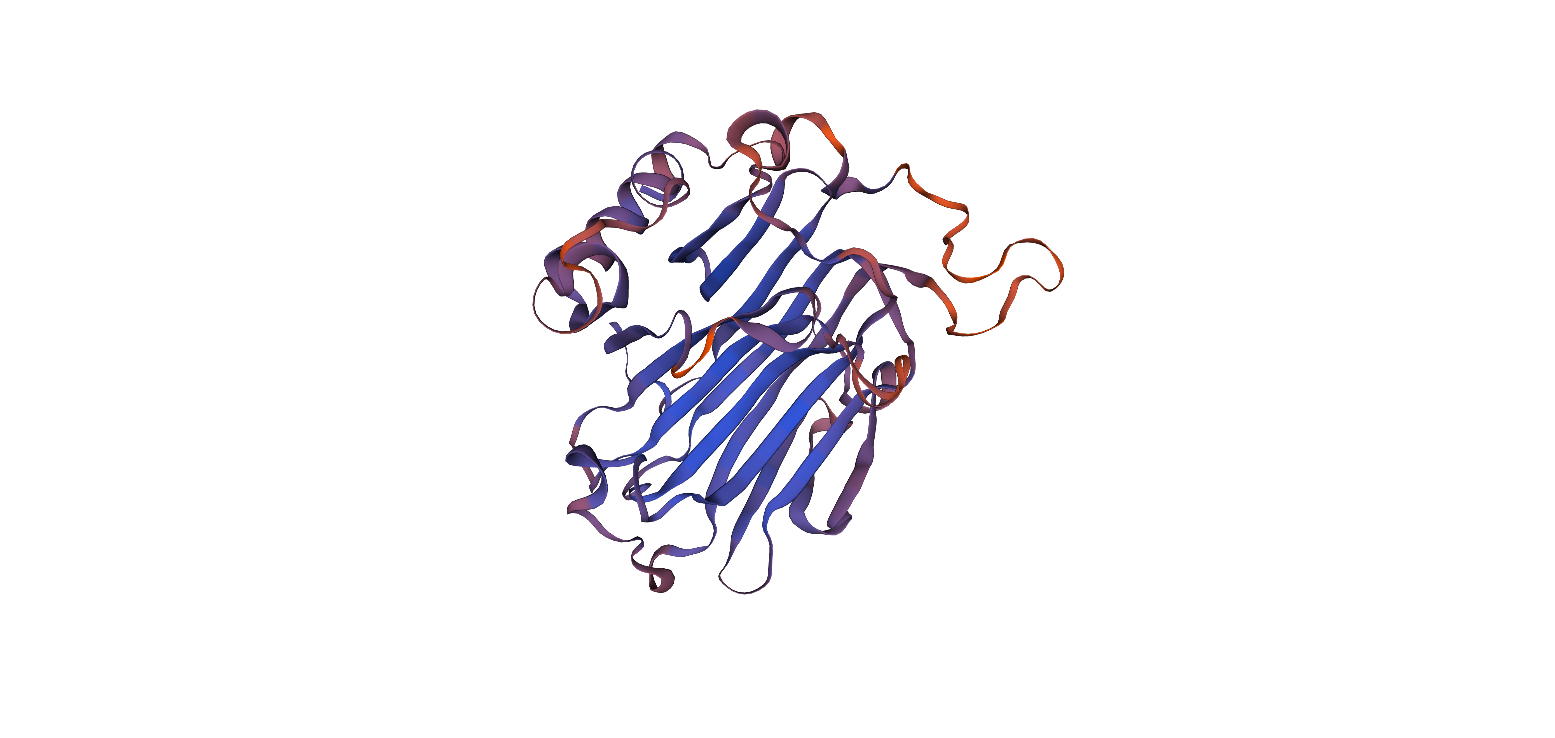**  **SlXTH5** | **Template** | **1umz.1.A** |
|  | **Seq Identity (%)** | **39.33** |
|  | **GMQE** | **0.64** |
|  | **QMEANDisCo** | **0.73 ± 0.05** |
|  | **Ligand** | **1.- BGC-BGC-BGC-XYS-XYS-GAL**  **2.- NAG-NAG-BMA** |
| 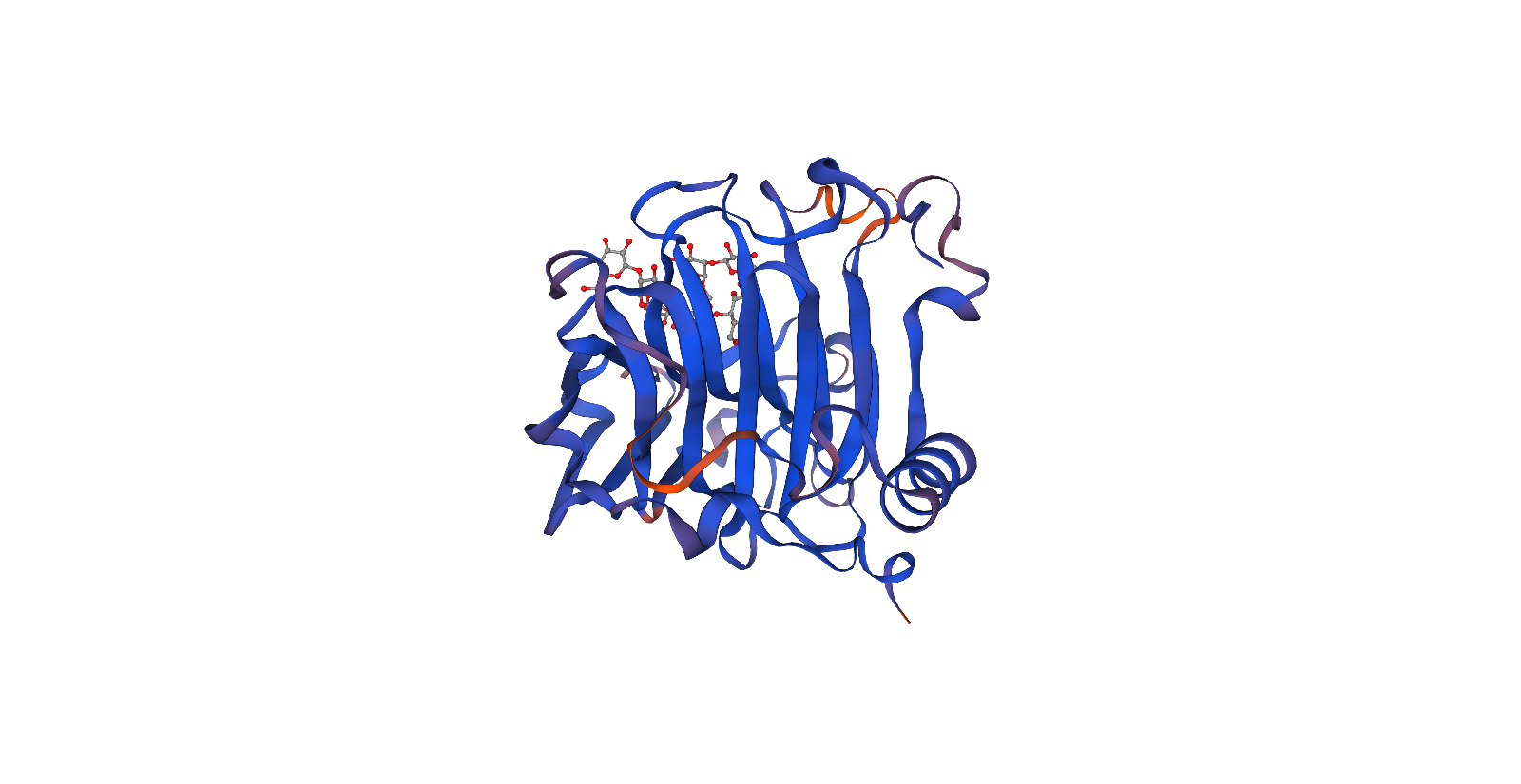  **SlXTH6** | **Template** | **2vh9.1.A** |
|  | **Seq Identity (%)** | **73.03** |
|  | **GMQE** | **0.84** |
|  | **QMEANDisCo** | **0.86 ± 0.05** |
|  | **Ligand** | **1.- BGC-BGC-XYS-GAL**  **2.- BGC-XYS-GAL**  **3.- BGC-XYS-GOL**  **4.- Zn** |
| **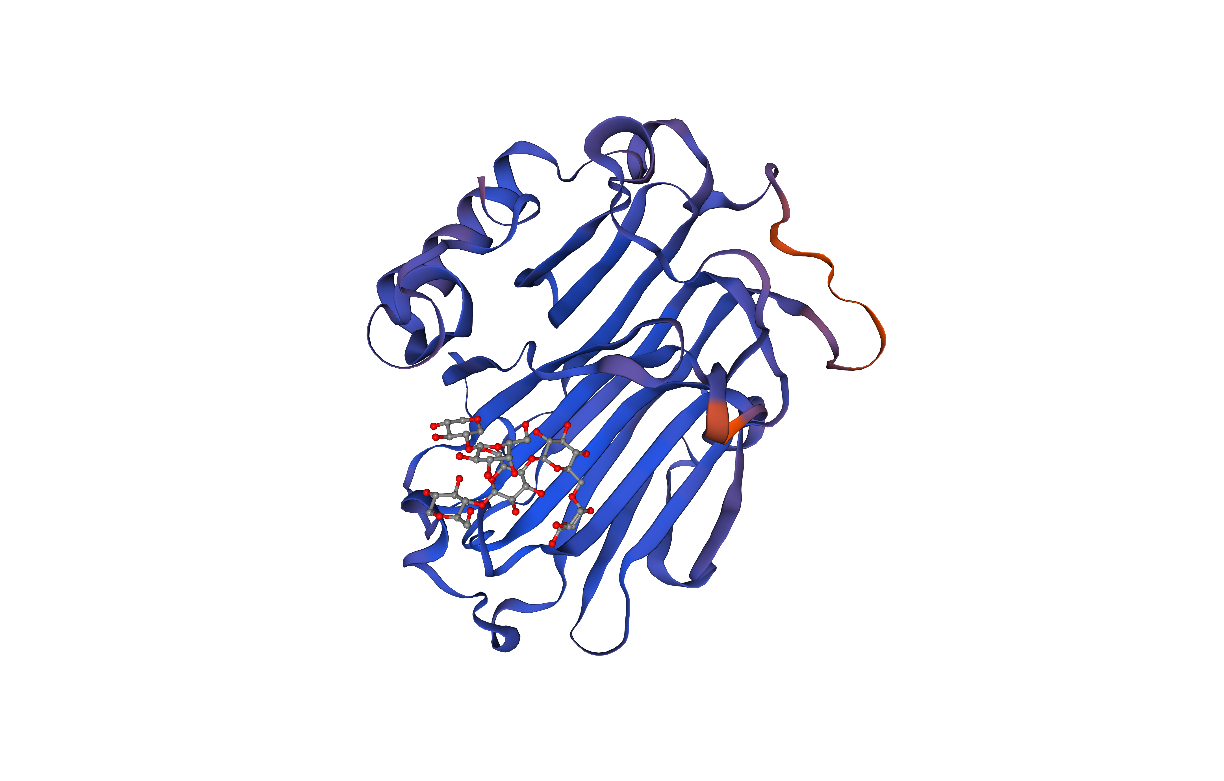**  **SlXTH7** | **Template** | **1umz.1.A** |
|  | **Seq Identity (%)** | **55.04** |
|  | **GMQE** | **0.79** |
|  | **QMEANDisCo** | **0.84 ± 0.05** |
|  | **Ligand** | **1.- BGC-BGC-BGC-XYS-XYS-GAL**  **2.- NAG-NAG-BMA** |
| 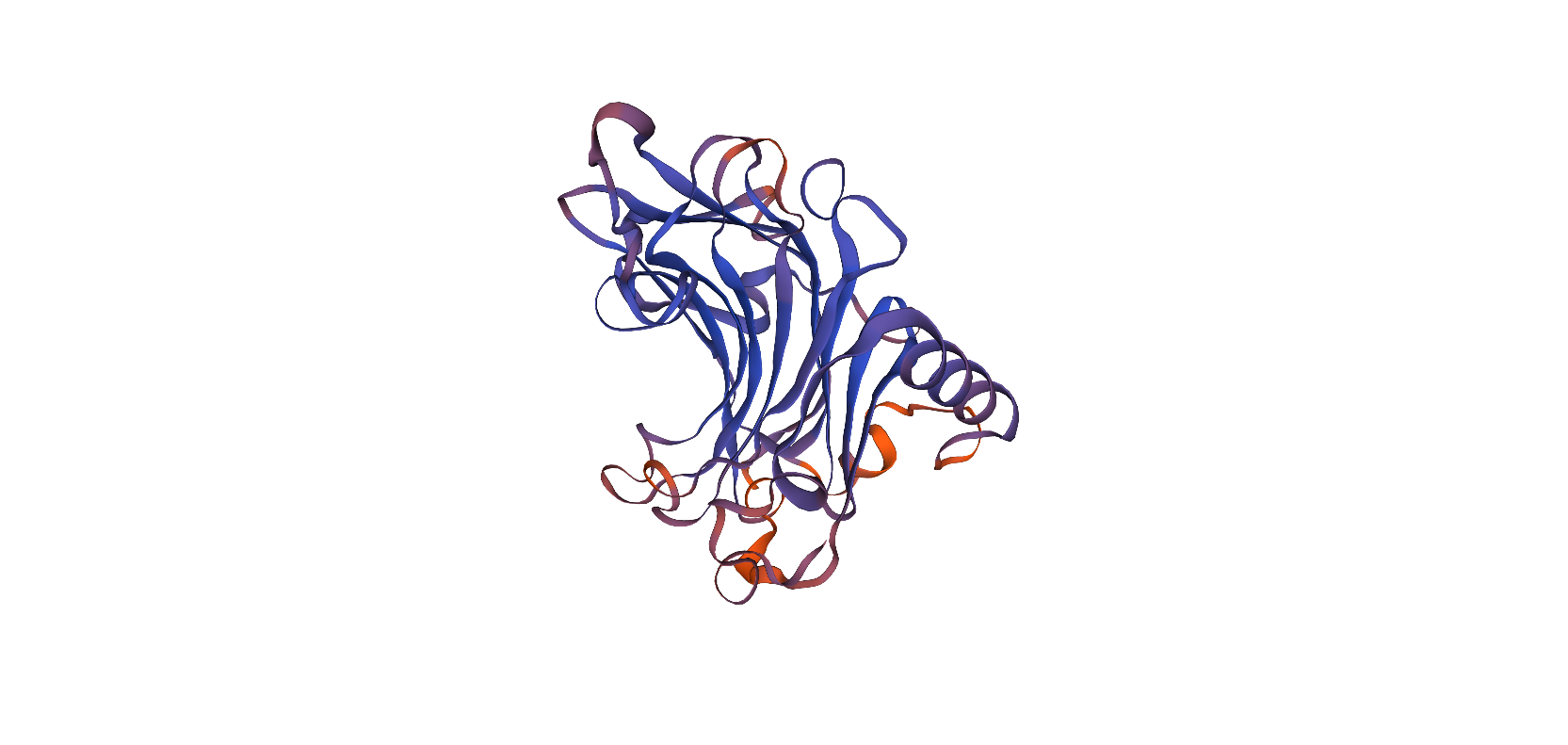  **SlXTH8** | **Template** | **2uwa.1.A** |
|  | **Seq Identity (%)** | **45.74** |
|  | **GMQE** | **0.65** |
|  | **QMEANDisCo** | **0.74 ± 0.05** |
|  | **Ligand** | **None** |
| **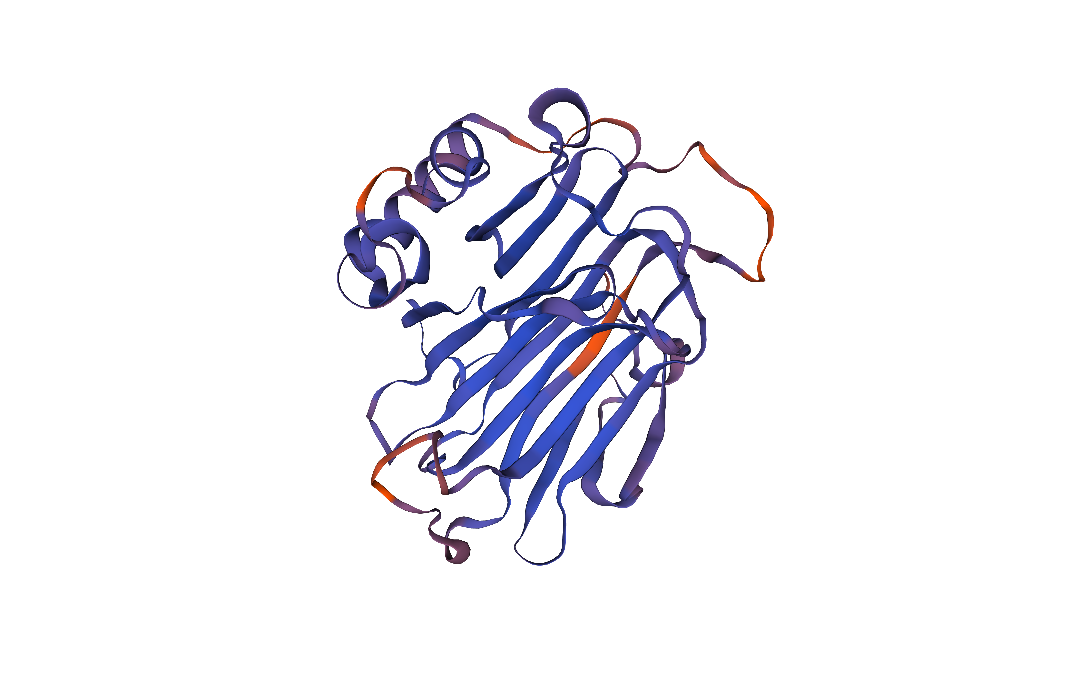**  **SlXTH9** | **Template** | **1umz.1.A** |
|  | **Seq Identity (%)** | **46.12** |
|  | **GMQE** | **0.78** |
|  | **QMEANDisCo** | **0.78 ± 0.05** |
|  | **Ligand** | **1.- BGC-BGC-BGC-XYS-XYS-GAL**  **2.- NAG-NAG-BMA** |
| 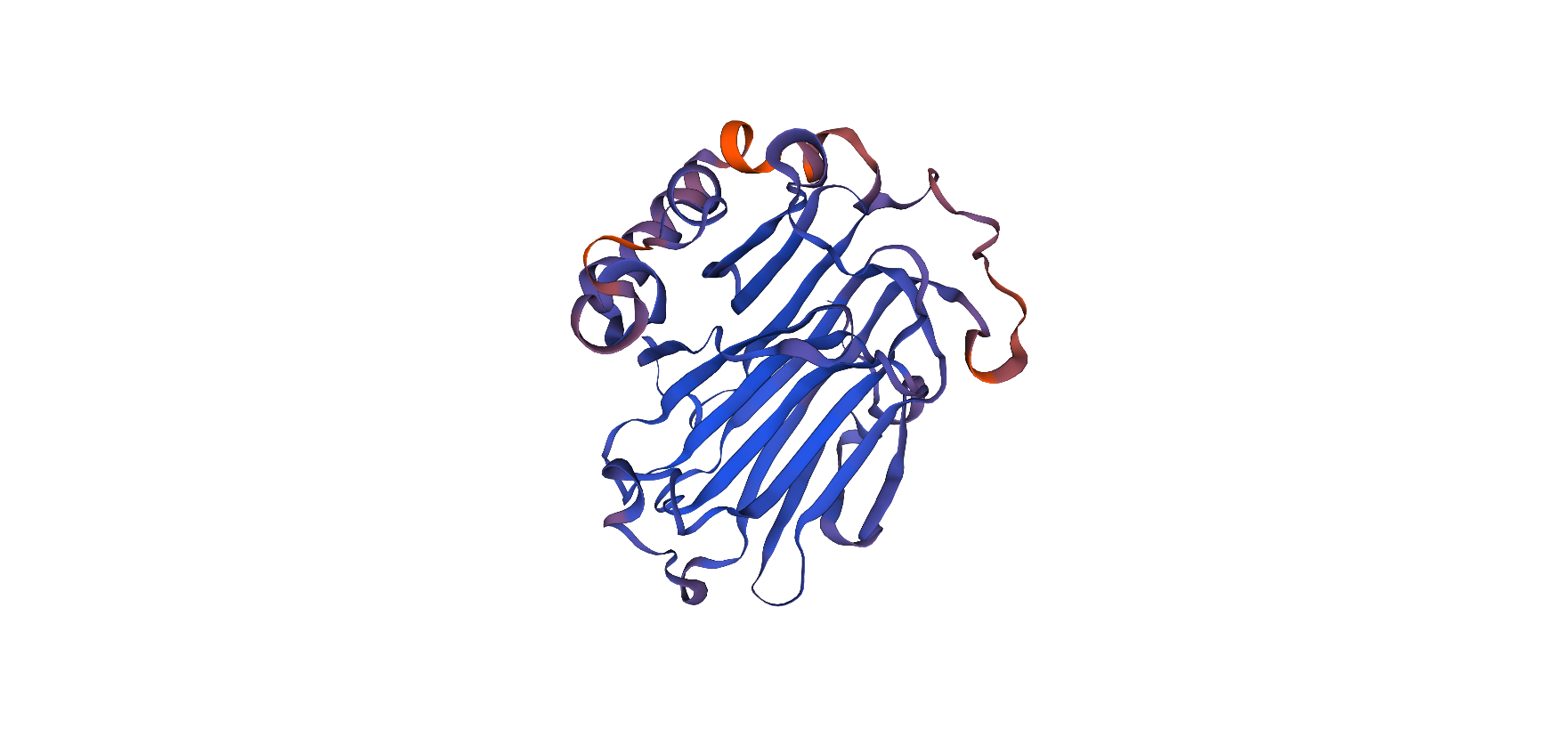  **SlXTH10** | **Template** | **1umz.1.A** |
|  | **Seq Identity (%)** | **52.57** |
|  | **GMQE** | **0.82** |
|  | **QMEANDisCo** | **0.81 ± 0.05** |
|  | **Ligand** | **1.- BGC-BGC-BGC-XYS-XYS-GAL**  **2.- NAG-NAG-BMA** |
| **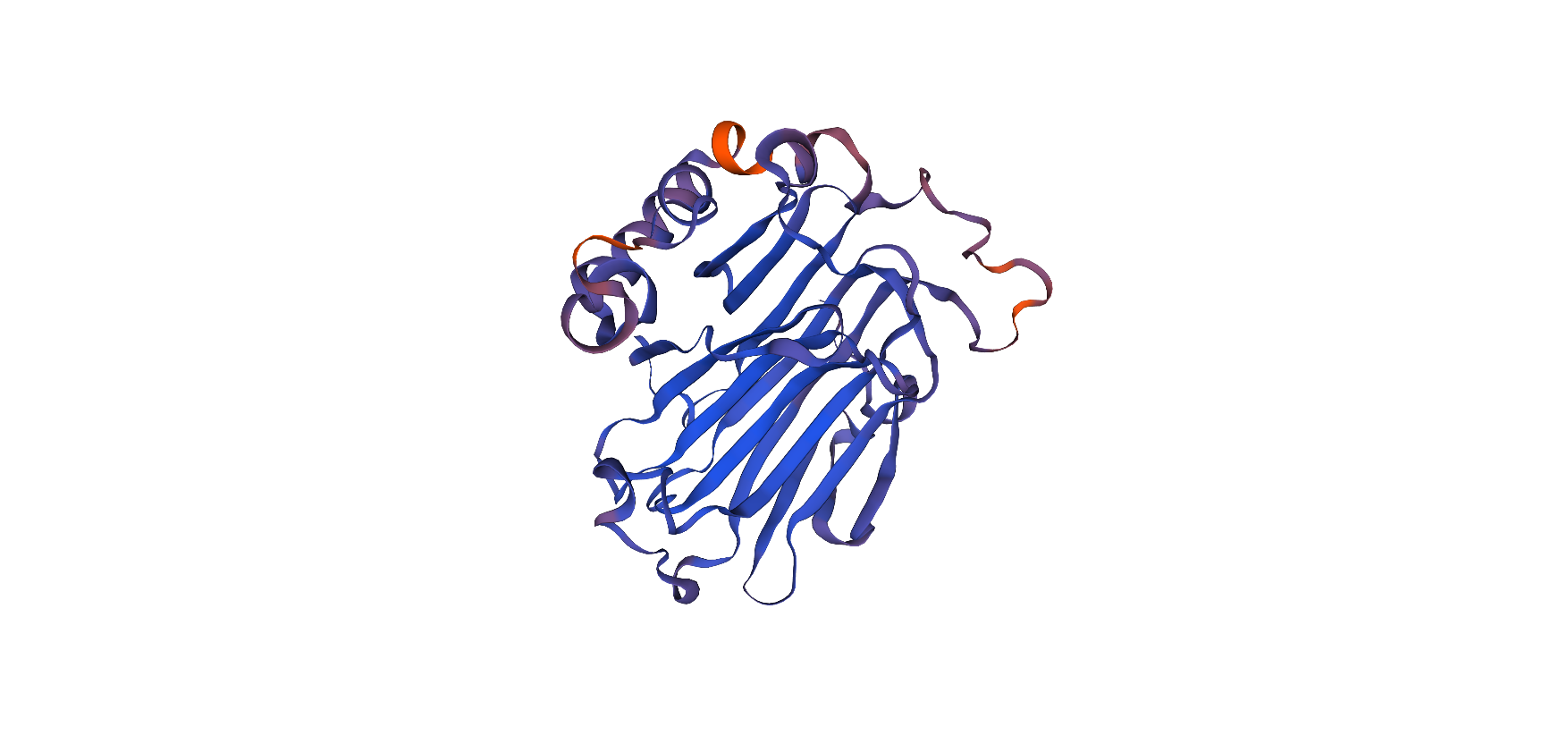**  **SlXTH11** | **Template** | **1umz.1.A** |
|  | **Seq Identity (%)** | **53.53** |
|  | **GMQE** | **0.82** |
|  | **QMEANDisCo** | **0.82 ± 0.05** |
|  | **Ligand** | **1.- BGC-BGC-BGC-XYS-XYS-GAL**  **2.- NAG-NAG-BMA** |
| 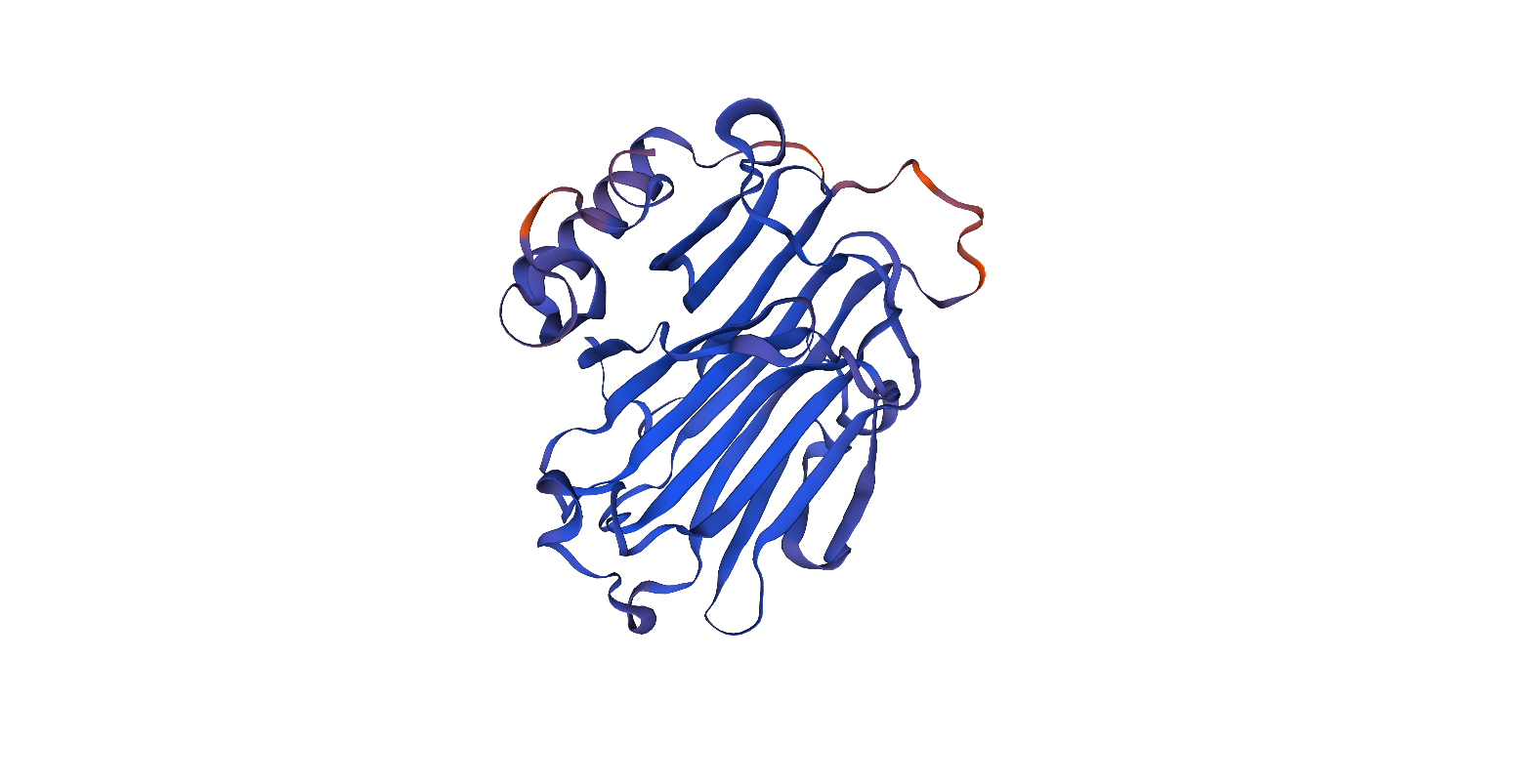  **SlXTH12** | **Template** | **1umz.1.A** |
|  | **Seq Identity (%)** | **56.42** |
|  | **GMQE** | **0.85** |
|  | **QMEANDisCo** | **0.86 ± 0.05** |
|  | **Ligand** | **1.- BGC-BGC-BGC-XYS-XYS-GAL**  **2.- NAG-NAG-BMA** |
| **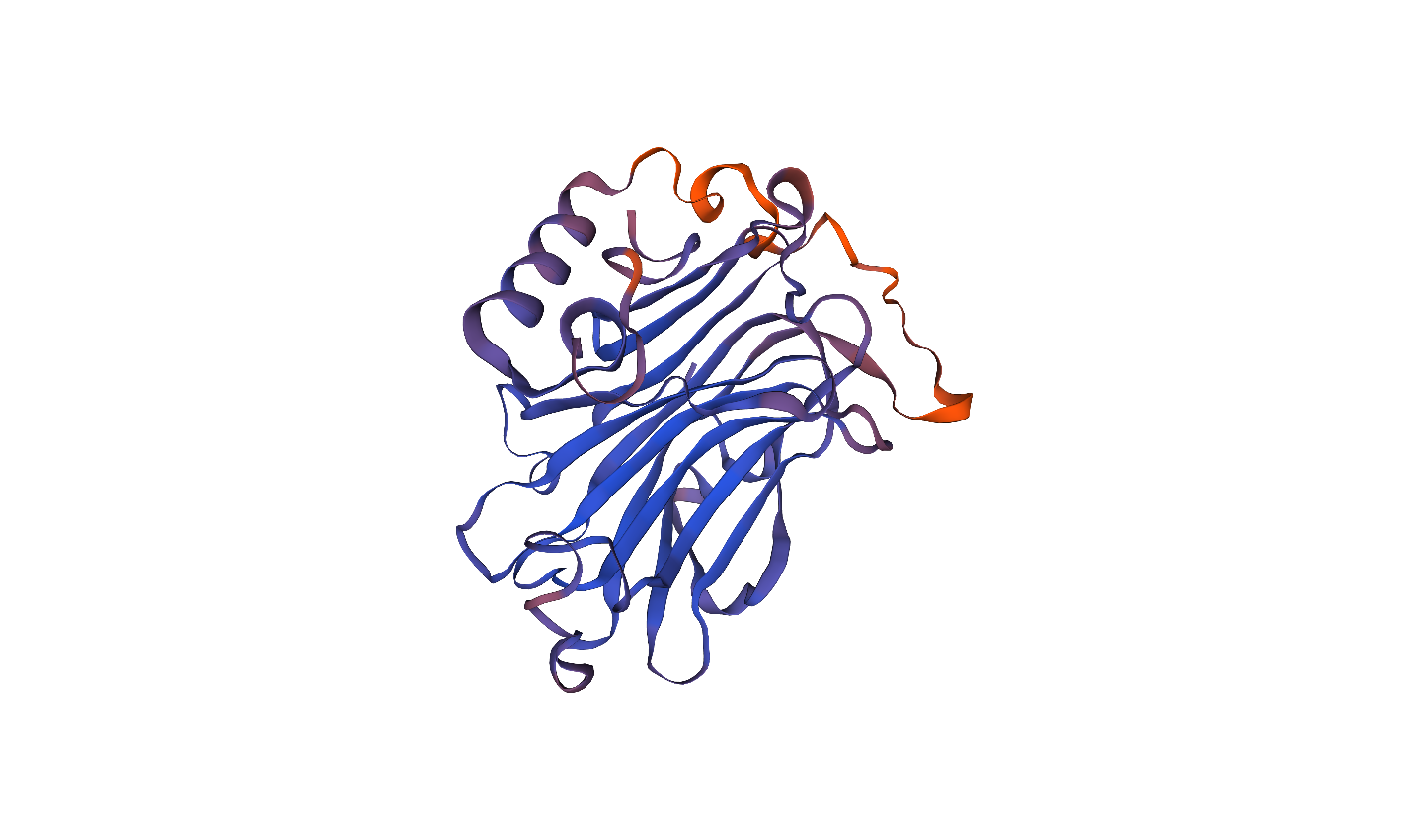**  **SlXTH13** | **Template** | **1umz.1.A** |
|  | **Seq Identity (%)** | **51.34** |
|  | **GMQE** | **0.65** |
|  | **QMEANDisCo** | **0.78 ± 0.05** |
|  | **Ligand** | **1.- BGC-BGC-BGC-XYS-XYS-GAL**  **2.- NAG-NAG-BMA** |
| 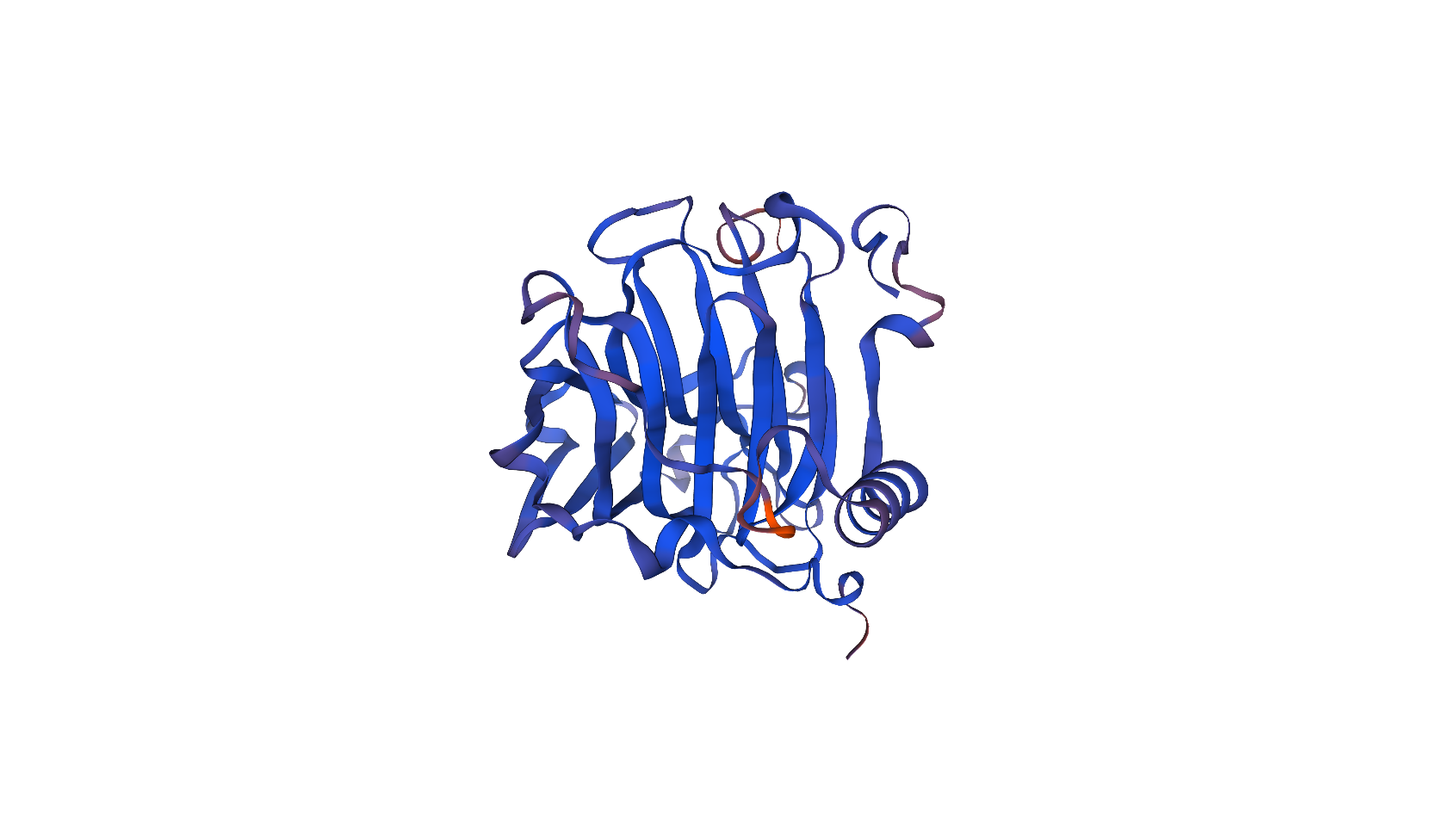  **SlXTH14** | **Template** | **2uma.1.A** |
|  | **Seq Identity (%)** | **68.01** |
|  | **GMQE** | **0.86** |
|  | **QMEANDisCo** | **0.87 ± 0.05** |
|  | **Ligand** | **None** |
| **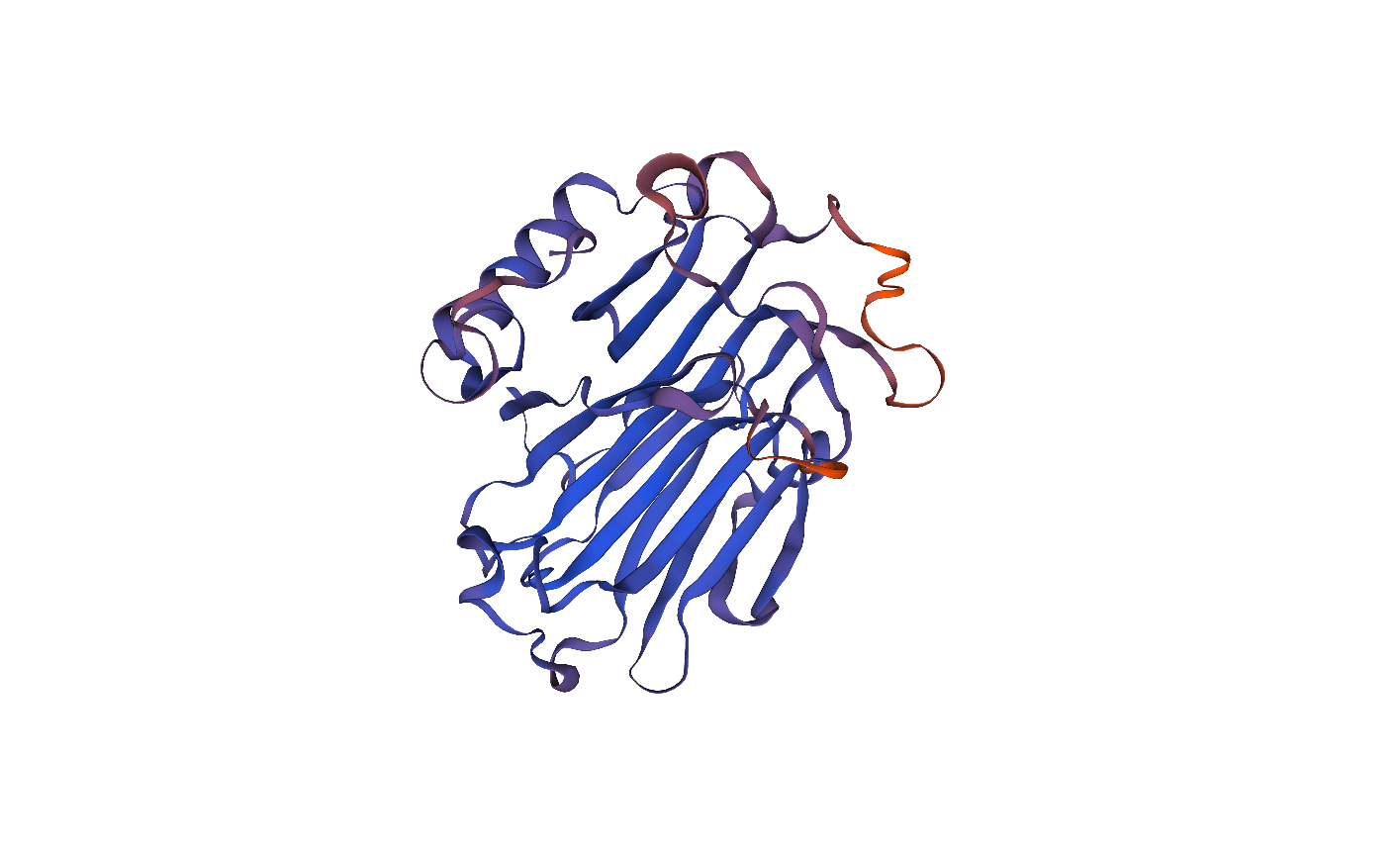**  **SlXTH15** | **Template** | **1umz.1.A** |
|  | **Seq Identity (%)** | **49.44** |
|  | **GMQE** | **0.78** |
|  | **QMEANDisCo** | **0.80 ± 0.05** |
|  | **Ligand** | **1.- BGC-BGC-BGC-XYS-XYS-GAL**  **2.- NAG-NAG-BMA** |
| 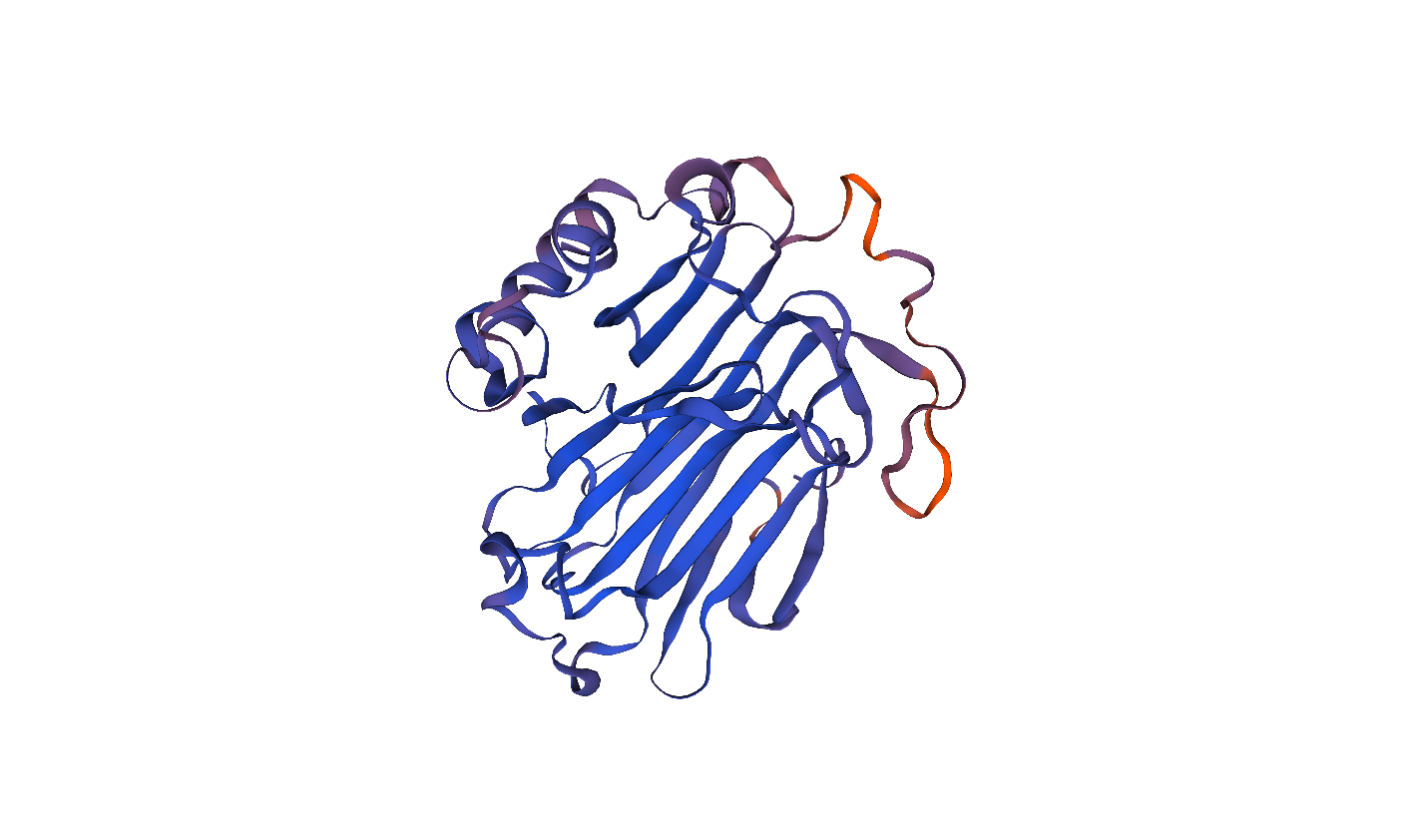  **SlXTH16** | **Template** | **2uwa.1.A** |
|  | **Seq Identity (%)** | **53.88** |
|  | **GMQE** | **0.79** |
|  | **QMEANDisCo** | **0.82 ± 0.05** |
|  | **Ligand** | **1.- BGC-BGC-BGC-XYS-XYS-GAL**  **2.- NAG-NAG-BMA** |
| **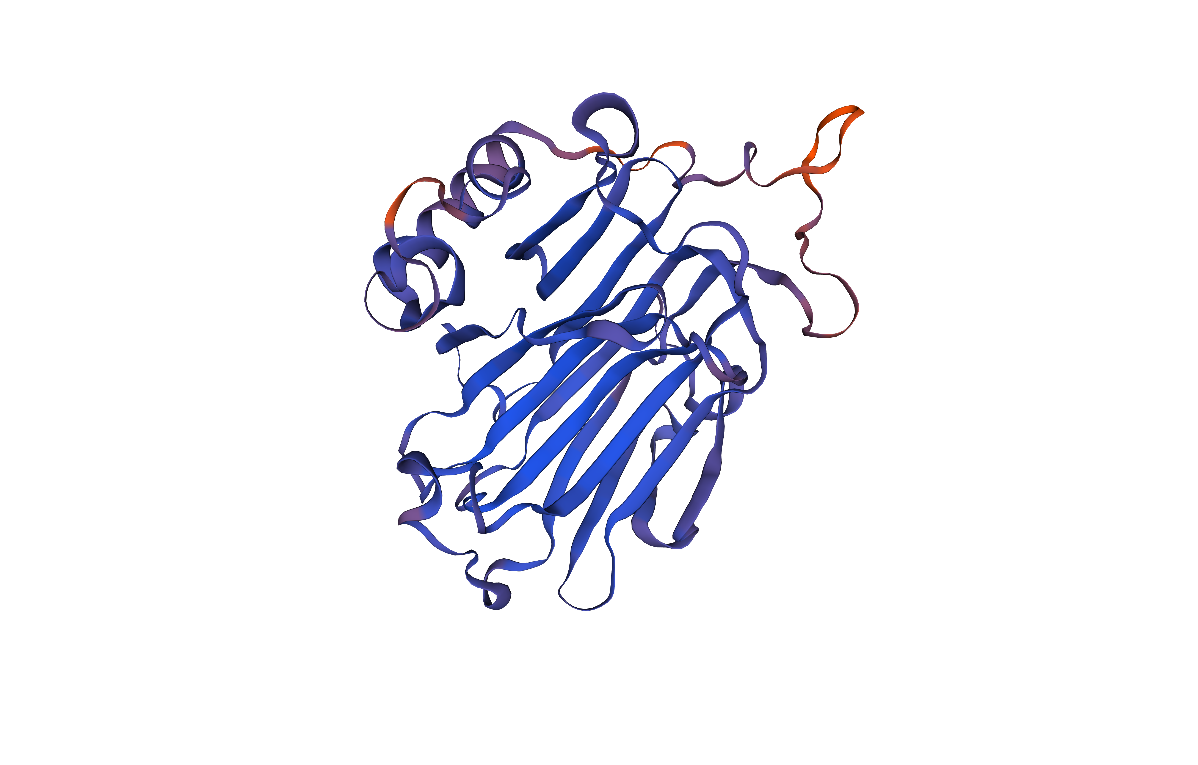**  **SlXTH17** | **Template** | **1umz.1.A** |
|  | **Seq Identity (%)** | **52.06** |
|  | **GMQE** | **0.81** |
|  | **QMEANDisCo** | **0.82 ± 0.05** |
|  | **Ligand** | **1.- BGC-BGC-BGC-XYS-XYS-GAL**  **2.- NAG-NAG-BMA** |
| 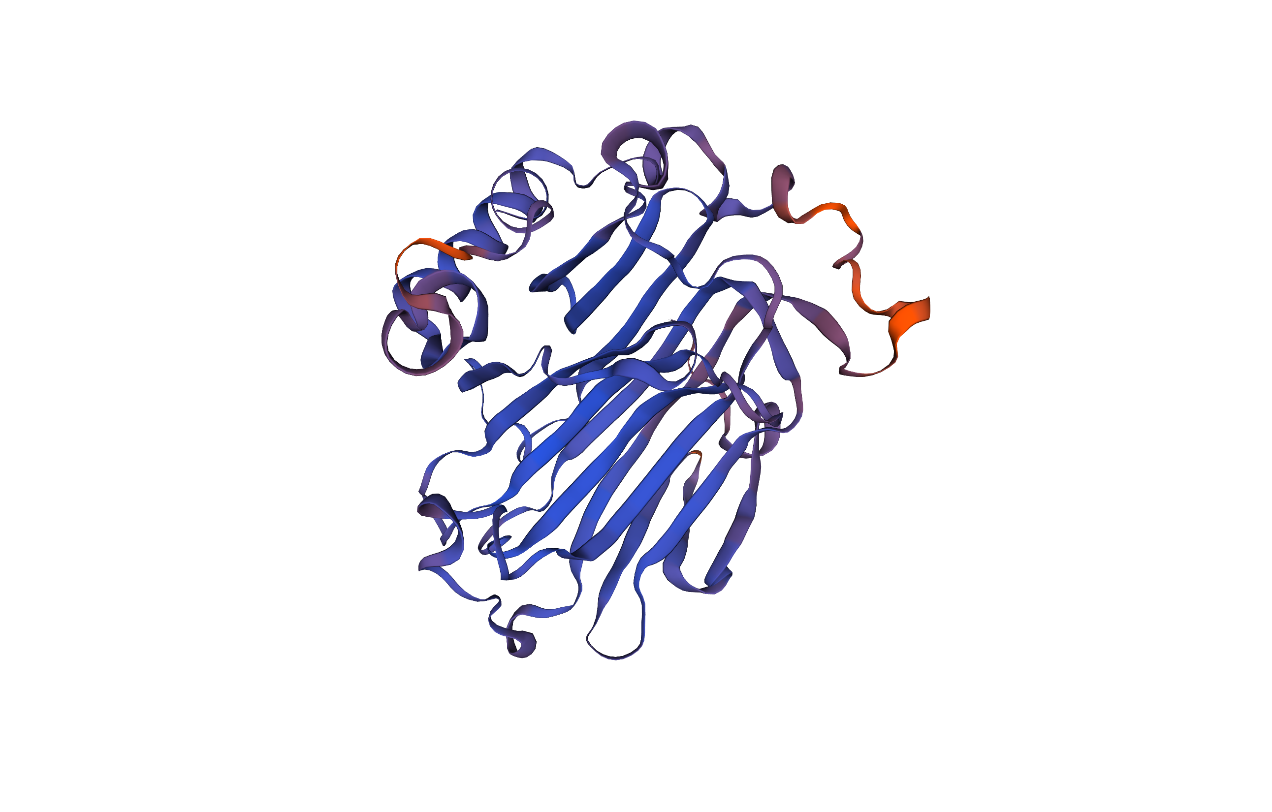  **SlXTH18** | **Template** | **1un1.2.A** |
|  | **Seq Identity (%)** | **47.19** |
|  | **GMQE** | **0.79** |
|  | **QMEANDisCo** | **0.80 ± 0.05** |
|  | **Ligand** | **1.- BGC-BGC-BGC-XYS-XYS-GAL**  **2.- NAG-NAG-BMA** |
| **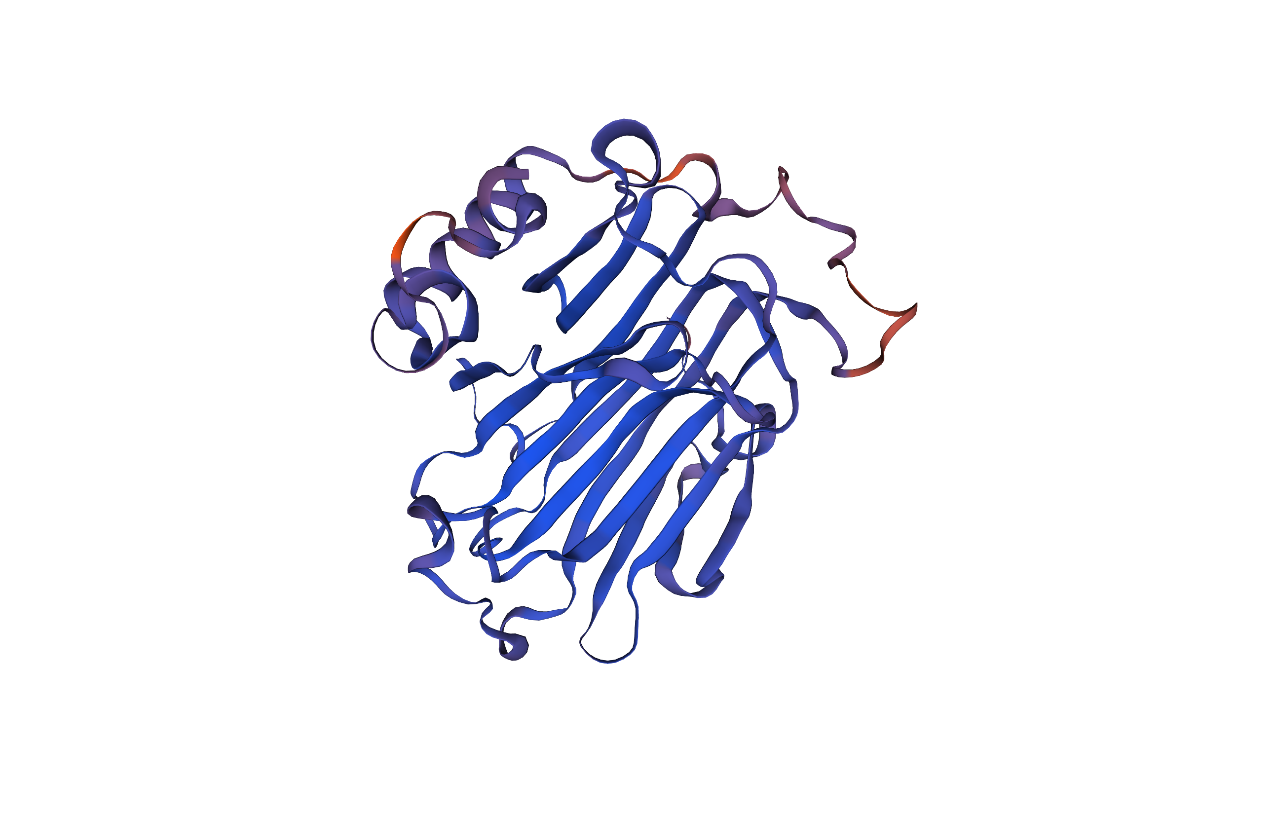**  **SlXTH19** | **Template** | **1umz.1.A** |
|  | **Seq Identity (%)** | **54.17** |
|  | **GMQE** | **0.82** |
|  | **QMEANDisCo** | **0.83 ± 0.05** |
|  | **Ligand** | **1.- BGC-BGC-BGC-XYS-XYS-GAL**  **2.- NAG-NAG-BMA** |
| 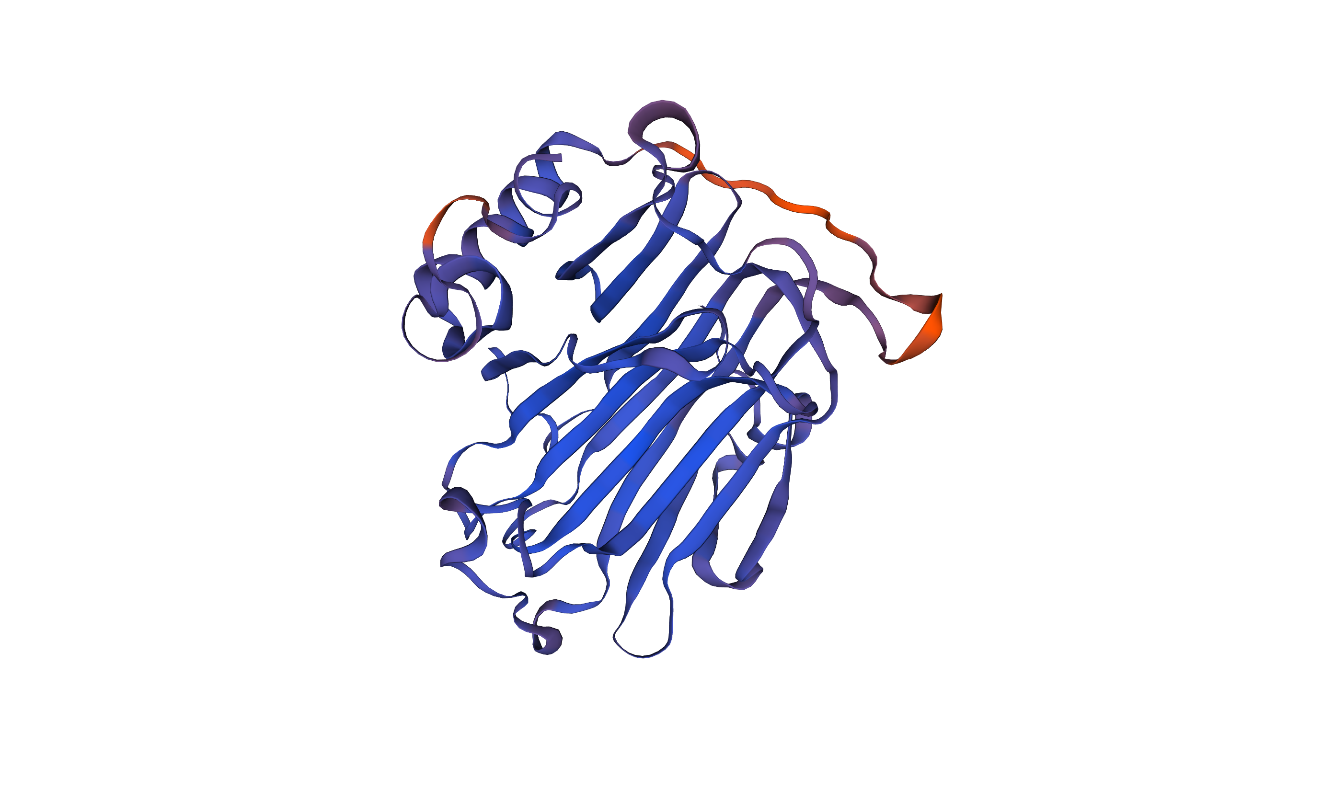  **SlXTH20** | **Template** | **1umz.1.A** |
|  | **Seq Identity (%)** | **55.69** |
|  | **GMQE** | **0.80** |
|  | **QMEANDisCo** | **0.82 ± 0.05** |
|  | **Ligand** | **1.- BGC-BGC-BGC-XYS-XYS-GAL**  **2.- NAG-NAG-BMA** |
| **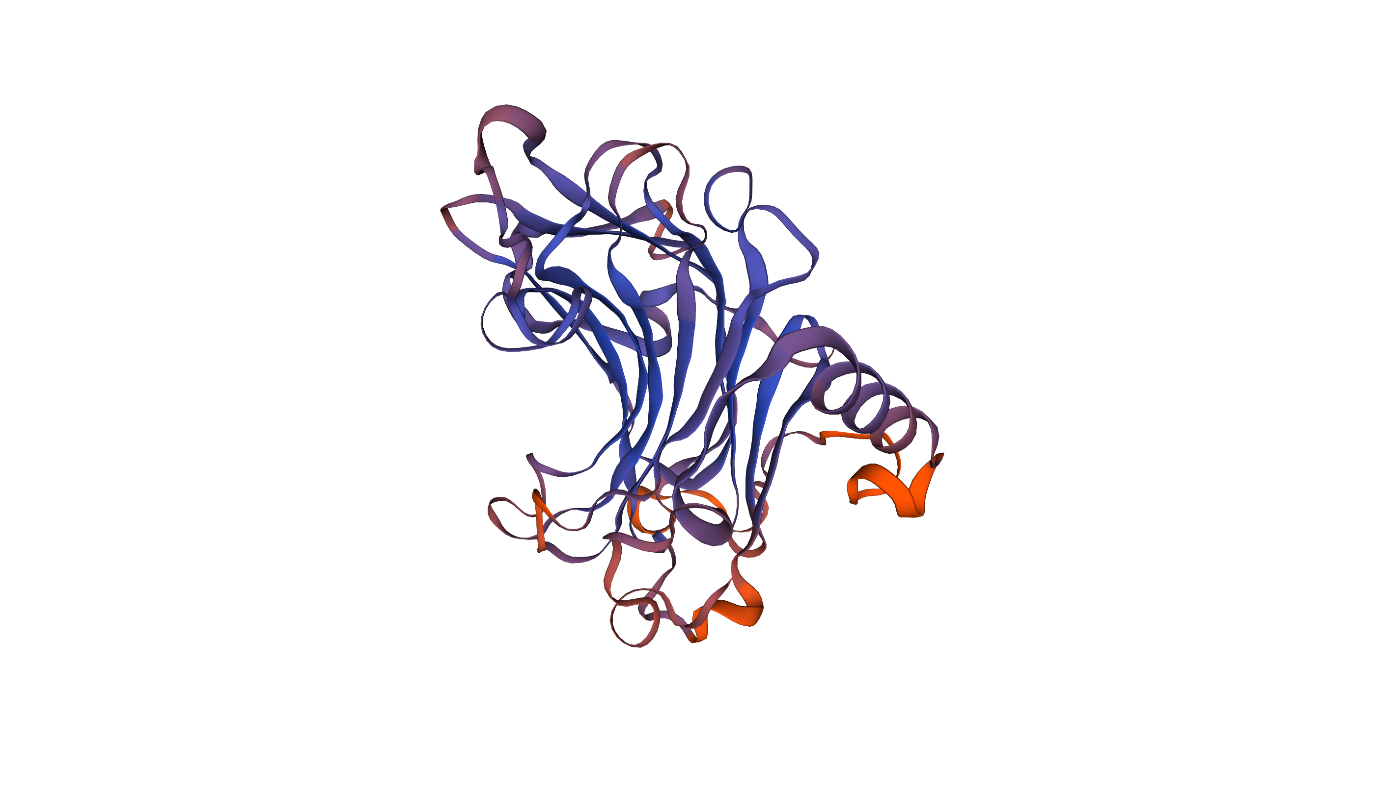**  **SlXTH21** | **Template** | **2uwa.1.A** |
|  | **Seq Identity (%)** | **43.80** |
|  | **GMQE** | **0.63** |
|  | **QMEANDisCo** | **0.73 ± 0.05** |
|  | **Ligand** | **None** |
| 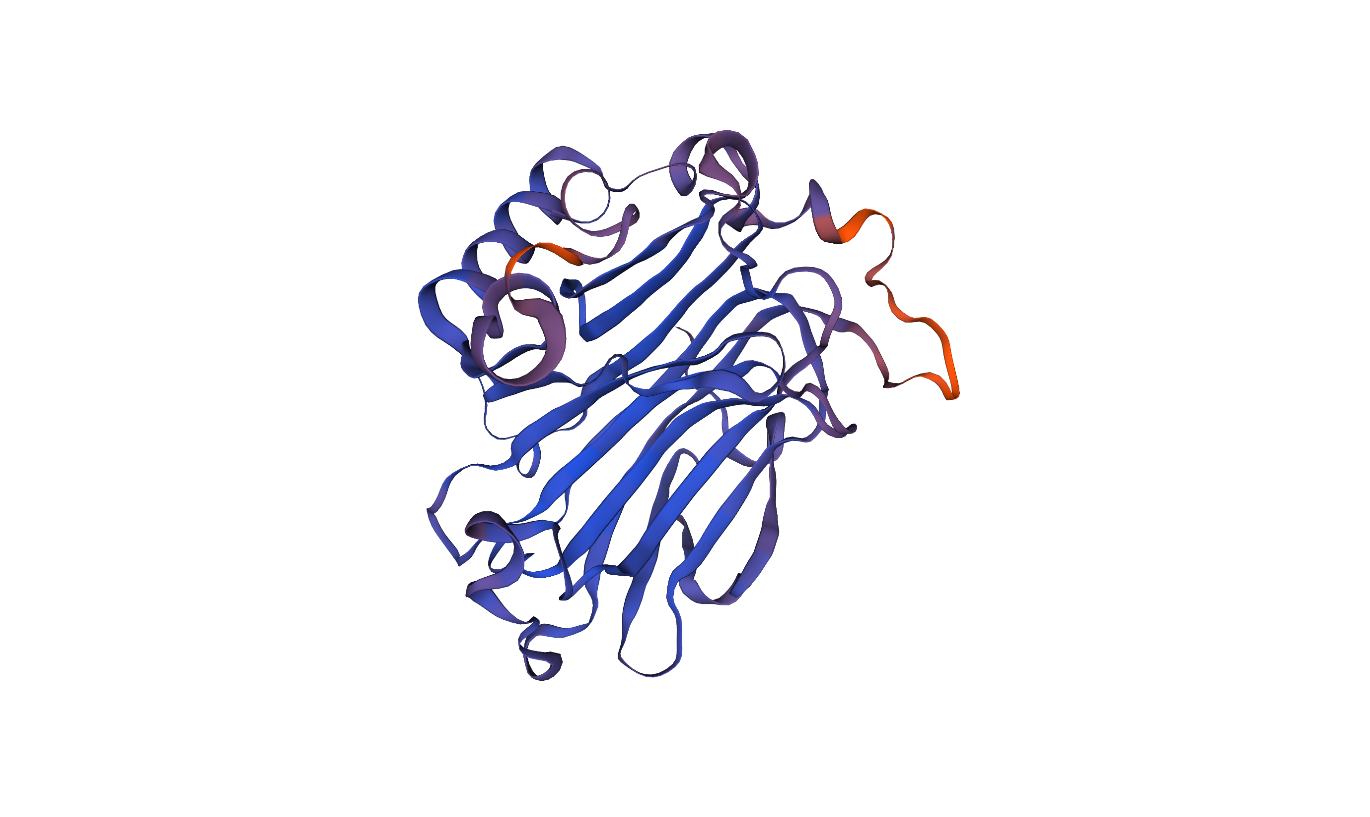  **SlXTH22** | **Template** | **1umz.1.A** |
|  | **Seq Identity (%)** | **49.43** |
|  | **GMQE** | **0.79** |
|  | **QMEANDisCo** | **0.80 ± 0.05** |
|  | **Ligand** | **1.- BGC-BGC-BGC-XYS-XYS-GAL**  **2.- NAG-NAG-BMA** |
| **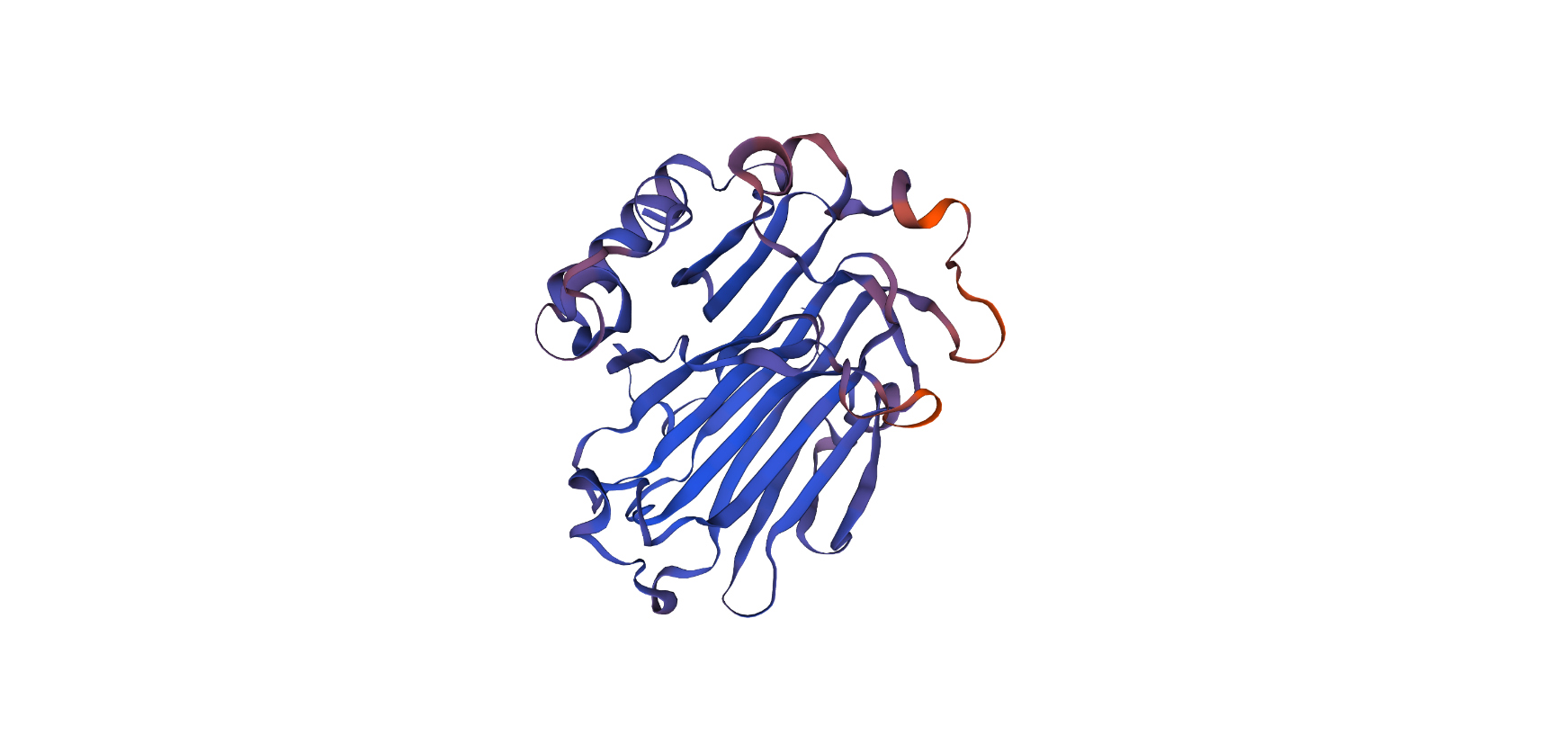**  **SlXTH23** | **Template** | **1umz.1.A** |
|  | **Seq Identity (%)** | **48.50** |
|  | **GMQE** | **0.78** |
|  | **QMEANDisCo** | **0.81 ± 0.05** |
|  | **Ligand** | **1.- BGC-BGC-BGC-XYS-XYS-GAL**  **2.- NAG-NAG-BMA** |
| 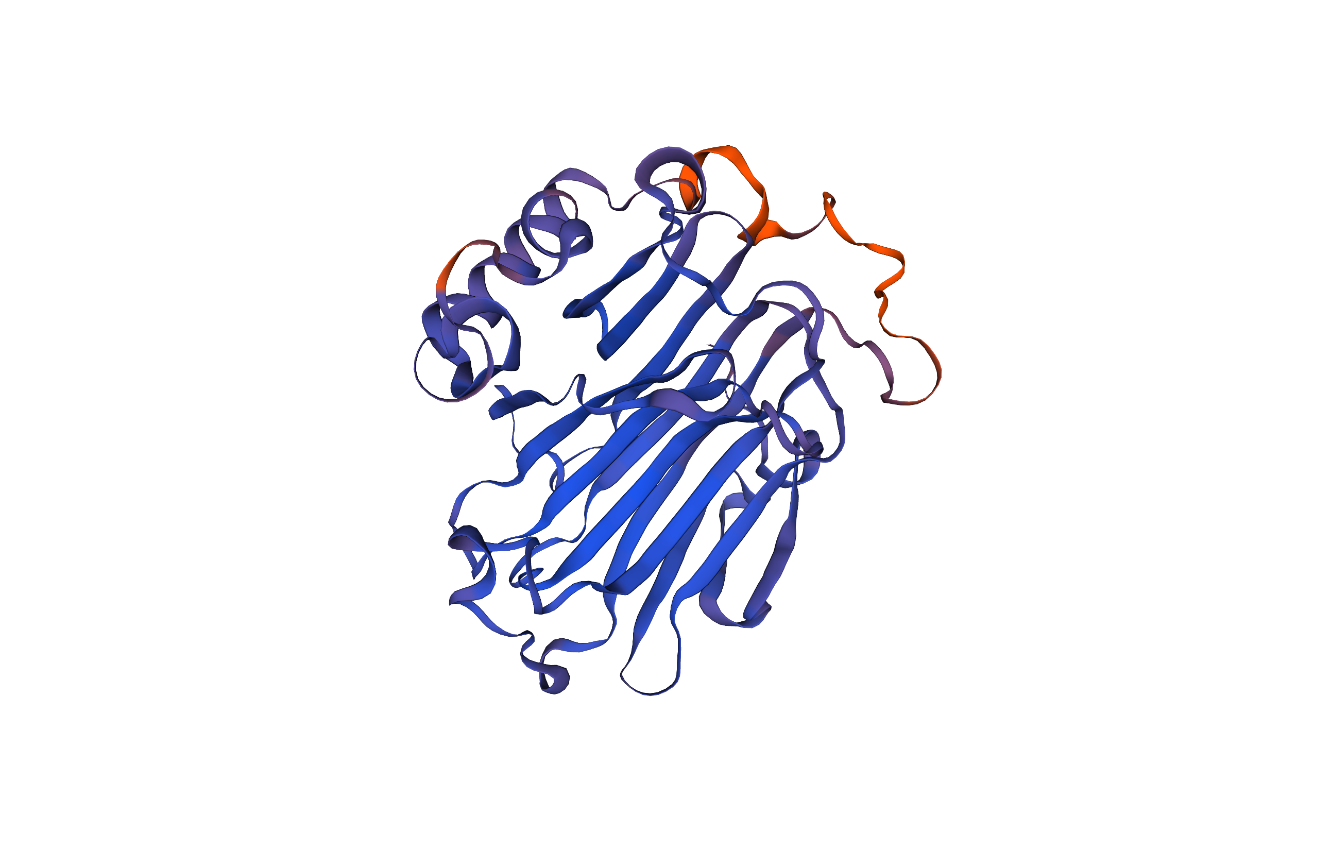  **SlXTH24** | **Template** | **1umz.1.A** |
|  | **Seq Identity (%)** | **51.48** |
|  | **GMQE** | **0.82** |
|  | **QMEANDisCo** | **0.81 ± 0.05** |
|  | **Ligand** | **1.- BGC-BGC-BGC-XYS-XYS-GAL**  **2.- NAG-NAG-BMA** |
| **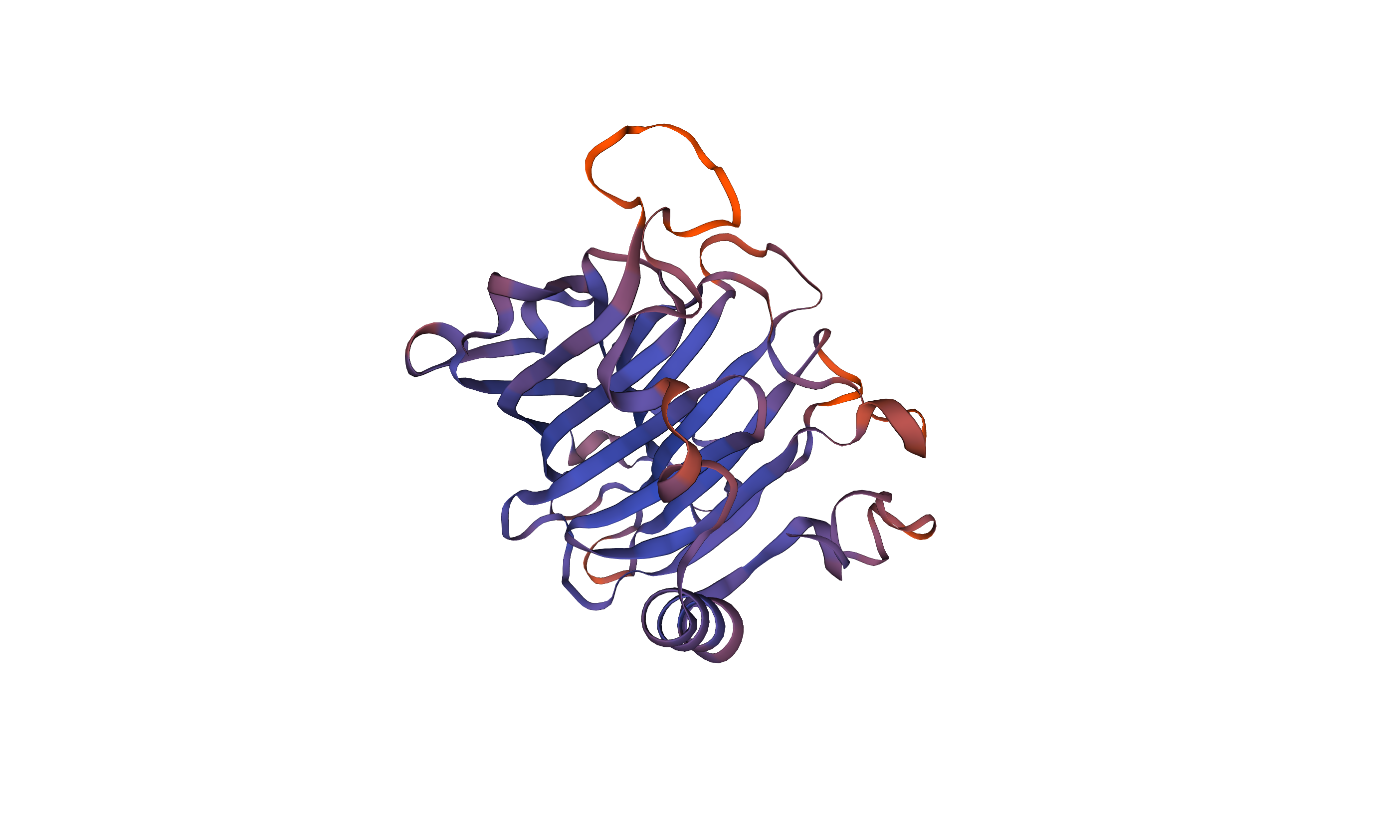**  **SlXTH25** | **Template** | **1umz.1.A** |
|  | **Seq Identity (%)** | **36.54** |
|  | **GMQE** | **0.67** |
|  | **QMEANDisCo** | **0.73 ± 0.05** |
|  | **Ligand** | **1.- BGC-BGC-BGC-XYS-XYS-GAL**  **2.- NAG-NAG-BMA** |
| 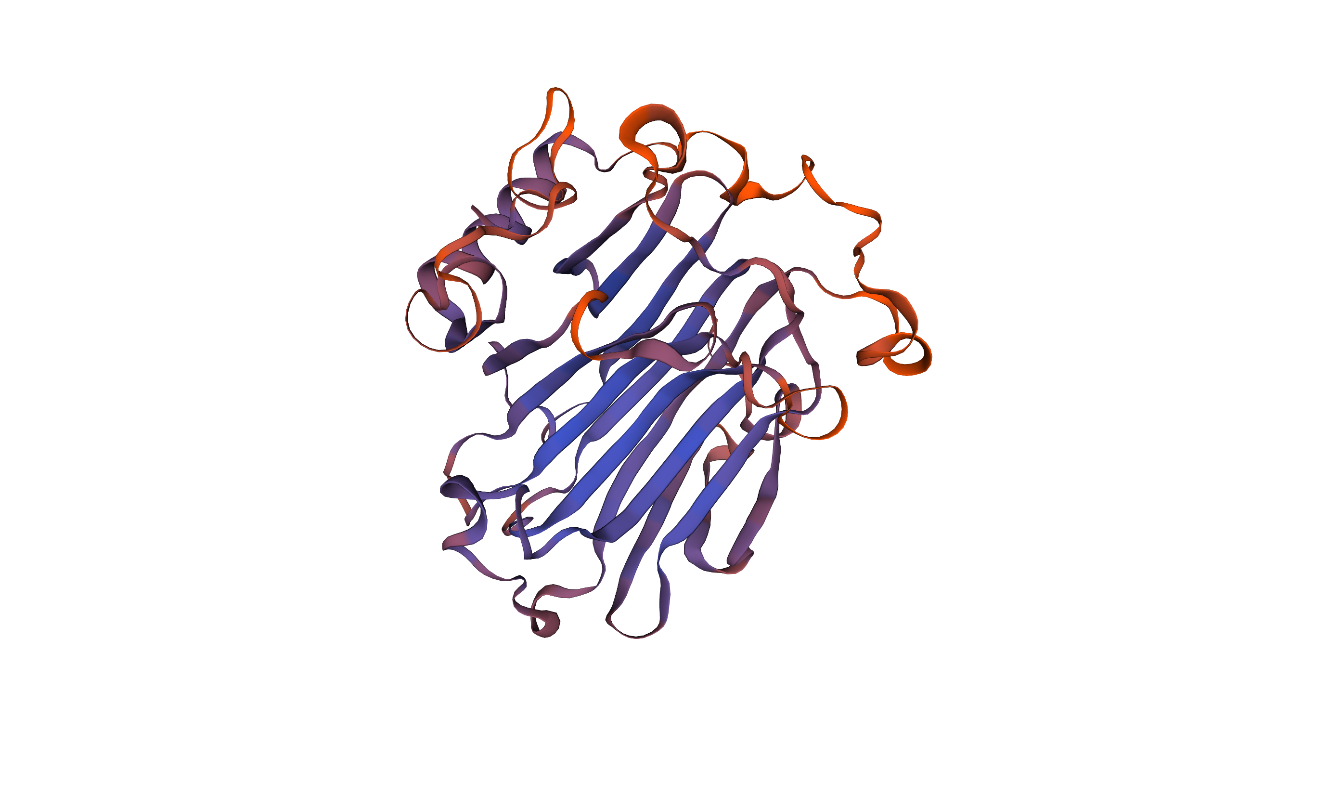  **SlXTH26** | **Template** | **1umz.1.A** |
|  | **Seq Identity (%)** | **36.86** |
|  | **GMQE** | **0.53** |
|  | **QMEANDisCo** | **0.67 ± 0.05** |
|  | **Ligand** | **1.- BGC-BGC-BGC-XYS-XYS-GAL**  **2.- NAG-NAG-BMA** |
| **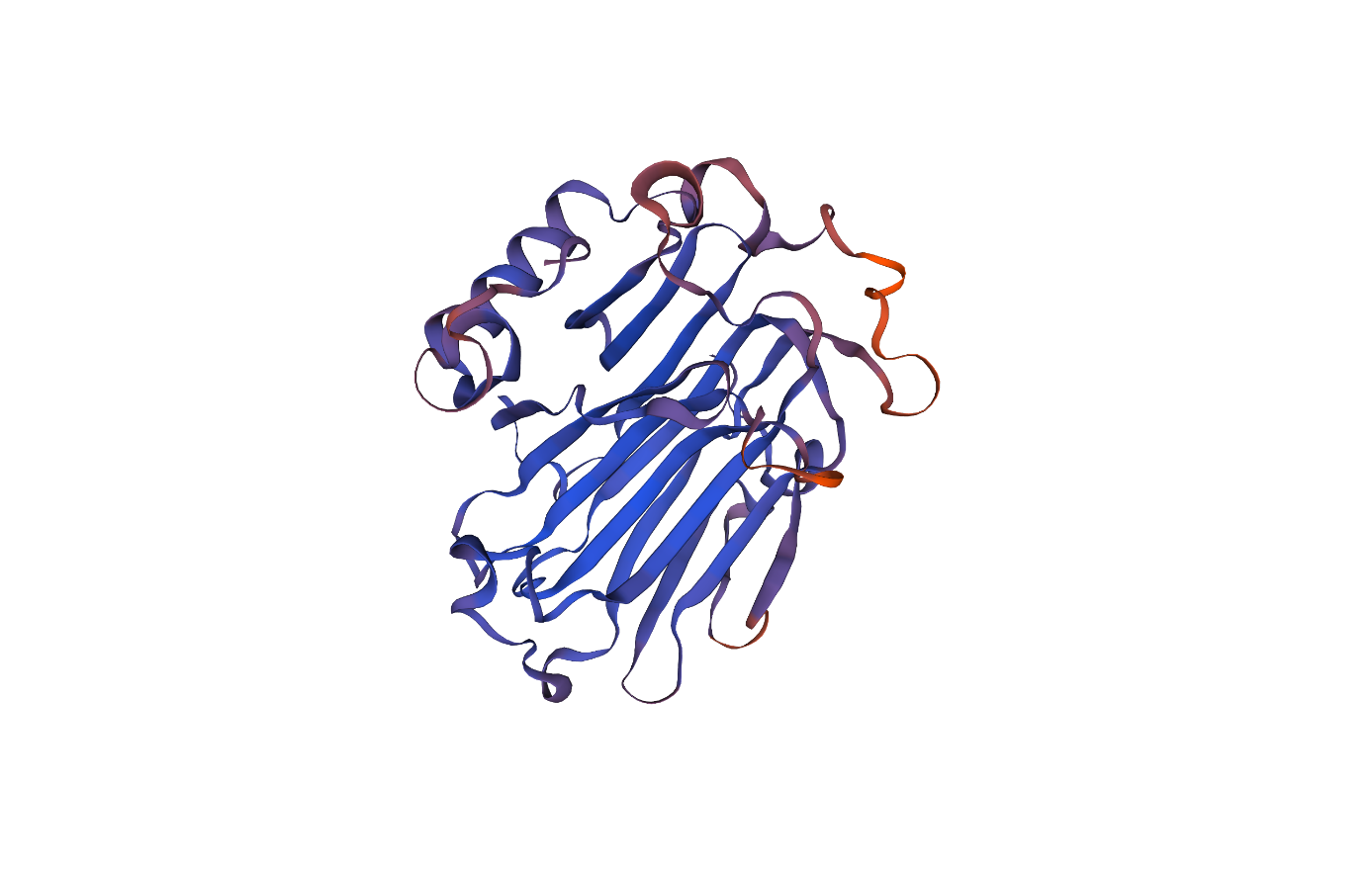**  **SlXTH27** | **Template** | **1umz.1.A** |
|  | **Seq Identity (%)** | **47.58** |
|  | **GMQE** | **0.79** |
|  | **QMEANDisCo** | **0.78 ± 0.05** |
|  | **Ligand** | **1.- BGC-BGC-BGC-XYS-XYS-GAL**  **2.- NAG-NAG-BMA** |
| 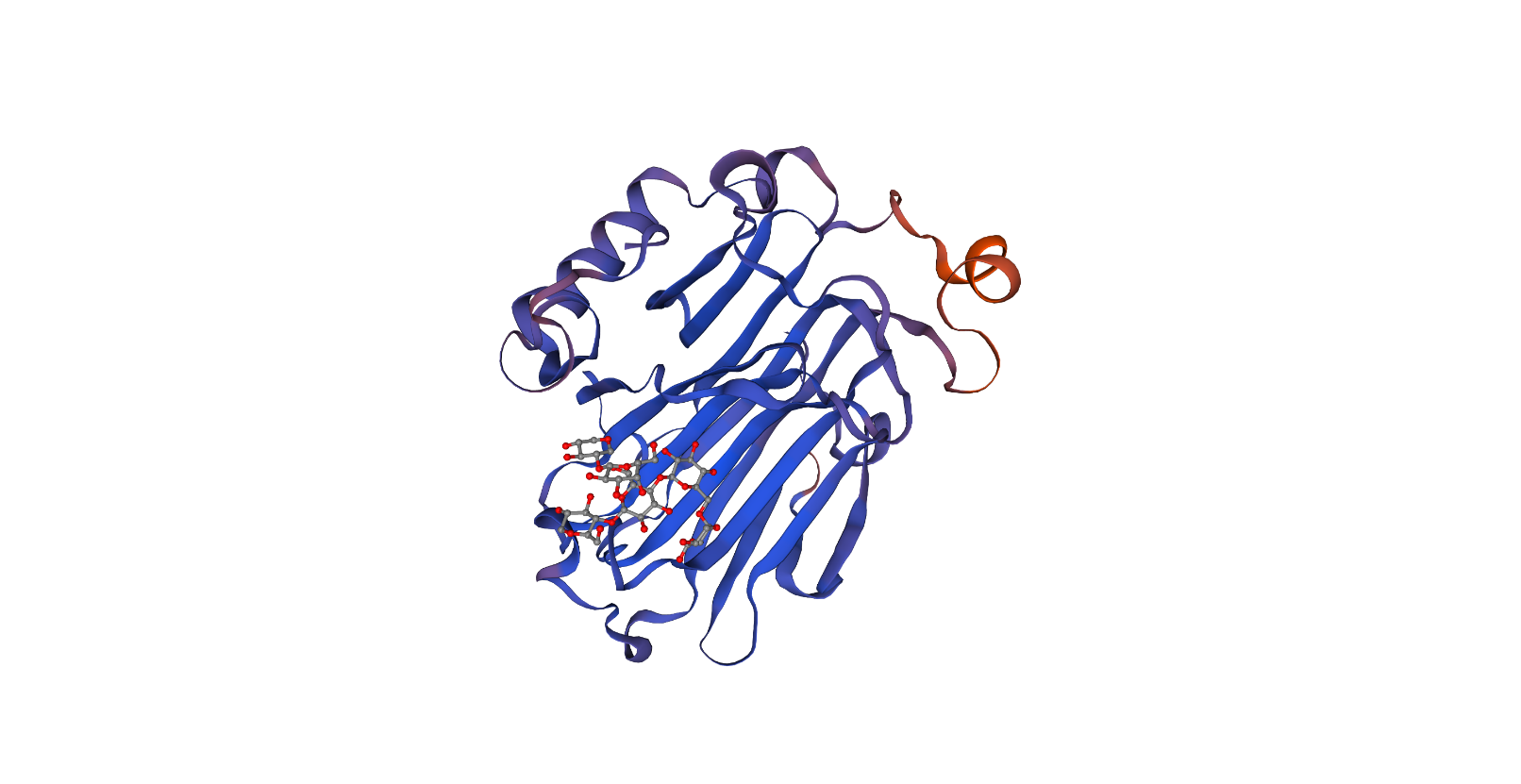  **SlXTH28** | **Template** | **1umz.1.A** |
|  | **Seq Identity (%)** | **51.91** |
|  | **GMQE** | **0.82** |
|  | **QMEANDisCo** | **0.82 ± 0.05** |
|  | **Ligand** | **1.- BGC-BGC-BGC-XYS-XYS-GAL**  **2.- NAG-NAG-BMA** |
| **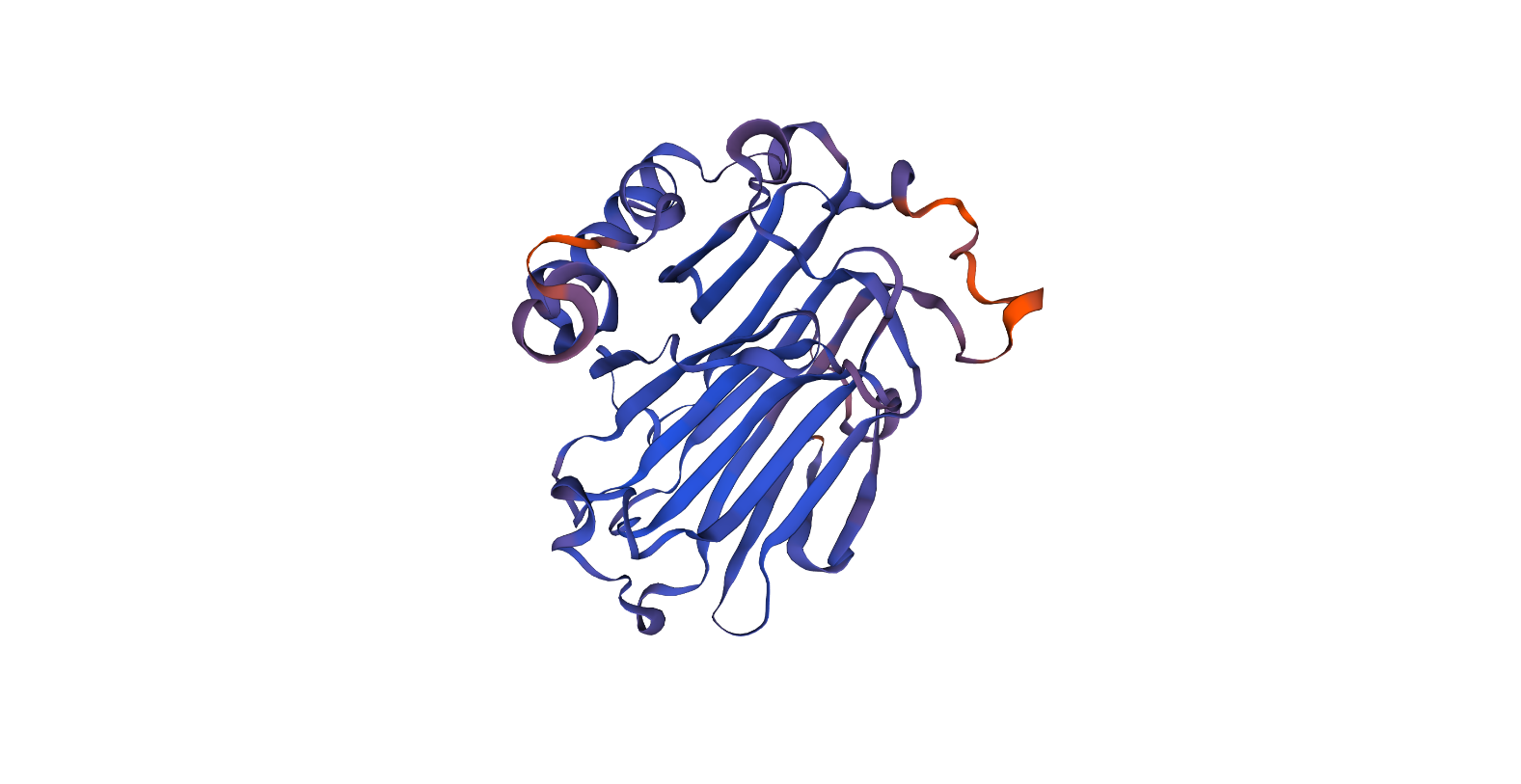**  **SlXTH29** | **Template** | **1umz.1.A** |
|  | **Seq Identity (%)** | **47.17** |
|  | **GMQE** | **0.80** |
|  | **QMEANDisCo** | **0.81 ± 0.05** |
|  | **Ligand** | **1.- BGC-BGC-BGC-XYS-XYS-GAL**  **2.- NAG-NAG-BMA** |
| 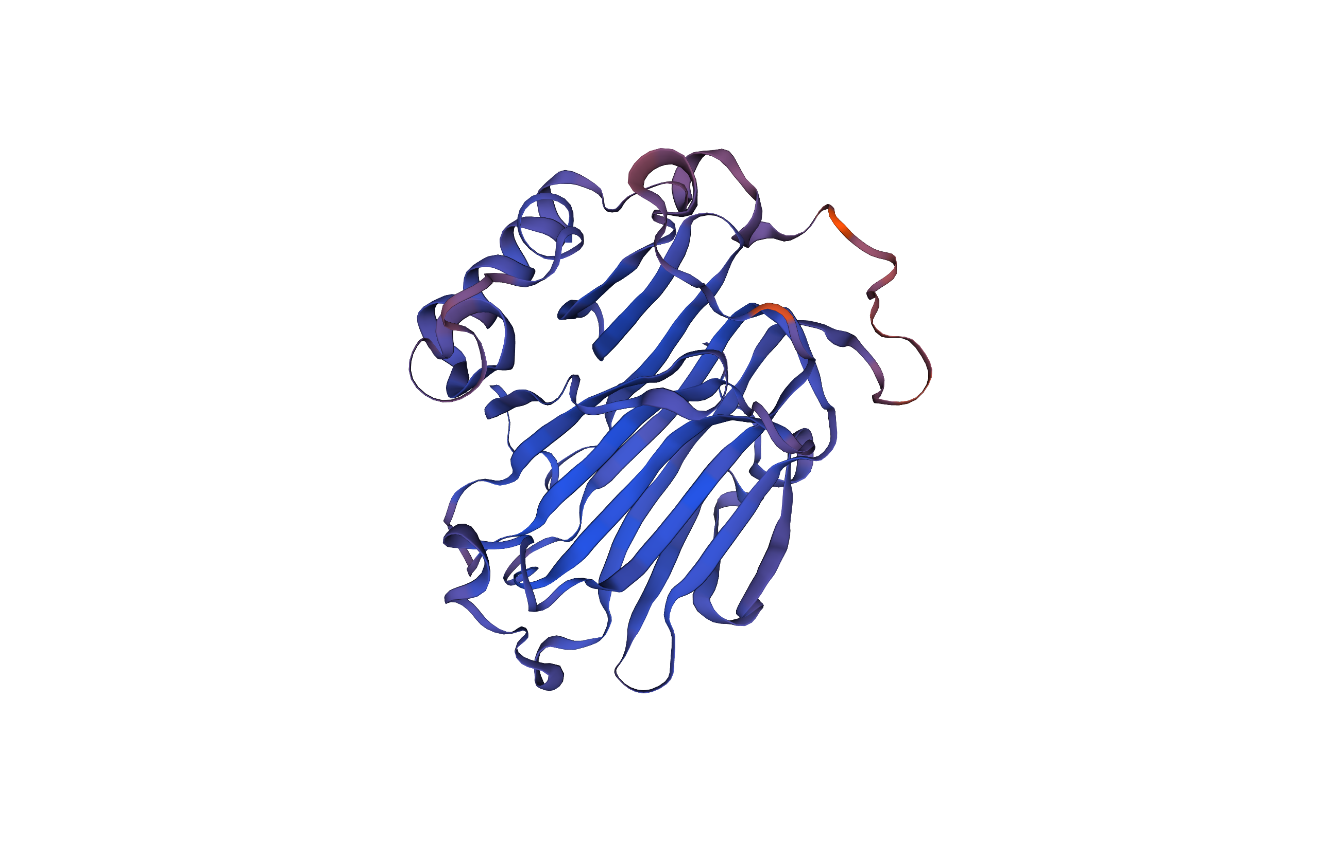  **SlXTH30** | **Template** | **1umz.1.A** |
|  | **Seq Identity (%)** | **47.19** |
|  | **GMQE** | **0.82** |
|  | **QMEANDisCo** | **0.83 ± 0.05** |
|  | **Ligand** | **1.- BGC-BGC-BGC-XYS-XYS-GAL**  **2.- NAG-NAG-BMA** |
| **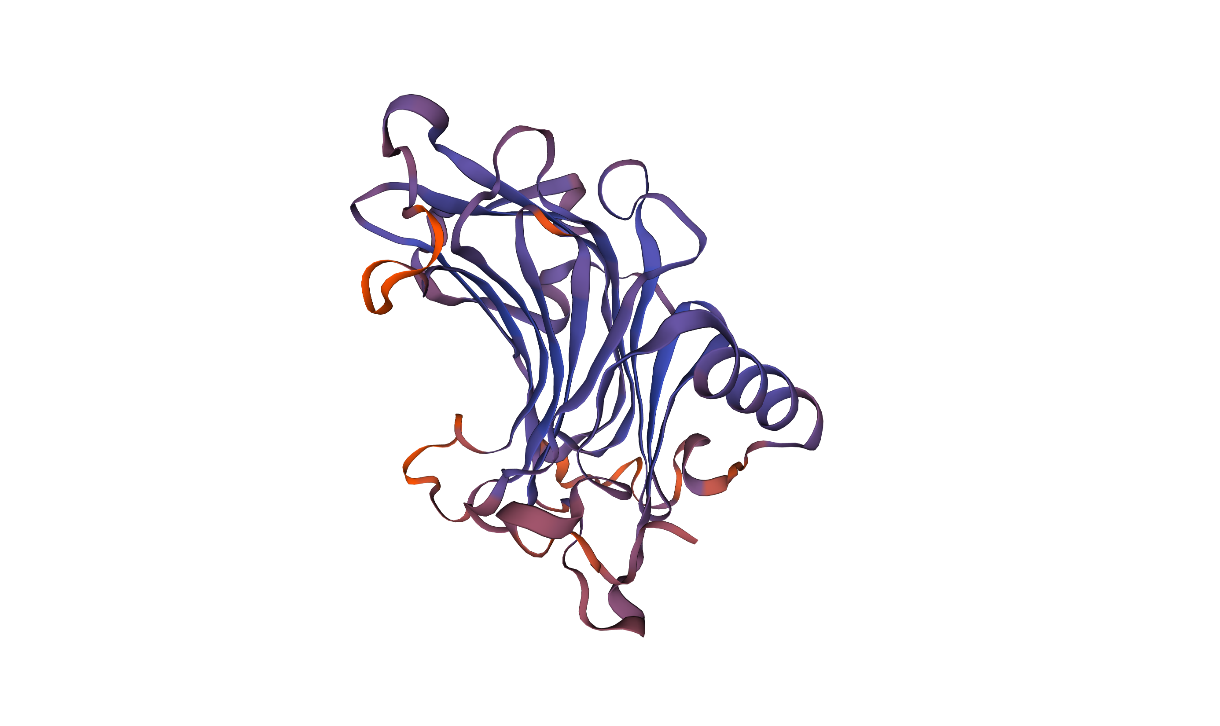**  **SlXTH31** | **Template** | **1umz.1.A** |
|  | **Seq Identity (%)** | **39.27** |
|  | **GMQE** | **0.72** |
|  | **QMEANDisCo** | **0.73 ± 0.05** |
|  | **Ligand** | **1.- BGC-BGC-BGC-XYS-XYS-GAL**  **2.- NAG-NAG-BMA** |
| 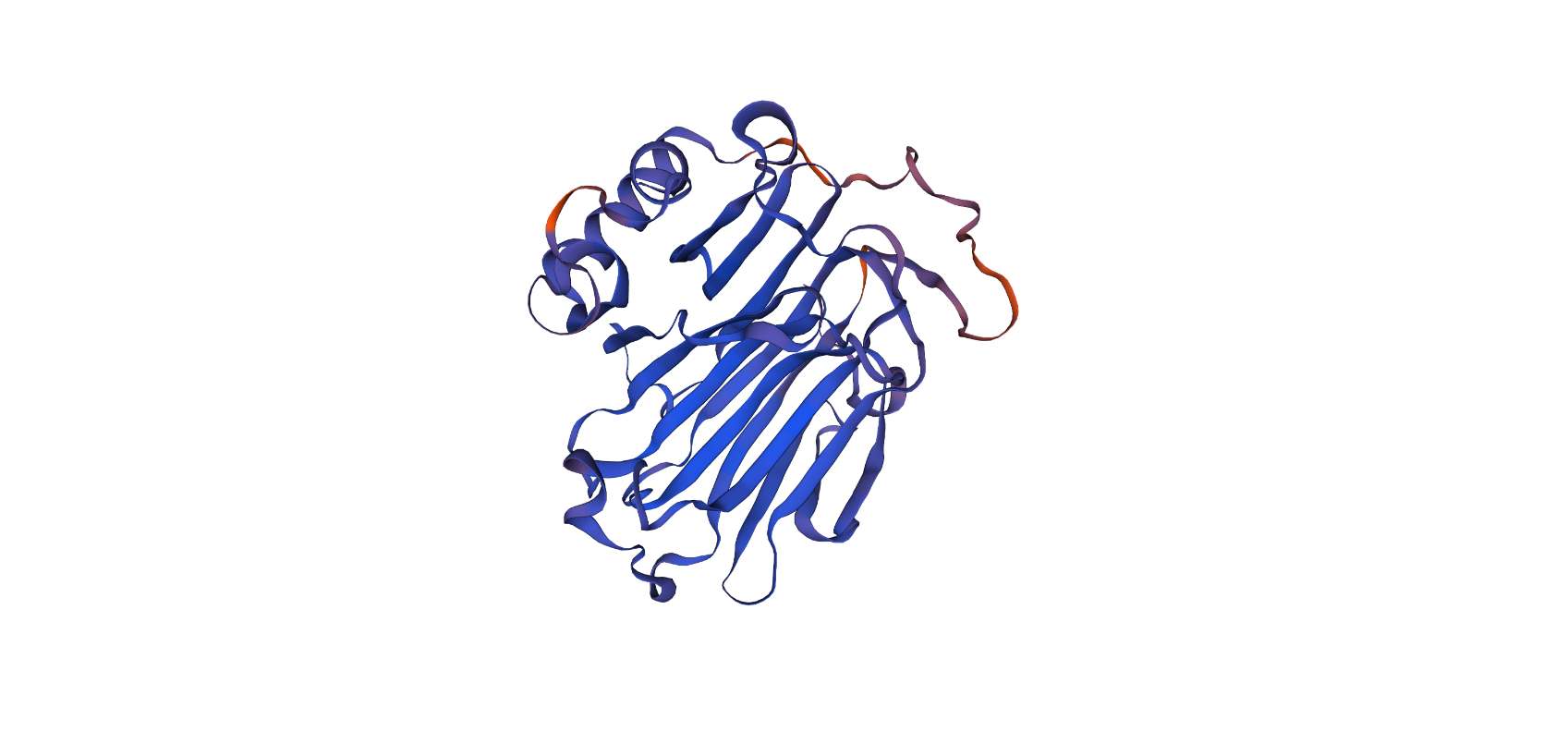  **SlXTH32** | **Template** | **1umz.1.A** |
|  | **Seq Identity (%)** | **51.88** |
|  | **GMQE** | **0.82** |
|  | **QMEANDisCo** | **0.83 ± 0.05** |
|  | **Ligand** | **1.- BGC-BGC-BGC-XYS-XYS-GAL**  **2.- NAG-NAG-BMA** |
| **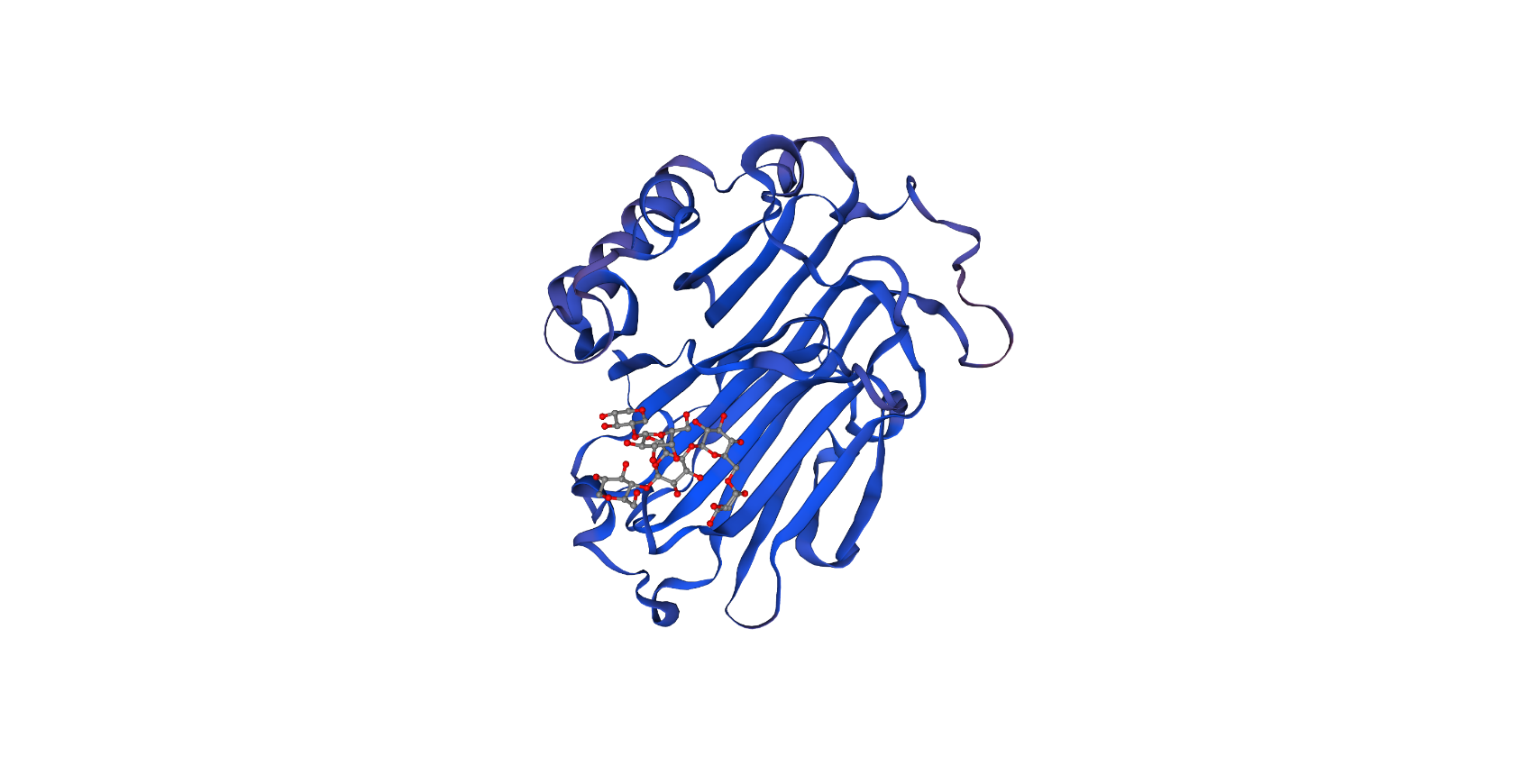**  **SlXTH33** | **Template** | **1umz.1.A** |
|  | **Seq Identity (%)** | **79.03** |
|  | **GMQE** | **0.85** |
|  | **QMEANDisCo** | **0.90 ± 0.05** |
|  | **Ligand** | **1.- BGC-BGC-BGC-XYS-XYS-GAL**  **2.- NAG-NAG-BMA** |
| 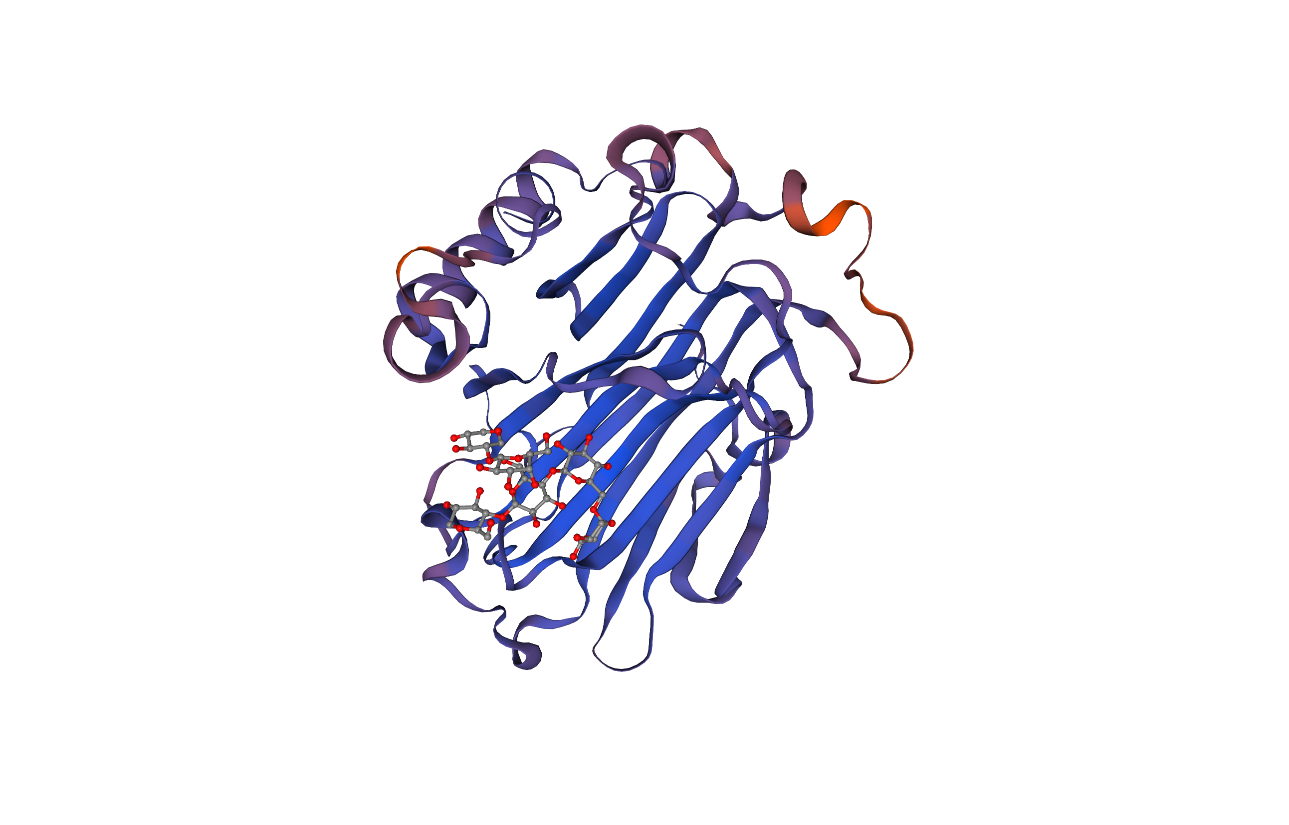  **SlXTH34** | **Template** | **1umz.1.A** |
|  | **Seq Identity (%)** | **47.01** |
|  | **GMQE** | **0.79** |
|  | **QMEANDisCo** | **0.80 ± 0.05** |
|  | **Ligand** | **1.- BGC-BGC-BGC-XYS-XYS-GAL**  **2.- NAG-NAG-BMA** |
| **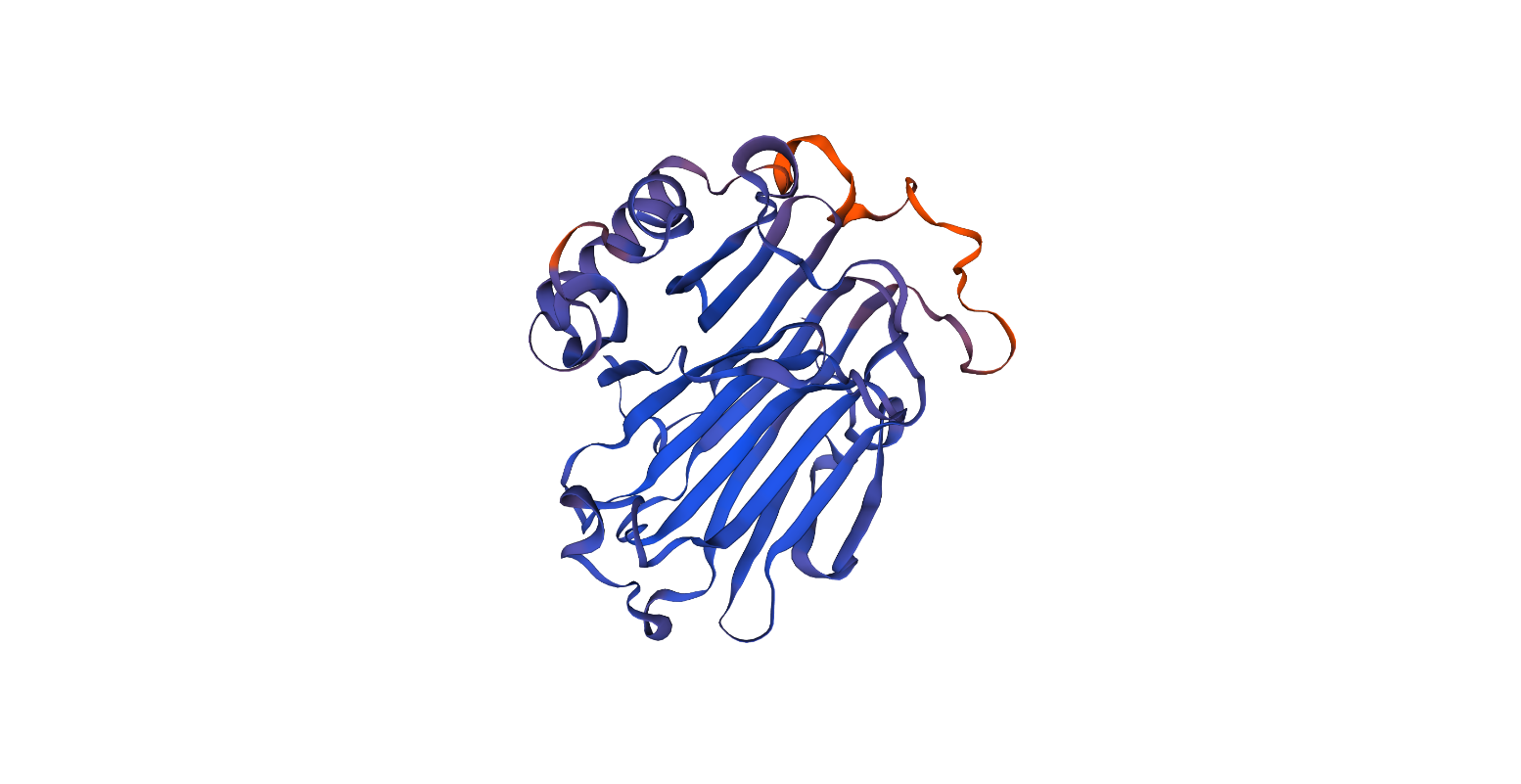**  **SlXTH35** | **Template** | **1umz.1.A** |
|  | **Seq Identity (%)** | **52.40** |
|  | **GMQE** | **0.82** |
|  | **QMEANDisCo** | **0.82 ± 0.05** |
|  | **Ligand** | **1.- BGC-BGC-BGC-XYS-XYS-GAL**  **2.- NAG-NAG-BMA** |
| 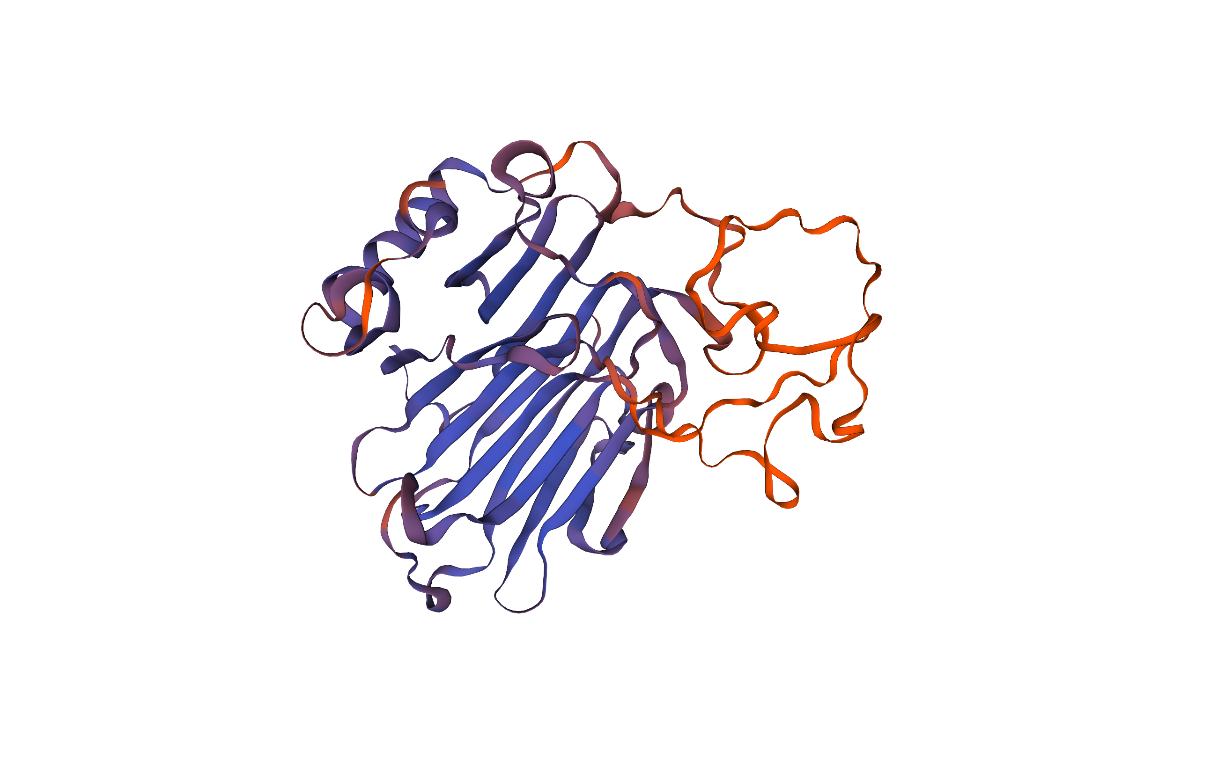  **SlXTH36** | **Template** | **1umz.1.A** |
|  | **Seq Identity (%)** | **37.80** |
|  | **GMQE** | **0.60** |
|  | **QMEANDisCo** | **0.66 ± 0.05** |
|  | **Ligand** | **1.- BGC-BGC-BGC-XYS-XYS-GAL**  **2.- NAG-NAG-BMA** |
| **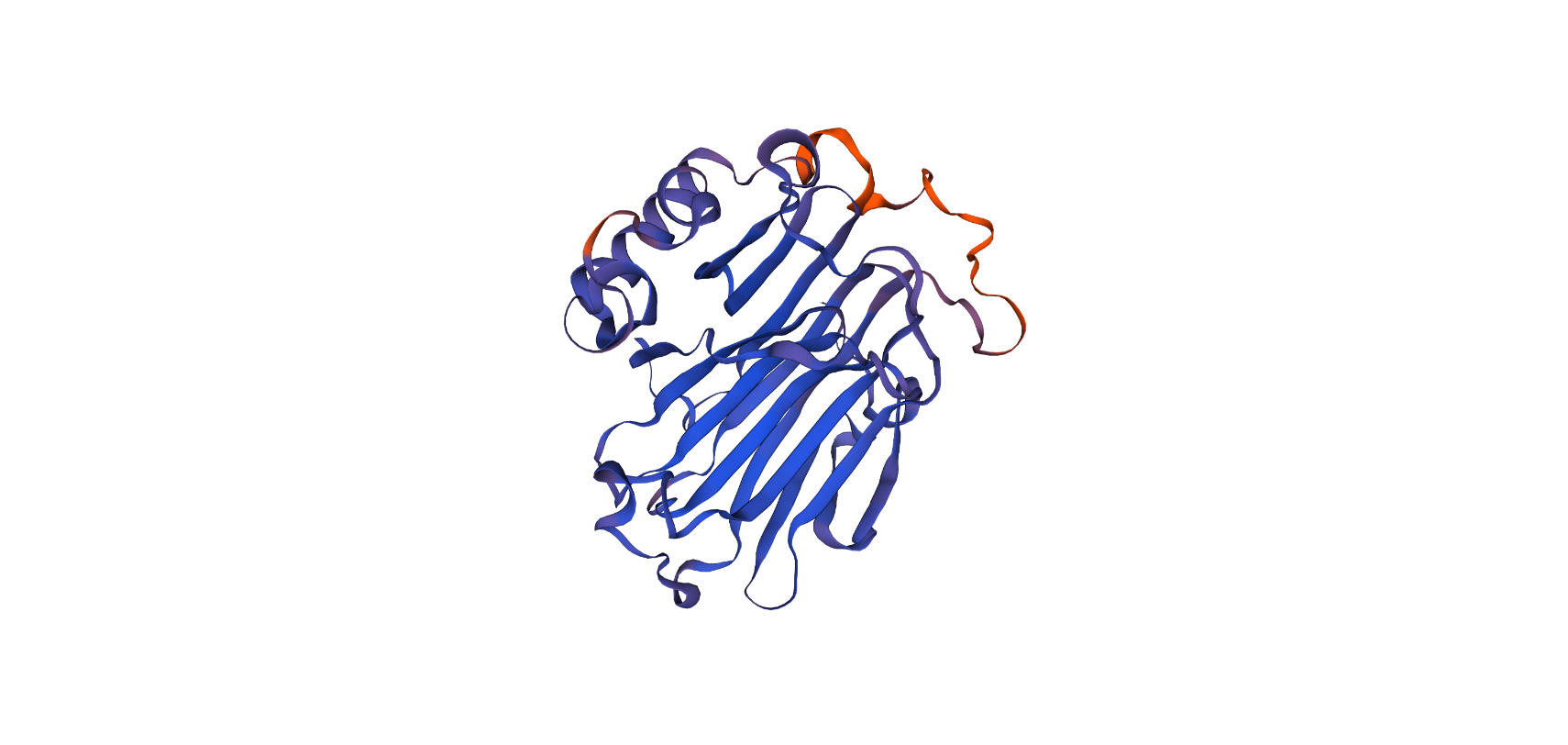**  **SlXTH37** | **Template** | **1umz.1.A** |
|  | **Seq Identity (%)** | **51.11** |
|  | **GMQE** | **0.81** |
|  | **QMEANDisCo** | **0.81 ± 0.05** |
|  | **Ligand** | **1.- BGC-BGC-BGC-XYS-XYS-GAL**  **2.- NAG-NAG-BMA** |

The identity between the template and sequence should be > 30%; The GMQE value is (0-1), and the greater the value, the better the quality; QMEANDisCo value is in (0-1), the larger the value, the better the matching degree. Acronyms of the ligands used in table: BGC-BGC-BGC-XYS-XYS-GAL (alpha-D-xylopyranose-(1-6)-beta-D-glucopyranose-(1-4)-[beta-D-galactopyranose-(1-2)-alpha-D-xylopyranose-(1-6)]beta-D-glucopyranose-(1-4)-beta-D-glucopyranose), NAG-NAG-BMA (beta-D-mannopyranose-(1-4)-2-acetamido-2-deoxy-beta-D-glucopyranose-(1-4)-2-acetamido-2-deoxy-beta-D-glucopyranose).
