# Supplementary material for: Genome-wide characterization of the xyloglucan endotransglucosylase/hydrolase gene family in Solanum lycopersicum L. and gene expression analysis in response to arbuscular mycorrhizal symbiosis
Source: PeerJ. 2023 May 3;11:e15257. doi: 10.7717/peerj.15257 (PMC10163873; doi:10.7717/peerj.15257)
Supplement: Supplemental Information 12 [file peerj-11-15257-s012.docx]

**Supplemental table 4.** Tandem duplication of sister pair genes of xyloglucan endotransglucosylase/hydrolase (*XTH*) in tomato (*Solanum lycopersicum* L.).

| Gene pairs | Score | Identity (%) | Similar (%) |
| --- | --- | --- | --- |
| *SlXTH3/SlXTH37* | 1932 | 98.6 | 99.3 |
| *SlXTH24/SlXTH35* | 1925 | 98.6 | 99.3 |
