# Supplementary material for: Genome-wide characterization of the xyloglucan endotransglucosylase/hydrolase gene family in Solanum lycopersicum L. and gene expression analysis in response to arbuscular mycorrhizal symbiosis
Source: PeerJ. 2023 May 3;11:e15257. doi: 10.7717/peerj.15257 (PMC10163873; doi:10.7717/peerj.15257)
Supplement: Supplemental Information 13 [file peerj-11-15257-s013.docx]

**Table S5. S**egmental duplication of xyloglucan endotransglucosylase/hydrolase (*XTH*) genes pairs in tomato (*Solanum lycopersicum* L.).

| Sub-family | Duplicate genes | Ka | Ks | Ka/Ks | Duplication Date (MY) | Purifying Selection |
| --- | --- | --- | --- | --- | --- | --- |
| XTH-I/II | *SlXTH36-SlXTH31* | 0.192 | 0.328 | 0.5853 | 10.93 | No |
|  | *SlXTH15-SlXTH27* | 0.068 | 0.222 | 0.3063 | 7.4 | Yes |
|  | *SlXTH4-SlXTH1* | 0.109 | 0.332 | 0.3283 | 11.06 | Yes |
|  | *SlXTH16-SlXTH28* | 0.084 | 0.288 | 0.2916 | 9.6 | Yes |
|  | *SlXTH18-SlXTH29* | 0.097 | 0.265 | 0.3660 | 8.83 | Yes |
|  | *SlXTH10-SlXTH11* | 0.073 | 0.343 | 0.2128 | 11.43 | Yes |
|  | *SlXTH9-SlXTH17* | 0.074 | 0.320 | 0.2312 | 10.66 | Yes |
|  | *SlXTH2-SlXTH19* | 0.091 | 0.372 | 0.2446 | 12.40 | Yes |
|  | *SlXTH24-SlXTH37* | 0.009 | 0.035 | 0.2659 | 233.33 | Yes |
| XTH-III | *SlXTH14-SlXTH6* | 0.215 | 0.369 | 0.5826 | 12.30 | No |
|  | *SlXTH21-SlXTH8* | 0.107 | 0.388 | 0.2757 | 12.93 | Yes |
|  | *SlXTH26-SlXTH5* | 0.271 | 0.436 | 0.6215 | 14.533 | No |

Abbreviations used in table: Ka (nonsynonymous substitutions), Ks (synonymous substitutions), MY (Millions of Years), Ka/Ks<0.5 is considered as purifying selection for tomato Xyloglucan Endotransglucosylase/Hydrolases.
