# Supplementary material for: Genome-wide characterization of the xyloglucan endotransglucosylase/hydrolase gene family in Solanum lycopersicum L. and gene expression analysis in response to arbuscular mycorrhizal symbiosis
Source: PeerJ. 2023 May 3;11:e15257. doi: 10.7717/peerj.15257 (PMC10163873; doi:10.7717/peerj.15257)
Supplement: Supplemental Information 14 [file peerj-11-15257-s014.docx]

**Table S6.** Gene ontology description of each xyloglucan endotransglucosylase/hydrolase (*XTH*) gene in tomato (*Solanum lycopersicum* L.).

|  | Biological process | | | Molecular function | | Cellular component | | |
| --- | --- | --- | --- | --- | --- | --- | --- | --- |
| Gene | Cell wall biogenesis  (GO:0071840) | Cell wall organization  (GO:0071554) | Cellular metabolic process  (GO:0044237) | Hydrolase activity  (GO:0016787) | Transferase activity  (GO:0016740) | Cell wall  (GO:0016020) | Apoplast  (GO:0005576) | Intrinsic component of membrane  (GO:0031224) |
| *SlXTH1* | Yes | Yes | Yes | Yes | Yes | Yes | Yes | Yes |
| *SlXTH2* | Yes | Yes | Yes | Yes | Yes | Yes | Yes | No |
| *SlXTH3* | Yes | Yes | Yes | Yes | Yes | Yes | Yes | No |
| *SlXTH4* | Yes | Yes | Yes | Yes | Yes | Yes | Yes | No |
| *SlXTH5* | Yes | Yes | Yes | Yes | Yes | Yes | Yes | No |
| *SlXTH6* | Yes | Yes | Yes | Yes | Yes | Yes | Yes | No |
| *SlXTH7* | Yes | Yes | Yes | Yes | Yes | Yes | Yes | No |
| *SlXTH8* | Yes | Yes | Yes | Yes | Yes | Yes | Yes | Yes |
| *SlXTH9* | Yes | Yes | Yes | Yes | Yes | Yes | Yes | No |
| *SlXTH10* | Yes | Yes | Yes | Yes | Yes | Yes | Yes | No |
| *SLXTH11* | Yes | Yes | Yes | Yes | Yes | Yes | Yes | No |
| *SlXTH12* | Yes | Yes | Yes | Yes | Yes | Yes | Yes | Yes |
| *SlXTH13* | Yes | Yes | Yes | Yes | Yes | Yes | Yes | Yes |
| *SlXTH14* | Yes | Yes | Yes | Yes | Yes | Yes | Yes | No |
| *SlXTH15* | Yes | Yes | Yes | Yes | Yes | Yes | Yes | No |
| *SlXTH16* | Yes | Yes | Yes | Yes | Yes | Yes | Yes | No |
| *SlXTH17* | Yes | Yes | Yes | Yes | Yes | Yes | Yes | No |
| *SlXTH18* | Yes | Yes | Yes | Yes | Yes | Yes | Yes | Yes |
| *SlXTH19* | Yes | Yes | Yes | Yes | Yes | Yes | Yes | No |
| *SlXTH20* | Yes | Yes | Yes | Yes | Yes | Yes | Yes | No |
| *SlXTH21* | Yes | Yes | Yes | Yes | Yes | Yes | Yes | No |
| *SlXTH22* | Yes | Yes | Yes | Yes | Yes | Yes | Yes | Yes |
| *SlXTH23* | Yes | Yes | Yes | Yes | Yes | Yes | Yes | No |
| *SlXTH24* | Yes | Yes | Yes | Yes | Yes | Yes | Yes | No |
| *SlXTH25* | Yes | Yes | Yes | Yes | Yes | Yes | Yes | Yes |
| *SlXTH26* | Yes | Yes | Yes | Yes | Yes | Yes | Yes | No |
| *SlXTH27* | Yes | Yes | Yes | Yes | Yes | Yes | Yes | No |
| *SlXTH28* | Yes | Yes | Yes | Yes | Yes | Yes | Yes | No |
| *SlXTH29* | Yes | Yes | Yes | Yes | Yes | Yes | Yes | Yes |
| *SlXTH30* | Yes | Yes | Yes | Yes | Yes | Yes | Yes | No |
| *SlXTH31* | Yes | Yes | Yes | Yes | Yes | Yes | Yes | Yes |
| *SlXTH32* | Yes | Yes | Yes | Yes | Yes | Yes | Yes | No |
| *SlXTH33* | Yes | Yes | Yes | Yes | Yes | Yes | Yes | No |
| *SlXTH34* | Yes | Yes | Yes | Yes | Yes | Yes | Yes | Yes |
| *SlXTH35* | Yes | Yes | Yes | Yes | Yes | Yes | Yes | No |
| *SlXTH36* | Yes | Yes | Yes | Yes | Yes | No | No | No |
| *SlXTH37* | Yes | Yes | Yes | Yes | Yes | Yes | Yes | No |

GO Identifier indicates the annotation in which each gene is involved in the different biological processes, molecular functions and cellular components.
