# Supplementary material for: Genome-wide characterization of the xyloglucan endotransglucosylase/hydrolase gene family in Solanum lycopersicum L. and gene expression analysis in response to arbuscular mycorrhizal symbiosis
Source: PeerJ. 2023 May 3;11:e15257. doi: 10.7717/peerj.15257 (PMC10163873; doi:10.7717/peerj.15257)
Supplement: Supplemental Information 15 [file peerj-11-15257-s015.docx]

**Table S7**. Student’s t-test p values obtained from relative expression (RT-qPCR) of colonized vs. non-colonized tomato leaves and roots.

| Gene | Leaves *p* value | Roots *p* values |
| --- | --- | --- |
| *XTH2* | 0.0001 | 0.3279 |
| *XTH3* | 0.0001 | 0.0012 |
| *XTH6* | 0.0001 | 0.1798 |
| *XTH7* | 0.0023 | 0.0001 |
| *XTH9* | 0.0001 | 0.1723 |
| *XTH14* | 0.0008 | 0.0767 |
| *XTH17* | 0.3995 | ------- |
| *XTH21* | 0.0001 | 0.0033 |
| *XTH35* | 0.0001 | 0.0002 |
