# Supplementary figures and images for: Genome-wide characterization of the xyloglucan endotransglucosylase/hydrolase gene family in Solanum lycopersicum L. and gene expression analysis in response to arbuscular mycorrhizal symbiosis
Source: PeerJ. 2023 May 3;11:e15257. doi: 10.7717/peerj.15257 (PMC10163873; doi:10.7717/peerj.15257)

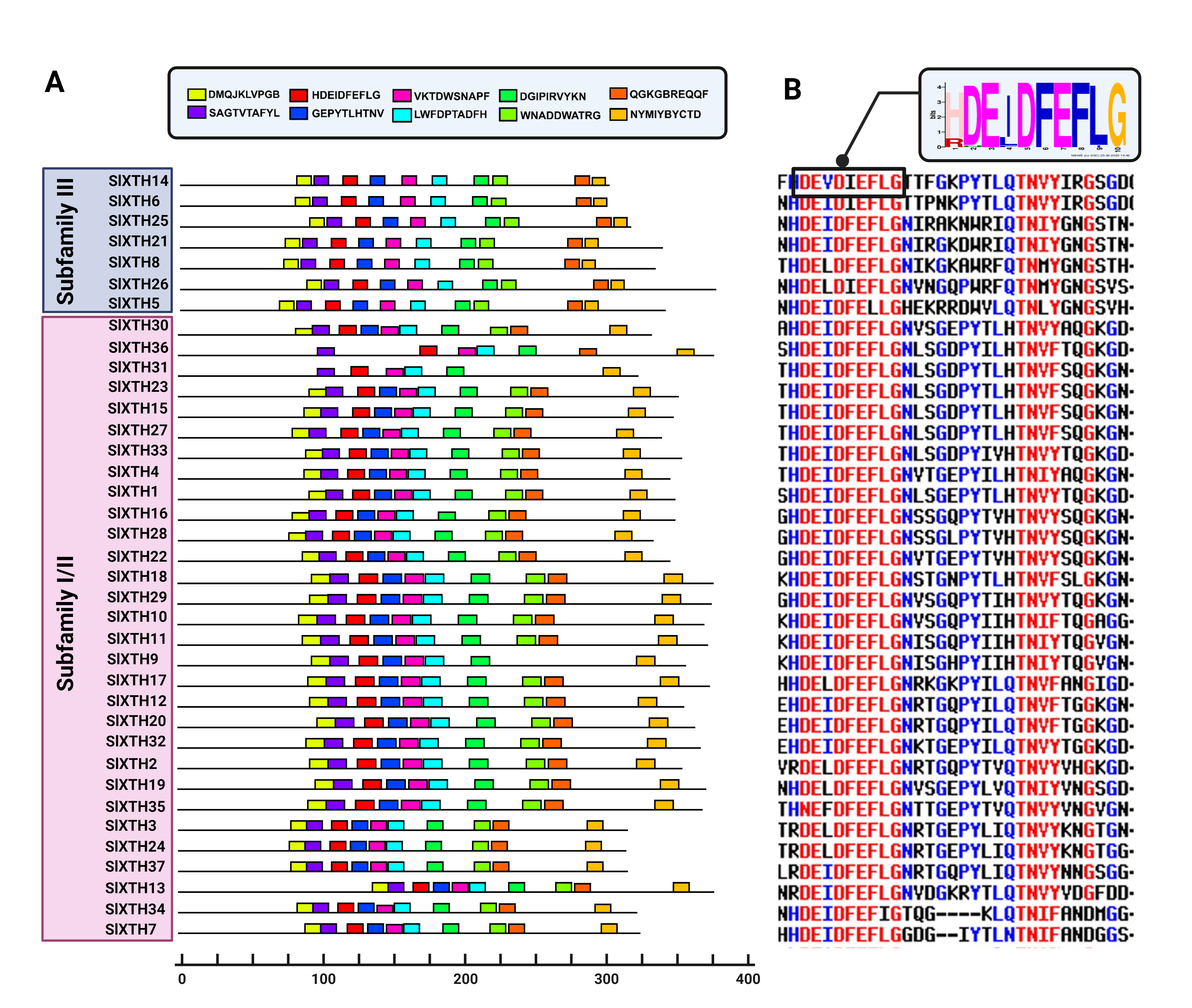

Supplement: Supplemental Information 17 — (A) Location of the ten conserved motifs within SlXTH proteins that were identified using the MEME software. The ten predicted motifs are represented by distinct colored boxes, and the grey lines indicate non-conserved regions. The length of the sequences can be estimated using the scale in base pairs at the bottom. (B) Sequence alignment of the catalytic conserved motif in SlXTH proteins. The sequence logo was created with the 37 SlXTH protein sequences using the MEME software. [file peerj-11-15257-s017.png]

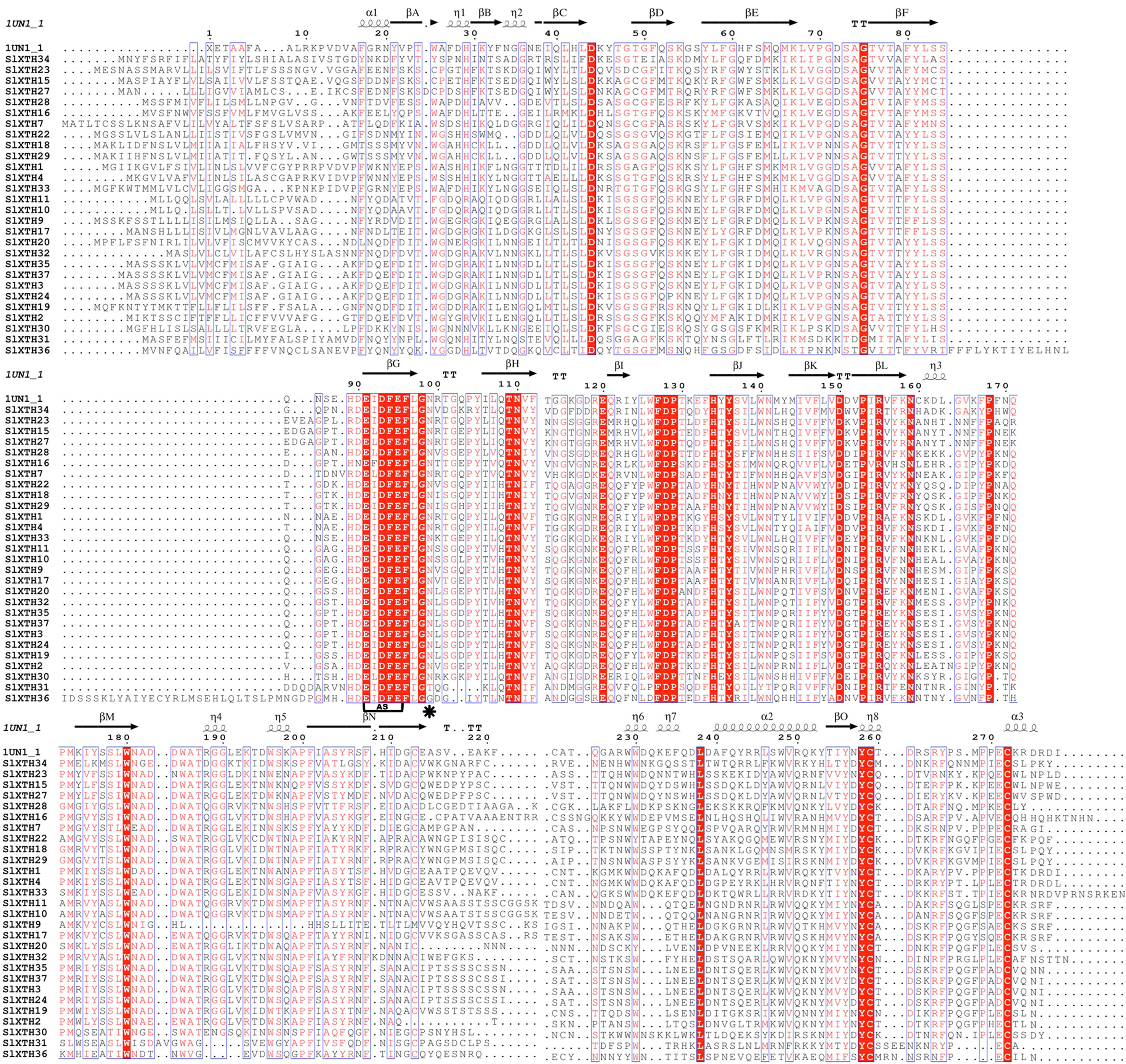

Supplement: Supplemental Information 18 — Sequences were aligned using MultAlin and secondary structures predicted using ESPript. The secondary structures of β sheets (arrows), α-helices (spiral), and N-glycosylation residues (black asterisks) are indicated. AS, active site. [file peerj-11-15257-s018.png]

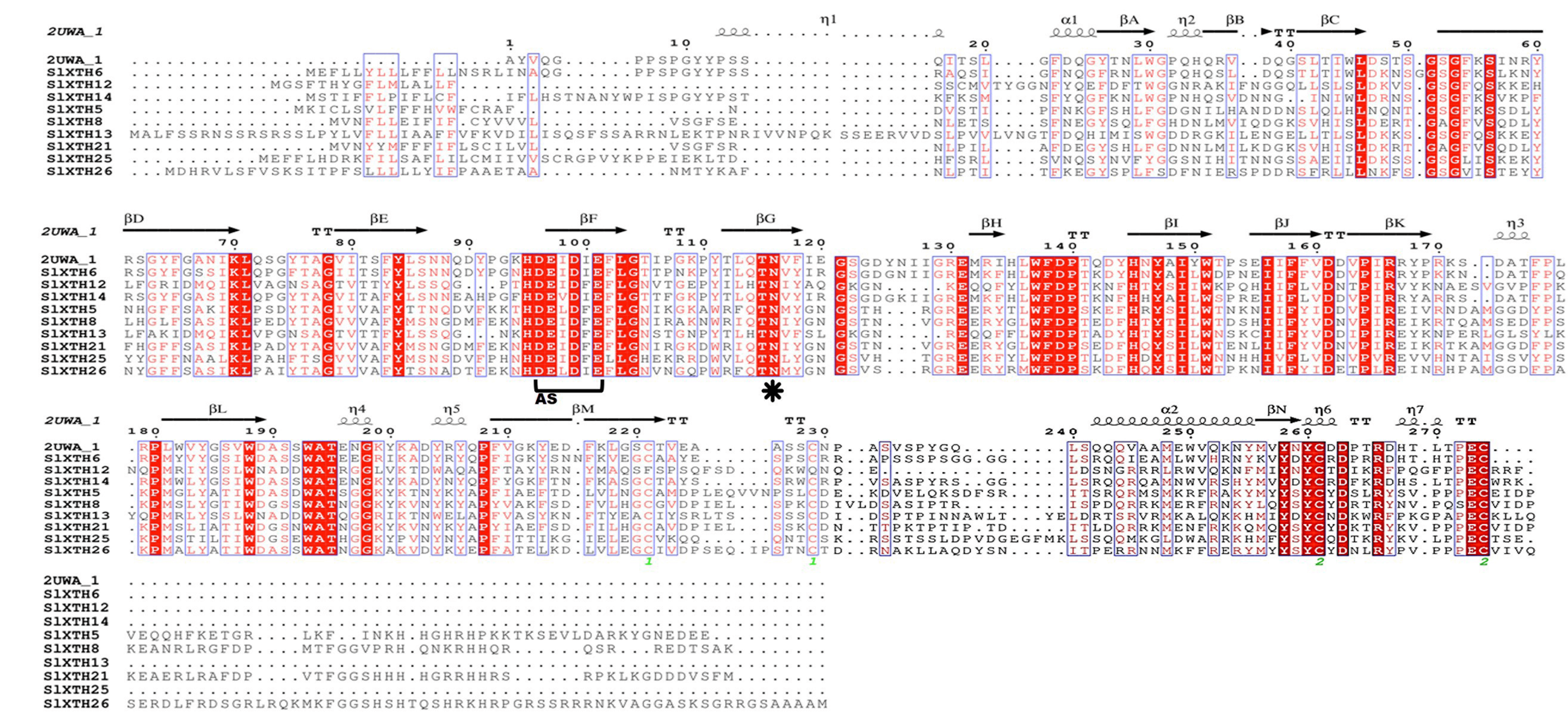

Supplement: Supplemental Information 19 — Sequences were aligned using MultAlin and secondary structures predicted using ESPript. The secondary structures of β sheets (arrows), α-helices (spiral), and N-glycosylation residues (black asterisks) are indicated. AS, active site. [file peerj-11-15257-s019.png]

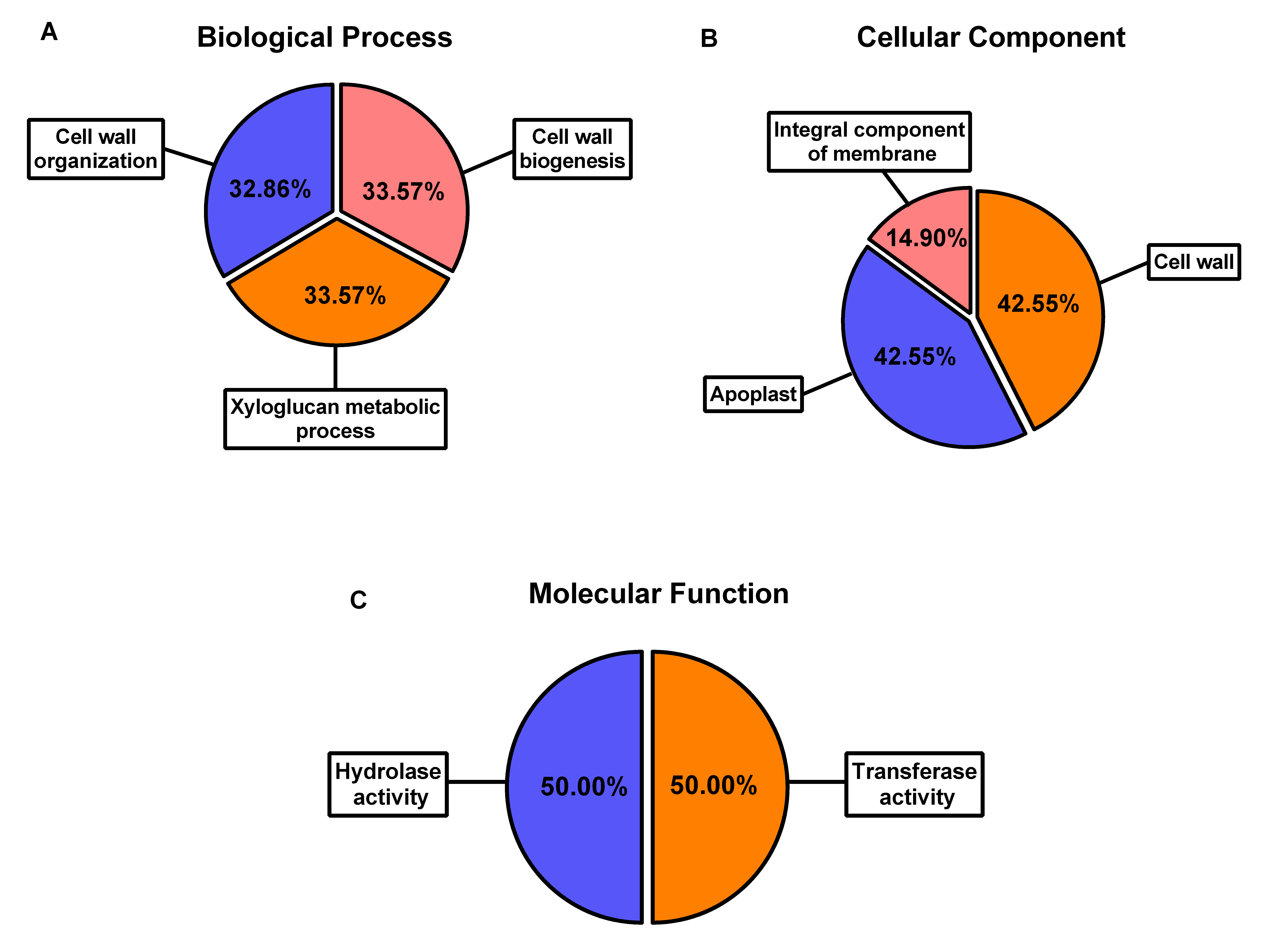

Supplement: Supplemental Information 20 — (A) Biological process, (B) cellular component, and (C) molecular function of the Sl XTH gene family in tomato. [file peerj-11-15257-s020.png]

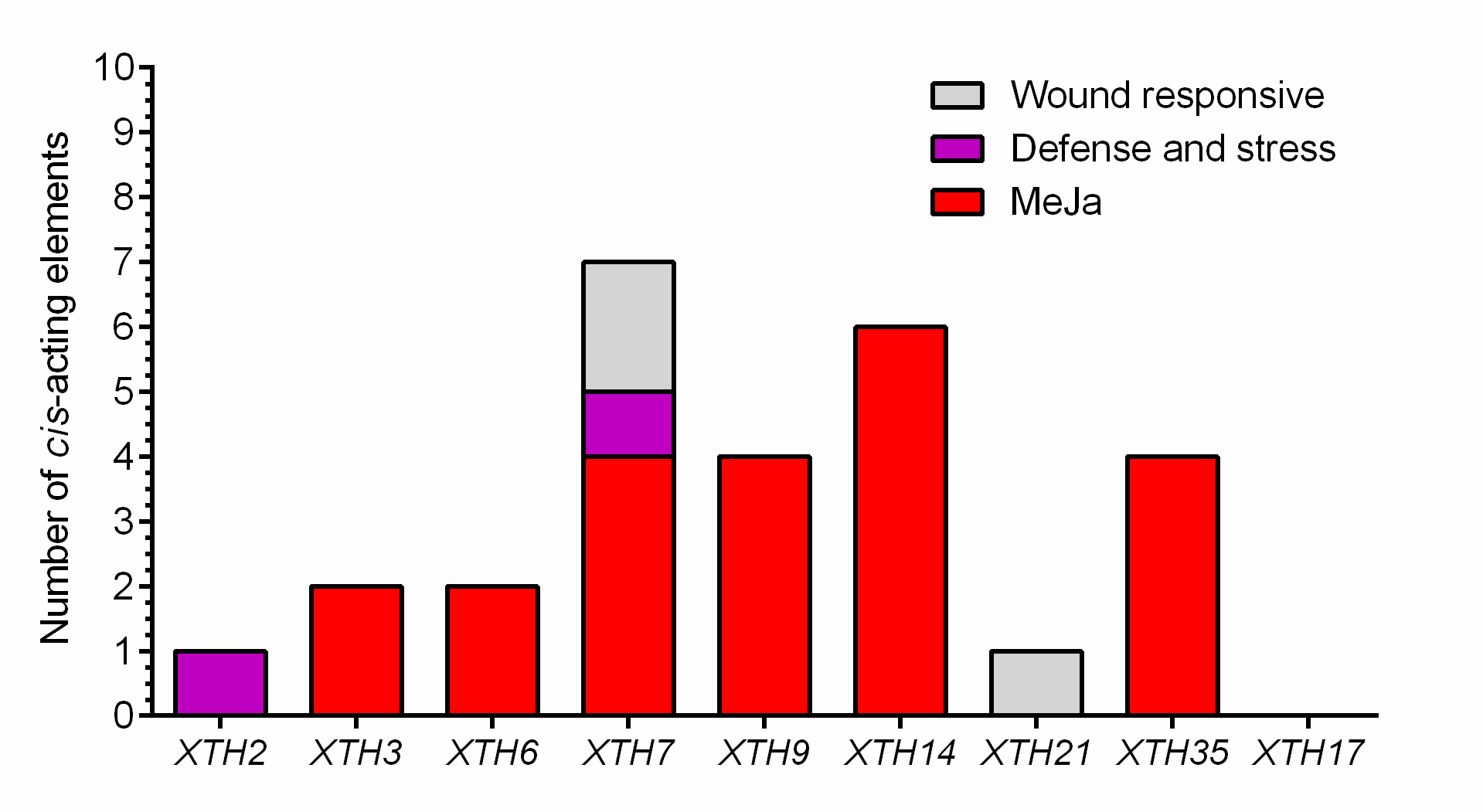

Supplement: Supplemental Information 21 — The bar graphs represent the total number of methyl jasmonate: MeJa, wound, and defense and stress-responsive elements, in each gene. [file peerj-11-15257-s021.png]
